# Medical hyperspectral imaging: an updated review of technology advancements and biomedical applications

**DOI:** 10.1117/1.JBO.31.3.030901

**Published:** 2026-03-20

**Authors:** Minh H. Tran, Ling Ma, Mandy Yuan, Baowei Fei

**Affiliations:** a University of Texas at Dallas, Center for Imaging and Surgical Innovation, Richardson, Texas, United States; b University of Texas at Dallas, Department of Bioengineering, Richardson, Texas, United States; c University of Texas Southwestern Medical Center, Department of Radiology, Dallas, Texas, United States

**Keywords:** medical hyperspectral imaging, optical imaging, spectral imaging, image analysis, cancer detection, disease diagnosis, intraoperative image guidance, pathology, machine learning, deep learning

## Abstract

**Significance:**

Hyperspectral imaging (HSI) is an advanced spectral imaging technique that captures spatial and spectral information across numerous wavelength bands. This capability allows tissue characterization, disease detection and diagnosis, surgical guidance, and digital histopathology, making it an increasingly valuable tool with wide biological and medical applications.

**Aim:**

We aim to provide readers with (1) an understanding of the principles and technological advancements in HSI, (2) a comprehensive overview of HSI data processing and analysis methods, and (3) an updated survey of biomedical applications, from disease detection, intraoperative imaging, to histopathology.

**Approach:**

A systematic literature search was conducted using PubMed and Google Scholar with the keyword “hyperspectral imaging.” We previously published a comprehensive review paper on medical HSI in 2014, which was widely cited in the field. Therefore, this updated review focused on new technology advancements and emerging applications. Based on their biological and medical relevance, 612 HSI papers were included and analyzed in this review.

**Results:**

Recent advances in HSI span both hardware and computational techniques, including improvements in sensor technology, data processing and analysis, short-wave near-infrared imaging, and deep-learning and AI tools. HSI is actively explored for various applications in oncology, neurology, ophthalmology, dermatology, cardiology, gastroenterology, hepatology, wound care, endocrinology, dentistry, infectious disease, plastic and reconstructive surgery, general surgery, intraoperative guidance, histopathology, microbiology, nanopathology, and pharmacology.

**Conclusions:**

HSI has become an emerging imaging modality in biomedical research and clinical settings. Continued advancements in hardware miniaturization, computational efficiency, and clinical validation will further solidify the role of next-generation HSI in biomedicine.

## 1 Introduction

Hyperspectral imaging (HSI) is an optical technique that acquires a three-dimensional dataset (a “hypercube”) with two spatial dimensions and one spectral dimension. Unlike conventional color imaging, which captures only broad red, green, and blue (RGB) channels, HSI collects dozens to hundreds of contiguous spectral bands, providing a detailed spectral signature at each pixel. The capability to combine imaging and spectroscopy enables the identification of materials and tissues based on their unique spectral fingerprints. HSI originated in NASA’s remote-sensing programs and was first used to map the Earth’s surface composition.^1^ Over the past few decades, it has transitioned from this remote-sensing heritage into many other domains. Today, HSI systems are employed in environmental monitoring and precision agriculture, defense and security, and industry for quality control and food safety inspection.^2^ These early successes across disciplines underscore the versatility of HSI and set the stage for its adoption in medicine.

In the biomedical field, HSI has evolved from an experimental concept into a popular modality for research and clinical applications. Pioneering studies in the late 1980s and early 1990s demonstrated that multispectral imaging (MSI) could assess tissue properties (e.g., determining burn wound depth),^3^ sparkling interest in optical spectral techniques for medicine. Over the years, HSI has shown promise for noninvasive disease detection, tissue characterization, and image-guided surgery.^4^ In recent years, significant advancements in HSI camera technology, computational power, and data-analysis algorithms have greatly accelerated the development of medical HSI. Modern HSI systems can acquire data faster and with higher spectral fidelity.^2^ New analysis techniques, especially those leveraging machine learning, enable more robust interpretation of complex spectral data. We published a comprehensive review paper on medical HSI in 2014,^4^ which was widely cited by the field. Given the rapid progress and the expanding range of clinical research using HSI, an updated review of the literature is necessary to capture the current state of the art. The objective of this paper is to provide a comprehensive and up-to-date guide on HSI in biomedicine, consolidating recent developments in technology, analytical methods, and biomedical applications.

The remainder of this paper is organized into five main sections. Section 1 summarizes our approaches for the literature survey and covers the fundamental optics concepts for new readers. Section 2 describes HSI system design and validation, including methods of acquisition, illumination, and configurations used for various biomedical purposes. Section 3 discusses computational techniques for data processing and algorithm development, including preprocessing steps, spectral feature extraction, band selection, spectral unmixing, and machine learning– and deep learning–based methods for classification and segmentation. Section 4 focuses on biological and medical applications, highlighting how this technology is applied in practice for diagnosis, surgical guidance, and other healthcare-related uses. Last, Sec. 5 provides perspectives on the advancement of HSI as well as challenges and future directions.

### 1.1 Scope and Methodology

The literature search was conducted using two electronic databases, PubMed and Google Scholar. The PubMed search was conducted for articles from January 2014 to May 2024 using the keyword “hyperspectral imaging.” HSI was defined as a technique that creates a three-dimensional (3D) data cube from capturing no less than 10 spectral bands. MSI papers were not included in the review, with a few exceptions, such as those papers that described MSI as having more than 10 bands or the ones that were used to trace the history of spectral imaging. Hyperspectral optical coherence tomography (OCT) and Raman HSI were excluded from this review because of their technological and mechanistic differences as compared with the definition of HSI herein. Review articles, tutorials, theses, book chapters, and HSI studies not closely related to biomedical applications were not included in the formal analysis, although some may have been cited for background information. We reviewed and included a total of 612 publications of original research, including peer-reviewed journal articles, conference papers, and proceedings in this review.

### 1.2 Basics of Light–Tissue Interaction

As the intended audience of this review paper not only includes researchers with backgrounds in optics and photonics but also those from biological and medical areas, a basic overview of biophotonics is necessary. This overview will discuss only matters relevant to hyperspectral imaging and provide some basic assumptions for later sections.

When light interacts with an object or tissue, it can be absorbed, reflected, scattered, diffracted, refracted, or transmitted through the material. By illuminating tissue with lights of broad wavelengths, HSI captures dozens or hundreds of images at different wavelength bands. The intensity at each pixel of the hyperspectral image represents the combination of light reflection, absorption, and scattering of the tissue. The spectral signature of tissue properties can then be used for the detection and diagnosis of different types of tissue and disease conditions. Medical HSI researchers primarily focus on reflection, transmission, absorption, and scattering of light within tissues.^5^

Light attenuation refers to how much light loses intensity when passing through a medium and is the sum of two components: absorption and scattering. In homogeneous materials, absorption and scattering are usually denoted by the absorption coefficient $\mu_{a}$ and scattering coefficient $\mu_{s}$. Both have units of $m^{-1}$. According to the Beer–Lambert law, the intensity of light I(x) passing through a uniform medium is exponentially decayed the further the light passes through the length x of the medium:

$$I(x)=I_{0}\;\exp(-\mu x)\approx I_{0}\;\exp(-(\mu_{a}+\mu_{s})x).$$

If absorption is much greater than scattering, the Beer–Lambert law can be used as an approximation of light transport. In those cases, absorption is linear, i.e., it scales up with the concentration of the mixture and with the length of the path the light takes. Furthermore, mixing multiple substances together adds absorbance, which is the basis for linear unmixing models (see Sec. 3.4). It is also common to use the wavelength-dependent molar absorption coefficient ε to identify biological molecules.

Scattering depends on wavelength, material, and particle size. Mie scattering models the interaction with large particles, whereas Rayleigh scattering describes scattering by small particles much smaller than the wavelength of light.^5^ Scattering is anisotropic, i.e., the amount of light scattered is not directed uniformly. To account for this, an anisotropic term g is introduced that ranges from −1 to 1, where g is the cosine of the mean scattering angle throughout the medium and is also wavelength dependent. With this anisotropic effect, the scattering coefficient is replaced by a reduced scattering coefficient $\mu_{s}^{\prime }$ using the equation $\mu_{s}^{\prime }=\mu_{s}(1-g{)}^{2}$. Depending on the complexity and availability of data, light transport models can also consider the refractive index of different tissues as refraction modifies the path length of light. Jacques et al.^5^ compiled a review of scattering coefficients, anisotropy factors, and absorption values of various types of tissues in humans and other mammals. They fit the reduced scattering to the following equation ^5^^,^^6^:

$$\mu_{s}^{\prime }(\lambda )=a^{\prime }(f_{\text{Mie}}(\frac{\lambda }{500\;(\mathrm{nm})}{)}^{-b_{\text{Mie}}}+f_{\text{Rayleigh}}(\frac{\lambda }{500\;(\mathrm{nm})}{)}^{-4}),$$

where $f_{\text{Mie}}=1-f_{\text{Rayleigh}}$.

In this equation, $a^{\prime }$, $f_{\text{Mie}}$, $f_{\text{Rayleigh}}$, and $b_{\text{Mie}}$ are all coefficients that are supposed to be unique to each biological material. Note that in their equation, as well as in observational experiments, scattering is lower the higher the wavelengths. This contributes to the observation that near-infrared light penetrates deeper into tissue compared with visible light.

## 2 System Design and Validation

We published a comprehensive review paper on medical HSI in 2014,^2^ which summarized the major progresses and applications of HSI and was widely cited in the field. This updated review focuses on new literature and uses a similar structure and categories for recent advances, such as polarized hyperspectral imaging (PHSI), hyperspectral endoscopy, hyperspectral handheld systems, the use of augmented reality (AR) with HSI, and the validation of HSI systems using biological phantoms. Table 1 summarizes the representative HSI systems and their medical applications. Systems that use a commercial device without modification or customization and those without technical details are not included in the table.

**Table 1:** Summary of representative hyperspectral imaging systems and their biological and medical applications.

Ref	Acquisition mode	Spectral range (nm)	Bands	Light source	Detector	Dispersive device	Measurement mode	Application
7	Spatial scanning	200 to 430	>461	Deuterium	CMOS	Convex grating	Transmission; microscopy	UV range biomolecule and tissue analysis
8	Spatial scanning	350 to 1100	151	Halogen	—	Diffraction grating	Transmission; microscopy	Pancreatic cancer
9	Spatial scanning	400 to 800	∼160	Halogen; LED	EMCCD	Diffraction grating	Reflection; endoscopy	Gastrointestinal tract
10	Spatial scanning	400 to 700	31	LED	sCMOS	Diffraction grating	Transmission; brightfield microscopy	Spatial resolution enhancement; histopathology
11	Spatial scanning	350 to 1000	>200	Tungsten halogen	—	PGP	Transmission; brightfield microscopy	Gastric cancer
12 ^,^ 13	Spatial scanning	400 to 1000	826	Halogen	CCD	PGP	Transmission; brightfield microscopy	Brain cancer histopathology
14	Spatial scanning	430 to 750	272	LED	CCD	PGP	Reflection	Diabetic foot ulcers
15	Spatial scanning	400 to 1000	∼215	Halogen; mercury	EMCCD	PGP	Autofluorescence; microscopy	Dental plaque bacteria
16	Spatial scanning	500 to 1000	100	Halogen; LED; Xenon	—	Transmission grating	Reflection; laparoscopy	Laparoscopic surgery
^17^	Spatial scanning	400 to 1000	270	Xenon	CCD	**—**	Reflection; endoscopy	Laryngeal surgery
18–20	Spatial scanning	400 to 1000	601	LED	CMOS	**—**	Reflection	Peritoneal fibrosis; peritonitis; tissue characteristics
21	Spatial scanning	400 to 1000	218	Tungsten halogen	CMOS	**—**	Reflection	Custom scanning HSI system development
22	Spatial scanning	400 to 1000	756	Broadband	EMCCD	**—**	Reflection; endoscopy	Endoscopic bioimaging
23	Spatial scanning	881 to 1710	510	Halogen	InGaAs CCD	**—**	Reflection	Allergic contact dermatitis
24	Spatial scanning	400 to 1000	408	Halogen	—	—	Transmission; brightfield microscopy	Hepatocellular carcinoma
25	Spatial scanning	440 to 1023	277	Halogen	—	—	Transmission; brightfield microscopy	Infection pathogens
26	Spectral scanning	600 to 800	101	Halogen	CCD	AOTF	Transmission; brightfield microscopy	Cervical intraepithelial neoplasia
27,28	Spectral scanning	550 to 1000	60	Halogen	CCD	AOTF	Transmission; brightfield microscopy	Liver tumor; melanoma
26,29	Spectral scanning	600 to 800	101	Xenon	CCD	AOTF	Reflection	Cervical intraepithelial neoplasia
30	Spectral scanning	450 to 850	89	Halogen	CMOS	AOTF	Reflection	Skin erythema
31	Spectral scanning	450 to 700	51	Xenon	EMCCD	AOTF	Fluorescence microscopy	Tissue autofluorescence
32	Spectral scanning	900 to 1700	22	Incandescent	InGaAs	AOTF	Reflection	Polarizer-free AOTF imaging
33	Spectral scanning	480 to 900	22	Halogen with monochromator	CMOS	Diffraction grating	Reflection	Iris imaging
34	Spectral scanning	405 to 665	27	Xenon	CCD	Filter wheel	Reflection; flexible endoscopy	Colorectal cancer
35	Spectral scanning	400 to 850	19	Xenon	**—**	Filter wheel	Reflection; surgical microscopy	Intraoperative procedures
36	Spectral scanning	550 to 885	76	Halogen	—	FPI	Reflection	Skin cancer; Lentigo maligna
37	Spectral scanning	450 to 850	120	Halogen	—	FPI	Reflection	Skin erythema; hyperpigmentation
38,39	Spectral scanning	477 to 891	33	LED	—	FPI	Reflection	Skin tumors; pigmentation; handheld
40	Spectral scanning	400 to 750	—	Halogen with monochromator	—	Grating	Transmission; microscopy	Histology imaging
41	Spectral scanning	420 to 730	63	Halogen	—	LCTF	Transmission; brightfield microscopy	Neurons; glial cells
42	Spectral scanning	900 to 1200	31	Incandescent; laser	—	LCTF	Photoluminescence	Nanoprobes
43	Spectral scanning	380 to 780	201	Tunable light source	CCD	—	Reflection	Tissue topography; oxygen saturation
		400 to 720	—	Broadband		LCTF		
44	Spectral scanning	400 to 720	65	Halogen	CCD	LCTF	Reflection; surgical microscopy	Ischemic stroke
45	Spectral scanning	400 to 1100	141	Halogen	CCD	LCTF	Reflection	Renal cancer; breast cancer; lung cancer
46	Spectral scanning	500 to 600	26	Halogen	CCD	LCTF	Reflection; scattering; microscopy	Breast cancer
47	Spectral scanning	400 to 1100	141	Halogen	CCD	LCTF	Reflection	Operating room system; tissue characterization
		950 to 1700	72		InGaAs CCD			
48,49	Spectral scanning	500 to 620	13	LED	CCD	LCTF	Reflection; stereomicroscopy	Microvasculature
50	Spectral scanning	500 to 620	13	Xenon	CCD	LCTF	Reflection; laparoscopy	GI tract
51	Spectral scanning	400 to 720	33	Xenon	CCD	LCTF	Reflection	Inflammatory hyperemia
52	Spectral scanning	460-660	101	Halogen; Xenon + filter	CMOS	LCTF	Transmission; fluorescence; microscopy	Intestinal fungi
53,54	Spectral scanning	400 to 700	31	LED	CMOS	LCTF	Reflection	Full–face skin analysis
55,56	Spectral scanning	450 to 680	116	Xenon + filter	CMOS	LCTF	Fluorescence microscopy	Gastric cancer
57	Spectral scanning	600 to 720	—	Xenon + filter	CMOS	LCTF	Fluorescence; surgical microscopy	PpIX; brain tumor surgery
58	Spectral scanning	540 to 650	12	Tungsten	EMCCD	LCTF	Darkfield microscopy	Membrane receptors; nanoparticles
59	Spectral scanning	950 to 1800	86	Halogen	InGaAs CCD	LCTF	Transmission; brightfield microscopy	Tissue lipid visualization
60	Spectral scanning	420 to 720	151	Halogen	sCMOS	LCTF	Transmission; brightfield microscopy	Alzheimer’s disease; retinal amyloid beta-protein
61,62	Spectral scanning	420 to 730	63	LED	sCMOS	LCTF	Reflectance; surgical microscopy	Malignant gliomas
			104				Fluorescence; surgical microscopy	
63	Spectral scanning	470 to 720	31	LED; laser	sCMOS	LCTF	Fluorescence; cryo-imaging	Whole-body fluorescence imaging
64	Spectral scanning	460-660	74	Halogen	CMOS	Prism	Transmission; microscopy	Whole-slide histology imaging
65	Spectral scanning	600 to 894	11	Supercontinuum laser	sCMOS	Prism	Reflection	Biomarkers in cerebral cortex
66,67	Spectral scanning	360 to 550 (excitation)	39	Xenon	EMCCD	TFTF (after light source)	Autofluorescence; excitation-scanning fluorescence microscopy	Colorectal cancer; tissue characterization
68	Spectral scanning	420 to 720	16	—	—	Tunable filter	Transmission; brightfield microscopy	Lung cancer; immunotherapy
69	Spectral scanning	844 to 1452	152	Laser	InGaAs	VBG	Fluorescence microscopy	Nanotubes
70	Spectral scanning	420 to 730	311	LED	CCD	**—**	Reflection	Pigmented skin lesions
71–73	Spectral scanning	390 to 680	30	Monochromator	CCD	**—**	Reflection; endoscopy	Laryngeal cancer
74	Spectral scanning	350 to 950	21	Sequential scanning LEDs	CCD	**—**	Reflection; dermoscopy	Melanoma
75,76	Spectral scanning	450 to 900	91	Tunable light source	CCD	**—**	Reflection; fundus camera	Diabetic retinopathy; Alzheimer’s disease; cerebral amyloid
77,78	Spectral scanning	468 to 857	33	Tunable Xenon	CCD	**—**	Reflection; dermoscopy	Nevi; melanin
79	Spectral scanning	420 to 660	—	Sequential scanning LEDs	CMOS	**—**	Reflection	Skin erythema; skin physiology
80	Spectral scanning	380 to 1000	36	Sequential scanning LEDs	CMOS	**—**	Reflection	Mobile HSI
81–83	Spectral scanning	334 to 495 (excitation)	18	Sequential scanning LEDs	EMCCD	**—**	Excitation scanning fluorescence microscopy	Cellular metabolism; articular cartilage
84	Spectral scanning	830 to 870	80	Tungsten halogen w/ monochromator	EMCCD	**—**	Transmission; microscopy	Extracellular vesicle molecular profiling
85	Spectral scanning	1100 to 1700	—	Optical parametric oscillator	InGaAs	**—**	Reflection	Liver cancer
86,87	Spectral scanning	320 to 680	361	Xenon + monochromator	sCMOS	**—**	Reflection; ophthalmoscopy	Alzheimer’s disease; Parkinson’s disease
88	Spectral scanning	480 to 705	16	Halogen with monochromator	**—**	**—**	Reflection; endoscopy	Alzheimer’s disease; amyloidopathy
89,90	Spectral scanning	420 to 720	31	Mercury	**—**	**—**	Autofluorescence; epi-fluorescence microscopy; excitation/emission	Macular degeneration
91	Spectral scanning	405 to 910	18	Sequential scanning LEDs	**—**	**—**	Reflection; endoscopy	GI cancer; catheter
92	Spectral scanning	380 to 780	41	Tunable Xenon	—	**—**	Transmission; brightfield microscopy	WSI devices; color accuracy assessment
93	Spatial–spectral scanning	470 to 900	150	—	CMOS	FPI	Transmission; brightfield microscopy	Whole-slide imaging; head and neck cancers
94	Spatial–spectral scanning	470 to 900	150	LED; halogen	CMOS	FPI	—	Hyperspectral camera innovation
95	Spatial–spectral scanning	470 to 780	104	Xenon	CMOS	FPI	Reflection; surgical microscopy	Brain tumor; intraoperative guidance
96,97	Spatial–spectral scanning	458 to 904	151	Halogen	**—**	—	Transmission; brightfield microscopy	Lymphoma; myocarditis; mucosal oral melanoma
98	Spatial–spectral scanning	470 to 750	97	Halogen	**—**	—	Transmission; scattering; brightfield microscopy	Head and neck cancer; collagen
99	Snapshot	460 to 870	41	Halogen	**—**	Filter	Reflection; surgical microscopy	Tissue and oxygenation characterization; intraoperative guidance
100	Snapshot	350 to 1000	155	Xenon	—	Filter	Reflection	Brain tumor surgery
35	Snapshot	463 to 638	16	Xenon	**—**	Filter	Reflection; surgical microscopy	Intraoperative procedures
		693 to 966	25					
101	Snapshot	460 to 620	16	—	CMOS	Filter	Reflection; fundus camera	Alzheimer’s disease
102	Snapshot	470 to 630	16	LED	—	FPI	Reflection	Microcirculation; skin oxygen saturation
103,104	Snapshot	460 to 630	16	—	CMOS	FPI	Reflection; fundus camera	Retinal vessels
105	Snapshot	500 to 900	—	Halogen	CMOS	FPI	Reflection	Skin properties
106	Snapshot	665 to 960	24	Halogen	CMOS	FPI	Transmission	Blood component analysis
107	Snapshot	463 to 648	41	LED; laser	CMOS	FPI	Reflection; endoscopy	Esophageal imaging; simultaneous bimodal imaging
		659 to 891					Fluorescence; endoscopy	
108	Snapshot	481 to 632	16	Xenon	CMOS	FPI	Reflection; surgical microscopy	Epilepsy; intraoperative brain hemodynamics
109	Snapshot	460 to 600	16	Xenon	CMOS	FPI	Reflection; endoscopy	High-resolution retinal imaging
110,111	Snapshot	500 to 900	78	Xenon; LED	CMOS	FPI	Reflection; surgical microscopy	Glioma surgery
112	Snapshot	450 to 650	24	LED	—	Prism	Endomicroscopy	Tissue characteristics measurements
113,114	Snapshot	470 to 670	43	Camera flash	CCD	Prism	Reflection; fundus camera	Retinal angiography
115	Snapshot	484 to 652	38	Quartz tungsten halogen	CCD	Prism	Reflection	Cortex intrinsic signals
116	Snapshot	515 to 842	46	Halogen	sCMOS	Prism	Fluorescence; reflection; microscopy	Live cellular processes
117	Snapshot	480 to 660	60	Laser	sCMOS	Prism	Fluorescence; microscopy	Pancreatic islet
118,119	Snapshot	450 to 950	125	Quartz tungsten halogen	CCD	—	Reflection	Skin cancer; pigmented skin lesions
120	Snapshot	400 to 750	250	Spectral encoding module	CMOS	—	Transmission; microscopy	Histology imaging
121	Snapshot	460 to 870	31	Halogen	**—**	**—**	Reflection; laparoscopy	Abdominal surgery
122	Snapshot	461 to 641	31	Halogen	**—**	**—**	Transmission; brightfield microscopy	Ductal carcinoma *in situ*
123	Snapshot	464 to 636	16	LED	**—**	**—**	Fluorescence	Oral tissue

### 2.1 Image Acquisitions

**Fig. 1:**
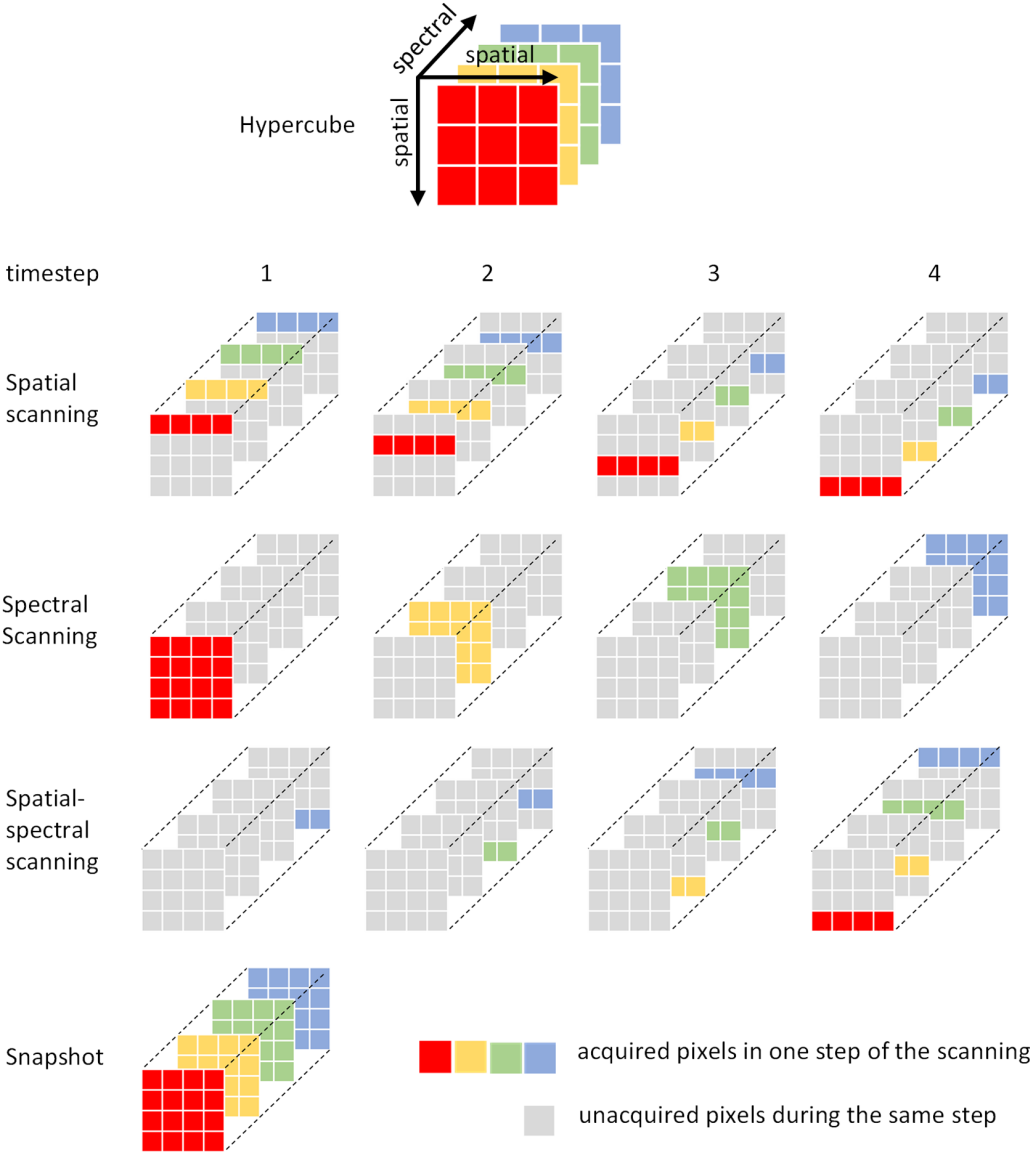
Illustration of common hyperspectral image acquisition strategies, including spatial scanning, spectral scanning, spatial–spectral scanning, and snapshot imaging. The hypercube consists of two spatial dimensions and one spectral dimension, where the depth (stacked layers) represents discrete wavelength bands. Red, yellow, green, and blue colors denote different spectral bands along the spectral dimension. Colored pixels indicate pixels that are acquired during a single acquisition step, whereas gray pixels represent spatial–spectral locations not acquired during that step. Timesteps indicate the sequential order of data acquisition used to construct the full hypercube.

We identify four main mechanisms of hyperspectral image acquisition: spatial scanning, spectral scanning, spatial–spectral scanning, and snapshot imaging. Briefly, spatial scanning captures the spectra one or several pixels at a time using a moving mechanism to cover the entire space. Spectral scanning captures the entire spatial field, one or several spectra at a time, using an optical dispersive mechanism. Spatial–spectral scanning captures both spatial and spectral data by the movement of a sensor integrated with filters. Snapshot imaging captures the entire hypercube within a single exposure. Figure 1 shows a diagram of different acquisition techniques. More details on each scanning method are described in the following section.

#### 2.1.1 Spatial scanning

Spatial scanning is commonly called “push-broom” or “whiskbroom,” depending on how much of the spatial field of view (FOV) is acquired at a time.^124^^,^^125^ The optical layout of spatial scanning cameras consists of a small aperture, a refractive or diffractive optical component, and a digital sensor at the receiving end of the optical component. Light passing through the aperture gets collimated (directed into parallel rays), then diffracted or refracted, and finally focused onto the sensor, where it is converted into spectral measurements. Spatial imaging systems can scan across a broad wavelength range with high spectral and spatial resolution. There are two main configurations of spatial scanning HSI systems: reflective-based scanning and transmission-based scanning. Examples of reflective-based scanning systems include the Offner spectrometer^12^^,^^126^^–^^133^ and Czerny–Turner reflective grating spectrometer.^2^ In an Offner configuration, the collimator is a concave mirror, and the diffractor is a reflection grating mirror. The optical components of the Offner spectrometer can be manufactured as a single monolithic block of glass, producing a very compact and lightweight system. One such system is commercially available, which has 826 spectral bands ranging from 400 to 1000 nm and is widely used in biomedical research.^134^^–^^138^ In a Czerny–Turner configuration, reflection grating and focusing components can be flat, simplifying the construction compared with the Offner configuration, but at the expense of increased spectral artifacts.^2^ Nevertheless, several authors constructed customized systems using the Czerny–Turner configuration, such as Stergar et al.^40^ Examples of transmission-based scanning systems include transmission gratings, holographic prisms, and prism–grating–prism (PGP) architectures.^2^^,^^139^^–^^142^ Notably, PGP architectures can be used to construct compact spectrometers, which in turn can be used for spatial scanning HSI cameras. PGP consists of one transmission grating sandwiched between two prisms. One common system is a commercially available spectrograph, which can capture 205 bands from 400 to 1000 nm. This particular spectrograph is widely used in biomedical research.^143^^–^^145^

Spatial scanning HSI cameras need the optical system to move to scan the FOV. There are two ways to achieve this: by relative movement between the camera and the object or by internal scanning mechanisms. The first method is used by older systems and was achieved by placing the entire camera, or the object, on a linear moving stage. For example, in the system used by Leon et al.,^137^ the camera was placed on a moving motor, which in turn was placed on a robotic arm within the surgical room. If such a system is used for histological imaging, then a motorized stage moves the sample in relation to the camera instead, like the systems used by Urbina Ortega et al.^135^ or Quintana-Quintana et al.^136^ In both cases, the motions of the camera (or the sample) have to be timed correctly in relation to the frame rate of the camera. Such movements mean the acquisition system takes up more space. More novel designs use internal mechanisms to either move the sensor or manipulate the optical paths to scan the entire FOV. Yan et al.,^15^ for example, used a stepping motor to translate a relay lens, which in turn translated the location where the light landed on the sensor. Gutiérrez-Gutiérrez et al.^21^ used a rotating mirror to scan the output of a spectrograph onto the sensor.

#### 2.1.2 Spectral scanning

Spectral scanning systems use filter wheels, electronically tunable filters, or tunable light sources such as spectrally programmable light-emitting diode (LED) arrays or monochromators. Filter wheels are not commonly used to generate hyperspectral images due to the limited number of wavelengths they can produce.^2^ Instead, hyperspectral scanners predominantly use tunable filters such as acousto-optic tunable filters (AOTFs), liquid crystal tunable filters (LCTFs), interferometer-based filters such as the Fabry–Pérot interferometers (FPIs), or the Fourier transform imaging spectrometers (FTISs). Both AOTF and LCTF use crystals that bandpass light based on input signals. The key difference is that LCTFs are tuned through electrical signals, whereas AOTFs are tuned using acoustic waves at radio frequencies. AOTF and LCTF were introduced early in the history of spectral imaging and are more commonly seen in biomedical applications.^26^^,^^27^^,^^32^^,^^146^^–^^150^ Fourier transfer spectrometer with interferometers^151^ and FPI tunable filters were also investigated for spectral scanning.^105^^,^^152^^–^^154^

#### 2.1.3 Spatial–spectral scanning

Spatial–spectral scanning systems use filters that vary their pass-through wavelengths based on their location within the sensor. There are two ways to achieve this. The first is by linear variable filters (also called wedge filters). Linear variable filters consist of two dielectric layers with a spacing that gradually increases along the filter’s length. This makes the filter’s pass-through wavelengths vary as a function of position along its main axis. The second way is by manufacturing miniscule FPIs on top of the sensor so that the pass-through wavelengths are dependent on the length along the sensor’s main axis.^94^ To acquire the hypercube, the sensor is translated along the sample. This creates the effect that the hypercube is acquired diagonally. One example system uses an FPI manufactured directly on top of the sensor and is available commercially.^94^ This spatial–spectral scanning system has been used in microscopy imaging by Tran et al.,^93^ Ma et al.,^155^^,^^156^ Zhou et al.,^98^^,^^157^^,^^158^ Laimer et al.,^96^ Willenbacher et al.,^97^ and Giannantonio et al.^95^

#### 2.1.4 Snapshot scanning

Snapshot imaging cameras can capture the entire hypercube within a single exposure. There are multiple ways to achieve this, as described in the review paper of Hagen et al.^159^ Here, we review the most common snapshot imaging methods, including spectrally resolved detector arrays (SRDAs), image mapping spectrometers (IMSs), microlens arrays, and compressive sensing (CS).

An SRDA consists of a layer of spectral filters placed on top of the camera sensor. FPI filters are often used because they have very narrow full width at half maximums (FWHMs) and accurate center wavelengths. The filters can be laid out in a square grid of size 4×4, or 5×5.^160^^,^^161^ Cameras made with the SRDA technology can be compact and can capture hyperspectral images in real time. They have been used in many biomedical applications, including fundus imaging, skin imaging, fluorescence imaging, endoscopy, and surgery.^99^^,^^101^^,^^123^^,^^162^^–^^164^ However, the image resolution is usually low, and the narrow FWHM of FPI filters means that the scene must be well illuminated.

IMS is a technique that uses thin strips of mirrors to “slice” the incoming image. The image is then projected onto different parts of the sensor and digitally reconstructed. IMS systems can capture images at more wavelengths compared with those using SRDA.^113^^,^^116^^,^^117^ Dwight et al.^113^^,^^114^ constructed a hyperspectral fundus camera using IMS principles. Their system is capable of capturing 43 wavelength bands from 470 to 670 nm. Konecky et al.^115^ used an IMS system that could capture 38 wavelength bands from 484 to 652 nm.

A microlens array uses a set of lenslets (small lenses) to “duplicate” the incoming image. The array of images then goes through a monochromator to produce images at different spectra. One such system was developed for microscopy by Yu et al.^165^ In recent years, another concept called hyperspectral light field imaging was developed using a microlens array.^166^ Because each microlens within the array captures the object at a slightly different angle, the resulting image contains not only spectral information but also angular information of the incoming light. As such, hyperspectral light field imaging captures four dimensions of data (two spatial dimensions, a depth dimension, and a spectral dimension). MacCormac et al.^100^ used a commercial light field hyperspectral camera during brain tumor surgery to evaluate feasibilities.

CS operates on the assumption that hyperspectral images can be compressed far beyond the Nyquist rate estimated from their bandwidth. If this assumption is true, HSI can be represented on the basis that the majority of information is concentrated. One way to convert the hypercube to another basis is through modulation (encoding the incoming light with another signal). In recent works, researchers used aperture masks (a series of printed masks placed at the camera aperture that let light in at selected patterns) to modulate the signal.^112^^,^^120^^,^^167^ When the sensor captured the encoded data, it acquired a higher amount of information than the resolution of the sensor allowed. To extract this higher order of information, reconstruction algorithms or neural networks were used to reconstruct the hypercube from the encoded data. Meng et al.^112^ used a deep U-Net to reconstruct from the encoded data. Klein et al.^167^ and Oiknine et al.^120^ both characterized the encoding process to reconstruct the hypercube.

#### 2.1.5 Comparison of acquisition methods

Each scanning method has tradeoffs in terms of image resolution, number of spectral bands, and acquisition time. Under proper illumination conditions, they all can provide spatial and spectral information for specific applications.^168^ Spatial scanning methods such as push-broom and spectral scanning methods using LCTF or AOTF can have high spatial and spectral resolution but relatively slow acquisition speed. Because snapshot cameras do not have a “scanning” component, they must project the entire hypercube onto the sensors; thus, the snapshot trades off spatial and spectral resolution for fast acquisition.

Push-broom, spectral scanning, and snapshot cameras can all be configured to capture images in the visible or infrared light range. High-quality push-broom cameras can have a FWHM of 5 to 10 nm.^169^^,^^170^ AOTFs can have similar FWHM in the range 1 to 5 nm,^171^^,^^172^ whereas LCTFs often have a wider FWHM of 5 to 20 nm.^171^^,^^173^ FPIs also have large FWHM, ranging from 5 to 30 nm.^170^^,^^174^^,^^175^ More detailed analysis and comparison were described in our previous paper.^2^

Another point of comparison is the acquisition speed. If the acquisition target is *in vivo*, fast acquisition is important to avoid motion blur. For spatial scanning systems, the acquisition speed is often slow because scanning is done using mechanical movements. On the other hand, in spectral scanning systems such as LCTF, AOTF, and FPI, switching between filters is often performed by varying electrical signals, which makes them much faster. LCTFs have varied switching times, ranging from 50 to 500 ms per wavelength.^176^ FPIs are slightly faster, with an estimated switching time of around 2 ms.^177^ AOTFs have response times reported to be in the nanoseconds or microsecond scales.^178^ Snapshot imaging cameras are often instantaneous, and SRDA can acquire images at frame rates similar to those of RGB video cameras. On the other hand, the physical dimensions and weights of HSI cameras have been remarkably reduced in recent years, especially snapshot cameras fabricated using FPI sensors. They can be of comparable size to RGB sensors. One such camera, a snapshot imaging system capable of capturing 25 spectral bands in real time, weighs only 400 g.^103^

### 2.2 Illumination and Optical Setups

Multiple factors need to be considered when applying HSI for specific applications. The first factor is the wavelength. Visible wavelengths (∼400 to 700 nm) are widely used for biological applications, including detecting hemoglobin that has notable peaks within this range.^5^ Visible wavelengths are also suitable to detect melanin and colored dyes such as hematoxylin and eosin (H&E). Near-infrared (NIR, 700 to 2500 nm) wavelengths have higher penetration depth compared with visible light and are used in applications that involve lipids or water. Visible wavelengths can be used with regular complementary metal-oxide-semiconductors (CMOS) or charge-coupled device (CCD) sensors, whereas NIR wavelengths require the use of the more expensive indium gallium arsenide (InGaAs) as the sensor material.^4^ A study by Zhang et al.^179^ demonstrated that deep tissues show high spatial contrast using the NIR wavelengths within 1300 to 1375 nm and 1550 to 1600 nm. Another study by Shahzad et al.^180^ demonstrated that 800 to 850 nm wavelengths serve as the optimum illumination range for venous imaging regardless of skin tone types.

The second factor is the light sources. Halogen light can provide illumination in the visible light range, extending into the NIR range. However, there are significant disparities in the power delivered at blue wavelengths (∼400  nm) compared with red wavelengths (∼600  nm), which may lead to spectral imbalances. Xenon light is more uniform in the visible light range; however, it has strong, nonuniform peaks beyond 800 nm. LEDs provide varied illuminations at different wavelengths at low cost. Because of this, LEDs can be used to provide illumination at specific wavelengths for HSI applications.

**Fig. 2:**
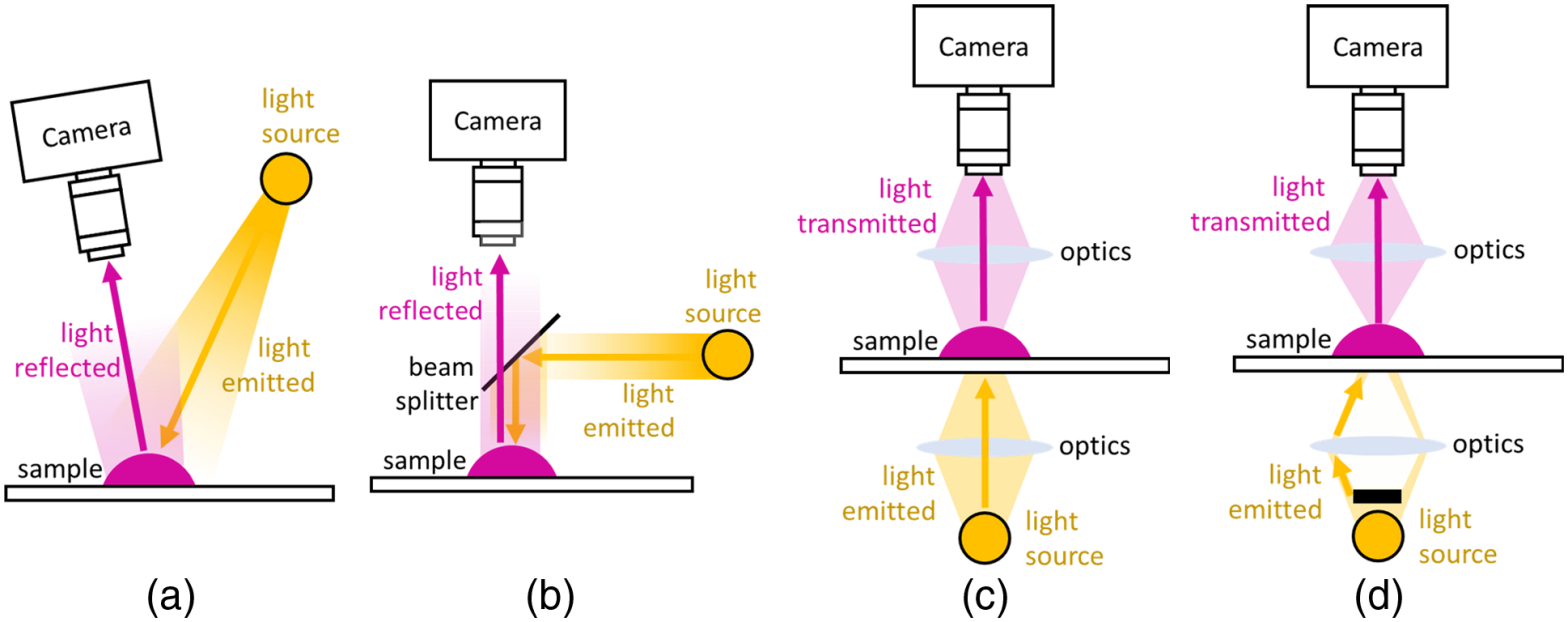
Different lighting configurations for biomedical hyperspectral imaging: (a) frontal, direct illumination, (b) co-axial illumination, (c) transmission light-field microscopy illumination, and (d) darkfield microscopy illumination.

The third factor is the optical setup. Biomedical imaging often uses four types of setups: brightfield illumination, co-axial illumination, transmission brightfield microscopy, and transmission darkfield microscopy (Fig. 2). Brightfield illumination is commonly used for gross *ex vivo* specimens or during surgical settings. Co-axial illumination is used for imaging highly reflective objects^43^^,^^181^^,^^182^ and can be achieved with a beam splitter, a ring light, or dedicated optical systems such as endoscopes. Transmission illumination lit the subject from behind and is commonly seen in transmitted light microscopy. Darkfield illumination, which results in a dark background and bright objects by blocking direct illumination and making only scattered light seen, is commonly used in hyperspectral darkfield microscopic imaging.

The fourth factor is uniformity, including spatial uniformity (differences between well-illuminated and non-illuminated regions), angular uniformity (occurrences of cast shadows due to the angles of the light), and spectral uniformity (variation in spectral power density between different light sources). A study by Sawyer et al.^183^ evaluated different setups in terms of uniformity and found that a diffuse scattering dome achieves high angular uniformity, but an illumination ring (co-axial) achieves spatial and spectral uniformity. Hyperspectral cameras often have lower throughput compared with RGB cameras, so they might need longer exposure time or higher illumination. This is important because some tissues are sensitive to light exposure. For example, the retina is a particularly sensitive structure within the eye. Exposure to the retina in humans should therefore not exceed ∼180  μW for 5 s or ∼120  μW for 30 s.^2^ In measuring the illumination power, we recommend using the term radiance (measured in Watts) instead of luminance (measured in lux). Even though they both measure the same physical quantity, radiance encompasses the full electromagnetic spectrum, including both the visible and the NIR range. Luminance, on the other hand, is more commonly used in the domain of visible light systems.

The fifth factor is glare. Glare is an unwanted effect caused by the specular reflection of light into the sensor. The glare effect index was introduced to quantify the amount of glare in HSI data.^184^ In biomedical research, glare is common due to the moist tissue surfaces and can reduce the overall classification accuracy.^185^ To reduce or eliminate glare, it is better to address it during the acquisition stage rather than the pre-processing stage. Positioning the light to reduce glare is one of the simplest methods. For example, the incoming light can be placed at an angle of 45 deg relative to the camera.^160^ Sometimes, the anatomical features of the targets should also be considered. Di Cecilia et al.^33^ used the geometry of the iris to find the position of light that reduces Purkinje images (reflection of the light inside the eyes). Because reflected lights are often linearly polarized, lens polarizers can also be used to reduce glare.^102^

### 2.3 Hyperspectral Surgical Microscope

Surgical microscopes are major equipment widely utilized in various surgical applications, particularly in neurology. Integrating HSI with surgical microscopes allows for seamless incorporation of spectral information in the operating room, eliminating the need for bulky additional instrumentation while leveraging the sophisticated optical design of the microscopes. In addition, HSIs can be versatile, supporting both reflectance imaging^186^ and contrast enhancement in fluorescence imaging.^57^

**Fig. 3:**
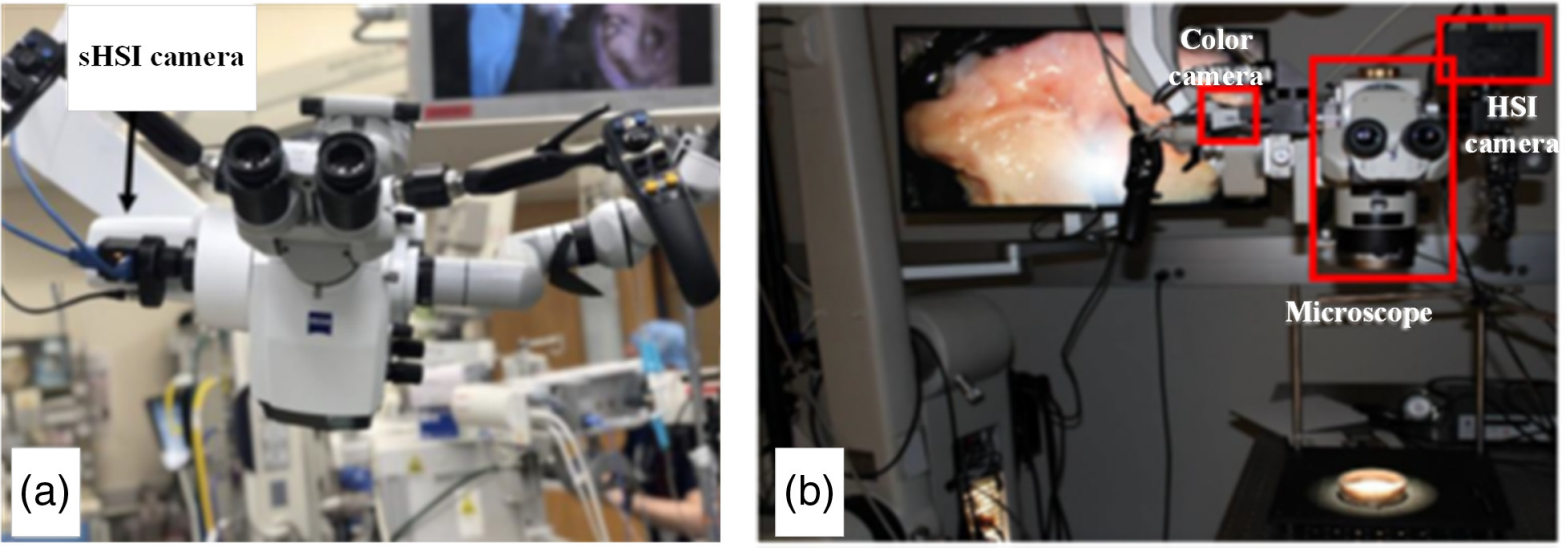
Hyperspectral imaging surgical microscopes. (a) A compact VIS-NIR snapshot hyperspectral camera mounted on a surgical microscope. Figure reproduced with permission from Kifle et al.^187^ (b) A dual-camera surgical microscope for high-resolution hyperspectral image guidance with super-resolution reconstruction. Figure reproduced with permission from Ma et al.^156^

Snapshot hyperspectral cameras are compact and lightweight, making them easy to integrate into surgical microscopes via a video adapter, as shown in Fig. 3(a). Their high-speed imaging capability is optimal for hemodynamic monitoring in neurological surgeries. For example, Pichette et al.^108^ mounted a 16-band snapshot camera on the side port of a surgical microscope for intraoperative hemodynamic response assessment. The camera covered a 481 to 632 nm spectral range with an image resolution of 256×512  pixels, and it operated at 20 frames per second (fps) during intraoperative imaging. Furthermore, a multimodal imaging surgical microscope was developed by Lee et al.^99^ incorporating laser speckle contrast imaging and snapshot HSI, leveraging the speed and compactness of modern cameras. With advancements in hyperspectral imagers, such as the ones with tunable FPI, snapshot hyperspectral cameras now offer wider spectral range and improved spectral resolution, making them more suitable for intraoperative imaging in surgical microscopy.^110^^,^^111^^,^^187^

Spectral-scanning HSI systems consist of a monochromatic sensor and a spectral-scanning component, which can be either an electrical tunable filter^44^^,^^57^ or a filter wheel.^35^ When a tunable filter is used, the imaging system, including a tunable filter and monochromatic sensor, is mounted on one of the microscope’s optical ports via a video adapter. This form of HSI system enables the utilization of tunable filters with high spectral resolution and potentially selectable bands, together with monochrome cameras with high spatial resolution, depending on the specific application requirements. In addition, because LCTFs have an inherent nature of linear polarization, an optional linear polarizer can be placed in the incident path of the microscope light, cross-polarized with the tunable filter, to suppress surface reflections.^188^ For filter wheel-based systems, the filter wheel can be positioned either after the illuminator or in front of the imaging sensor. Wisotzky et al.^189^ utilized a digital surgical microscope equipped with a filter wheel containing 19 bandpass filters, enabling hyperspectral imaging in the 400 to 700 nm range. The wheel was positioned after the light source, allowing specific wavelength bands to be selected before the filtered light was transmitted through a fiber guide to the microscope optics to illuminate the surgical site.

Spatial–spectral scanning is a relatively new format of hyperspectral cameras, offering high spatial and spectral resolution while remaining compact and eliminating the need for external scanning mechanics. This makes integrating a spatial–spectral hyperspectral camera with a surgical microscope^95^ an attractive alternative to spectral-scanning HSI options. However, due to its internal scanning mechanism, image acquisition time remains a limiting factor for its widespread adoption in operation theaters.^95^ To address this, Ma et al.^156^ developed a dual-camera surgical microscope comprising a spatial–spectral hyperspectral camera and a high-definition color camera, as shown in Fig. 3(b). By configuring the hyperspectral camera to acquire low-resolution images and then reconstructing high-resolution hyperspectral images using RGB guidance, they successfully reduced HSI scanning time, making fast and high-resolution HSI feasible for surgical interventions.

### 2.4 Hyperspectral Endoscope

**Fig. 4:**
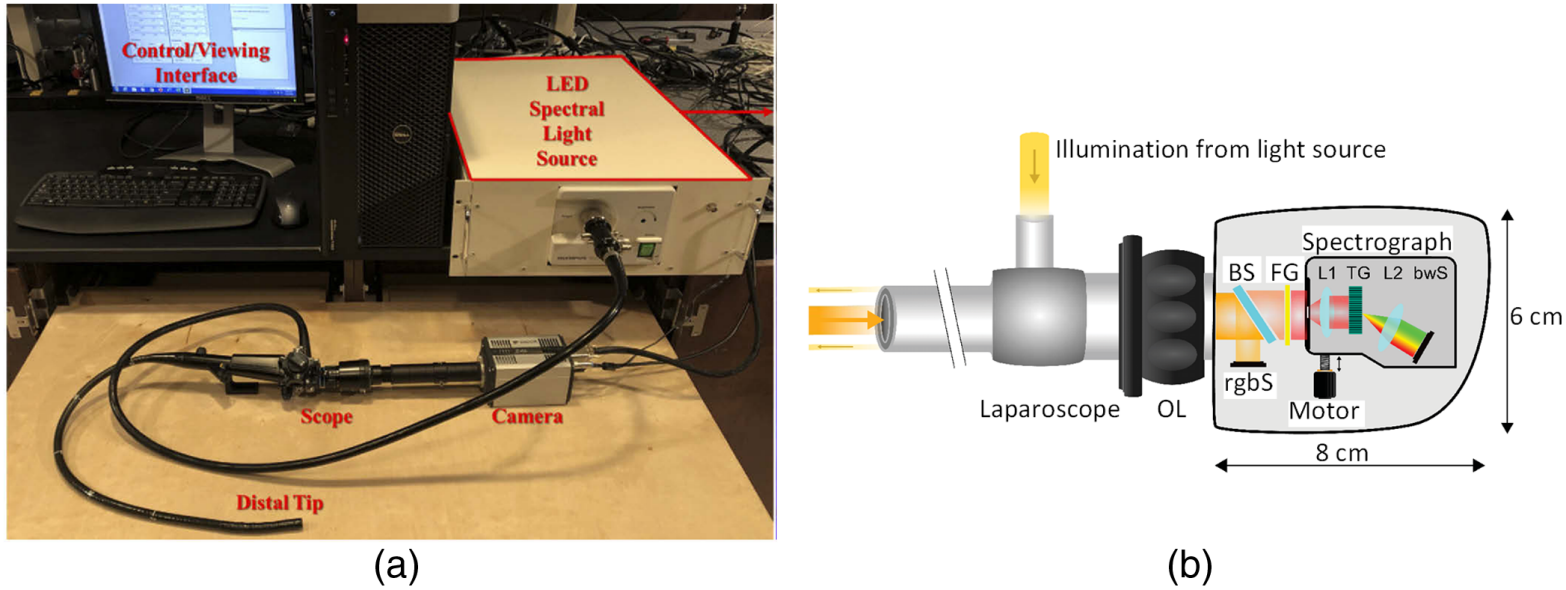
Examples of hyperspectral imaging endoscopes. (a) Hyperspectral endoscopic imaging system using LED illumination. Figure reproduced with permission from Browning et al.^191^ (b) Handheld push-broom hyperspectral endoscopic imaging system. Figure reproduced with permission from Kohler et al.^16^

Endoscopic imaging has been a critical tool in minimally invasive surgery, providing surgeons with the visualization required during procedures. Endoscopes, which are central to this technology, relay light to a target area and transmit images to a detector. These devices can be rigid, as in laparoscopes, or flexible, as in colonoscopes. To illuminate the target, endoscopes require a light source and a mechanism for guiding light. Early research focused on modifying light sources to incorporate HSI, enabling spectral data collection alongside standard visualization for improved tissue differentiation. Fawzy et al.^190^ introduced a filter wheel to a xenon light source, creating a system capable of acquiring 18 spectral bands ranging from 400 to 760 nm. Similarly, Han et al.^34^ utilized a 27-band filter wheel for illumination in a flexible endoscope, achieving spectral imaging from 405 to 665 nm. Browning et al.^191^^–^^193^ developed a system using LEDs to provide narrow-band illumination for endoscopes, as shown in Fig. 4(a). Early studies often used push-broom systems, which may not be able to provide a live view. This limitation was addressed by employing beam splitters to divide the incoming image into two paths: one directed to the push-broom HSI system and the other to a regular CCD camera for live viewing.^9^^,^^16^^,^^22^^,^^194^ One notable system, developed by Köhler et al.,^16^^,^^195^ used small motors to drive the push-broom mechanism, resulting in a compact system with dimensions of 8 cm by 6 cm [Fig. 4(b)]. Beam splitters have also been used to create multimodal HSI endoscopic systems, combining HSI with other imaging modalities. Luthman et al.^107^ developed a bimodal system capable of capturing spectra of reflectance and fluorescence from tissues. Lin et al.^17^ combined a push-broom HSI system with a 3D camera to reconstruct tissue surface shapes and reflectance spectra.

When combining HSI with endoscopy, one must account for optical distortions caused by the endoscopic system and their impact on hyperspectral images. One common distortion is fisheye distortion, a type of optical aberration where straight lines appear curved, especially near the edges of the image. This occurs because endoscopic lenses are often wide-angle to maximize the FOV, resulting in a barrel-shaped distortion. Fisheye distortion can be corrected through calibration techniques that map the distorted image to a standard rectilinear grid using mathematical models, such as polynomial transformations or lens-specific correction algorithms. Another significant distortion is nonuniformity, caused by variations in the angle between the endoscope tip and the imaging surface. Lee et al.^196^ investigated potential spectral distortions that are dependent on the angle of the endoscope. They found that the wavelengths 450 to 550 nm are more susceptible to angle-dependent distortion, and an increased endoscope angle also increases the distortion. Flexible endoscopes further introduce challenges as the image is transmitted through a fiber bundle. This results in the image being “chopped” into discrete fibers, producing a honeycomb-like structure. Correcting these patterns requires decombing and demosaicing techniques to reconstruct the continuous image. Moreover, Regeling et al.^72^ investigated specialized filtering methods to remove honeycomb patterns and developed a technique that outperformed Gaussian and other basic low-pass filters.

Ebner et al.^197^ outlined key technical requirements for HSI systems in surgical settings, specifying a physical size of less than 10×10×12  cm, a weight of less than 1 kg, and a spectral capability of at least 16 bands. Modern HSI cameras, particularly snapshot SRDA systems, now exceed many of these requirements. Advances in micro-electronics have enabled the development of hyperspectral endoscopes that are compact and lightweight, improving usability for physicians. Modir et al.^91^^,^^198^^,^^199^ designed a hyperspectral endoscopic imaging system using micro-LED technology. Unlike traditional setups, this system places all components at the tip of the catheter, minimizing artifacts caused by optical relays. Pruitt et al.,^200^ Ma et al.,^155^ and Tran et al.^109^ incorporated SRDA snapshot cameras into endoscopes. To improve the spatial resolution of these cameras, they fused hyperspectral images with high-resolution RGB images using super-resolution techniques.

### 2.5 Hyperspectral Histology Microscope

The recent 10 years witnessed the growth of research interest in hyperspectral microscopy. Most hyperspectral microscopes operate in the visible and lower end of the NIR (<1000  nm) range, with a few specifically targeting ultraviolet (UV)^7^ or short-wave infrared (SWIR, typically 900 to 1700 nm)^59^ ranges. Depending on the acquisition mode of hyperspectral data, these systems have different characteristics.

Push-broom hyperspectral microscope systems, though popular for their high spatial and spectral resolution as well as widespread availability, necessitate relative motion between the camera and the object, i.e., histologic slide.^13^^,^^24^^,^^201^ When employing push-broom HSI systems in microscopy, the relative motion is typically achieved using a synchronized motorized stage.^12^^,^^13^^,^^24^ However, movement artifacts during acquisition can lead to spatial aliasing issues, significantly diminishing image quality. One solution to use push-broom HSI with no external translation is to complete the scanning process internally by moving optics in front of the spectrograph^15^^,^^202^ or propelling the imaging sensor behind the diffraction grating.^8^^,^^93^^,^^94^^,^^96^^,^^203^ However, due to the inherent nature of push-broom systems, each frame only contains the spatial information of a single line in the object, making it somewhat challenging to achieve homogenous focus based on limited spatial components.^136^ In addition, the spatial resolution of push-broom HSI systems is determined by the width of the entrance slit. A narrower slit yields higher spatial resolution but also leads to a longer exposure time and imaging speed.^10^ Nevertheless, this can be potentially overcome with software-based spatial resolution enhancement.^10^^,^^135^

Similar to push-broom, spectral scanning ensures good spatial and spectral resolution.^2^^,^^4^^,^^125^ Among published studies, spectral-scanning microscope systems are frequently employed due to their excellent image and spectral resolution without external motion, making them relatively easy to implement. Like in many other HSI systems, tunable filters such as LCTF^59^^,^^149^^,^^204^^–^^207^ and AOTF^27^^,^^28^^,^^208^^–^^211^ remain the most popular spectral-scanning devices, with other options available such as Turret-mounted volume Bragg grating (VBG)^69^^,^^212^ and thin-film tunable filters (TFTF).^213^ They usually offer a wide wavelength range from visible^149^ to short-wave infrared,^59^ so users can choose the correct filter for their required specifications. When using a tunable filter, the imaging system, including the tunable filter and the monochromatic sensor, is mounted on the microscope optical port via a video adapter. A set of filters on a filter wheel,^66^ on the other hand, is sufficient and cost-effective when a smaller number of wavelength bands are needed, such as in fluorescence imaging. Comparatively, tunable filters offer faster and easier adaptation than filter wheels while scanning more spectral bands. Another way to implement spectral-scanning HSI microscopy is to use a tunable light source with a monochrome imaging sensor. For example, Lemaillet et al.^92^ replaced the microscope lamp with a tunable xenon light source that spans in steps of 10 nm over the 380 to 780 nm wavelength range. Stergar et al.^40^ employed a custom-made single-grating monochromator in the Czerny–Turner configuration after the halogen lamp to provide monochromatic illumination at selectable wavelengths. Lu et al.^214^ utilized a programmable optical filter comprising a grating and digital micromirror device, which was capable of providing either monochrome, coded, or white light illumination in the 400 to 676 nm spectral range. Furthermore, spectral-scanning HSI is more commonly used for fluorescence microscopy, such as in Liu et al.,^204^ Podlipec et al.,^215^ and Li et al.^55^ Favreau et al.^216^ developed a TFTF-based excitation-scanning microscope, which had higher sensitivity for autofluorescence imaging compared with the traditional emission-scanning approach. Similarly, Deal et al.^66^ used a filter wheel in excitation-scanning microscopy for the analysis of colon cancer autofluorescence. Roxbury et al.^69^ employed a VBG-based hyperspectral microscope to image infrared fluorescence signals of nanoparticles. Lin et al.^52^ developed a LCTF-based dual-type hyperspectral microscope with a halogen lamp for transmission spectra acquisition and a xenon lamp with a narrow bandpass filter centered at 361 nm for fluorescence excitation.

Snapshot hyperspectral cameras, known for their compact size and quick acquisition time, can be easily adapted to microscopes through a video adapter. In addition to utilizing commercial snapshot cameras, Yu et al.^165^ developed a snapshot hyperspectral microscopy system based on a microlens array. The system yields a 28  pixel×14  pixel×180 band hypercube with a significantly high spectral resolution of ∼0.56  nm, accompanied by a spectral reconstruction algorithm for the system.^217^ Hedde et al.^218^ created an ultrafast phasor-based hyperspectral snapshot microscopy using sine/cosine interference filters for biomedical imaging that are not feasible with conventional methods. Nevertheless, despite the advantages of video-rate acquisition speed in snapshot cameras for certain applications such as live cell imaging or time-lapse imaging, compromised spectral and spatial resolution may limit their use in other applications where high resolution is required, such as automatic cancer detection in histologic slides.

Other than snapshot cameras, another approach to rapid acquisition is compressive HSI, which captures hyperspectral data with fewer measurements than traditional scanning-based HSI methods. Oiknine et al.^120^ developed a compressive hyperspectral microscope, which reconstructed hypercubes with 250 spectral bands from 47 spectrally encoded images in the 450 to 700 nm range. Klein et al.^167^ constructed a broadband spatially encoded compressive hyperspectral microscope, which was capable of imaging in visible and NIR wavelength ranges. Meng et al.^112^ built an endomicroscopy system based on spectral snapshot compressive imaging. With the help of a deep neural network (DNN), they could capture and reconstruct hyperspectral data with up to 24 bands in near-real time. Liu et al.^219^ combined structured illumination and HSI in microscopy for cell analysis. They could reconstruct a data cube containing 31 spectral bands in 500 to 650 nm from only nine exposures, with an imaging time of 2.7 s per data cube.

In addition to brightfield microscopy, HSI has been investigated with other modalities such as fluorescence microscopy, light-sheet microscopy, darkfield microscopy, and confocal microscopy. A research group developed an excitation-scanning HSI microscope, which incorporated TFTFs to provide excitation at different wavelengths and acquired the fluorescence emission signals using a camera after a longpass filter.^67^^,^^220^^,^^221^ Lavagnino et al.^117^ developed a five-dimensional (5D) snapshot hyperspectral light-sheet imaging microscope based on an IMS HSI camera. Ray et al.^222^ attached a tunable filter and a monochrome CCD camera to a darkfield microscope to measure nanoparticle scattering in the 450 to 650 nm range. Zhang et al.^58^ placed an LCTF in front of the darkfield condenser to tune the excitation wavelength. Zucker et al.^223^ attached a push-broom HSI system to a microscope, which was capable of darkfield and fluorescence imaging. A commercially available system integrated with push-broom visible and near-infrared (VIS-NIR) HSI and a darkfield condenser has been employed for various applications in recent 10 years.^224^^–^^235^ Furthermore, Bertani et al.^236^ imaged HaCaT and melanoma cells with a hyperspectral confocal reflectance microscope, which covered a 500 to 1000 nm range with 1.6 nm spectral resolution. To measure cyclic adenosine monophosphate (cAMP) signals, Rich et al.^237^ and Howard et al.^238^ used a hyperspectral confocal microscope that acquired emission signals from 414 to 724 nm. Nyström et al.^239^ used a hyperspectral camera to acquire the emission spectrum of amyloid tissue between 503 and 687 nm. In 2023, Sung et al.^240^ developed a hyperspectral confocal microscopy system in the SWIR range of 1100 to 1600 nm.

Fourier transform infrared (FTIR) microspectroscopy has been widely used for chemical analysis applications. In the past decade, with IR spectrums revealing the absorption bands associated with different biochemical components such as proteins and DNA, FTIR has drawn attention as an alternative diagnostic tool.^241^ The system collects an interferogram and converts it to a spectrum using the Fourier transform.^242^ Even though FTIR spectroscopy is different from HSI in a traditional sense (≥10 discrete bands with dispersive optics or filters), FTIR microscopy with a focal plane array detector, especially for biomedical imaging applications, arise similar to a form of HSI targeting mainly at the mid-infrared (MIR) range (∼4000 to $600\;{\mathrm{cm}}^{-1}$).^241^^,^^243^^–^^246^

As imaging applications have become more demanding for higher throughput, new technologies have begun to replace FTIR. The use of tunable MIR quantum cascade laser (QCL) systems has recently proved more effective than FTIR, with better signal-to-noise ratios and time-to-results with no cooling required for the detector.^245^ A QCL-IR microscope, close to the mechanism of spectral-scanning HSI, employs a tunable QCL or string of QCLs working in concert to cover wider spectral ranges for illumination. Then, a wide-field imaging IR objective lens forms image on an infrared sensitive focal plane array imager. At any given instant, only a narrow wavelength band emits from the QCL. The wide-field imaging offers a very large FOV compared with an FTIR and enables live, single-frequency, and rapid hyperspectral imaging of samples.

#### 2.5.1 Whole-slide scanning hyperspectral microscope

**Fig. 5:**
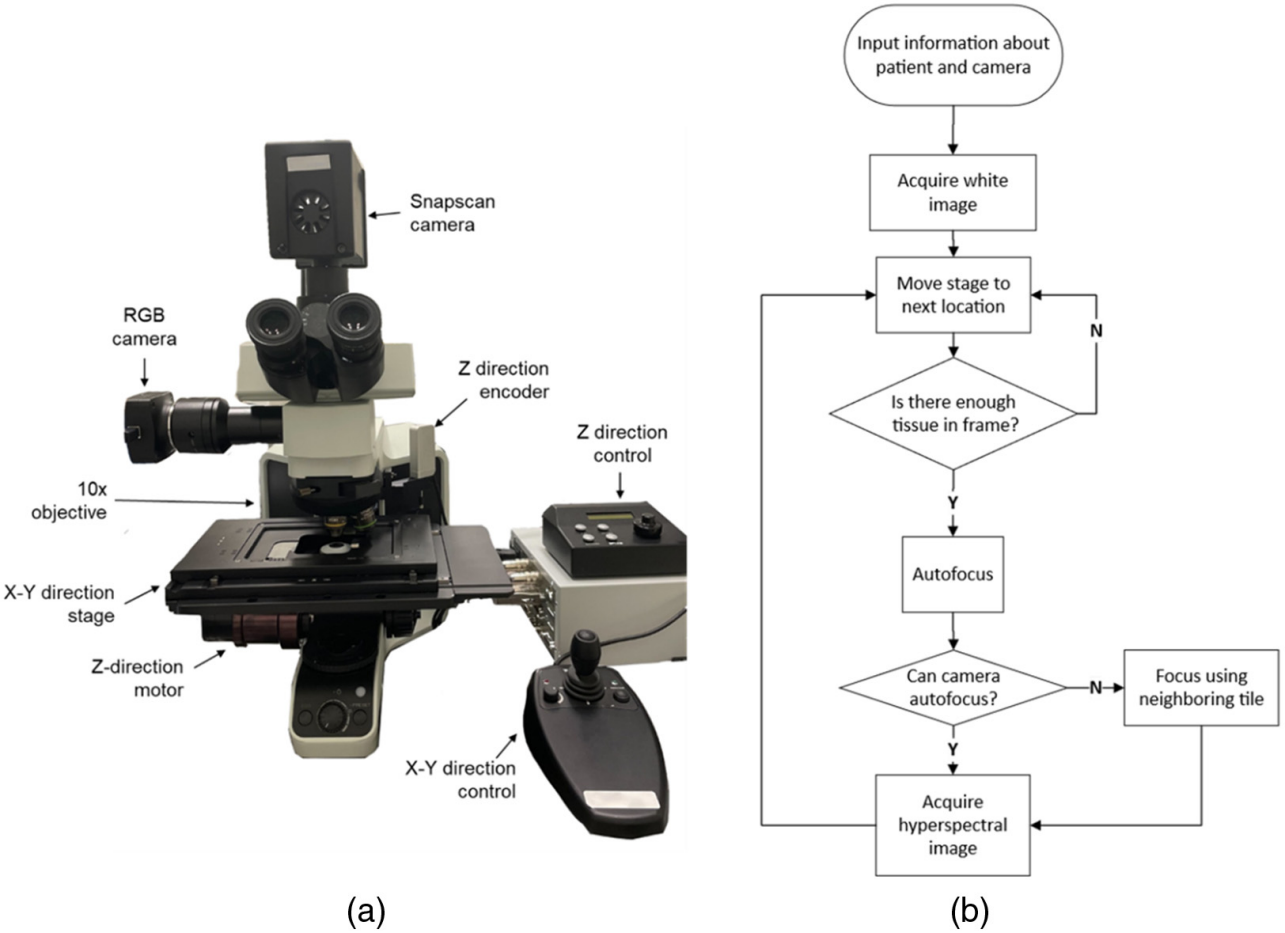
Whole-slide hyperspectral microscope system by Tran et al.^93^ (a) Customized, automatic hyperspectral imaging microscopic system. (b) The flowchart for the whole-slide hyperspectral image acquisition.

Whole-slide imaging (WSI) involves scanning and digitizing the entire slide at high magnification for subsequent analysis. This process utilizes specialized scanners and software for viewing, analyzing, and annotating the resulting digital images. Despite the advancements in hyperspectral histologic imaging, there are challenges in training networks due to the scarcity of whole-slide hyperspectral data. Several studies have focused on developing and utilizing whole-slide hyperspectral microscopes. Enfield et al.^68^ created an in-house automated whole-slide hyperspectral microscope using spectral-scanning imaging, studying spatial tumor-immune cell interactions in lung cancer recurrence. The system incorporated an upright microscope, a motorized X−Y translational stage, an LCTF, and a CMOS camera. Operating with a 20× objective lens, the microscope achieved a spatial resolution of 0.33  μm/pixel and collected up to 16 spectral bands per hypercube within the 420 to 720 nm wavelength range. The system seamlessly stitched overlapping camera fields to create a cohesive hyperspectral image. Ishikawa et al.^8^ constructed a whole-slide HSI acquisition system for cancer detection in H&E-stained human pancreatic tumor tissue microarray slides. This system, featuring an upright light microscope, a hyperspectral camera, and a motorized X−Y stage, employed a push-broom hyperspectral camera with an internal shifting system. With a 20× objective magnification, the system achieved a spatial resolution of 0.25  μm/pixel, acquiring a single hypercube in 3 to 7 s with 480×752  pixels. During whole-slide scanning, the X−Y stage moved in a serpentine fashion, and Z focus with 2  μm increments was actively performed. Tran et al.^93^ utilized a spatial–spectral hyperspectral camera to develop a customized automatic hyperspectral imaging microscopic system for whole-slide scanning of head and neck cancer histology slides. The camera captured the entire hypercube without any movements. The system, featuring a commercial X−Y motorized stage and a high-precision encoded stepper motor focus drive, as shown in Fig. 5, not only carried out autofocus but also reconstructed the whole-slide hyperspectral image using a phase correlation-based method. Interestingly, they relied on the frames acquired by the spatial–spectral camera sensor and applied a 3×3 filter kernel based on the Brenner focus measure for autofocus. Due to the integrated FPI filters covering all spectral bands, this approach eliminated the need to focus on various bands individually. Liao et al.^64^ developed a terapixel hyperspectral WSI system based on slit-array detection and projection. This system, reliant on specific optical equipment, scanned the sample in a push-broom fashion along one spatial dimension, producing rapid hyperspectral data. They also addressed the auto-focusing by illuminating the sample with two LEDs from oblique angles and recovering focus points based on 1D autocorrelational analysis.^247^ Focus map was acquired row-by-row in the sample, taking ∼20  s per row.^64^

### 2.6 Polarized Hyperspectral Imaging

Polarizers are commonly used with HSI to reduce specular reflection.^20^^,^^53^^,^^54^^,^^248^^,^^249^ In some studies, polarizers were used to improve the optical throughput of hyperspectral imaging systems, as some components, such as AOTF and LCTF, might be optimized for lights with certain polarization.^30^^,^^141^ However, except for these basic functions of polarizers, polarized light imaging (PLI) emerges as a potent optical technique for exploring the structure and morphology of biological tissues by capturing their polarization characteristics.^250^ The organic combination of PLI and HSI leads to PHSI, which represents an amalgamation of polarization measurement, spectral analysis, and spatial imaging technology. This modality concurrently captures three types of information, providing richer image data for both tissue imaging and histopathology.

The idea of combining spectral imaging and polarimetry can trace back to the last century.^251^ Later, it was found that PLI helps with multispectral imaging in dermatology,^252^ as it restrains the penetration depth so that the imager only obtains single-scattered light from the superficial layer of skin.^253^ In recent years, attempts have been made to upgrade the system with more spectral bands. Vasefi et al.^78^ developed a polarization-sensitive hyperspectral imaging system for dermoscopy. Two hyperspectral cameras were spatially registered and shared the same FOV via a beam splitter. Each camera had a polarizer that was oriented orthogonally to one another. With a linearly polarized light source, the dual-camera setup acquired hypercubes of skin in both parallel and cross polarization. The hypercubes contained 33 spectral bands in the wavelength range of 468 to 857 nm. Both the cross-polarized optical density spectrum and polarized attenuation spectrum show distinctive changes along with the melanin concentration and hemoglobin oxygenation in a skin lesion, especially in the 500 to 600 nm range. A research group, including Zherebtsov and Dremin et al.,^105^^,^^154^ customized a handheld PHSI system by adding two cross-polarized linear polarizers in front of the ring-shaped light source and the snapshot hyperspectral camera. The system acquired two 79-band hypercubes in the 510 to 900 nm range, with the polarizers being parallel and perpendicular oriented, respectively. Then, the degree of linear polarization (DOLP) was calculated using the two hypercubes, and the polarization index was generated based on DOLP across all wavelength bands. Though requiring manual rotating of the polarizer during image acquisition, the developed system showed potential in assessing skin complications of diabetes mellitus. Huang et al.^254^ developed a high-efficiency hyperspectral-polarization imaging system using a polarization camera array with notch filters. The device acquired a stack of three notch-filtered polarized images and one panchromatic polarized image. Then, by employing a compressive sensing–based spectral super-resolution method, the four-channel data got boosted to a hyperspectral-polarization data cube with 16 spectral bands. In addition to the systems that mainly exploit the linear polarization of tissues, PHSI setups that are capable of full Stokes imaging were developed. Ghassemi et al.^255^ constructed an imaging device consisting of a polarized hyperspectral imaging apparatus combining Stokes polarimetry and spatial frequency domain imaging. The PHSI part had a linearly polarized white LED and a spectro-polarimeter, which comprised two liquid crystal variable retarders (LCVRs), one LCTF, one linear polarizer, and a monochrome digital CCD. The LCTF had a wavelength range of 450 to 700 nm with an FWHM of 7 nm. Later, Ma et al.^256^ replaced the wavelength-scanning HSI with a compact snapshot hyperspectral camera, which was able to conduct real-time imaging. By evaluating the spectral signatures of the degree of polarization (DOP), DOLP, and degree of circular polarization (DOCP) on various *ex vivo* tissues, they proved that both linear polarization and circular polarization are useful for tissue assessment.

**Fig. 6:**
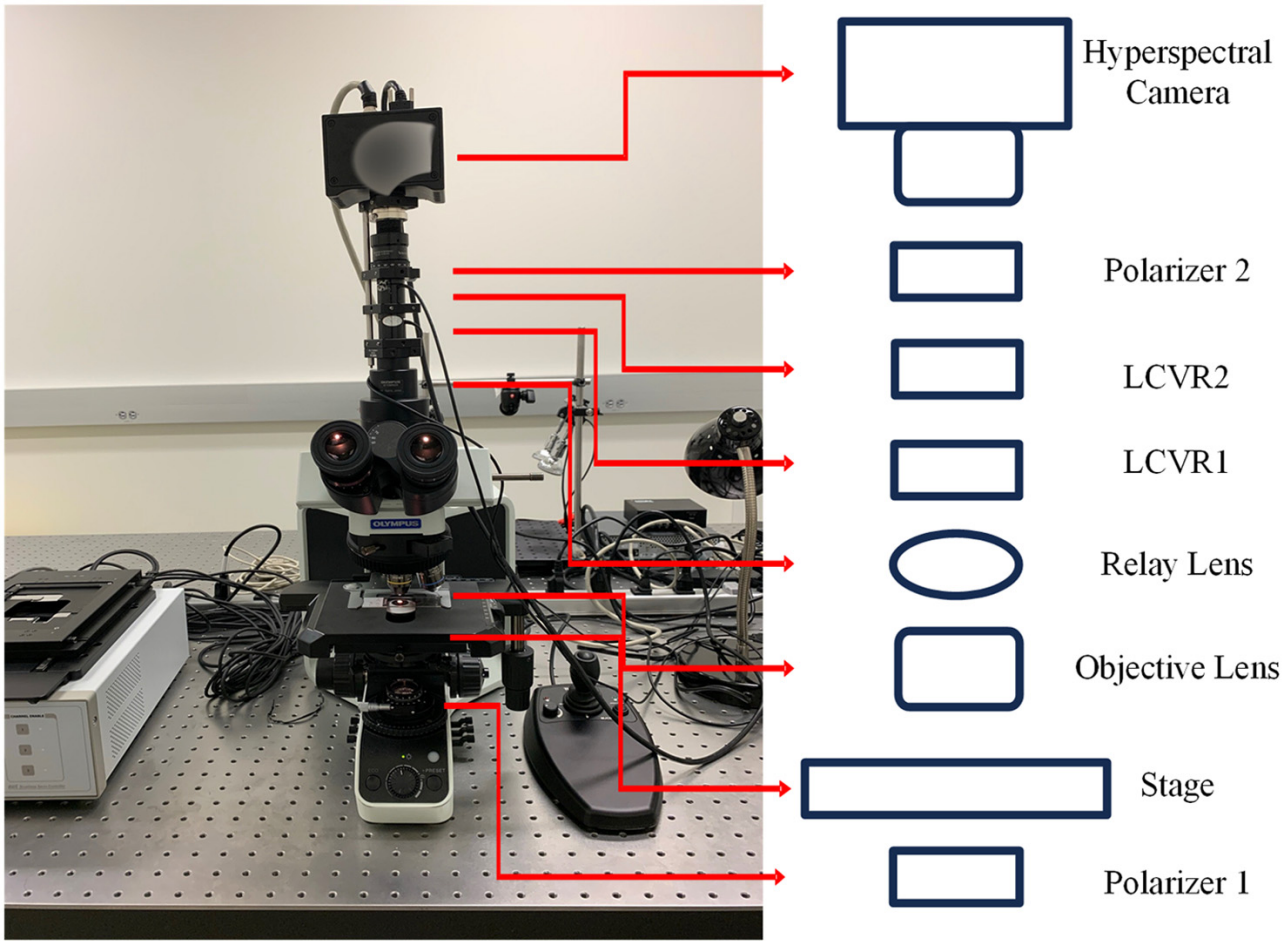
Polarized hyperspectral microscopic imaging system. The transmissive axis orientation of polarizer 1 is at 45 deg, and that of polarizer 2 is at 0 deg. The fast axis orientation of LCVR1 is at 0 deg, and that of LCVR2 at 45 deg. Figure reproduced with permission from Zhou et al.^98^

Except for the abovementioned PHSI systems for *in vivo* and *ex vivo* tissue imaging, Zhou et al.^98^^,^^157^^,^^158^^,^^257^^–^^260^ employed PHSI in histology. They integrated HSI and PLI into an optical microscope for enhanced visualization and automated cancer detection in histological slides, as shown in Fig. 6. The imaging system’s core components include an upright optical microscope with an integrated halogen light source, two linear polarizers, two LCVRs, and a customized spatial–spectral hyperspectral camera. The LCVRs and polarizers are dedicated to polarized light imaging. The spatial–spectral hyperspectral camera has a wavelength range of 470 to 750 nm and 97 spectral bands. The PHSI system, as shown in Fig. 6, sets the LCVRs to different retardance values to acquire four element images—horizontally polarized light image, vertically polarized light image, 45-deg polarized light image, and right circularly polarized light image—and subsequently calculates the full Stokes vector (S0, S1, S2, and S3). These spectral signatures aid in differentiating various cellular components. The customized PHSI microscope seamlessly integrates into the standard pathological analysis process, complementing existing optical microscopes.

### 2.7 Handheld Hyperspectral Imaging Systems

Leveraging compact hyperspectral cameras and 3D printing, handheld HSI devices have been developed for a range of applications.^38^^,^^39^^,^^100^^,^^119^^,^^261^^–^^266^ The combination of hyperspectral cameras and smartphones offers a powerful tool, with the smartphone serving as a platform for data transmission, image acquisition, processing, and visualization.^266^ For example, a commercial system was used by Kleiss et al.^264^ and van Schilt et al.^265^ for monitoring tissue oxygenation in healthy volunteers’ feet. Hosking et al.^74^ developed an advanced system, which used a high-sensitivity CCD camera and could capture 21 spectral bands in a wide wavelength range of 350 to 950 nm. Although the smartphone was not used for processing or main image acquisition, it created a live display for better acquisition quality. He et al.^267^ used the images acquired by a smartphone to produce hyperspectral images through a process called Weiner estimation. The process is a matrix reconstruction, where values were determined by calibrating the smartphone images against hyperspectral images of the same scene. After the parameters for the Weiner estimation were determined, the authors produced 16-band images from three-channel RGB smartphone images. The method was employed to estimate hemodynamic activities, e.g., heart rate and oxygenation rate, and melanin absorption.

### 2.8 Hyperspectral Augmented Reality

Augmented reality combines computer-generated virtual models and real-world scenes to enhance visualization and sensory experience. A typical AR system requires image acquisition, tracking, data processing, and display components.^268^ Three main types of AR display include: (1) head-mounted displays, which directly project the augmented simulation on a wearable device such as smart glasses or headsets, (2) head-up displays, which project the simulation onto a screen that the user can look at, and (3) direct projection, which projects the data directly onto the patient.^269^ AR and HSI can be combined to further improve visualizations. Barberio et al.^270^ developed a HYPerspectral Enhanced Reality (HYPER) system that can estimate perfusion of tissue and superimpose the heatmap with a real-time video during surgery. A commercial push-broom camera and a high-definition video camera were used to provide image inputs. The pseudo-color ${\mathrm{StO}}_{2}$ map and real-time video stream were registered for display on a monitor in real time. HYPER and similar systems have been used in animal studies during esophageal surgery,^271^ liver resection,^272^ and pancreatic surgery.^273^ Huang et al.^274^ used hyperspectral data to train a classification algorithm that can identify blood vessels, normal brain tissues, and tumor brain tissues. The segmentation result was then projected onto a head-mounted display using a tracking and registration mechanism. Sancho et al.^162^ proposed an AR system integrating HSI and light detection and ranging (LiDAR). Hyperspectral data were used to differentiate normal and tumor brain tissues, and the classification results were overlapped with the point cloud acquired by the LiDAR camera.

### 2.9 Validation and Characterization of Hyperspectral Imaging Systems

Multiple approaches can be used to validate HSI systems, including *in vitro* tissue cultures, *in silico* computer simulations, *ex vivo* animal or human tissues, *in vivo* animal models, and human subjects. Here, we focus on experimental designs commonly employed to characterize the performance and quality of HSI systems.

#### 2.9.1 Tissue preservation

To image biological targets, either *in vivo* or *ex vivo*, imaging time and settings are important. Tissues are prone to drying and degrading, especially under high-intensity light and exposed conditions. To preserve tissues, freezing or preservation fluids can be used. Tissue preservation can be performed rapidly using liquid nitrogen^83^^,^^210^^,^^275^^–^^277^ or slowly by freeze-drying.^222^^,^^275^^,^^278^ Typically, samples stored in freezers at temperatures from −60°C to −80°C^84^^,^^275^^,^^277^^,^^279^ can last up to many weeks or months. To bring tissue samples back to room temperature for imaging, a liquid buffer can be used to avoid direct contact with the environment. Studies were performed to differentiate fresh and frozen-thawed biological tissues using Raman spectroscopy^280^ or HSI.^281^ For example, Barbin et al.^281^ found that samples of *longissimus dorsi* muscles in pigs have lower spectra in the 950 to 1100 nm and 1150 to 1350 nm range, the longer they stay frozen. One explanation for this is the loss of moisture in the tissue during the freezing and thawing process, which can affect the tissue’s spectral absorption. Another method of tissue preservation is through dehydration fluid. Because water is a highly absorbing molecule, hydrated samples cannot be well imaged under a microscope unless they have a thickness of 10  μm or less.^282^ As such, it is common to dehydrate tissues and embed them in paraffin for hyperspectral histology.^97^^,^^205^^,^^226^^,^^283^^–^^287^ A standard dehydration process can be applied to the tissue.^245^ After fresh tissue is dehydrated and stained, HSI can be used to identify specific biomarkers such as Ki67.^97^

#### 2.9.2 Tissue-mimicking phantoms

Tissue-mimicking phantoms can be designed and manufactured to approximate the biological and spectral features of various tissues, such as skin and vasculature/hemodynamic environment.

A good skin phantom should simulate not only the spectral response of skin but also the scattering response of different layers.^288^ Zherebtsov et al.^105^ simulated skin by embedding red blood cells within a polyvinyl chloride-based matrix and validated their phantom by measuring its spectral absorption and scattering responses. General tissue phantoms typically simulate the presence of blood in the tissue medium. The common recipe for tissue-mimicking phantoms includes: a mixture of deionized water, biological or chemical chromophores, and a buffer solution. The buffer can be gelatinous (agar, gelatine, gel wax, silicone, polymer gels) or liquid (milk, Intralipid).^63^^,^^140^^,^^179^^,^^279^^,^^289^ The concentration of Intralipid can range anywhere from 0.5% to 10%.^44^^,^^290^^,^^291^ Fu et al.^44^ mixed a solution of Intralipid and blood at different concentrations. They found that higher concentrations of hemoglobin produce higher reflectivity from 475 to 625 nm, whereas higher concentrations of Intralipid produce higher reflectivity at 475 to 700 nm. To simulate the progression of cancer, fluorescent materials can be added to the phantoms.^57^^,^^62^ Certain dyes can be used to introduce specific spectral responses. For example, Luthman et al.^291^ added nigrosine and introduced an absorption peak at 633 nm to coincide with their LED wavelengths.

**Fig. 7:**
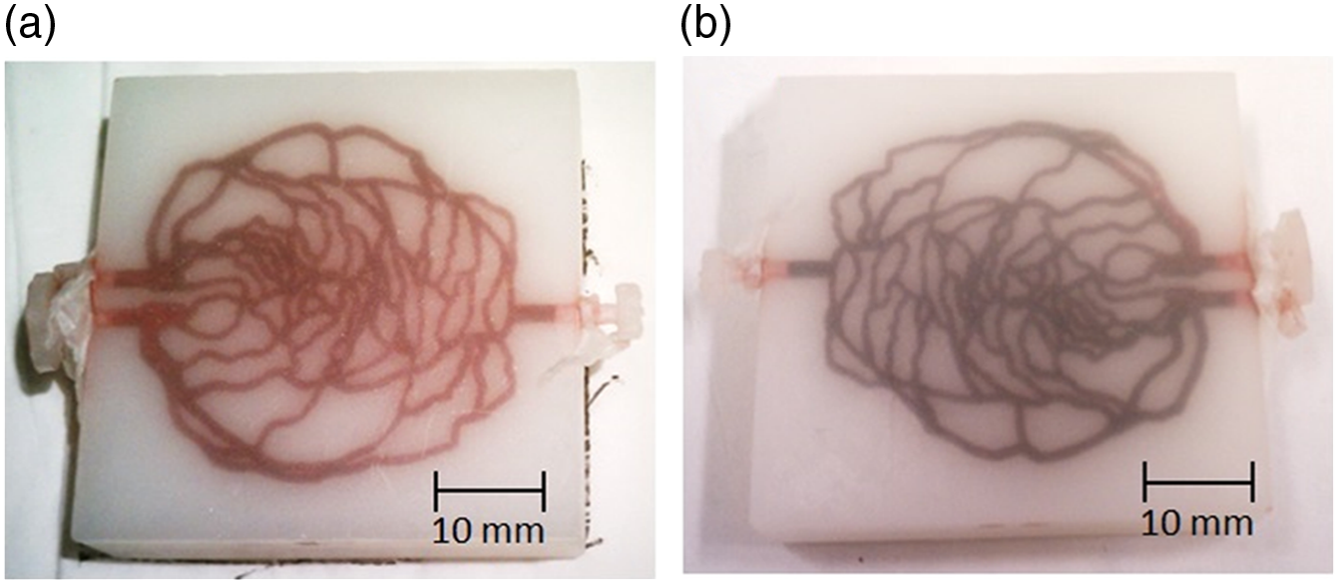
Example of a biological phantom used to validate the hyperspectral imaging system. The phantom is printed in 3D resin, based on a scan of a human retinal image. The phantom was injected with a solution of fully oxygenated hemoglobin (${\mathrm{HbO}}_{2}$) and 2% yeast so that the hemoglobin deoxygenates over time. (a) Phantom 30 min after injection. (b) Phantom 120 min after injection, showing deoxyhemoglobin (HHb). Figure reproduced with permission from Ghassemi et al.^294^

Preparation of blood phantom is similar to that of tissue-mimicking phantoms: a mixture of blood, deionized water, and plasma-mimicking buffer.^43^ The oxygenation of blood can be adjusted mechanically through exposure to pure oxygen or to inert gas such as nitrogen.^103^ It can also be achieved chemically.^43^^,^^107^^,^^292^^,^^293^ Sodium hydrosulfite, also known as ${\mathrm{Na}}_{2}S_{2}O_{4}$, reduces the oxygenation rate. Conversely, hydrogen peroxide ($H_{2}O_{2}$) increases oxygenation rate.^107^^,^^293^ In addition, yeast can be used to deplete oxygen.^294^ To properly validate the oxygenation of blood, a separate oxygen monitoring tool should be used.^291^ To mimic vasculature, small tubes of blood can be embedded in a tissue-simulating medium such as agar.^9^ For example, Huang et al.^274^ constructed a phantom of a brain tumor using agar, bovine gelatin, and yellow pigment ink, which was used to validate their AR system. In some cases, pumps or syringes are needed to simulate the hemodynamic properties of blood. To improve anatomical accuracy, 3D printing techniques can be used. Ghassemi et al.^294^ built a biological phantom of the retina equipped with blood vessels (Fig. 7), where a real fundus image was used to 3D print a model of the retinal vessels. Though the model had some deviation from the original image, a diameter of less than 1 mm was achieved for the channels.

#### 2.9.3 Digitally simulated phantoms

To better understand the interaction between light and tissue, digital simulations or *in silico* phantoms can be used to model different types of biological tissues and light sources. Monte Carlo (MC) transport simulation remains the primary method to model light propagation in biological tissue for HSI analysis.^6^^,^^65^^,^^74^^,^^79^^,^^92^^,^^105^^,^^115^^,^^123^^,^^191^^,^^193^^,^^257^^,^^295^^–^^305^ MC used to be computationally intensive,^306^ but modern implementations of MC use graphics processing unit (GPU) acceleration to perform parallel processing and improve the speed.^307^^,^^308^ Along with MC simulations in the forward direction, there’s also inverse MC (IMC), which fits measured HSI reflectance to tissue parameters. While also being computationally expensive, it is the gold standard for extracting optical properties.^306^

We first discuss the construction of biological models used in MC simulations. Many MC simulations assume tissue as one or several homogenous layers, each with specific optical properties (absorption, scattering, anisotropy). This is a reasonable approximation for layered tissues such as skin (epidermis, dermis, subcutaneous layers).^105^ For example, Zherebtsov et al.^105^ modeled a seven-layer skin model with different thicknesses, blood volume fractions, water contents, and melanin contents. The resulting data were used to train an artificial neural network that makes inferences on hyperspectral images. Similarly, Milanic et al.^309^ built a three-layer skin model and then embedded small lipid-rich inclusions in it to simulate subcutaneous xanthelasma lesions. They used the simulation to train machine learning algorithms to identify hypercholesterolemia (high cholesterol in blood), then validated their method on real data from 11 volunteers. They found a significant correlation between the HSI extracted features and the measured cholesterol of the subjects.

With improved processing speed, researchers have moved beyond simple and flat models toward more realistic 3D representations of tissues. For example, Giannoni et al.^308^ constructed a voxelized 3D model of a human brain biopsy (∼5  mm thick, containing gray matter tissues) to simulate how deep each wavelength penetrates into the sample. Giannoni et al.,^302^ in an earlier paper, used mesh-based MC to simulate the geometry and surface vasculatures of a mouse brain surface. With the simulation, they found that only eight optimally selected wavelengths between 780 and 900 nm are sufficient to identify the concentration of HHb and cytochrome-c-oxidase (CCO). When generating MC simulations, researchers can perform parameter sweeps, i.e., running the simulation multiple times with different physical values of oxygen saturation, vessel sizes, scattering coefficients, etc.^124^ This creates a library of synthetic spectra that can be used to train machine learning algorithms or test the robustness of HSI analysis methods. In addition, we noted one use of MC not in simulating biological systems, but in designing and optimizing an endoscopic hyperspectral light pipe that can maximize light delivery.^193^

## 3 Data Processing and Algorithm Development

The processing and analysis for biomedical HSI include preprocessing, classification, segmentation, and image enhancement. The workflow of an HSI study starts with a proper setup of cameras, optical, and illumination systems. After data acquisition, the images need to be preprocessed to remove noise, glare, and other artifacts. HSI data may also need dimension reduction for ease of processing. Finally, relevant features are extracted, and the images are analyzed. Machine learning and deep learning algorithms can be applied to extract higher-level features for further analysis. In the era of big data, deep learning models can leverage large volumes of input data and computational resources to identify complex patterns, enabling decision-making that rivals or even surpasses human visual perception.

Even with powerful computation in modern times, the large amount of data in HSI still poses some processing problems. Computation time should be considered when processing HSI data. As such, we make references to the time complexity of different algorithms, more specifically to the big O notation used in computer science. Big O notation describes how computation time and memory scale with data dimensionality and dataset size. For example, if there are n hyperspectral images, then an O(n)-time algorithm scales linearly with the amount of data, an O(log n)-time algorithm scales logarithmically with the amount of data, an $O(n^{2})$-time algorithm scales by the square order of the data, and an $O(2^{n})$-time algorithm scales exponentially with the data. The order of their time complexity on a large amount of data is $O(\log\;n)<O(n)<O(n^{2})<O(2^{n})$.

### 3.1 Hyperspectral Image Preprocessing

Preprocessing is critical to reduce variations in optics and illuminations during data acquisition. There are four major tasks for HSI preprocessing: reference calibration, artifact removal, normalization, and dimension reduction. In this section, we introduce the following notation: Let $X=[x_{1},x_{2}\ldots x_{C}{]}^{T}\in R^{n}$ be the vector that represents the spectrum of a single pixel in the hypercube with C channels. A hyperspectral image could be considered a multidimensional array that consists of many such X vectors. Let $X^{\prime }$ be the processed spectrum as a result of processing X.

#### 3.1.1 Reference calibration

The spectrum of light received by the camera, whether through transmittance or reflectance, is dependent on the incoming light source, the spectral response of the camera, the intrinsic dark noise of the imaging sensor, as well as the optical properties of the tissue. To accurately retrieve tissue optical properties (e.g., reflectance, transmittance), it is essential to perform both white reference and dark current calibrations on the image.^310^ A dark current reference image is taken with no incoming light to the sensor; this can be accomplished with a lens cap or in a dark room. Its purpose is to estimate and eliminate the dark noise of the system. White reference calibration is performed using an image captured under the same lighting conditions but with a spectrally uniform, highly reflective target. If using a reflective configuration, the target is a reflectance board with a diffuse reflective surface, such as Spectralon^®^ Diffuse Reflectance Standards (Labsphere, North Sutton, New Hampshire, United States). For transmission measurements, such as transmission light microscopy, the target can be a blank glass slide. The calibrated spectra are derived using the raw spectra, the white reference spectra $X_{\text{white}}$, and the dark reference spectra $X_{\text{dark}}$:

$$X^{\prime }=\frac{X-X_{\text{dark}}}{X_{\text{white}}-X_{\text{dark}}+\epsilon }.$$

This step produces spatially calibrated images that correct the spectral differences in light sources and cameras. To avoid dividing-by-zero errors, a small ε is added to the denominator. In addition, sample topography can affect the spectra. Rogelj et al.^19^ applied curvature correction to improve the spectral accuracy; however, their method required 3D profilometry imaging.

#### 3.1.2 Denoise

It is advised that noise processing should be done at the early stages of pre-processing as noise can influence the outcome of hyperspectral analysis. Many types of noise are present in hyperspectral images, such as striping (from incorrect push-broom operations), salt and pepper noise, and shot noise (Poisson noise). Here, we identified three methods of noise reduction or removal: smoothing filters, matrix factorization methods, and neural networks.

To denoise in the spatial dimension, smoothing filters such as median filters, averaging filters, Gaussian filters, or Savitzky–Golay filters can be used.^138^ Multiple images can be taken successively and then averaged together to reduce noise.^200^ Matrix factorization techniques decompose the hyperspectral image into orthogonal components and then remove or reduce the least relevant components that contribute to noise. One such popular factorization method is the minimum noise fraction (MNF) method.^6^^,^^71^^,^^124^^,^^145^^,^^216^^,^^296^^,^^311^^–^^317^ It consists of three stages. First, the image is decorrelated and reordered based on increasing signal-to-noise ratio. This stage requires prior knowledge of the noise and data covariance, so a better understanding of the noise profile improves the performance of MNF. Second, the noisiest components are either removed or smoothed. Third, the resulting image is reconstructed. Singular value decomposition can also be used as an alternative denoising method.^318^^–^^320^ A nonlocal meets global algorithm uses singular value decomposition prior to spatial denoising to denoise hyperspectral denoising.^321^ The method was used by Aloupogianni et al.^319^ to denoise images before tumor segmentation. Neural networks can estimate noise parameters and therefore be used to reduce noise in hyperspectral images. They are trained by adding noisy images to normal, nonnoisy images.^322^ Although there is active research on neural network-based noise reduction in remote sensing, this review focuses on medical HSI and therefore does not include those papers.

#### 3.1.3 Spectral normalization

After white and dark references calibration, normalization can be applied to the spectra.^323^ Normalization has the following advantages: the spectra are rescaled to fit statistical learning methods, differences in light intensity between subjects are reduced, the shape of the spectra becomes a more important feature compared to the amplitude, and the inter-class similarity and intra-class differences increase.^160^^,^^164^^,^^324^^,^^325^ One method of spectral correction is called standard normal variate (SNV).^326^ SNV is applied on every spectral vector X using the mean $\mu_{x}$ and the standard deviation $\sigma_{x}$, as shown in the following equation:

$$X^{\prime }=\frac{X-\mu_{X}}{\sigma_{X}}.$$

SNV works well with statistical tests and simple statistical machine learning algorithms.^327^^,^^328^ However, Barberio et al.^329^ found that applying SNV could harm the performance of a deep learning network, potentially due to the fact that SNV reduces the variation needed for deep learning algorithms to produce meaningful inferences. Alternative normalization methods include min-max normalization,^160^^,^^330^ negative logarithmic transformation,^304^ and L2 norm normalization.^164^^,^^330^ Min-max normalization scales the spectra into a range from 0 to 1 using the following equation:

$$X^{\prime }=\frac{X-\min(X)}{\max(X)-\min(X)}.$$

Negative logarithmic transformation aims to center the spectra around 0 with the following equation:

$$X^{\prime }=-\log(\frac{X}{\sum_{i=1}^{C}x_{i}}).$$

Vector normalization (L2 norm normalization) scales the length (L2 norm) of each vector X to one by dividing the vector by its L2 norm using the following equation:

$$X^{\prime }=\frac{X}{\|X{\|}_{2}}=\frac{X}{\sqrt{\sum_{i=1}^{C}x_{i}^{2}}}.$$

#### 3.1.4 Demosaicing

With the emergence of snapshot HSI using SRDA, it is essential to develop demosaicing methods to reconstruct full spectral images from filter array outputs. The method was inspired by techniques used in color photographs and color filter arrays. However, extending from three channels in conventional RGB to 16 channels or more in HSI presents significant challenges. The most straightforward method to demosaic an image is by linear interpolation.^107^ Luthman et al.^107^ generated the values of the demosaiced pixels by interpolating adjacent micropixels in their 16 and 25 spectral bands configuration. Raita-Hakola et al.^38^ applied bilinear interpolation to demosaic images. Pruitt et al.^121^^,^^200^^,^^331^ investigated three different image demosaicing methods: white reference calibration, low-pass filtering, and filter convolution. A better spatial resolution was achieved by white reference calibration in the visible light range (460 to 600 nm) and by filter convolution in the red to near-infrared range (600 to 900 nm). Li et al.^332^^,^^333^ used deep learning to tackle the demosaicing problem. In one study,^333^ they generated simulated mosaiced images with full-resolution hyperspectral images to train a model in a supervised manner. In a later study,^332^ an unsupervised network was trained for the task.

### 3.2 Dimension Reduction

Hyperspectral data are often large with sizes up to orders of gigabytes per single image. Thus, it is common to reduce the size of the data prior to analysis, either through dimensional projection, features extraction, or feature selection. Dimensional projection aims to convert the hyperspectral data into a lower-dimensional representation with less features. The projection could be linear, as in the case of principal component analysis (PCA),^11^^,^^14^^,^^81^^,^^127^^,^^128^^,^^185^^,^^208^^,^^220^^,^^236^^,^^312^^,^^315^^,^^334^^–^^345^ nonnegative matrix factorization (NMF),^89^^,^^90^^,^^114^^,^^124^^,^^129^^,^^292^^,^^318^^,^^342^^,^^346^^–^^352^ and *t*-distributed stochastic neighbor embedding (t-SNE),^319^^,^^347^^,^^353^^–^^357^ or it could be nonlinear, as in the case of isomap^358^ and uniform manifold approximation.^359^

PCA is one of the most common methods in hyperspectral dimension reduction.^11^^,^^14^^,^^81^^,^^127^^,^^128^^,^^185^^,^^208^^,^^220^^,^^236^^,^^312^^,^^315^^,^^334^^–^^345^ The goal of PCA is to produce orthogonal projections such that the variance can be maximized on the first coordinate (so-called the first principal component). The second, third, and subsequent principal components are determined by selecting the coordinates with the second, third, and subsequent largest variances. To reduce the number of channels in a hyperspectral image with C channels and N pixels using PCA, each of the N pixels is considered a data vector with C dimensions. First, SNV is performed on the individual pixels to zero-center them. Then, the covariance matrix of the image $\mathrm{Cov}=\frac{1}{N}\overset{N}{\underset{i=1}{\sum }}(X_{i}-\overset{¯}{X})(X_{i}-\overset{¯}{X}{)}^{T}$ is calculated where $\overset{¯}{X}$ is the mean of the entire image. As the covariance matrix has dimension C×C, C eigenvalues $\lambda_{1},\lambda_{2},\ldots \lambda_{C}$ are extracted. Thus, an eigenvalue diagonal matrix $D=\mathrm{diag}(\lambda_{1},\lambda_{2},\ldots \lambda_{C})$ can be constructed. The transformational matrix A can be computed as the result of the decomposition $\mathrm{Cov}={\mathrm{ADA}}^{T}$. Finally, the principal components can be computed from the original pixel values $Y=A^{T}X$.

Compared with PCA, NMF can decompose hyperspectral data into more interpretable matrices.^89^^,^^90^^,^^114^^,^^124^^,^^129^^,^^292^^,^^318^^,^^342^^,^^346^^–^^352^ The goal is to split the hypercube into two data matrices, both of which contain no negative elements. In practice, NMF solutions are approximated through optimization. Therefore, the time complexity of NMF is determined by the user. NMF is particularly useful when the number of extracted features is known beforehand. For example, Lu et al.^292^ applied NMF to hyperspectral images of tissue to reduce the image size from 61 channels to three features, corresponding to oxyhemoglobin (${\mathrm{HbO}}_{2}$), deoxyhemoglobin (HHb), and light scattering.

Unlike other methods described above, t-SNE is more often used as a visualization tool for post-hoc analysis.^319^^,^^347^^,^^353^^–^^357^ The objective of t-SNE is to create a linear embedding of the data such that data points that are close together in the hyperspectral space are also close together in the embedding space and vice versa. The t-SNE method has a time complexity of $O(N^{2})$. It can be considered an unsupervised clustering algorithm, meaning it does not require labeled data, unlike supervised methods. However, it does not guarantee consistent embedding among different images. To address this, Ravi et al.^126^ proposed a fixed referenced t-SNE (FR-t-SNE) method, which included an optimal fixed reference system such that the manifold embedding can be consistent across multiple images. Fabelo et al.^129^ applied FR-t-SNE to obtain a one-dimensional embedding from a dataset of 750-band images and then used the embedded images as features to feed into a support vector machine (SVM) classifier. Table 2 shows a summary of dimension reduction methods.

**Table 2:** Comparison of dimension reduction approaches. m is the number of channels; n is the number of pixels per channel. Time complexity assumes m is much less than n.

Method	Time complexity	Advantages	Disadvantages
Principal component analysis (PCA)^14^^,^^127^^,^^128^^,^^185^^,^^208^^,^^236^^,^^312^^,^^315^^,^^334^^–^^337^^,^^339^^,^^341^^,^^343^^–^^345^	$O(m^{2}n)$,	Removes noise from data. Optimal variance	Less interpretable data
Negative matrix factorization (NMF)^347^	Depends on user inputs	Features are more interpretable	Components are only approximations. Requires knowing the number of features beforehand
t-Distributed stochastic neighbor embedding (t-SNE)^319^^,^^347^^,^^354^^,^^356^^,^^357^	$O(n^{2})$	Unsupervised. Good for visualization purposes	Mostly for a low number of dimensions. May require additional reduction techniques prior to t-SNE

### 3.3 Band Selection

Band selection methods aim to choose a subset of the existing bands that best retain relevant features in the image. As compared to dimension reduction that are well suitable for downstream classification tasks, band selection makes the data more interpretable.^360^ Band selection methods can be classified into six different categories^360^^,^^361^: (1) rank the bands based on some criterion (ranking-based), (2) select a subset of bands that optimize some downstream metrics (searching-based), (3) cluster the bands then select representative bands (clustering-based), (4) select bands that optimize a sparsity-constrained optimization problem (sparsity-based), (5) select bands that optimize an embedding of the high-dimensional data (embedded-learning based), and (6) combine one or more methods (hybrid-schemes). Within the field of biomedical HSI, the majority of band selection methods fall into either ranking-based, searching-based, or embedding-learning-based methods, which are also called filter, wrapper, and embedded methods, respectively.^304^ Table 3 shows a summary of band selection methods.

**Table 3:** Comparison of band selection methods.

Method	Advantages	Disadvantages
Ranking-based methods^7^^,^^28^^,^^47^^,^^211^^,^^362^^,^^363^	Does not depend on the application	Ignore interactions between bands. May lead to suboptimal selection.
Searching-based methods^304^^,^^364^^,^^365^	Can be fine-tuned for specific purposes	Computationally expensive, especially for large datasets
Embedding-based methods^366^^,^^367^	Deep learning can be a powerful feature extractor	Requires significant computational resources

Ranking-based methods are task-agnostic, which means they select bands based on the intrinsic qualities of the bands, not on the application. A common intrinsic quality to select bands is entropy,^7^^,^^28^^,^^47^^,^^211^^,^^362^^,^^363^ which quantifies the amount of “good” or relevant information. The entropy of a channel is defined as:

$$H=-\overset{2^{b}-1}{\underset{i=0}{\sum }}p_{i}\;\log\;p_{i},$$

where b is the number of bits used to represent the digital image (for an unsigned 8-bit integer data type, b=8), and $p_{i}$ is the probability of a pixel having grey level i.

Gu et al.^363^ described the four steps for entropy-based band selection. First, entropy is calculated for each channel, and the channel with the highest entropy as $c_{1}$ is selected. Next, the channel that has the lower mutual information with $c_{1}$ is identified, and mutual information between two channels $c_{1}$ and $c_{2}$ is calculated using the following equations:

$$MI(c_{1},c_{2})=H(c_{1})+H(c_{2})-H(c_{1},c_{2}),$$

H(c1,c2)=−∑i=0i∈c12b−1∑j=0j∈c22b−1pij log pij,

where $H(c_{1},c_{2})$ is the joint entropy of the channels $c_{1}$ and $c_{2}$. The lowest mutual information channel can provide the most additional information with respect to $c_{1}$. Third, calculate the channel with the minimum dependent of information $\theta_{DI}$ with respect to $c_{1}$ and $c_{2}$. Last, repeat step 3 until enough bands are selected. The equations for $\theta_{DI}$ among $c_{1}$, $c_{2}$, and $c_{n}$ are as follows:

$$\theta_{DI}=H(c_{1},c_{2},c_{n})-\overset{C}{\underset{i=0}{\sum }}H(c_{1}|c_{2},c_{n}),$$

$$H(c_{1}|c_{2},c_{n})=H(c_{1},c_{2},c_{n})-H(c_{2},c_{n}).$$

Wrapper selection methods identify wavelengths that contribute most to the target task. Many such methods solve the problem greedily, i.e., once they find features that improve accuracy, they immediately discard features that do not. Asfour et al.^364^ tested all combinations of wavelengths to find ones that produce the best spectral unmixing results. Ayala et al.^304^ compared the performance of wrapper and filter selection methods for the task of oxygen estimation and found that proper selection can reduce the number of needed wavelengths from 101 bands to 10 to 20 bands. Chang et al.^365^ selected wavelength bands that minimize the mean absolute error of their prediction models.

Embedded learning techniques used neural networks to identify important wavelengths. Full hyperspectral images are used to train the network for the desired task. After training, methods such as saliency mapping can be applied to identify which wavelengths contribute most to the output. Trajanovski et al.^366^ utilized the method to identify relevant wavelengths for tumor detection. Zhang et al.^367^ used saliency mapping to explain the results of tumor segmentation.

### 3.4 Spectral Unmixing

In hyperspectral images, the spectrum at each pixel often represents a mixture of multiple substances or tissue types. Spectral unmixing is to identify individual substances (called “endmembers”) and then estimate their concentration (called “fractional abundance”).^311^ Both linear and nonlinear models are used for spectral unmixing. Linear unmixing models are commonly used due to their simplicity and robustness.^311^ Linear unmixing models assume minimal scattering between components, such that incident light interacts with only one component at a time. Meanwhile, nonlinear unmixing models consider both scattering and nonlinear interactions between light and particles. To simplify a spectral unmixing model, it is also assumed that only several endmembers dominate the hyperspectral scene. Let there be M different endmembers in a scene, $f_{i}$ being the fractional abundance of the endmember i, and $r_{i}(\lambda )$ be the optical absorption spectra of each endmember; then, the spectrum of a single pixel can be modeled as^337^:

$$R(\lambda ,x,y)=\overset{M}{\underset{i=1}{\sum }}r_{i}(\lambda )f_{i}(x,y)+\epsilon .$$

The equation accounts for errors using the error term ϵ. To further improve the abundance estimates, two more constraints can be applied: (1) the nonnegative constraints, which require $f_{i}\geq 0$ ∀  i=1…M, and (2) the additivity constraints, which requires $\overset{M}{\underset{i=1}{\sum }}f_{i}=1$.

Linear unmixing workflows contain three major steps: dimension reduction (Sec. 3.2), endmember determination, and fractional abundance estimation.^311^ Endmembers’ determination can be performed by either supervised or unsupervised methods. In supervised endmember selection, the endmembers and their spectra are known beforehand. For example, blood at different oxygen saturation rates is often modeled and used for supervised linear unmixing.^9^^,^^50^^,^^292^^,^^294^ The absorbance is a function of the concentration of oxyhemoglobin $\left[{\mathrm{HbO}}_{2}](x,y\right)$ and the concentration of deoxyhemoglobin [HHb](x,y). The model accounts for the scattering using a wavelength-independent constant term α:

$$A(\lambda ,x,y)=[{\mathrm{HbO}}_{2}](x,y)\varepsilon_{{\mathrm{HbO}}_{2}}(\lambda )+[\mathrm{HHb}](x,y)\varepsilon_{\mathrm{Hb}}(\lambda )+\alpha .$$

The wavelength-dependent absorbances for ${\mathrm{HbO}}_{2}$ and HHb are taken from known databases.^368^ Ghassemi et al.^294^ used a similar linear model to estimate the concentration of endmembers in a vascular phantom. They added linear contributions of water and resin into the equation. If the spectra of endmembers are not available in any databases, specific regions of interest in the HSI that correspond to pure concentrations of endmembers can be selected.^369^ Ideally, the spectra of the endmembers should bind the spectra of all other pixels.^311^ Once the endmembers are known, the most common method to estimate fractional abundance is through the least squares method.^9^^,^^50^^,^^107^^,^^108^^,^^294^^,^^364^^,^^369^^,^^370^ This method minimizes the distance between the measured absorbance and the estimated absorbance $\left\|A-\overset{M}{\underset{i=1}{\sum }}r_{i}f_{i}\right\|$ subjected to the nonnegative constraints and full additive constraints. The full additive constraint produces closed-form solutions, but these solutions often violate the nonnegative constraints.^311^ To address this, the alternating least squares method can be used.^371^

### 3.5 Image Classification

A common classification task in medical imaging is binary classification, which classifies images as positive (disease) or negative (healthy). Classification performance can be evaluated using different metrics. The simplest metric is accuracy, which is the number of correct predictions as a percentage of the total number of predictions. However, accuracy can be misleading under class imbalance, i.e., when one class is more prevalent than the other. Sensitivity, also known as recall, is the percentage of positive data that is correctly predicted out of the total positives. Specificity is the percentage of negative data that is correctly predicted out of the total negatives. Precision is the percentage of positive predictions that are correctly predicted out of all predictions, including both positives and negatives. An F1 score combines precision and recall using the following equation:

$$F1=2\times \frac{\text{precision}\times \text{recall}}{\text{precision}+\text{recall}}.$$

The area under the receiver operating characteristic curve (AUC) is a measure of overall performance that considers both sensitivity and specificity.

Beyond reporting performance metrics, it is also important to consider generalization, i.e., how reliably a classifier performs on unseen data, which is particularly relevant in biomedical HSI where data are often high dimensional, and dataset sizes can be limited. This challenge is commonly discussed in terms of the bias-variance trade-off.^372^ In general, models with higher complexity can better predict data they have seen (smaller “bias”), but this will subsequently reduce their ability to predict unseen data (higher “variance”).^372^ Notably, in DNNs, a surprising observation occurs: if the complexity of the model keeps increasing beyond the amount of training data, the model suddenly performs better on unseen data.^373^ This phenomenon is known as double descent.^373^ This observation has motivated the development of deep learning algorithms that increasingly leverage larger datasets and higher-capacity models.

#### 3.5.1 Traditional machine learning–based classification

Machine learning algorithms can be divided into different approaches. If an algorithm requires pairs of input vectors and corresponding targets for training, it falls under the category of supervised machine learning. By contrast, if target labels are not available, the algorithm is considered unsupervised machine learning.^372^^,^^374^^,^^375^ Table 4 highlights traditional machine learning models that have been used in medical hyperspectral image analysis. We separated our discussions into two broad categories: supervised learning and nonsupervised learning (unsupervised, semi-supervised, reinforcement learning, and anomaly detection).

**Table 4:** Comparison of traditional machine learning methods for medical hyperspectral imaging. In this table, m is the number of features (channels or pixels), n is the number of data points in the training class, t=min(m,n) (LDA only), d is the specified depth of the decision tree in cases of linear discriminant and decision tree classifier, c is the number of classes (Naïve Bayes, LDA, and QDA), s is the number of support vectors (SVM only), i is the number of iterations (k-means only), r is the number of principle components (PCA only). LDA, linear discriminant analysis; QDA, quadratic discriminant analysis; DBSCAN, density-based spatial clustering of applications with noise; PCA, principal component analysis.

Algorithms	Training time	Inference time	Advantages	Disadvantages	Ref.
Supervised learning					
Linear regression	$O(nm^{2}+m^{3})$	O(m)	Simple and easy to implement, interpretable results	Only works for linearly separable data	48, 51, 66, 101, 181, 183, 194, 262, 330, 376–392
Logistic regression	O(mn)	O(m)	Good for binary classification, efficient for large datasets	Assumes linear decision boundaries	11, 74, 85, 201, 227, 258, 262, 286, 325, 347, 383, 393–405
Naïve Bayes	O(mn)	O(cm)	Fast, working well with high-dimensional data	Assumes independence among features, which may not hold	74, 258, 326, 402, 406–409
Support vector machine	$O(n^{3})$	O(sm)	Effective in high-dimensional spaces, works well for complex, nonlinear boundaries	Memory-intensive, slower for large datasets	24, 80, 410, 160, 162, 187, 256, 287, 357, 402, 404, 409, 411
Linear discriminant analysis (LDA)	$O(mnt+t^{3})$	O(cm)	Works well for linearly separable classes, simple to implement	Assumes normal distribution of features	30, 101, 328, 394, 397, 402, 412–421
Quadratic discriminant analysis (QDA)	—	—	Works for nonlinearly separable data	Assumes quadratic distribution of features	413, 415
K-nearest neighbors (KNN)	O(1)	O(nm)	Simple, no training required	High memory and computation costs for large datasets	74, 185, 187, 300, 325, 329, 343, 344, 357, 394, 397, 406, 409, 415, 422–430
Decision tree	O(mn log(n))	O(d)	Easy to interpret, handles both numerical and categorical data	Prone to overfitting without pruning, unstable	8, 12, 41, 68, 74, 119, 187, 229, 258, 389, 402, 409, 414, 415, 431–435
Unsupervised learning					
K-means	O(mnki)	O(km)	Simple to implement, scalable to large datasets	Requires pre-determined number of clusters, sensitive to outliers	9, 25, 41, 102, 119, 122, 138, 162, 185, 208, 227, 282, 315, 323, 343, 344, 408, 425, 436–449
Hierarchical clustering	$O(n^{3})$	—	No need to pre-specify the number of clusters	Computationally expensive for large datasets	450
Density-based spatial clustering of applications with noise (DBSCAN)	O(n log(n)) (average); $O(n^{2})$ (worst case)	—	Identifies clusters of arbitrary shapes, robust to outliers	Not effective for clusters with varying densities	451
Principal component analysis (PCA)	$O(nm^{2})$	—	Reduces dimensionality, removes redundancy	Linear method, may lose important information in nonlinear data	11, 14, 36, 81, 126–128, 185, 208, 220, 236, 312, 315, 334–345

##### Supervised learning–based classification

Supervised machine learning techniques require pairs of inputs and desired outputs to train the model. Given the input data X and output data Y, the goal of supervised learning is to approximate a function f such that Y≈f(X). To quantify the correctness of the approximation during training, a loss function is used. For example, a common loss function in binary classification is the binary cross-entropy (BCE):

$$L_{\mathrm{BCE}}(y,f(x))=-[y\cdot \log f(x)+(1-y)\cdot \log(1-f(x))].$$

The goal of training is to minimize loss functions, either through closed-form solutions or through optimization methods such as gradient descent. Supervised classifiers are often grouped as discriminative or generative models (e.g., perceptron, logistic regression model, SVM, and decision tree). Discriminative classifiers draw boundaries that best separate classes of data (e.g., linear discriminant analysis (LDA), quadratic discriminant analysis (QDA), Naïve–Bayes classifier). Generative classifiers study the distribution of data within each class. Some discriminative classifiers rely on linear parameters to draw their decision and are also called linear classifiers (e.g., linear SVM and perceptron).

A combination of low-complexity models can be grouped together into an ensemble model. Ensemble methods can outperform their constituents, given that the model types are diverse, and/or that they are trained on different data. Ensemble techniques include bagging, boosting, and stacking models.^452^ Ensemble approaches can also be applied with deep learning models.

##### Unsupervised learning–based classification

Unsupervised learning does not require labeled data and instead aims to extract commonality in the training data. Methods of unsupervised learning include dimension reduction (Sec. 3.2), anomaly detection, reinforcement learning, and clustering. In addition, there are techniques that rely on partially labeled data or imprecisely labeled data, such as semi-supervised learning, weakly supervised learning, and meta-learning.^346^^,^^453^

There are several classical clustering methods, each with its own strengths and applications. k-means clustering is one of the most popular methods due to its simplicity and efficiency.^41^^,^^119^^,^^122^^,^^443^ Here, we reviewed several different goals of k-means clustering. The first is to identify diseased tissue from healthy tissue. Aboughaleb et al.^444^ used k-means clustering with eight clusters to identify connected regions of tissue that are similar spectrally, a technique known as contour mapping. They applied the technique to *ex vivo* breast tissues, taken with a hyperspectral camera in the wavelength range 400 to 1000 nm. They found that the wavelengths 420 to 500 nm have the greatest differences in reflectance intensity between normal and cancerous breast tissues. Leon et al.^119^ combined k-means clustering with SAM to classify melanoma and benign nevus tissues in hyperspectral images. Pixels were grouped into clusters based on spectral features, and then, these clusters were assigned labels of “tumor” or “healthy” by comparing the cluster spectra to known reference spectra. Khouj et al.^122^ applied k-means to identify ductal carcinoma *in situ* in hyperspectral reflectance images of breast tissue biopsies. The authors used the value of k=2, which was lower compared to studies discussed in the previous sentences. Without any manual labels, the algorithm grouped pixels into tumor and normal regions and achieved a sensitivity of ∼85.5% and specificity of ∼94.6%, as verified by a pathologist-annotated ground truth. Another use of k-means is to identify and classify different types of tissues or cell structures. Ogi et al.^41^ used a variant of k-means known as k-means++,^454^ which improves centroid initialization over standard k-means. Hyperspectral images of neural stem cells were acquired across a wavelength range of 420 to 730 nm. The authors initially clustered the image into 16 groups, which were subsequently consolidated into four classes (neuronal cell body, glial cell body, process end, and extracellular region), achieving an average accuracy of 88% and precision of 94%. k-means was also used to identify different physiological sections of tissues. Fabelo et al.^344^ used hierarchical k-means in conjunction with DNN to generate unsupervised classification maps of four classes (tumor, normal, hyper-vascularized tissue, and background). They applied the method on hyperspectral images of *in vivo* brain tumors in the range 400 to 1000 nm, achieving an accuracy of 95% for binary classification and 85% for multiclass classification. Aref et al.^443^ used k-means on the image of *ex vivo* ablated livers in the wavelength range 400 to 1000 nm. They aimed to identify eight different tissue zones, including those directly ablated and those that remained normal. They observed clear differences in reflectance intensity among the zones, with the normal zone exhibiting an intensity of 23 a.u. (arbitrary unit), thermal zone 1 at 42 a.u., and thermal zone 2 at 55 a.u. Besides unsupervised learning, k-means is also used for dimension reduction (Sec. 3.2) and image segmentation (Sec. 3.7.1). Unlike k-means, hierarchical clustering does not require the number of clusters to be specified upfront and can produce a nested sequence of clusters. Our review found hierarchical clustering often combined with k-means to produce hierarchical k-means.^185^^,^^343^^,^^344^^,^^425^ For example, Kumar et al.^347^ presented a hierarchical clustering approach to MIR hyperspectral images of tissue samples to enhance the segmentation of sub-cellular structures. Their method achieved an unsupervised F1 score of 71.18% on twofold cross-validation.

Another method for unsupervised clustering is density-based spatial clustering of applications with noise (DBSCAN).^455^ DBSCAN is particularly effective in identifying clusters of arbitrary shape and handling noise within the data. Lucas et al.^451^ developed a wide-field HSI microscopy system to measure blood vessel oxygenation and employed DBSCAN for automatic vessel segmentation. After detecting vessel edge points via an edge detector, DBSCAN was applied to cluster the edge pixels into two groups corresponding to the upper and lower vessel boundaries. Their results demonstrated that DBSCAN effectively segmented blood vessels in the hyperspectral image.

#### 3.5.2 Deep learning–based classification

The deep learning revolution in the mid-2010s improved the classification accuracy of numerous tasks such as image recognition, natural language, sound, image generation, and diagnosis. Deep learning networks can extract complex features from inputs through intermediate layers without as much expert input as compared with traditional machine learning techniques. However, due to the large number of parameters, training also requires a substantial amount of input data. Many researchers utilize pre-trained neural networks, which have been trained on a large corpus and can be tuned on novel data. In this section, we provide an overview of commonly used deep learning architectures, followed by a discussion of training strategies, including supervised and unsupervised machine learning. We then examine techniques for adapting deep learning architecture to the use of hyperspectral imaging. Please note that the scope of this review is only limited to publications within the biomedical field and not the entire field of hyperspectral imaging. Interested readers should consider a comprehensive review of deep learning in hyperspectral imaging by Li et al.^456^ We recognized that advancements were first published in the remote sensing field, using publicly available remote sensing datasets.

##### Supervised deep learning–based classification

Most supervised deep learning networks used for hyperspectral images are convolutional neural networks (CNNs). CNNs rose to prominence in 2012 when AlexNet achieved state-of-the-art performance in classifying everyday images. A simple CNN has multiple convolution blocks in succession of each other, in conjunction with pooling and activation layers. Convolution blocks identify low-level features at the beginning layers, such as edges and corners, and identify more complex features the deeper they go. In the biomedical HSI field, researchers have utilized CNN layers which can be two-dimensional (2D CNN),^11^^,^^185^^,^^343^^,^^345^^,^^394^ three-dimensional (3D CNN),^11^^,^^24^^,^^28^^,^^71^^,^^73^^,^^319^^,^^329^^,^^353^^,^^356^^,^^418^^,^^457^ or a hybrid of both (hybrid).^418^^,^^457^ For brevity, we discuss only representative uses within each architecture. Using 2D-CNN for feature extraction was seen in earlier literature.^11^^,^^185^^,^^343^^,^^345^^,^^394^ Fabelo et al.^343^ employed a simple band selection scheme to select three representative bands from the hyperspectral images to train a fully convolutional CNN. They trained their network on 26 *in vivo* hypercubes and achieved an overall accuracy of 80% for multiclass classification. Halicek et al.^185^^,^^345^^,^^394^ modified the inception network for HSI classification by changing the size of the filters in the first convolution layer. They^458^ used the model to identify*ex vivo* thyroid and salivary tumors, resulting in an AUC score of 0.90 for thyroid tumor classification and an AUC score of 0.92 for salivary gland tumor classification. Three-dimensional CNNs are particularly useful for HSI as they can analyze both spatial and spectral dimensions simultaneously.^11^^,^^24^^,^^28^^,^^71^^,^^73^^,^^319^^,^^329^^,^^353^^,^^356^^,^^418^^,^^457^ Hu et al.^11^ applied 3D-CNN to an HSI database of human gastric cancer. They found that 3D-CNN is fully capable of extracting features without the need for PCA dimension reduction. The model got an accuracy of 97.57% and a sensitivity of 97.19%. More recently, Eggert et al.^73^ applied 3D-CNN in a DenseNet architecture. They used the network for classifying head and neck cancer with hyperspectral images of laryngeal, hypopharyngeal, and oropharyngeal mucosa, achieving an accuracy of 91% and a sensitivity of 83%.

A key limitation of CNNs in biomedical imaging is the scarcity of large, labeled datasets, which are often costly and time-consuming to generate. To address this, researchers have turned to techniques such as data augmentation and transfer learning. Multiple augmentations can be combined using methods such as trivial augment^359^ or RandAugment.^459^ In classification tasks, label smoothing can also be used to account for incorrect labels in the ground truth data.^359^ Transfer learning helps overcome limited data by fine-tuning pretrained models for HSI tasks, allowing CNNs to achieve better performance even with small datasets.^39^^,^^134^^,^^211^^,^^260^^,^^339^^,^^353^^,^^356^^,^^357^^,^^359^^,^^418^^,^^420^^,^^434^^,^^460^^–^^462^ Table 5 lists the neural networks that were used in hyperspectral image processing.

**Table 5:** List of neural network architectures used in the processing of hyperspectral images. All trainable parameters refer to the base version of the network.

Architecture	Notable features	Trainable parameters (millions)
AlexNet^207^^,^^339^^,^^343^^,^^414^^,^^463^^,^^464^	Convolutional neural network	61
VGG^149^^,^^207^^,^^464^^–^^469^	Very deep networks	144 (VGG-19)
ResNet^25^^,^^56^^,^^207^^,^^211^^,^^260^^,^^319^^,^^359^^,^^418^^,^^433^^,^^462^^,^^464^^,^^469^^–^^474^	Skip connections	45 (ResNet-101)
Inception^185^^,^^203^^,^^319^^,^^345^^,^^414^^,^^416^^,^^424^^,^^458^^,^^463^^,^^472^^,^^475^^–^^477^	Convolution at different scales	24 (Inception V3)
MobileNet^211^	Depthwise separable convolutions	3.5 (MobileNet V2)
U-Net^462^^,^^478^	Skip connections	31 (Vanilla U-Net)
Transformer^479^^–^^481^	Self-attention layer	87 (Vision transformer)
		121(TimeSformer)

Attention mechanisms and transformers are becoming key components in biomedical HSI, offering a way to enhance feature extraction by focusing on the most relevant parts of the data.^432^^,^^434^^,^^435^^,^^479^^,^^482^^,^^483^ Wang et al.^482^ applied an attention mechanism on a 3D convolutional network for white blood cells classification. The attention module increased accuracy from 96% to 97.2%. Wang et al.^435^ employed a hyperspectral attention mechanism to select features for radiomics. Their method improved radiomics prediction on liver cancer HSI data and achieved an AUC score of 0.96. Transformers are a type of network that uses the attention mechanism as its backbone. The use of transformers in biomedical HSI remains limited.^479^^,^^480^^,^^484^^,^^485^ La Salvia et al.^479^ used the vision transformer as a base for the classification of benign and malignant skin lesions using a dataset of 76 hyperspectral images. The model achieved an accuracy of 91% and a sensitivity of 99% for malignant melanoma (MM), but an accuracy of only 41% and a sensitivity of 5% on malignant epithelial. Tran et al.^480^^,^^485^ used a modified vision transformer to identify malignant thyroid carcinoma cells in hyperspectral images of H&E-stained slides. The vision transformer achieved an accuracy of 90.87%, outperforming both ConvNeXt and the vision transformer trained on RGB data.

##### Unsupervised deep learning–based classification

Autoencoders (AE) are widely used in hyperspectral biomedical imaging for unsupervised feature extraction and dimension reduction.^407^^,^^439^^,^^460^ The primary objective of an AE is to minimize the difference between the input and the reconstructed output, allowing the network to capture key features of the data while discarding irrelevant information. Ma et al.^407^ used an AE structure for feature extraction on their dataset of head and neck hyperspectral images. Using the AE for pre-training, they achieved a sensitivity of 92.3% and a specificity of 91.3%. Zelger et al.^460^ employed a convolutional autoencoder (CAE), a variant of AE that has convolutional layers, for feature extraction of MIR (2560 to 11,760 nm) images of human lymphomas and achieved an accuracy of 90% for binary lymphoid carcinoma classification. In self-supervised learning (SSL), the model is trained in a surrogate task, where the labels are generated directly from the data itself. Zhang et al.^211^ applied SSL using the SimSiam framework to pretrain models on hyperspectral images for the classification of precancerous lesions in gastric cancer. In this approach, two augmented hyperspectral images are input into shared encoders, consisting of a CNN and a multilayer perceptron (MLP). The cosine similarity of the two outputs is computed, and a stop-gradient is applied to prevent collapse during training. Xie et al.^486^ proposed the self-supervised spectral regression method. In their approach, the authors mask out one spectral band at a time and task the network with predicting it from the remaining bands. When their learning model was later fine-tuned on small, labeled datasets for cancer classification in pathology slides, it achieved up to 14% improvement in accuracy compared with networks trained from scratch or with generic SSL methods.

### 3.6 Hyperspectral Image Generation and Enhancement

Generative models can generate new hyperspectral images, often as a modification of the inputs. In recent years, the invention of the generative adversarial network (GAN) helps produce images with high spatial and spectral quality. Introduced by Goodfellow et al.,^487^ GAN was developed to solve the problem of unrealistic image generation. By introducing a discriminator in addition to the generator, GAN can drive generated images more toward the proper distribution of realistic data. Over the years, many types of GAN have developed from the original GAN, including conditional GAN, cycle GAN, or Wasserstein GAN. We do not review diffusion models because there is not enough research that applied diffusion models to biomedical hyperspectral images. Metrics to judge generated images often require supervised ground truth. To compare the spatial details of the generated image, root mean square error (RMSE), structural similarity index measure (SSIM), peak signal-to-noise ratio (PSNR), and perceptual VGG loss can all be used.^488^ For unsupervised image generation (no ground truth available or accessed during training), independent expert judgements can be a useful method that qualitatively assesses the output of the generated image. The main applications of image generation are to generate more synthetic data for training neural networks,^489^^,^^490^ to generate super-resolution images,^155^^,^^156^^,^^203^ and to generate synthetic chemically stained images.^491^

#### 3.6.1 Super-resolution hyperspectral imaging

As described previously, hyperspectral image acquisition often requires trade-offs. Acquiring a hypercube requires significantly more light to enter the sensor compared with that of a three-channel RGB image. Although significant progress has been made in creating faster push-broom or snapshot cameras with higher spatial resolution, the dilemma remains. The remote sensing community solved this problem by augmenting low-resolution HSI with high-resolution panchromatic images; the process is called super-resolution reconstruction or pansharpening. There are several approaches toward producing a pansharpened image: component substitution, multiresolution analysis, and deep learning methods.^488^ Deep learning methods provide the most robust and adaptable super-resolution image for analysis.^203^^,^^492^^–^^495^ However, training deep learning networks often requires supervised data—images that are already hyperspectral and high resolution. In many cases, the supervised ground truth is not available. Here, we reviewed methods of pansharpening specific to biomedical imaging applications—the problem is often named “super-resolution” within the biomedical imaging field and “pansharpening” in the remote sensing field.

**Fig. 8:**
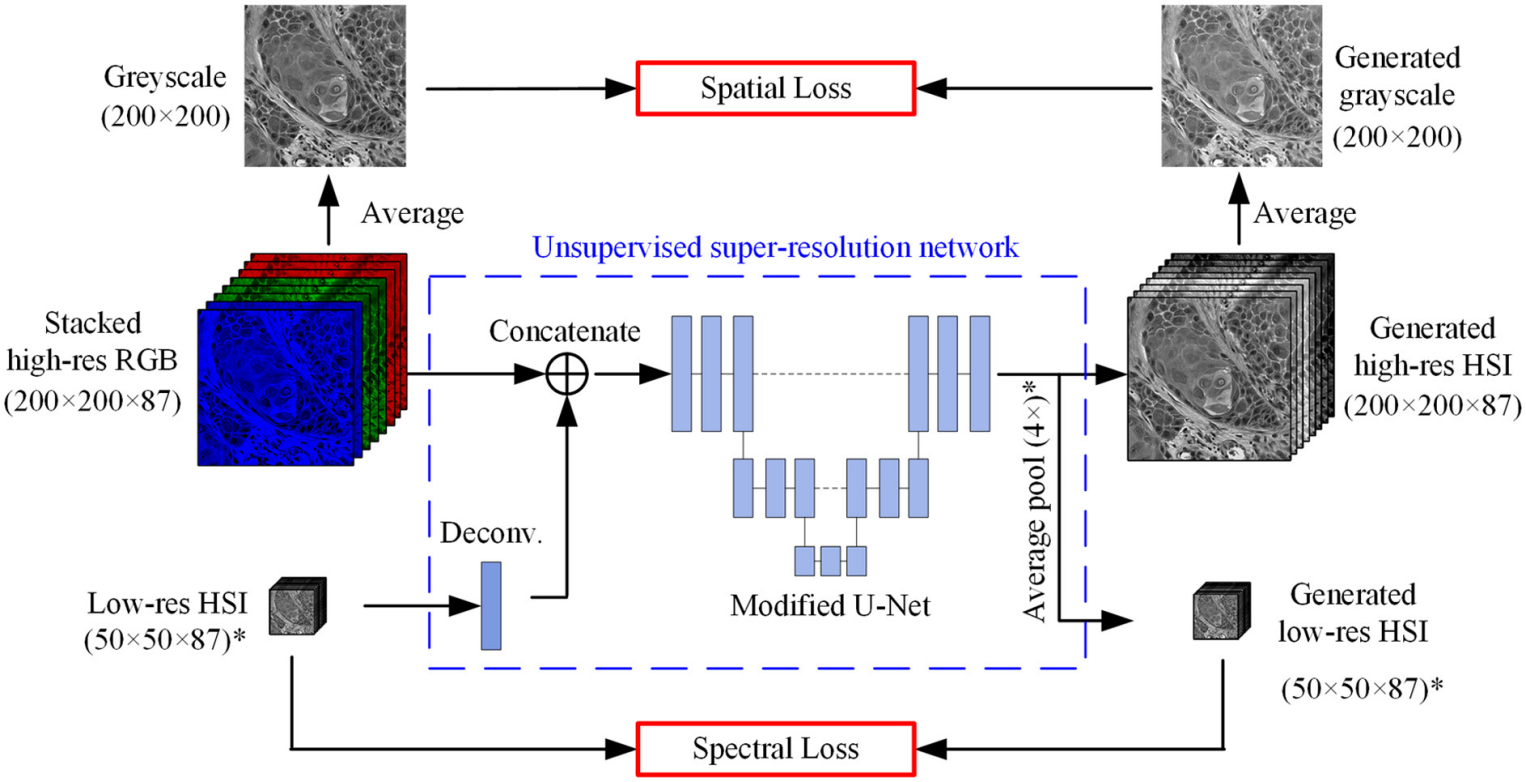
Unsupervised super-resolution reconstruction neural network. Figure reproduced with permission from Ma et al.^203^

The workflow of many traditional methods is very similar. The high-resolution image is run through a high-pass filter, which extracts the detail components. The details are then added to the low-resolution image, upsampled to match the dimensions of the high-resolution one. The algorithms differ in how the spectral details and the spatial details are fused together. Jiang et al.^10^ tested several classical pansharpening algorithms, component substitution included, to combine high-resolution RGB images and low-resolution hyperspectral images. They produced super-resolution images for their hyperspectral microscope. Dey et al.^496^ provided a novel image fusion method, which used convex optimization, to generate super-resolution histology hyperspectral images. Ma et al.^203^ used deep learning for their super-resolution method. They used a U-Net architecture (Fig. 8) as the backbone for unsupervised super-resolution learning. They successfully applied the network to histology images^203^ as well as tissue images acquired with a surgical microscope and an endoscope.^155^^,^^156^ Applications for pansharpening in biomedical imaging are still few, in spite of their large potential.

#### 3.6.2 Virtual staining with hyperspectral imaging

**Fig. 9:**
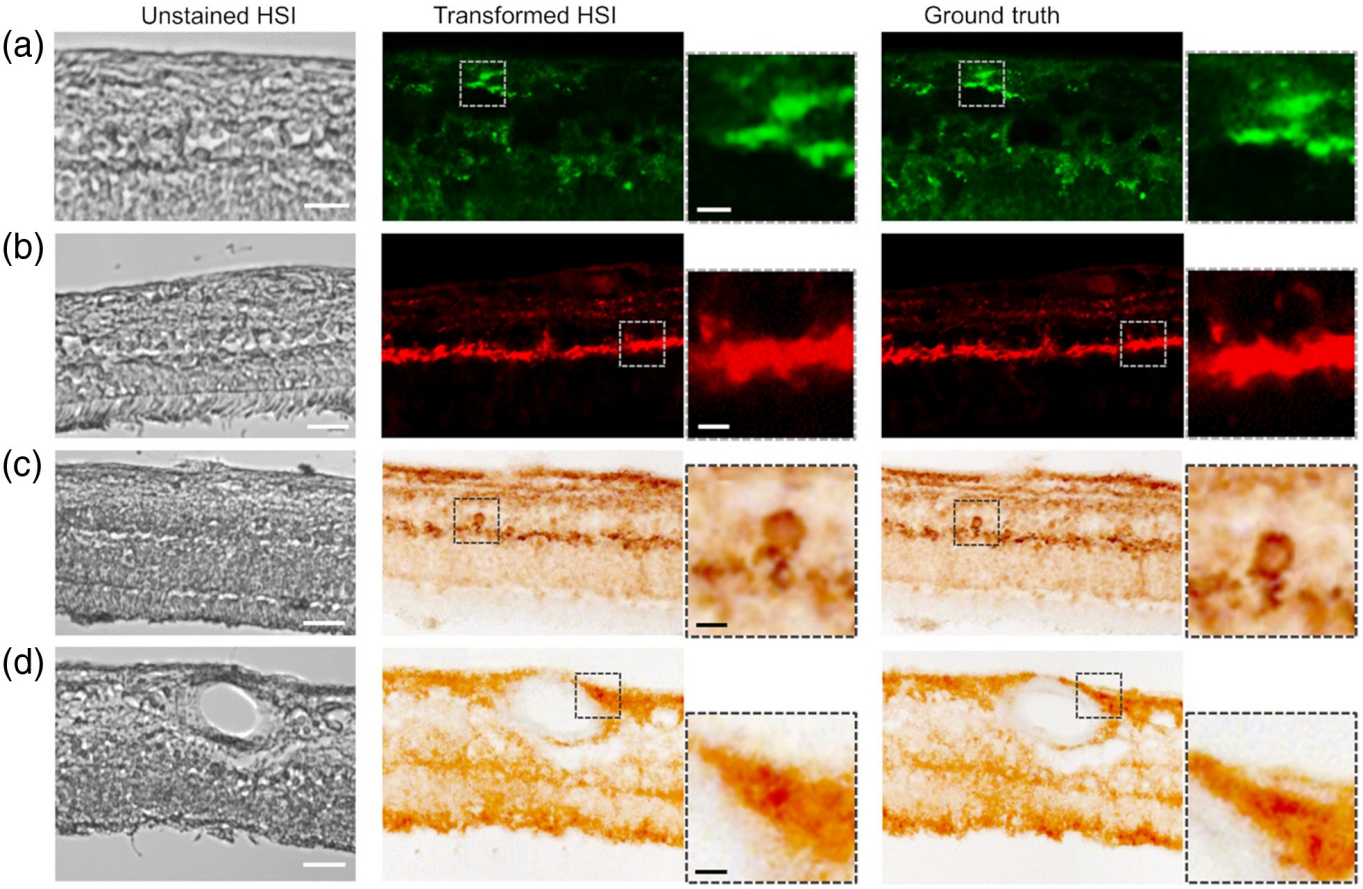
Results of virtual staining using hyperspectral images achieved by Du et al.^60^ The authors trained a GAN to generate images $A\beta_{42}$ fluorescence images (a), pS396-Tau (b), pS396-Tau DAB stain (c), and $A\beta_{42}$ DAB stain (d). Scale bar, 50  μm for large FOV images, 10  μm for bordered inserts.

In histology, stains are used to highlight different structures of the cells. Hematoxylin and eosin are the most common stains used to highlight the differences between nuclei and the extracellular matrix. Immunostaining is the use of specific antibodies to bind to antigens in tissues. The antibodies can be highlighted using diaminobenzidine (DAB) or fluorescence imaging. Staining helps researchers highlight different structures easily compared with the unstained slide. However, staining requires the slide to be embedded and dehydrated, which is a time-consuming process. Using HSI cameras, a hyperspectral image of the unstained slide can be acquired, and then virtual staining can be applied to generate artificial staining. Surprisingly, digital staining was initially proposed as a concept in 2005 by Bautista et al.^497^ using three different steps: segmentation of tissue in an unstained tissue image, classification of segmented tissue, and visualization of tissue using false coloring. The hyperspectral image was spectrally compressed using PCA, and then, the k-nearest neighbors algorithm was used for the classification of tissues. Bayramoglu et al.^498^ proposed using a conditional GAN to generate pseudo H&E-stained images. The authors were able to generate H&E-stained lung tissue samples directly from unstained data. GAN remains the state-of-the-art method to generate virtual stained slides.^60^^,^^491^^,^^498^^–^^500^ The workflow is similar across different methods. First, the tissue is embedded and sectioned into thin slides. Then, the unstained slide is imaged using an HSI camera. After, the slide is stained with either H&E or immunohistology stains. The unstained and stained images were used as training for the generator and discriminator pair. Qualitative comparison is made on the paired test data. Outside of H&E staining, virtual DAB and fluorescence staining of amyloid beta proteins and phosphorylated tau was also proposed by Du et al.^60^ (see Fig. 9).

### 3.7 Hyperspectral Image Segmentation

The techniques for segmentation have evolved significantly over the years, starting with simple classical methods that rely on basic image properties to sophisticated deep learning models that can understand complex, high-level information within an image. In hyperspectral image analysis, segmentation was used to identify glare,^17^ background regions,^25^^,^^344^ tumors or diseased segments,^442^^,^^471^ or different classes of tissues.^465^ In this paper, we reviewed the use of traditional methods and deep learning–based methods in biomedical HSI, divided into supervised and unsupervised learning.

To perform supervised segmentation tasks, annotations made by professionals have to be provided beforehand. Common metrics in segmentation include the Jaccard score (intersection-over-union) and the Hausdorff distance. Jaccard score is defined as the ratio of pixels intersecting over the total number of pixels. If we let A be the segmentation made by the predictor and B be the ground truth segmentation, then the Jaccard score is defined as

$$J(A,B)=\frac{\left|A\cap B\right|}{\left|A\cup B\right|}.$$

The Hausdorff distance is used to give context to the classification by attaching a physical dimension. It is defined as the largest distance between two discrete morphological segments A and B:

$$d_{H}(A,B)=\max\{h(A,B),h(B,A)\},$$

$$h(A,B)=\underset{a\in A}{\max}\;\underset{b\in B}{\min}\;d(a,b),$$

where d(a,b) is the Euclidean distance between point a and point b. Intuitively, the Hausdorff distance captures the greatest discrepancy between the two sets, making it a robust measure of the worst-case difference between the boundaries. It can be seen as a good metric for the assessment of tumor margin predictions.^478^

#### 3.7.1 Traditional machine learning–based segmentation

We reviewed segmentation methods applied that were invented prior to deep learning. These included methods that were based on thresholding and clustering. Thresholding produces segmentations based on pixel intensity above or below a threshold. Hyperspectral images improve the quality of thresholding by including a threshold at different wavelengths. For example, blood vessels can be segmented by focusing on the wavelength 500 to 600 nm, where blood has a stronger absorption compared with the background tissues.^48^ For automatic thresholding, the Otsu method can be used to determine the threshold value.^68^^,^^147^^,^^445^^,^^501^ However, the Otsu method is only applicable for a single channel.^147^ Wang et al.^147^ proposed a method to extend the Otsu algorithm onto the multichannel shape of HSI. Another method is watershed segmentation. The watershed segmentation algorithm is a region-based image segmentation technique inspired by the concept of watersheds in topography. It is particularly useful in separating overlapping objects or regions in an image, such as histological cells.^27^^,^^427^

Often, individual pixels can be fed into a supervised or unsupervised classifier, and then, the classification results are grouped into segmentations. Using pixel-wise classification, SVM,^126^^,^^187^^,^^287^^,^^502^ SAM,^11^^,^^140^^,^^186^^,^^196^^,^^203^^,^^229^^,^^233^^,^^234^^,^^423^^,^^503^^–^^505^ and random forest (RF)^126^^,^^187^ have all been used for segmentation purposes. In terms of unsupervised segmentation, hierarchical clustering^129^^,^^347^^,^^506^ and k-means clustering^26^^,^^29^^,^^102^^,^^119^^,^^131^^,^^146^^,^^227^^,^^344^^,^^406^^,^^438^^,^^442^^,^^444^^,^^445^^,^^448^^,^^449^^,^^507^ have been employed. Particularly, our review found that k-means clustering is a popular method. It works by partitioning the image into k clusters based on the intensity values of the pixels. However, one limitation of k-means is that it assumes that the clusters are spherical and of equal size, which is not always the case in real-world images, especially in medical contexts where anatomical structures vary greatly in shape and size. Moreover, the algorithm is sensitive to the choice of k, and an inappropriate selection of k can lead to poor segmentation results. Simple linear iterative clustering (SLIC) is an algorithm that generates superpixels based on clustering. SLIC produces segmentation that is less sensitive compared with traditional k-means.^406^^,^^442^^,^^507^ For example, Ortega et al.^442^ leveraged SLIC to detect glioblastoma tumors. They found that their method can detect brain tumors with an average accuracy, specificity, and sensitivity of 83%, 87%, and 81%, respectively. They compared their method with previous deep learning methods and found similar performances in terms of accuracy and sensitivity.

#### 3.7.2 Deep learning–based segmentation

**Fig. 10:**
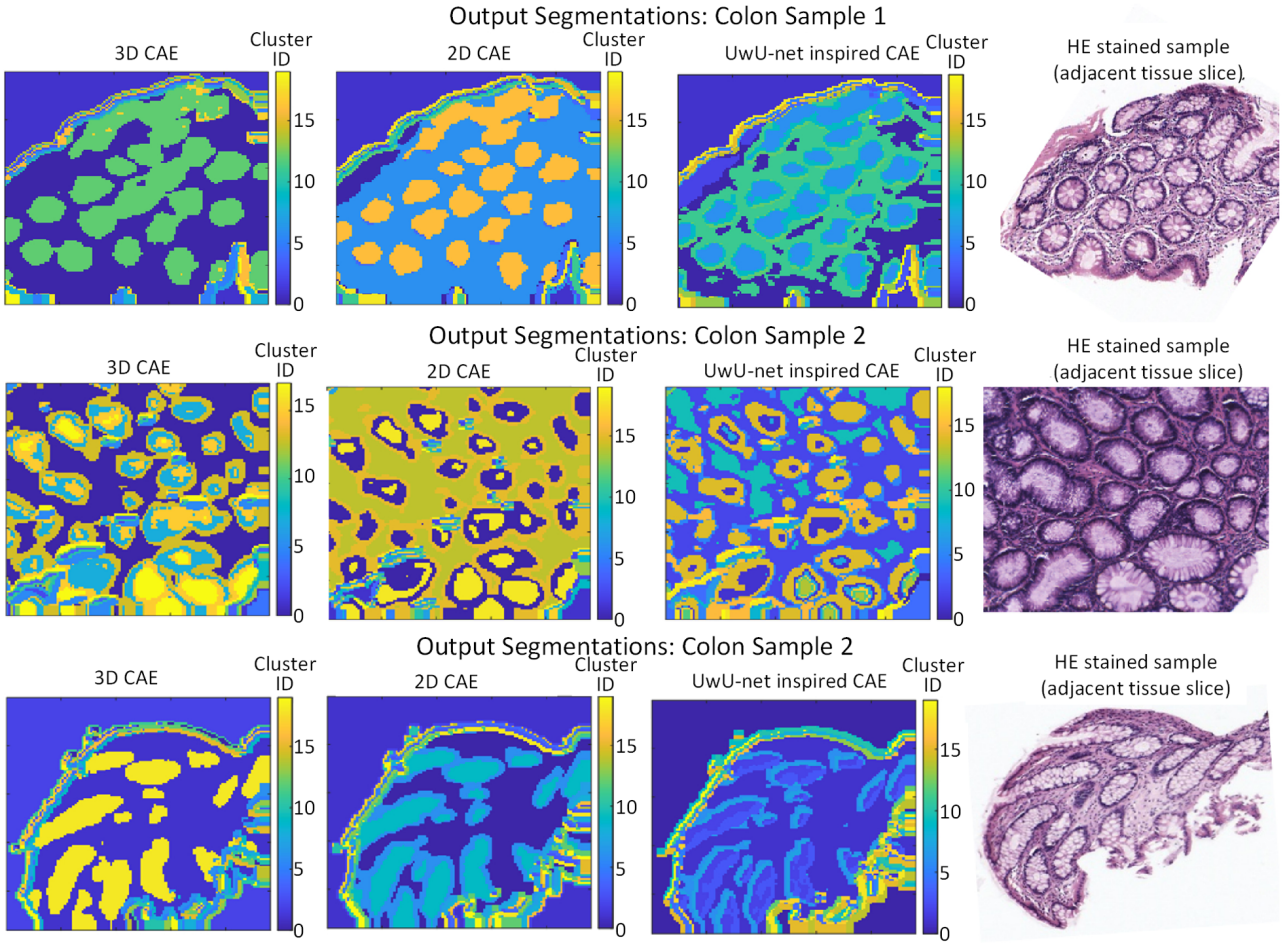
Unsupervised segmentation of H&E-stained histology samples of human colon with different network architectures by Bench et al.^439^ From left to right: segmentation using a 3D convolutional autoencoder (CAE), segmentation using a 2D CAE, and segmentation using a variant of UwU-Net. Different colors refer to different segmentation classes.

Artificial neural networks can be used as a pixel-wise classifier in a similar manner to previous machine learning techniques.^508^^,^^509^ Powerful deep learning networks leverage fully convolutional network (FCN) structures.^17^^,^^328^^,^^343^^,^^366^^,^^510^ The FCN structure allows them to efficiently handle input images of arbitrary size by applying convolutional operations throughout the network. FCNs have been the foundation for many advanced deep learning–based segmentation architectures, such as U-Net^511^ and SegNet.^512^ U-Net architecture follows an encoder–decoder structure, where the encoder extracts feature representations through convolutional and pooling layers, progressively reducing the image resolution. The U-Net design with skip connections enables it to achieve highly accurate pixel-wise segmentation and was used by many HSI researchers for segmentation tasks.^17^^,^^328^^,^^343^^,^^366^^,^^510^^,^^513^ In biomedical HSI, U-Net was used to segment glioblastoma tumors,^343^ breast tumors,^328^ tongue carcinoma,^510^ and liver tissues.^510^ A variant of U-Net called UwU-Net was used by Bench et al.^439^ for the task of unsupervised hyperspectral histology segmentation (Fig. 10). The UwU-net inspired architecture first processes input HSI patches with 2D convolutional layers, reducing the data into fewer activation maps. Each activation map is then individually fed into a separate inner U-Net to extract specialized spatio-spectral features. The authors found that the segmentation results from the UwU-net architecture significantly outperformed CAE in synthetic data but were not significantly better when it comes to real colon HSI data. Dilated convolution is seen in the DeepLab architecture, which was used by Zenteno et al.^163^ to segment out the fiber optic instruments from the hyperspectral biopsy images. Table 6 summarizes the methods for HSI segmentation that are discussed in Sec. 3.7, including different traditional, machine learning, and deep learning approaches.

**Table 6:** Overview of HSI segmentation approaches.

Category	Method	Segmentation mechanism
Traditional	Spectral thresholding^48^	Threshold at selected wavelength(s)
	Otsu thresholding^68^^,^^147^^,^^445^^,^^501^	Automatic single-band thresholding can be extended to multichannel
	Watershed^27^^,^^427^	Region-based separation of touching objects
Supervised machine learning	SVM^126^^,^^187^^,^^287^^,^^502^	Pixel-wise classification, then grouped into segments
	SAM^11^^,^^139^^,^^140^^,^^186^^,^^196^^,^^203^^,^^229^^,^^233^^,^^234^^,^^423^^,^^483^^,^^503^^–^^505^	
	RF^126^^,^^187^	
Unsupervised machine learning	Hierarchical clustering^129^^,^^347^^,^^506^	Builds a dendrogram (linkage-based); clusters from a chosen cut level
	k-means clustering^26^^,^^29^^,^^102^^,^^119^^,^^131^^,^^146^^,^^227^^,^^344^^,^^406^^,^^438^^,^^442^^,^^444^^,^^445^^,^^448^^,^^449^^,^^507^	Iterative centroid-based partitioning into k clusters in spectral space
	SLIC^406^^,^^442^^,^^507^	Superpixel clustering using spectral similarity and spatial proximity
Supervised deep learning	FCN^17^^,^^328^^,^^343^^,^^366^^,^^510^	Fully convolutional pixel-wise prediction
	SegNet^512^	Encoder–decoder FCN architecture
	U-Net^328^^,^^343^^,^^510^^,^^511^	Encoder–decoder + skip connections
	DeepLab^163^	Dilated convolutions
Unsupervised deep learning	UwU-Net^439^	Patch compression + multiple inner U-Nets

## 4 Hyperspectral Imaging in Medical and Biological Applications

HSI is noncontact, noninvasive, nonionizing, and label-free and therefore can have some unique advantages for biological and medical applications. HSI has been increasingly explored for disease diagnosis and image-guided surgery. HSI utilizes the optical properties of tissue, including absorption, scattering, and fluorescence, which are essentially determined by the molecular composition and morphological changes of tissue. Among various optical properties, reflectance and fluorescence (mainly autofluorescence), or the combination of both, are most commonly used. Despite the wide available wavelength range from UV to MIR, medical HSI mainly utilizes visible and NIR wavelength range.

### 4.1 Overview of Common Biomarkers

Biomarkers are defined as measurable indicators of biological, physiological, or pathological states. They can be molecules, physical properties, appearance, or form. In the context of HSI, the spectra and appearance of the macromolecules in the human body are potential indications of diseases. Major macromolecules of the human body include lipids, carbohydrates, proteins, and nucleic acids. So far, clinical research only focused on the spectra of specific molecules. This section discusses known spectra of clinically relevant molecules and research to translate them toward understanding diseases. Table 7 lists common biomarkers and their working wavelengths.

**Table 7:** List of common wavelengths and relevant biomarkers.

Wavelength (nm)	Significance
400 to 700	Visible light wavelengths
400 to 1000	Operating range for silicon-based detectors
423	Absorption peak of oxidized CCO
430	Chlorophyll absorption peak
420 to 440	Absorption peak of S cone (“blue” cone)
450	Absorption peak of reduced CCO
540	Absorption peak of ${\mathrm{HbO}}_{2}$
543	Absorption peak of HHb
534 to 555	Absorption peak of M cone (“green” cone)
577	Absorption peak of ${\mathrm{HbO}}_{2}$
564 to 580	Absorption peak of L cone (“red” cone)
590	Isosbestic point of hemoglobin
598	Absorption peak of oxidized and reduced CCO
662	Absorption peak of chlorophyll
**700 to 10,000**	**Infrared wavelengths**
808	Isosbestic point of hemoglobin
970	Minor absorption peak of distilled water
1000 to 1800	Operating range for InGaAs detectors
1040	Minor absorption peak of lipid^514^
1200	Major absorption peak of lipid^519^ and collagen^514^
1400	Major absorption peak of lipid^514^
1450	Major absorption peak of distilled water^515^
1500	Major absorption peak of collagen^514^
1700	Major absorption peak of lipid^514^
1950	Major absorption peak of distilled water^515^
2500	Major absorption peak of distilled water^515^

**Fig. 11:**
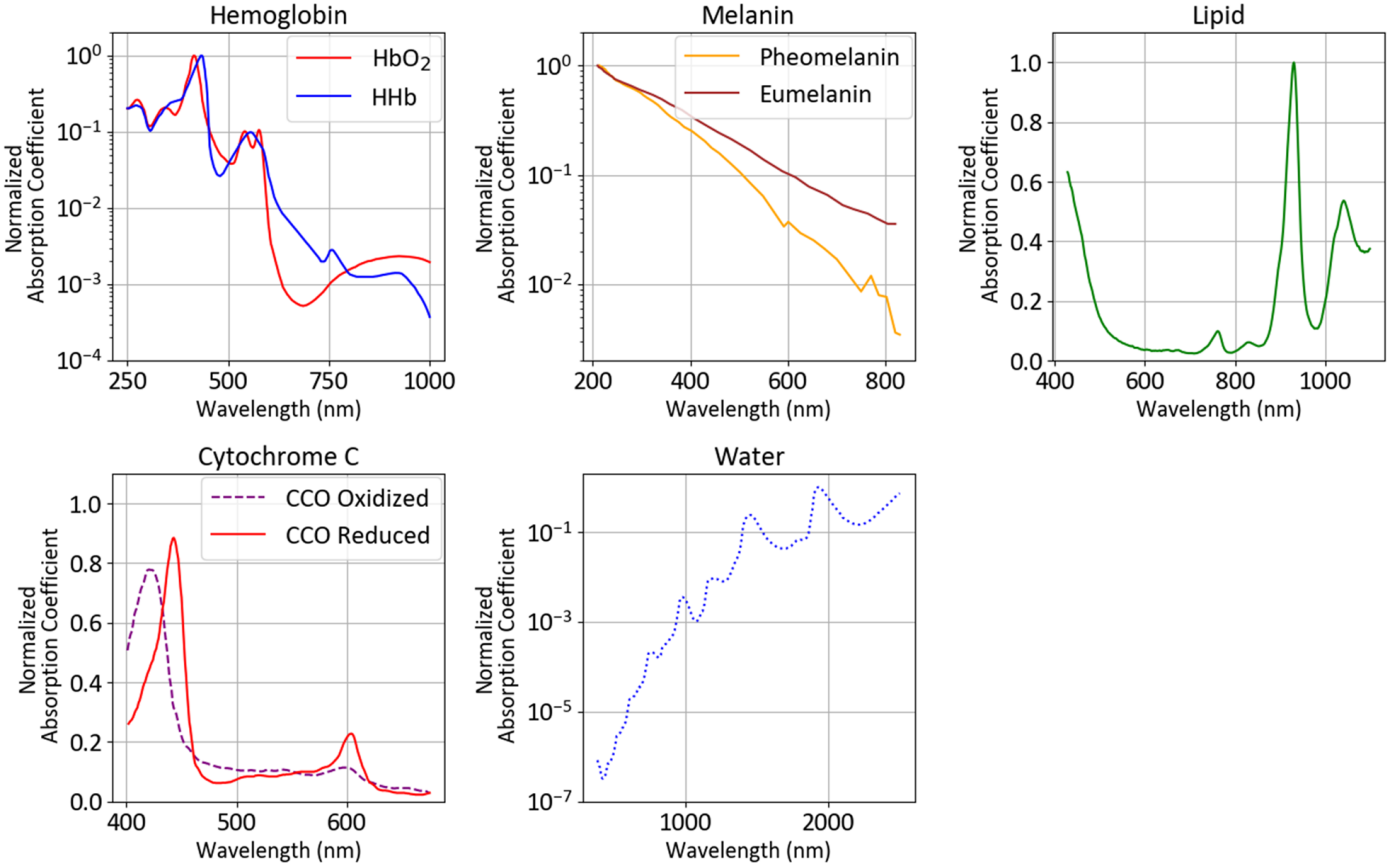
Normalized absorption spectra of common biomarkers. Figure generated from data compiled by Prahl et al.^516^ (hemoglobin), Sarna and Swartz^517^ (pheomelanin and eumelanin), van Veen et al.^518^ (lipid), Mason et al.^519^ (cytochrome-c-oxidase), and Pope et al.^520^ and Kou et al.^521^ (water).

The optical properties of blood and water serve as the basis for many clinical applications. Blood is the principal fluid that transports oxygen, nutrients, and waste across the body. Water is accountable for 75% of tissue mass and is often the major source of optical absorption. Therefore, many HSI applications rely on the absorption spectra of blood and water. The optical property of blood is dominated by hemoglobin in the visible spectrum and by water in the infrared spectrum. Removing blood cells from blood produces plasma, which is optically dominated by albumin and water. Bosschaart et al.^368^ compiled a literature review of the optical properties of blood from seven different studies. Figure 11 shows the normalized absorption coefficient of different biomarkers.

#### 4.1.1 Hemoglobin

Hemoglobin (also spelled haemoglobin) is a protein that transports oxygen in red blood cells. In healthy human adults, approximately 45% of blood volume is made up of red blood cells.^368^ They are the major spectral absorbing element of blood, being 100 to 1000 times more absorbent than water or other proteins in the visible light range. Therefore, the optical properties of hemoglobin provide insight into the health of blood. Hemoglobin can become saturated with oxygen molecules, which become oxyhemoglobin, or desaturated with oxygen molecules (no molecules are bound), which become deoxyhemoglobin. There are other configurations of hemoglobin molecules such as: carboxyhemoglobin (COHb, when carbon monoxide binds to hemoglobin), glycated hemoglobin (A1C, bonded to glucose), and methemoglobin (hemoglobin that is oxidized and cannot carry oxygen).^522^ The spectral signatures of ${\mathrm{HbO}}_{2}$ and HHb are by far the most researched and understood. The first to study the absorption spectra of ${\mathrm{HbO}}_{2}$ was likely Felix Hoppe-Seyler, who published a study in 1872 and noted two major absorption peaks at around 410 and 540 nm.^523^ The methods used to produce and measure spectra of hemoglobin are outside the scope of this review. Within visible light range, ${\mathrm{HbO}}_{2}$ has three major absorption peaks: the strongest is at 410 nm, the second at 540 nm, and the third at 580 nm. Deoxyhemoglobin has two major absorption peaks: the strongest is at 430 nm, and the second is at 550 nm.^368^ The differences between HHb and ${\mathrm{HbO}}_{2}$ become most apparent at the wavelengths from 600 to 1000 nm, with HHb having a higher absorption from 600 to 800 nm and ${\mathrm{HbO}}_{2}$ having a higher absorption from 800 to 1000 nm.

The concentration of HHb and ${\mathrm{HbO}}_{2}$ is used to calculate oxygen saturation in blood. If we let the molar concentration of HHb in blood be [HHb] and the concentration of ${\mathrm{HbO}}_{2}$ in blood to be $\left[{\mathrm{HbO}}_{2}\right]$, then the oxygen saturation of blood is defined as^148^:

$$\%{\mathrm{SO}}_{2}=\frac{\left[{\mathrm{HbO}}_{2}\right]}{\left[{\mathrm{HbO}}_{2}]+[\mathrm{HHb}\right]}\times 100\%.$$

In healthy adults, oxygen saturation should range from 95% to 100%. In practice, the oxygenation rate is often estimated using the spectra of HHb and ${\mathrm{HbO}}_{2}$. The simplest method is to measure the absorbance at two separate wavelengths. This is called two-wavelength oximetry.^148^ Many oximetry devices use two LEDs, one at 660 nm and one at 940 nm, to measure this. However, this method may not be entirely accurate. First, oximetry devices are often not reliable for saturation rates of less than 70%. Second, many other types of hemoglobin, such as COHb and methemoglobin, do contribute to the absorption of blood. Manufacturers calibrate this assumption into their devices; however, in some adults, such as smokers who may have elevated COHb concentration, the readings from pulse oximetry might not be entirely accurate.^524^

#### 4.1.2 Cytochrome-*c*-oxidase

Cytochrome-c-oxidase (also called CCO, Cyt-ox, or complex IV) is a transmembrane protein that plays an important role in oxygen respiration. It is the fourth and last enzyme in the electron transport chain. It converts hydrogen ions, $H^{+}$, and dioxygen, $O_{2}$, into water and generates a hydrogen ion gradient to pump $H^{+}$ ions out of the mitochondrial matrix. The electron transport chain accounts for more than 90% of the cellular free energy generated from oxygen respiration.^525^ Therefore, the oxidation state of CCO infers important information about tissue metabolism; the absence of oxygen in cells resulted in the complete reduction of CCO.^525^ Oxidized CCO has two absorption peaks in the visible spectrum at around 423 and 598 nm and an absorption band in the infrared spectrum of 820 to 840 nm.^519^^,^^525^ Reduced CCO has two absorption peaks in the visible spectrum at around 450 and 600 nm.^519^ In the visible spectrum, the absorption of tissue is dominated by hemoglobin, whereas in the high IR wavelength above 1300 nm, the absorption of tissue is dominated by water. Therefore, spectral observation of CCO is performed mostly in the near-infrared wavelength from 700 to 1300 nm.^525^

The concentration of oxidized CCO is a biosignature for ATP production, cell metabolism, cell apoptosis, and oxidative stress.^4^^,^^526^ Because the total concentration of CCO does not change in tissue, it is more applicable to measure the changes from oxidized to reduced stages of CCO and therefore the difference spectra between oxidized and reduced CCO. However, spectral observation of CCO is mostly performed using spectroscopy systems^379^^,^^527^^–^^529^ due to the lack of HSI instruments that operate in the NIR regions. Prior to 2014, CCOs were used as a signaling molecule in monitoring age-related macular degeneration (AMD).^530^ In recent years, the most notable contribution to CCO monitoring using the HSI system was from Giannoni et al.^65^^,^^302^^,^^531^ who used an HSI camera to image the cortex. Giannoni et al.^531^ used a commercial snapshot system that images from 445 to 998 nm wavelengths. They used the system to observe relative changes of HHb, ${\mathrm{HbO}}_{2}$, and oxidized CCO in mice cortex as the mice were subjected to normal, hypoxia, and hyperoxia conditions. The study was notable for using specific wavelengths (778 to 902 nm) to estimate the variation in the concentration of oxidized CCO. However, the authors found that the signal of CCO was too small and localized to be captured accurately using the NIR camera. Giannoni et al.^302^ later presented a Monte Carlo simulation framework for the hemodynamics of the cerebral cortex. They^65^ also developed a hyperspectral imaging system in the NIR range, imaging 11 bands from 600 to 894 nm. They were able to map the changes of oxidized CCO in the exposed mice cortex throughout different stages of oxygen deprivation. The authors were able to resolve low-resolution problems from their previous publication and achieved a spatial resolution of 0.42  μm/pixel.

Outside of brain hemodynamics applications, Bjorgan et al.^532^ used a push-broom HSI system (400 to 1000 nm) to image epithelial wounds. The authors found that the wound spectra have an absorption peak at 561 nm on day 1 and day 22 of healing. One theory the authors proposed was that this was related to the concentration of oxidized CCO.

#### 4.1.3 Lipid

Lipid is an important macromolecule in the human body. Studies by Van Veen et al.^533^ in the VIS-NIR range and Nachabé et al.^534^ in the NIR range are notable works in measuring the absorption spectra of mammal fat. In the VIS-NIR range, lipid has absorption peaks at around 750 and 920 nm.^533^ In the NIR to SWIR range, lipid has absorption peaks at 1050, 1200, 1400, and 1700 nm.^386^ Because of the recognizable peaks and the high penetration depth of SWIR light, SWIR is the preferred option to estimate lipid concentration in tissue.^386^ The concentration of fatty tissue and lipid in the organs is an indication of diabetes and fatty liver diseases. Okubo et al.^386^ used a NIR HSI system (1000 to 1600 nm) to scan images of mouse liver samples. The images were then analyzed to predict the concentration of lipids in the liver. They found that the concentration of lipids in the liver is highly correlated with the normalized absorbance at around 1200 and 1400 nm. Furthermore, support vector regression was able to predict fat concentration in the liver with a mean absolute error of 10.9  mg/g (an 8.5% error).

#### 4.1.4 Melanin

Melanin is a group of molecules that provide biological pigments. In humans, melanin is produced by specialized cells called melanocytes. Upon exposure to UV light, melanocytes produce melanin and make the skin darker. Melanin has strong absorbance and scattering properties^252^ and is one of the main chromophores of the human skin, alongside hemoglobin. Its primary location in the epidermal layer^53^^,^^123^^,^^535^ makes it particularly relevant to optical studies, including HSI, where precise spectral information is required for the analysis of tissue composition.

In the wavelength range 400 to 700 nm, melanin exhibits logarithmic scattering and absorption properties.^320^^,^^536^ The equation for melanin absorption was modelled by Bjorgan et al.^301^ to be $\mu =\mu_{694}(\frac{\lambda }{694}{)}^{-3.46}$. Here, $\mu_{694}$ is the mean melanin content of the layer, which ranges from 200 to $300\;m^{-1}$ (lighter skin) to 500 to $900\;m^{-1}$ (darker skin). This can be used to calculate the contribution of melanin in skin absorption spectra and subsequently remove the contribution of melanin or quantify its concentration.^30^^,^^267^^,^^537^ This can be necessary in some cases as melanin can affect the estimation of oxygen through the skin.^538^ In some cases, it is necessary to estimate the concentration of melanin. Melanoma is a deadly type of cancer caused by the proliferation of melanocytes. The size of the melanoma tumor correlates with the density of blood (hemoglobin) and concentration of melanin.^326^ To differentiate between melanoma and benign skin tumors, methods to estimate melanin concentration can be used (see Sec. 4.2.4 for more detailed discussions). Melanin is also present in the retina^33^^,^^86^^,^^381^; however, its biomarker properties are not well explored. Fan et al.^461^ noted that hydroxychloroquine binds to melanin within the retina and used this fact to detect hydroxychloroquine retinopathy.

#### 4.1.5 Amyloid beta

Amyloid beta is a naturally occurring protein in human metabolism. However, the accumulation of amyloid beta into plaques in the central nervous system (CNS) is a hallmark of many neurodegenerative diseases, most notably Alzheimer’s disease (AD).^139^ Amyloid beta depositions are seen in the retina of both humans and mice with AD.^88^^,^^139^^,^^381^ This makes HSI a viable option for the detection of excess amyloid beta because the retina can be easily accessible through the pupil without invasive procedures. Amyloid beta plaques have wavelength-dependent (Rayleigh) scattering that is most distinguishable from the regular retina in the range 400 to 550 nm.^60^^,^^75^^,^^86^^,^^139^^,^^381^ As such, many researchers use the wavelengths 400 to 550 nm to distinguish between the retina of individuals with AD and the retina of healthy controls. The differences are very significant: More et al.^381^ found that the optical differences in spectral reflectance were not affected by some mild form of cataract or glaucoma. However, it is not known whether the accumulation of amyloid in the retina correlates with cognitive decline because the difference is more sensitive in the early stage of the disease.^381^ However, studies on mice showed increased scattering as the disease progresses.^88^ Some researchers also used the spectral properties of amyloid beta to identify them *ex vivo* without the use of stains. Du et al.^60^ trained a GAN to virtually stain retinal cells without the use of DAB stains.

#### 4.1.6 Nanoparticles

Nanoparticles play a vital role in biomedical imaging due to their unique optical properties and nanoscale size. Their characteristics, such as plasmonic resonance in gold and silver nanoparticles and fluorescence in quantum dots, make them effective contrast agents. Gold and silver nanoparticles scatter and absorb light in the visible to near-infrared spectrum, enhancing imaging techniques such as darkfield microscopy and hyperspectral imaging.^223^ Quantum dots, with their tunable emission spectra, enable the simultaneous detection of multiple biomarkers.^229^ These optical features, combined with the ability to functionalize nanoparticles for specific targets, make them powerful tools in advancing precision imaging and diagnostics. Coatings for nanoparticles are essential to improve their stability, prevent clumping, and enhance their targeting capabilities in biological environments. Common coatings include polyethylene glycol (PEG), antibodies such as bovine serum albumin (BSA), and citrates.^223^ In recent years, darkfield hyperspectral imaging has become a prominent method for detecting nanoparticles in biological tissues (see Secs. 2.5 and 4.6.9). By referencing known spectral libraries, researchers can identify the presence of multiple nanoparticles and their composites.^229^

Gold nanoparticles (AuNPs) are widely used as contrast agents and therapeutic tools.^539^ Jenkins et al.^539^ identified the spectra of plasmonic gold nanoparticles in blood, showing that their optical properties depend on coatings and particle agglomeration. For example, citrate-coated AuNPs have a peak at 537 nm, whereas BSA-coated AuNPs exhibit a peak at 539 nm (primary), a single peak at 541 nm (small agglomerates), or two peaks at 545 and 780 nm (large agglomerates). Silver nanoparticles (AgNPs) have antimicrobial properties, making them useful for monitoring infections or delivering drugs.^229^ Jenkins et al.^539^ observed scattering peaks around 500 nm for primary AgNPs and 600 to 700 nm for agglomerated AgNPs. Similarly, Ray et al.^222^ observed broad peaks of AgNP clusters at 550 and 570 nm.

### 4.2 Hyperspectral Imaging for Cancer Detection

It was estimated that there were 2 million new cancer cases and over 0.6 million cancer deaths in the United States in 2024.^540^ Surgical resection has long been the major and standard treatment approach for many types of cancers. During surgery, all tumor cells are desired to be removed, whereas healthy tissue should be preserved for normal organ function. However, visual observation and palpation, even intraoperative pathological consultation, are subject to errors. HSI has been investigated for *in vivo* and *ex vivo* detection of various cancers. The premise for HSI to do cancer detection lies in the change of tissue optical properties caused by the phenomena related to the presence, progression, or regression of neoplasia. Such biomarkers include metabolic rates, oxygenation, blood flow, spatial and structural characteristics of tissue architecture, biochemical pathways, and cell viability.^541^ This section focuses on how HSI has been used for the detection of various cancers.

#### 4.2.1 Brain tumor

Brain tumors pose a critical clinical challenge due to their location in the CNS, where even small tumors can lead to severe, life-altering neurological deficits. Malignant brain tumors such as glioblastoma (GBM) have among the worst prognoses of all brain cancers, with an average survival of ∼1.5 years after treatment.^542^ Moreover, there are multiple factors that make brain tumor detection challenging. First, brain tissue is highly functional and has limited regenerative capability^543^; thus, functional preservation is as critical as survival, and it is not feasible to guarantee tumor removal by “taking extra margins.” As such, brain surgeries require highly precise tumor boundary identification. Meanwhile, brain tumors, especially gliomas, are diffusely infiltrative,^164^ meaning tumor cells often extend beyond what is visible to the surgeon’s eye.^95^ In addition, brain biopsy carries a high risk of complications,^344^ making noninvasive optical imaging techniques more valuable. Furthermore, the blood-brain barrier limits the delivery of most imaging contrast agents or fluorescence dyes,^544^ which brings up the need for label-free alternatives. HSI has emerged as a powerful tool for brain tumor detection, offering a noninvasive and label-free method to capture rich spectral and spatial information during neurosurgical procedures. By acquiring data across hundreds of contiguous spectral bands, HSI enables pixel-level differentiation of tumor and healthy brain tissues based on their distinct spectral signatures, which is not easily achievable with bare human eyes or traditional imaging modalities.

The HypErspectraL Imaging Cancer Detection (HELICoiD) project in Europe carried out extensive studies on *in vivo* brain cancer detection.^126^^,^^128^^,^^129^^,^^131^^,^^343^^,^^425^ An intraoperative visualization system based on two push-broom hyperspectral cameras (VIS-NIR and NIR) was built, covering a total spectral range of 400 to 1700 nm and capable of fast acquisition and processing of hyperspectral images during neurosurgical operations.^126^^,^^128^ However, the misalignment and different fields of view of the two cameras made it difficult to utilize the full wavelength range. To address this limitation, Leon et al.^425^ implemented a VIS-NIR and NIR fusion with spatial and spectral registration in 2021. The fused hypercube had 785 bands in a spectral range of 435 to 1638 nm.

Based on the dataset acquired with their system, various cancer detection algorithms were developed. Ravi et al.^126^ proposed a two-step pipeline, including dimension reduction based on the t-distributed stochastic neighbor approach and tissue classification using semantic texton forest. The method was tested on two patients with a mean-class accuracy of 81.9% and 71.5%, respectively. Fabelo et al.^128^^,^^129^ employed a two-branch hybrid algorithm. A spatial–spectral homogenization map and an unsupervised segmentation map were generated in two branches and fused to pass a majority voting, which created three maximum density maps for intraoperative visualization of the primary brain tumor, normal tissue, and background. Later, Fabelo et al.^343^ proposed a deep learning–based multistep framework, which generated a blood vessel segmentation mask, a parenchymal segmentation mask, and a four-class classification map, and combined them for the final pixel-level classification map. The method was evaluated on their *in vivo* brain tumor dataset that included 26 VIS-NIR hypercubes from 16 different patients, with a total number of 258,810 labeled spectral signatures. The deep learning pipeline achieved an overall accuracy of 80% for multiclass classification. To provide faster intraoperative decision support, Campos-Delgado et al.^342^^,^^351^ used extended blind endmember and abundance extraction as a computational-efficient image classification method, and Florimbi et al.^131^ achieved a near-real-time intraoperative cancer detection with less than 3 s image classification time by exploiting a multi-GPU parallel computing system.

Furthermore, the HELICoiD group has researched several approaches on optimal band selection,^130^^,^^137^^,^^457^^,^^545^^,^^546^ to reduce data dimensionality and achieve higher data efficiency. Manni et al.^457^ applied an ant colony optimization algorithm to select a subset of 100 most useful bands and then used a deep spectral-spatial approach based on a 3D-2D hybrid CNN architecture to implement four-class classification. The model achieved an average accuracy of 80%, which was 8% higher than a traditional 2D CNN. Despite the hybrid network having a relatively low (76%) sensitivity for tumor tissue, it had a very good sensitivity (96%) for blood vessels. In addition, Martinez et al.^545^ investigated the most relevant spectral bands for brain cancer detection. They first used a sampling interval of 3.61 nm to evenly extract 128 bands from each of the original 826-band hypercubes, then tested several optimization algorithms to select the most useful 48 bands and implemented SVM classification. The results show that classification accuracy was improved 5% using the 48 selected bands compared with that using 128 bands, with a 30% increased median tumor sensitivity. Baig et al.^546^ further investigated the optimal band selection using empirical mode decomposition. By balancing the overall accuracy and number of features, they selected seven optimal bands and achieved an overall testing accuracy of 73.36%, which was comparable to that of the 48-band benchmark data. Based on a further expanded dataset,^130^^,^^137^ an absorbance peak around 760 nm related to HHb in tumor tissues was noticed regardless of grade and origin. They also reported the spectral bands with statistical differences among different types of tissues and tumor grades.^137^ The comparison of classification results using spectral and spatial–spectral methods also revealed the usefulness of both spectral and spatial information in hypercubes.

**Fig. 12:**
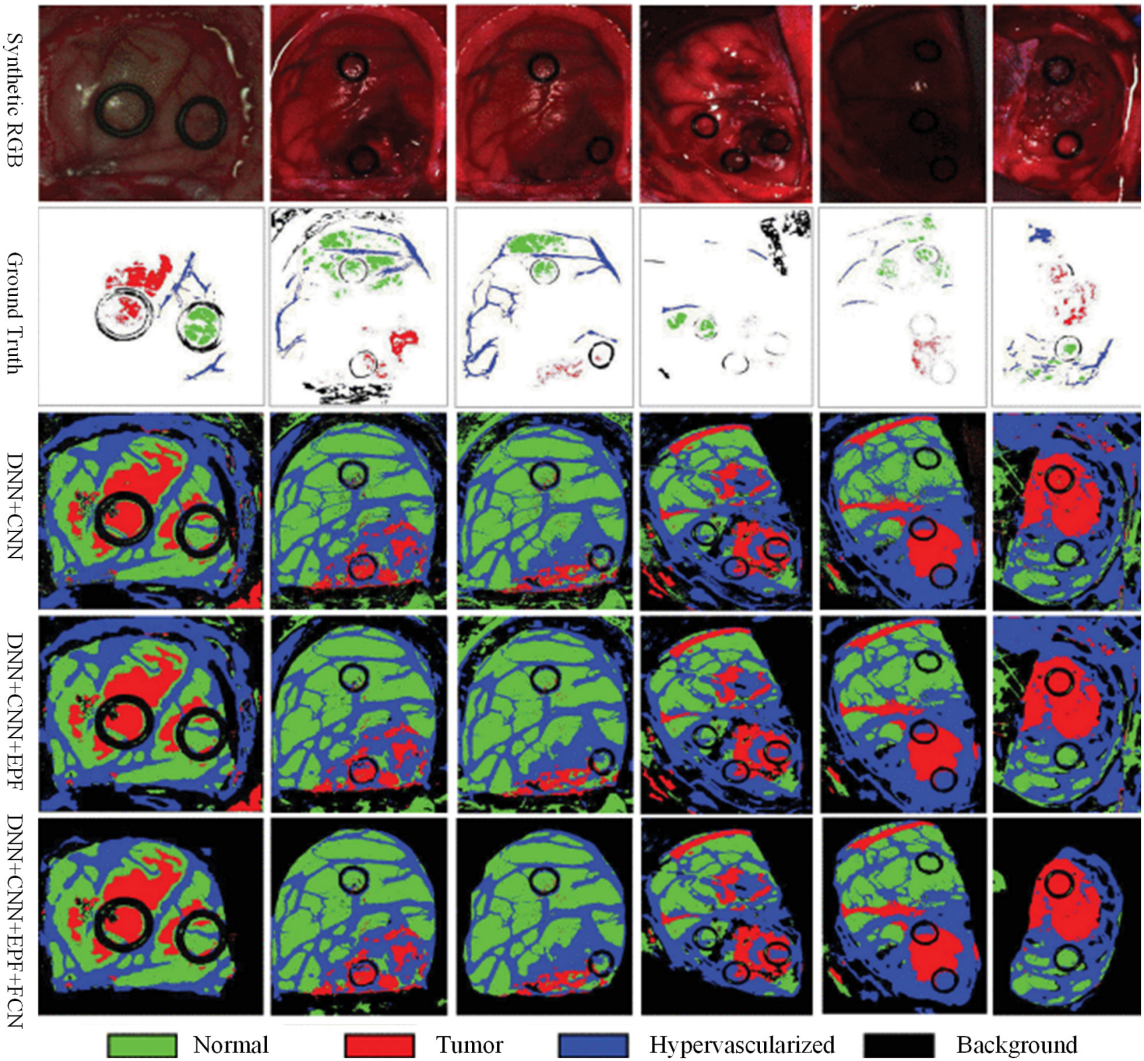
Classification maps yield by the DNN+CNN, DNN+CNN+EPF, and DNN+CNN+EPF+FCN methods on the test hypercubes from *in vivo* human brain HSI datasets. EPF, edge-preserving filter. The figure was reproduced with permission from Hao et al.^471^

In addition to the HELICoiD project, several research groups around the world also employed HSI for brain cancer detection. Hao et al.^471^ proposed a hyperspectral image classification method based on the fusion of an FCN, a 1D DNN, a 2D CNN, and an edge-preserving filter-based method for *in vivo* human GBM detection, as shown in Fig. 12. Urbanos et al.^164^ compared three machine learning methods, namely, SVM, RF, and CNN, for the five-class classification of *in vivo* high-grade gliomas hyperspectral images. Based on a dataset of 13 images and a double-cross-validation training strategy, SVM achieved the highest average overall accuracy of 60.1%. However, in this study, despite the three classifiers resulting in a relatively high accuracy for the venous blood, arterial blood, and dura mater classes, they all had fairly low accuracy and sensitivity for tumor tissues. Rinesh et al.^408^ employed a series of processes, including Frost filtering, firefly optimization, and k-means clustering, for brain tumor detection in hyperspectral images. The proposed method outperformed traditional machine learning classifiers such as kNN, DNN, and SVM with 96.47% accuracy, 96.32% sensitivity, and 98.24% specificity. Giannantonio et al.^95^ employed a custom-made hyperspectral surgical microscope system for intraoperative brain tumor detection. They acquired a dataset of 18 images from five patients, which were then cropped into over 8000 tiles. RF, SVM, and an MLP model with one hidden layer were used for classification between tumor and normal tissues, where the MLP achieved the highest accuracy of 92%. They also found that spectral bands in 650 to 780 nm have more importance for the classification. Puustinen et al.^110^ employed a surgical microscope equipped with a VIS-NIR snapshot hyperspectral camera for intraoperative brain cancer detection. They tested nine classification algorithms, among which the light gradient boosting machine decision tree achieved the best performance of 98.3% overall accuracy and 97.7% glioma accuracy. Wang et al.^547^ proposed a deep margin cosine autoencoder for hyperspectral image classification and tested it for different applications, including *in vivo* brain cancer detection. Based on the spectra extracted from images of two patients, the proposed network achieved 99.97% and 99.8% correct identification rates on both patients. However, both studies^110^^,^^547^ had limited data and therefore used spectra of different pixels of the same patients for model training and testing.

Last, HSI has been used to enhance fluorescence visualization for the detection of brain tumors. Wirth et al.^63^ employed HSI and spectral unmixing for whole-body fluorescence cryo-imaging and tested the proposed method on a glioma xenograft mouse model. A white light image as well as images of three different fluorophores could be obtained, each of which displays a distinct biodistribution behavior. They claim that using HSI and spectral unmixing significantly changed the interpretation of the agent distribution by increasing the tumor-to-background contrast in different fluorescence channels and avoiding the contamination of autofluorescence. To characterize the limits of protoporphyrin IX (PpIX) fluorescence for the detection of gliomas, Lehtonen et al.^542^ employed *in vitro* HSI on 16 PpIX concentration samples and eight control samples. HSI resulted in an average recognition rate of 96% and sensitivity of 100% compared with those of three expert neurosurgeons (63% and 48%, respectively). Note that although the snapshot HSI camera had a wide spectral range of 501 to 903 nm, this study only used four spectral bands in the visible range, targeting red, green, blue, and the fluorescence peak. Finally, Walke et al.^62^ evaluated the visualization of PpIX using a wide-field HSI system on 240 biopsies from 26 patients and compared it with a regular surgical microscope fluorescence module. It was found that HSI gave a higher sensitivity of fluorescence signals from the tumor.

#### 4.2.2 Head and neck cancer

Head and neck cancers (HNCs) are among the most common cancers worldwide.^73^ They can be diverse due to the anatomical diversity of this region. In the oral cavity and upper aerodigestive tract, ∼90% of cancers are squamous cell carcinoma (SCC),^201^ which predominantly originate from the squamous cell in the nasopharynx, oral cavity, oropharynx, nasal cavity, paranasal sinuses, hypopharynx, larynx, and trachea,^548^ whereas others include carcinomas from the thyroid and salivary gland.^458^ Efficient screening is important to lower incidence, whereas early detection and surgical treatment are all critical to improve survival rates. HSI provides more detailed information compared with visual inspection and palpation as well as faster diagnosis compared to biopsy.^478^ Therefore, it has gained much attention for head and neck cancer detection.

In the past decade, the Fei group^185^^,^^312^^,^^345^^,^^394^^,^^406^^,^^407^^,^^412^^–^^416^^,^^428^^,^^430^^,^^458^^,^^463^^,^^507^^,^^549^^–^^553^ has performed extensive research on hyperspectral head and neck cancer detection, especially focusing on head and neck SCC and thyroid cancer. The HSI technology was first validated in mouse models, and various types of features were investigated for effective cancer detection. Lu et al.^312^^,^^430^ proposed using tensor representation as the spectral-spatial feature for *in vivo* noninvasive head and neck SCC detection, which outperformed traditional spectral classification with 93.7% sensitivity and 91.3% specificity in an HNC xenograft mouse model. Based on the same *in vivo* mouse model, Ma et al.^549^ proposed a feature extraction method using discrete wavelet transformation. The wavelet-based features resulted in a comparable classification performance with raw spectral signatures but lowered the computation time by half. Pike et al.^550^ implemented automatic band selection based on mutual information between spectral bands and used a minimum spanning forest for classification. The results demonstrated that using selected bands, mostly focused in 800 to 870 nm range, improved cancer detection accuracy compared with using full bands. Chung et al.^507^ proposed using superpixels together with PCA and SVM, to utilize both spatial and spectral information. Using two principal components, they obtained 93% sensitivity and 85% specificity in 11 mice. Ma et al.^551^ investigated deep learning by utilizing a simple CNN, followed by another work^407^ using an adaptive deep learning method, which achieved 92.32% sensitivity and 91.31% specificity in 12 mice.^407^ In addition, Lu et al.^428^^,^^552^ further tested intraoperative hyperspectral HNC detection after tumors were exposed. Not only did they find that using all spectral bands in the 450 to 900 nm range gave the best sensitivity and specificity compared to using partial bands,^552^ but they also developed an intraoperative HSI cancer detection framework, including a pipeline of image calibration, band registration, glare removal, curvature correction, and feature extraction.^428^ Later, they focused on oral carcinogenesis and investigated *in vivo* cancer detection in a chemical-induced mouse tongue cancer model, where they achieved the best classification AUC of 89.2% between tumor and normal tissue using RF.^406^ In addition, in an *ex vivo* mouse tongue investigation, a 0.86 AUC and 79% accuracy were achieved using both linear SVM and ensemble LDA.^415^

**Fig. 13:**
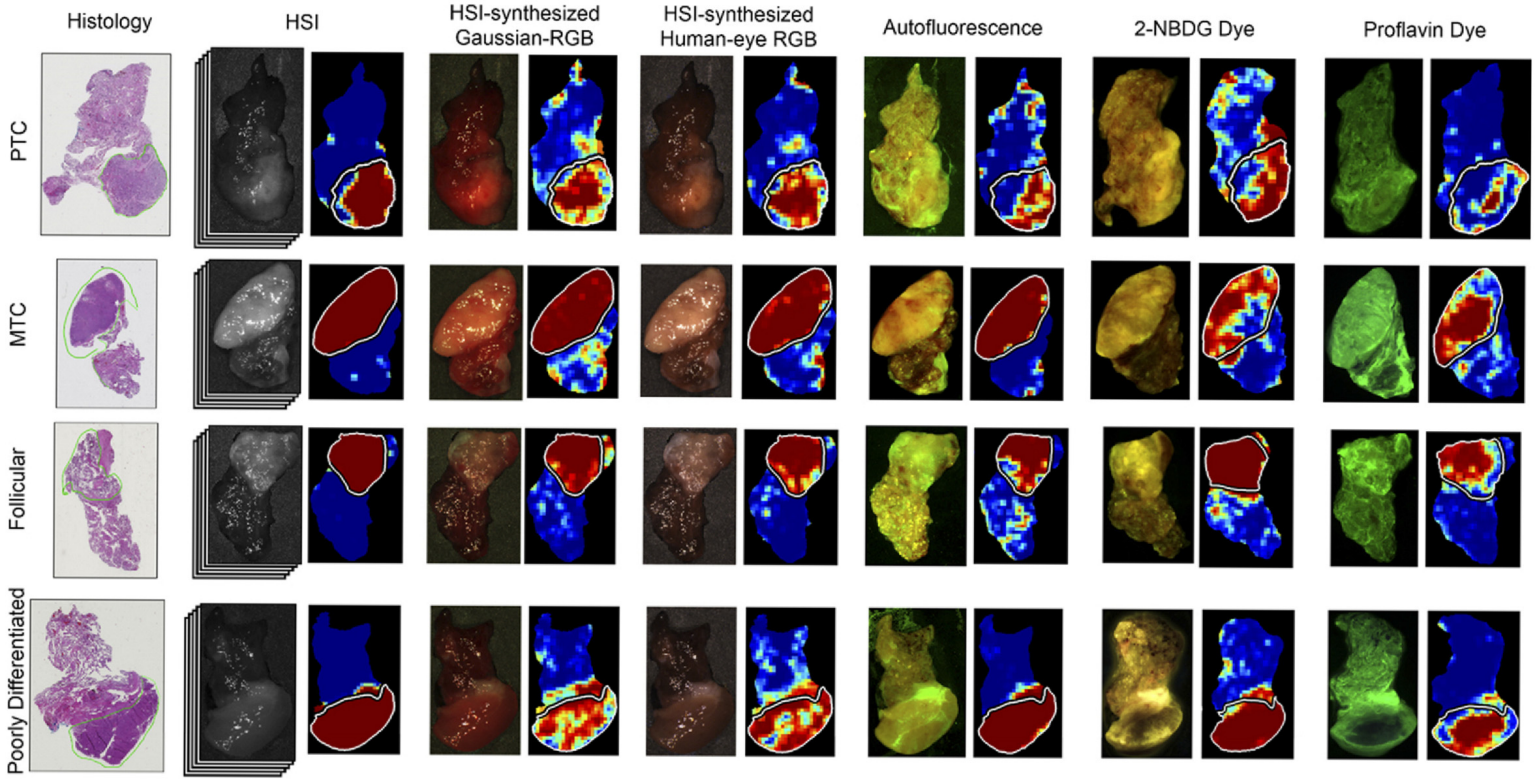
Representative tissue images and corresponding classification heat-maps from all modalities from patients with thyroid carcinoma. Columns from left to right: histology, HSI with heat-map, HSI-synthesized Gaussian-RGB multiplex with heat-map, HSI-synthesized human-eye RGB multiplex with heat-map, autofluorescence with heat-map, 2-NBDG dye image with heat-map, Proflavine dye image with heat-map. Rows from top to bottom: papillary thyroid carcinoma (PTC), medullary thyroid carcinoma (MTC), follicular thyroid carcinoma, and poorly differentiated thyroid carcinoma. The contours in white (in heat-maps) and green (on histology) outline the cancerous regions. Predicted tumor heat-maps range from dark blue (predicted normal) to dark red (predicted cancer).^458^

After a series of preliminary studies in animal models, the Fei group moved on to *ex vivo* human tissue samples.^553^ Based on a dataset of surgical tissue specimens collected from 16 patients, they achieved an average classification accuracy of 90% for oral cancer and 94% accuracy for thyroid cancer using an ensemble LDA classifier.^412^^,^^554^ Then, Lu et al.^413^ further used LDA classifiers to distinguish tumor and normal tissue using spectral signatures extracted from 36 patients (20 with SCC and 11 with thyroid cancer). For the inter-patient experiment, they implemented leave-one-patient-out cross-validation and compared HSI with autofluorescence, proflavine, and 2-deoxy-2-[(7-nitro-2,1,3-benzoxadiazol-4-yl)amino]-d-glucose (2-NBDG). The results showed that HSI outperformed autofluorescence, 2-NBDG fluorescence, or proflavine fluorescence imaging with a sensitivity higher than 90% for various types of tissues in the dataset. Following that, Halicek et al.^394^^,^^463^ expanded the *ex vivo* HNC dataset and explored various CNNs for the detection of SCC and thyroid cancer separately. Eventually, for SCC, they accumulated a dataset of 293 surgical specimens from 102 patients^416^ and used an inception-based neural network for classification.^414^^,^^416^ In the intra-patient experiment, HSI outperformed proflavine and autofluorescence for the detection of conventional SCC with an AUC of 0.82 up to 3 mm from the cancer margin, whereas autofluorescence imaging detected the human papilloma virus positive (HPV+) SCC better. In the inter-patient experiment, HSI detected conventional SCC better in the larynx, oropharynx, and nasal cavity with a 0.85 to 0.95 AUC score, and autofluorescence did better for HPV+ SCC in tonsil with 0.91 AUC.^416^ Meanwhile, for thyroid cancer, they collected 200 specimens from 76 patients and implemented a CNN modified from Inception-v4 network for cancer detection.^458^ HSI, HSI-synthetic RGB, autofluorescence imaging, and fluorescence imaging with two different dyes were compared. Interestingly, HSI-synthetic RGB achieved the best performance with an AUC score of 0.9, whereas HSI and autofluorescence still outperformed other dyes,^458^ as shown in Fig. 13. Furthermore, they found that specular glares could degrade the overall classification result, but incorporating a majority voting unsupervised segmentation could assist in reducing the effect of glare.^185^^,^^345^

HSI studies were also performed by other groups for HNC detection. Jeyaraj et al.^475^ utilized a partitioned deep CNN for oral cancer detection in *ex vivo* tissues and compared their method with conventional classification techniques such as SVM and deep belief network. Their proposed network outperformed conventional classifiers, achieving an accuracy of 94.5% with 98% specificity and 94% sensitivity. Brouwer de Koning et al.^555^ employed a network with four dense layers for tongue cancer detection in *ex vivo* human tissue samples. They first collected hyperspectral data from 14 patients who underwent tumor removal surgery. Then, the method was evaluated on three HSI datasets—the “visible” (400 to 1000 nm), “NIR” (950 to 1700 nm), and “combined” (450 to 1700 nm)—where the “visible” data gave the best classification results of 0.932 AUC, 82.4% accuracy, 83.6% sensitivity, and 80.3% specificity. Although using the “combined” data led to a better performance than using only the “NIR” data, it did not provide additional benefit to the “visible.” Meanwhile, Trajanovski et al.^366^ utilized HSI with deep learning semantic segmentation for tumor delineation in those *ex vivo* tongue SCC samples. They proposed a segmentation network that also learned to select the most useful bands, which achieved an average dice score of 0.891 and an AUC of 0.924. By looking into the band selected in both the VIS-NIR range, they claimed that the NIR spectrum is crucial for tumor capturing in some cases.

Several studies, particularly hyperspectral endoscopic imaging, were carried out for cancer detection in the throat and vocal box regions. Regeling et al.^71^ developed a pre-processing pipeline for laryngeal cancer detection using hyperspectral rigid endoscopy. The pipeline encompassed image registration based on normalized cross-correlation, noise removal using MNF, and specular reflection detection using the Marr–Hildreth algorithm. Subsequently, Regeling et al.^72^ developed a hyperspectral imaging flexible endoscopy system and tested it in two patients who underwent routine endoscopy for laryngeal cancer diagnosis. Eggert et al.^73^ employed a rigid hyperspectral endoscope system, which was developed by Gerstner et al.,^556^ and acquired an *in vivo* hyperspectral dataset of laryngeal, hypopharyngeal, and oropharyngeal mucosa from 98 patients undergoing examination. They compared cancer detection performances using a 2D Densenet on RGB images, a 2D Densenet on hyperspectral images, and a 3D Densenet on hyperspectral images. The results show that 3D Densenet with HSI got the best tissue classification accuracy of 0.813, as well as the best sensitivity, specificity, and AUC of 0.833, 0.792, and 0.872, respectively. By comparing the classification accuracy using 10, 14, 18, and 22 bands, they also found a positive dependency of classification on the number of spectral bands.

Finally, Chu et al.^127^ explored HSI to diagnose nasopharyngeal carcinoma, a rare type of HNC, from serum samples. They imaged sera from 100 healthy volunteers and 60 patients with nasopharyngeal carcinoma, selected characteristic spectral bands, and implemented SVM classification, where they achieved 99.15% accuracy, 98.79% sensitivity, and 99.36% specificity, respectively.

#### 4.2.3 Breast cancer

Accurate detection and diagnosis of breast cancer (BC) plays a crucial role in clinical pathology analysis and surgical margins assessment.^437^ Complete tumor removal during breast-conserving surgery remains challenging due to the lack of optimal intraoperative margin assessment techniques.^328^ HSI as an emerging imaging modality has been explored to improve BC detection and surgical guidance.

To evaluate the feasibility of BC analysis using HSI, a series of studies was completed on animal models. McCormack et al.^46^ coupled a spectral-scanning HSI system onto an inverted microscope to measure dynamic changes in tumor microvessel density as well as hemoglobin oxygen saturation (${\mathrm{sO}}_{2}$) in live BC xenograft mice after trastuzumab treatment. Despite both trastuzumab-sensitive and -resistant groups showing a decrease in ${\mathrm{sO}}_{2}$ when compared with controls, an increase in vessel density was revealed in trastuzumab-sensitive tumors, suggesting that longitudinal HSI could distinguish between the two types of tumors. Sahu et al.^429^ employed HSI and tactile imaging for the characterization of malignant and benign mammary tumors in canine models. Specifically, for HSI, they calculated a tissue optical index involving the content of hemoglobin, water, and lipid. Then, they fused the tactile and spectral data and implemented classification using machine learning algorithms. The results show that fusing data from two image modalities resulted in significantly improved sensitivity and specificity of 86% and 97%, respectively. Stewart et al.^45^ carried out *in vivo* tissue discrimination of tumors and nontumors in mice models with breast or lung tumors. They imaged six tumor-bearing mice along with six tumor-free mice in three ways, i.e., intact with fur, intact and shaved, and the flank region opened. For discrimination, a partial least squares discriminant analysis (PLS-DA) model was employed, which achieved an average performance of 100% sensitivity, 83.3% specificity, 88.9% accuracy, and 0.917 AUC.

To take advantage of the spectral information in HSI, researchers investigated effective spectral bands based on both animal tissues and human tissues. Shen et al.^80^ developed an open-source mobile HSI system with 36 different LEDs and tested it on various objects, including breast tumor-bearing mice. Extracted spectra revealed significant amplitude differences in the wavelength range of 400 to 600 nm. Aboughaleb et al.^444^ imaged *ex vivo* breast tissues that were resected from 10 patients in the wavelength range of 420 to 620 nm range, where they noticed a high variance between tumor and healthy tissues at 500 nm as well as the lowest variance at 620 nm. Later, Mahmoud and El-Sharkawy^437^ used HSI with laser-induced fluorescence to detect BC malignancy. A 450 nm laser was used to excite the fluorescence signal, which was captured by the hyperspectral camera and revealed significantly high intensity at 561 nm for tumor tissues. Furthermore, Zhang et al.^287^ evaluated the usefulness of near-infrared bands from 900 to 1700 nm for the detection of seven types of cancers, including breast cancer, lung cancer, kidney cancer, and cancers in the gastrointestinal (GI) tract. Classification results on all cancer types illustrated that using AI directly on NIR hypercubes outperformed conventional color images with or without X-ray.

Kho et al.^328^ employed a dual-camera push-broom HSI setup that covered the 400 to 1700 nm range and imaged fresh *ex vivo* tissue slices (2 mm thick) from 42 patients undergoing primary breast surgery. The authors first evaluated the usefulness of three wavelength ranges, i.e., VIS (450 to 950 nm), NIR (954 to 1650 nm), and VIS-NIR (450 to 1650 nm), with a supervised Fisher’s LDA spectral classifier. Then, they used a modified U-Net that incorporated the spectral and spatial information of tissues for classification. The results revealed that using the combination of VIS and NIR bands in the 450 to 1650 nm range achieves the best discrimination for tumor tissues, whereas all three wavelength ranges were comparable for healthy tissues. Regarding the classification, although LDA and U-Net had very similar performances on tumor and healthy tissues with certain histopathological labels, U-Net was significantly better for tissue discrimination over the entire tissue samples, especially for tumors. In another study, Kho et al.^290^ investigated the imaging depth of breast tissues and proposed to distinguish tumor and healthy tissues using the spectral slope at wavelength regions where the imaging depth in both tissues is equal (around 2 mm). Jong et al.^420^ acquired a dataset of breast lumpectomies in addition to the breast tissue slice dataset. They trained three neural networks, i.e., a spectral 1D CNN, 2 dual-channel spectral-spatial CNNs, and a 3D CNN with the breast tissue slice dataset, where the dual-channel CNN performed the best. A domain adaptation strategy was applied to the 1D CNN and the dual-channel CNN to transfer the knowledge learned from the breast tissue slices (source domain) to the breast lumpectomy data (target domain). The fine-tuned 1D CNN achieved an RMSE of 9%, whereas the other obtained an RMSE of 11%, which revealed the possibility of using HSI to support the surgeon intraoperatively in the decision of tissue removal. Furthermore, they accumulated a larger dataset of lumpectomy specimens from 189 patients and discriminated tissue patches within the resection margin of *ex vivo* breast lumpectomy specimens.^422^ Before training the classifier, they performed spectral unmixing to estimate the fractional abundance of four tissue types, i.e., carcinoma *in situ*, invasive carcinoma, connective tissue, and fat, for the spectrum of each pixel; the results of which were used to assign ground truth labels to pixels in image patches. With a kNN classifier, they carried out lumpectomy specimen discrimination with a 0.87 accuracy, 0.94 sensitivity, 0.85 specificity, and 0.92 AUC.

#### 4.2.4 Skin cancer

MM is known as one of the primary and aggressive skin cancers. Nonmelanoma skin cancers, most commonly SCC and basal cell carcinomas (BCC), are less invasive but can be up to 20 times more common than MM, especially among people with a lighter skin complexion.^133^^,^^557^ HSI has been used for tumor detection in skin lesions.^320^

**Fig. 14:**
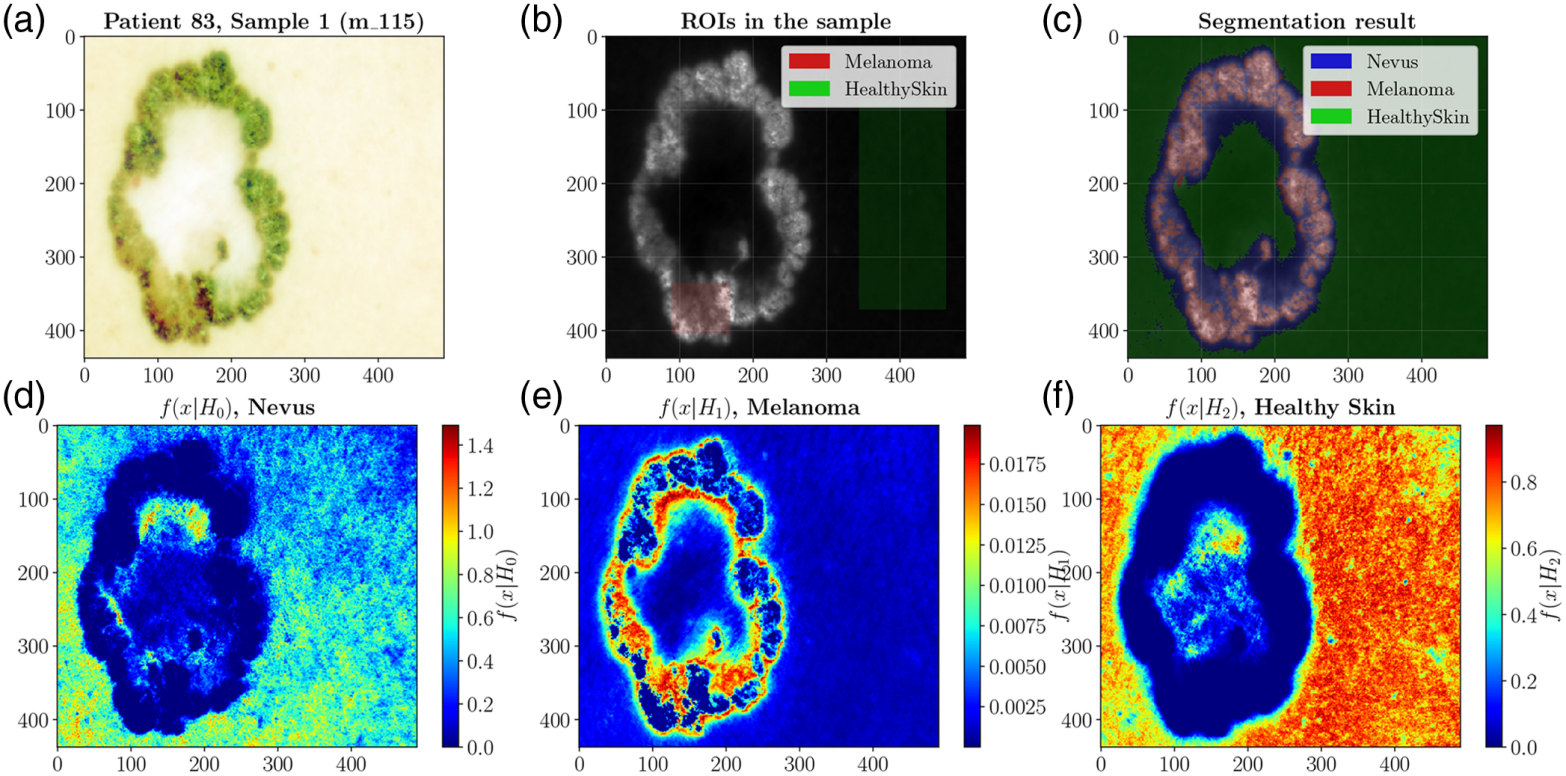
CIE 1931 RGB reconstruction of a melanoma sample (a), its in-lesion and out-of-lesion ROIs (b), and its final classification/segmentation output as a semitransparent overlay (c). False-color likelihood maps for the three hypotheses [(d) H0: nevus, (e) H1: melanoma, and (f) H2: healthy skin) for this sample, with their accompanying color bars.^326^

Nagaoka et al.^558^ imaged 24 MM and 108 non-MM lesions from 80 patients and 17 volunteers. Using the spectra from those hyperspectral images, the authors evaluated two discrimination indices, namely, the frequency distribution of the spectral angle and the entropy of the spectral angle. Later, Christensen et al.^559^ further evaluated the latter discrimination index specifically on Caucasian pigmented skin lesions (PSLs). Among 202 PSLs from 186 patients, they achieved 96.7% sensitivity, 42.1% specificity, and 0.8 AUC for the detection of cutaneous MM. Neittaanmäki et al.^152^^,^^560^ imaged *in vivo* lentigo maligna (LM) and lentigo maligna melanoma (LMM) lesions in the wavelength range of 500 to 900 nm and reported distinct contrast of these lesions in hyperspectral abundance maps. Salmivuori et al.^153^ used the same system to image 16 BCC, where HSI had superior lesion delineation in 12 cases and missed the subclinical extension in two cases. Pardo et al.^326^ acquired a VIS-NIR HSI dataset of 124 samples from 116 patients with nevi, raised nevi, or melanoma. A reservoir-based kernel density estimation framework was proposed to segment the lesion using the spectral signatures, as shown in Fig. 14, and they achieved a best performance of 96% accuracy, 96.8% sensitivity, and 95.7% specificity with leave-one-out cross-validation. In 2021, Courtenay et al.^133^ implemented statistical analysis on spectral signatures in the VIS-NIR range specifically for nonmelanoma skin cancers. They reported that the major differences among SCC, BCC, and healthy tissues are located between 573.45 and 779.88 nm. In addition, Calin and Parasca^145^ investigated the detection of nonmelanoma BCC in the hyperspectral image from one patient using anomaly detection algorithms. Liu et al.^293^ developed a dual-modality microscopy integrating both photoacoustic and hyperspectral imaging to evaluate and differentiate skin carcinoma *in vivo*. The system was tested on tumor-bearing mouse ears, where increased blood flow velocity and hypoxia were clearly revealed by photoacoustic and hyperspectral imaging, respectively.

Since 2020, deep learning has been playing an active role in hyperspectral skin cancer detection. Hirano et al.^476^ trained and evaluated a GoogLeNet-based network using VIS-NIR hyperspectral images of 619 lesions (melanoma and nonmelanoma), where they reached a fivefold cross-validation accuracy of 77.2%, as well as 72.3% sensitivity and 81.2% specificity. In 2020, Leon et al.^119^ acquired 76 hyperspectral PSL images from 61 patients using the handheld device developed earlier^118^ and implemented segmentation with k-means clustering as well as SVM classification using spectral signatures. La Salvia et al.^479^ used a vision transformer on this dataset and got a promising performance for malignant melanocytic (91% accuracy) and benign melanocytic (84% accuracy), but a less satisfactory accuracy for benign and malignant epithelial, being 63% and 41%, respectively. Paoli et al.^561^ acquired a dataset of 325 lesions from 285 patients, with which they trained and tested a modified Hyper3Dnet. With a majority vote, they achieved 95% accuracy for two-class (benign versus malignant) classification and 88% accuracy for multiclass (different types of lesions) classification. However, they used the left side of the images for training and validation and the right side for testing, which may have introduced data leakage. Based on a dataset of 28 *ex vivo* PSL samples from 21 patients, Aloupogianni et al.^319^ implemented pixel-wise classification using SVM and patch-wise classification using a 3D Xception network. The results in the test data showed that the neural network gave a clearer mask of the tumor, whereas SVM had a better detection of tumor tissues. Huang et al.^562^ employed the YOLO-v5 network to detect skin cancer, including BCC, SCC, and seborrheic keratosis. Hyperspectral narrowband images (NBIs) were generated from an RGB skin cancer dataset, and both were used to train the YOLO-v5 network for comparison. The results showed that the HSI-NBI model could achieve comparable performance to the RGB model with significantly increased sensitivity for SCC.

*In vivo* skin cancer detection has driven the development of portable HSI systems. Zheludev et al.^36^ employed a handheld HSI device for the *in vivo* delineation of LM and LMM. The device could acquire data in a wavelength range of 500 to 885 nm at once with an FPI. The device was tested on eight patients (three LMM and five LM cases), and they implemented classification with the regression trees as well as the average nearest neighbors decision unit. Fabelo et al.^118^ developed a handheld device in the range of 450 to 950 nm with 125 bands for assisting PSL diagnosis. Hyperspectral data of various lesions, including nevus, BCC, and melanoma, were obtained and processed. They used the spectral signatures to train and test an SVM classifier, which achieved over 99% accuracy for nevus, BCC, and melanoma, as well as an accuracy above 98% for other lesions. Based on this design, La Salvia et al.^462^ refined the handheld device with a low-power GPU for real-time clinical testing. In another study, Hosking et al.^74^ used a 21-band handheld imaging device in the 350 to 950 nm wavelength range to image pigmented lesions, after which hand-coded features were extracted for classification using machine learning algorithms. Even though they achieved a sensitivity of 100%, the specificity still could be improved, being only 36%. Lindholm et al.^39^ exploited a 33-band VIS-NIR handheld SICSURFIS spectral imager to study lesions on complex surfaces. They trained two CNNs for a pixel-wise three-class classification of pigmented lesions and a pixel-wise four-class classification of nonpigmented lesions, respectively. Similar to the study by Paoli et al.,^561^ they also used different regions from the same images for training and testing.

#### 4.2.5 Gastrointestinal cancer

Gastrointestinal cancers comprise approximately one-quarter (24.6%) of new cancer cases globally and over one-third (34.2%) of all cancer-related deaths, posing a significant health burden on global public health and clinical care efforts.^563^ The highest incidences of the most prevalent GI malignancies are from colorectal, gastric, liver, esophageal, and finally pancreatic cancers.^564^^–^^566^ Reducing the toll of GI cancers requires continuous coordination across research, detection, and treatment. Therefore, HSI poses as an important technological advancement for enhancing early GI cancer detection during endoscopy procedures and improving tumor delineation.

##### Esophageal cancer

There are ∼0.6 million diagnoses of esophageal cancer every year, with a high mortality worldwide.^325^ Esophageal carcinoma includes squamous cell carcinoma and adenocarcinoma.^327^ Because esophageal cancer does not exhibit early biomarkers, early detection can be difficult.^567^ Endoscopy is currently most commonly used for the detection of abnormalities, and surgery resection is the primary potentially curative treatment approach.^327^

Maktabi et al.^327^ intraoperatively acquired hyperspectral images of *ex vivo* esophago-gastric tissues from 11 patients, among which nine patients were used for five-fold cross-validation and two patients for testing. With an SVM classifier, they obtained 63% sensitivity and 69% specificity for the differentiation of tumor and normal tissues. Note that even though their system covered the 500 to 1000 nm wavelength range, only bands in 525 to 610 nm were used as they observed a greater difference between cancerous and normal tissues in that range. Collins et al.^325^ employed a commercial intraoperative hyperspectral imaging system and acquired data of resected tissue samples from 10 patients with esophagogastric cancer. A 3D CNN was trained for cancer detection using the leave-one-out cross-validation strategy and achieved an average AUC of 0.91.

As endoscopy is critical for the early detection of esophageal cancer, research has been done to investigate esophageal cancer detection using endoscopic HSI. Yoon et al.^9^ developed a hyperspectral endoscopy system (HySE), which simultaneously records co-registered hyperspectral and standard white-light images. They evaluated the system by imaging 12 *ex vivo* tissues from three patients, including normal gastric mucosa, normal squamous epithelium, normal submucosa, Barrett’s esophagus, and esophageal cancer. The spectral angle between various tissue types and the reference (average spectrum of esophageal cancer) revealed sufficient differences for discrimination. Grigoroiu et al.^194^ acquired 300 images of color charts using HySE and trained a five-layer CNN for color classification, which they later tested on human gastric-esophageal biopsies for tissue differentiation based on color classification. Yang et al.^568^ evaluated esophageal cancer detection in endoscopic images using white light and different types of NBIs. Despite hyperspectral images in this study being reconstructed from white-light images (WLIs), results suggested that HSI-simulated NBIs, as well as NBIs generated from WLIs using CycleGAN, are superior to conventional WLIs and NBIs.

In addition to the *ex vivo* studies, one group carried out a series of work for *in vivo* endoscopic esophageal cancer detection.^341^^,^^466^^,^^468^^,^^568^ Wu et al.^341^ acquired *in vivo* endoscopic images of esophageal tissues and reproduced hyperspectral data in the 380 to 780 nm range based on a pre-determined transformation matrix between various colors and their spectral signatures, to observe the spectra of normal, dysplasia, and cancerous esophageal tissues. Tsai et al.^466^ applied this method to generate hypercubes from endoscopic WLIs and NBIs, and they trained a VGG-16-based network for *in vivo* esophageal cancer detection. The accuracy of using the WLI-reconstructed hyperspectral image was 88%, and that of the NBI-reconstructed data was 91%, both being 5% higher than using only the original endoscopic images. In addition, using the spectral data resulted in a significant increase in sensitivity for normal, low-grade dysplasia, and high-grade dysplasia, though with a slight drop in the sensitivity of invasive cancer. Later, Yang et al.^568^ and Tsai et al.^468^ generated NBIs by selecting two bands (415 and 540 nm) from the WLI-reconstructed hypercubes, and Tsai et al.^468^ compared the classification performance using the original WLI, NBI, and hyperspectral-simulated NBI. The latter achieved the best overall accuracy of 90.7%, which was 2% higher than that of the WLIs and 2.4% higher than NBIs.^468^ Specifically, using the simulated NBI significantly improved the sensitivity for SCC and dysplasia, whereas the original NBI worked better for normal tissues.

##### Gastric cancer

Often referred to as stomach cancer, gastric cancer remains the fifth most prevalent malignant cancer in the world despite its decreasing incident rates over the past several decades.^569^^,^^570^ HSI provides a promising, noninvasive modality for more accurate differentiation between noncancerous and cancerous lesions within gastric tissue. Goto et al.^571^ imaged 104 gastric tumors resected from 96 patients, where a quarter of the cases were used to select the optimal wavelength for tissue differentiation based on the Mahalanobis distance between tumor and normal tissues. They claimed that 770 nm gave the best results with 71% sensitivity, 98% specificity, and 85% accuracy on 50 testing cases. However, only one band was used within the range of 400 to 800 nm, thus not fully exploiting the rich features of HSI. Li et al.^55^ utilized a fluorescence HSI system and imaged *ex vivo* gastric tissue samples. With an SVM classifier, they achieved 97.5% accuracy for gastric tumor detection based on tissue fluorescence spectra. In another study of Li et al.,^56^ they trained a ResNet34 for the classification among nonprecancerous and precancerous lesions as well as gastric cancers. 3D patches from hypercubes were reshaped into 2D spatial–spectral images to train the network, which achieved 96.5% overall accuracy. However, the data split for the training, validation, and testing in both studies was random; thus, there was possible data leakage that biased the results.

In addition to the visible wavelengths, a few studies looked into the power of HSI in the NIR range. Liu et al.^132^ imaged gastric tissues with a push-broom HSI system in the range of 900 to 1700 nm, selecting six out of all 168 bands based on PCA, which were then used for tissue differentiation with an SAM. They reported an accuracy up to 90% as well as three wavelength bands (975, 1215, 1450 nm) that showed major spectral differences between cancerous and normal gastric tissues. Another study by Sato et al.^572^ investigated specifically gastrointestinal stromal tumors in the range of 1000 to 2350 nm. With an SVM classifier trained in the leave-one-out cross-validation fashion, they achieved 86.1% accuracy, 91.3% sensitivity, and 73% specificity in surgically removed gastric tissues. In addition, they stated that even with the mucosal layer covering the lesion in multiple samples, NIR HSI could detect the tumor underneath. Mitsui et al.^411^ investigated the impact of tumor thickness on the detection of both exposed and unexposed tumors using NIR HSI. The results showed that tumor tissues with a thickness of >2  mm would be easier to correctly identify, even if unexposed (covered by normal tissue).

##### Colorectal cancer

Colorectal cancer is the third most common type of cancer and the second most common cause of cancer death worldwide.^564^^,^^573^ Differentiating malignant and nonmalignant colorectal tissues is one of the most critical tasks during diagnosis and surgical operation. Han et al.^34^ carried out an *in vivo* study by developing an HSI endoscope system based on a filter wheel and acquiring 21 hyperspectral images from 12 patients with colorectal tumors or adenomatous polyps. They implemented a band selection based on recursive divergence and spectral classification using an SVM. Despite the selected bands varying from patient to patient, certain bands, such as 525 nm and 625 nm, were more frequently seen in the results. In addition, the classification using all 27 wavelength bands had better performance than using only one to five selected bands. The spectral difference of the 525 nm band was also reported in a study of Kumashiro et al.^574^ where both *ex vivo* and *in vivo* colorectal tumors were imaged using a macroscope and an endoscope, respectively.

**Fig. 15:**
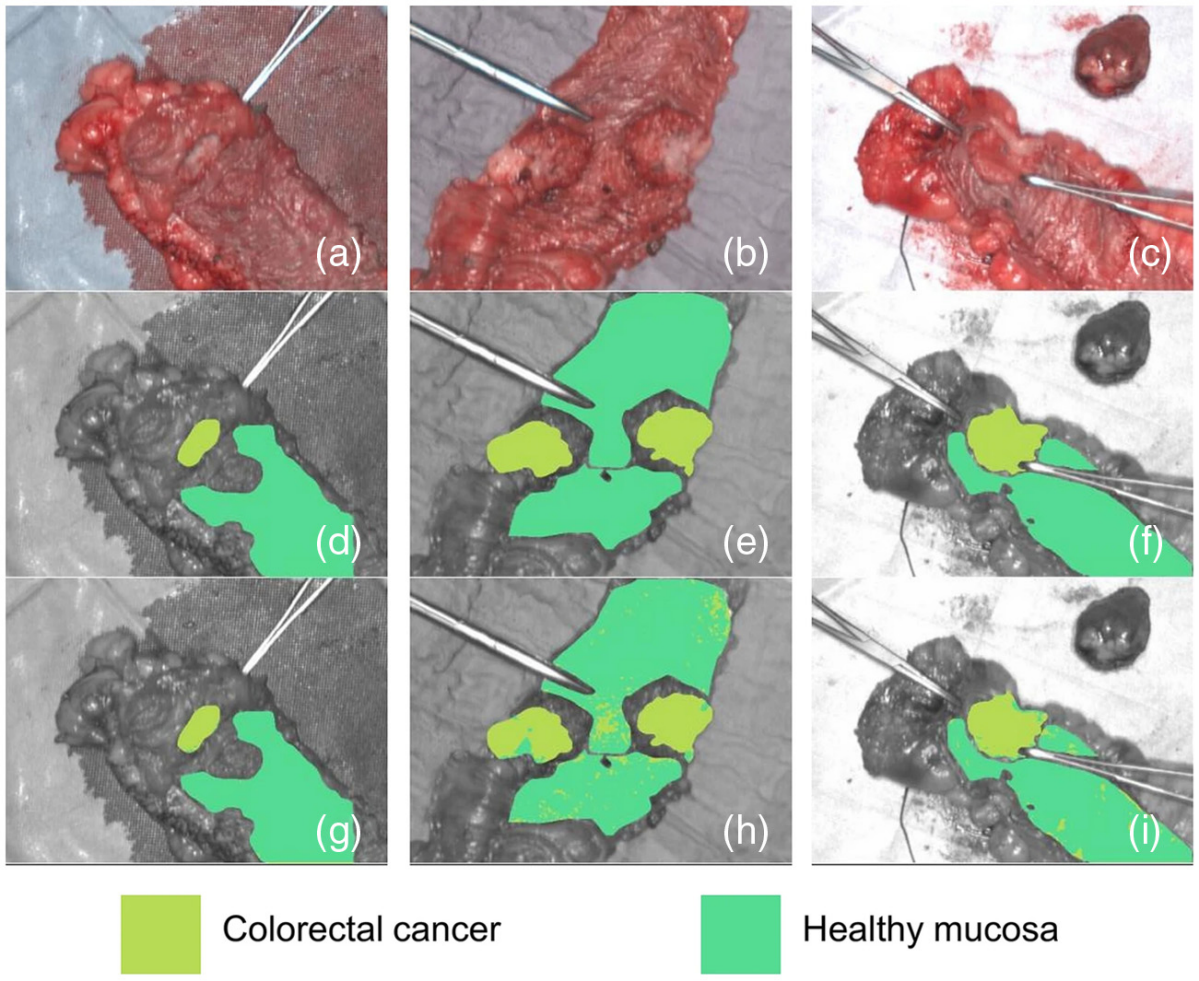
Visualization of automatic colorectal cancer detection by CNN from hyperspectral data. (a)–(c) HSI-synthetic color images of three tissue specimens with locally advanced stage cancer from the right (a), transverse (b), and left (c) colon. (d)–(f) The reference annotations that demarcate regions of cancer and healthy mucosa. (g)–(i) The automatic detections produced by the CNN.^573^ Reproduced with permission, courtesy of Springer Nature.

Targeting HSI-guided laparoscopic surgery, Baltussen et al.^323^ imaged tissue samples comprising tumor, healthy colon wall, and fat from 54 patients using VIS-NIR and NIR cameras. After combining images from two cameras, a two-step classification algorithm was developed to distinguish three types of tissues with an overall accuracy of 88%. Manni et al.^418^ investigated colon cancer detection in six *ex vivo* specimens. They employed the HybridSpectraNet, which comprised both 3D and 2D convolutional layers and achieved 0.82 AUC, 0.88 sensitivity, and 0.78 specificity in their leave-one-out cross-validation. In one of the previously mentioned references, Collins et al.^325^ explored tissue classification of surgical samples of colon cancer in addition to esophagogastric cancer samples. The 3D CNN trained on colon cancer tissues from 12 patients achieved an average AUC of 0.93. Interestingly, they also combined the dataset of two cancer types to train the model, which resulted in a 0.92 average AUC on colon cancer samples and 0.93 AUC on esophagogastric samples. Nevertheless, the network performance with dataset cross-training—training on one cancer type and testing on the other—was significantly decreased. Later, they evaluated the network on a larger dataset containing samples from 34 patients with k-fold cross-validation and achieved an overall 87% sensitivity and 90% specificity, as shown in Fig. 15, whereas the performance was better on tumors of local advanced stages than those of local initial stages.^573^ Maktabi et al.^424^ obtained physiological parameters, i.e., tissue oxygen saturation (${\mathrm{StO}}_{2}$), perfusion index (PI), tissue water index (TWI), and tissue hemoglobin index (THI) of resected colon tissues from hyperspectral images acquired with a commercial HSI system, and they trained a 3D CNN with Inception blocks using the physiological parameters for colon cancer detection, which resulted in 0.72 sensitivity and 0.90 specificity. Later, Tkachenko et al.^405^ from the same group trained an inception-based 3D CNN with hyperspectral images of *ex vivo* colon tissues combined with pre- and post-processing and got 92% sensitivity as well as 94% specificity.

##### Liver cancer

Hepatocellular carcinoma (HCC) and cholangiocarcinoma (i.e., bile duct cancer) are the two main types of adult primary liver cancer.^575^ Duan et al.^576^ evaluated the feasibility of HSI for liver tumor discrimination in five rabbits, where tumor spectra displayed different absorption peaks compared with normal tissue. Wang et al.^509^ imaged 14 HCC slices, each 40  μm thick, from 14 different patients in the wavelength range of 400 to 750 nm and used a 1D CNN for the pixel-wise classification of tumor and normal tissues. With leave-one-out cross-validation, they achieved an average performance of 0.881 accuracy, 0.871 sensitivity, 0.888 specificity, and 0.95 AUC. Zhang et al.^367^ implemented a multitask U-Net framework, which had a decoder and a parallel classification branch following the encoder, for the delineation of liver tumors in tumor-normal margin surgical specimens. A total of 36 surgical specimens from 19 patients with HCC or intrahepatic cholangiocarcinoma were imaged. The network was also implemented in a leave-one-out cross-validation fashion and resulted in an average accuracy of 93.16%, sensitivity of 94.48%, and specificity of 87.22%. In addition, they employed a saliency map strategy to help explain the judgment of the network spatially and spectrally. With two saliency-weighted channel selection methods proposed in this study, they selected five dominant bands, i.e., 420, 605, 702, 993, and 440 nm (or 847 nm, each for one channel selection method), and obtained a classification performance comparable to using all 224 bands in the range of 400 to 1000 nm. In another study by Schneider et al.,^85^ the absorption spectra of colorectal cancer liver metastasis (CRLM) and HCC in the wavelength range of 1100 to 1700 nm were analyzed. Though based on a very small number of patients (N=3 for CRLM and N=2 for HCC), this study reported an increased heterogeneous absorption in CRLM tissues, whereas the opposite was observed for HCC. In addition, they employed a generalized linear mixed model and analyzed the association of each spectral band with the presence of liver tumors. The results indicate that absorption at 1130, 1410, and 1690 nm is strongly associated with CRLM, whereas 1140, 1400, and 1450 nm reveal distinct absorption characteristics for HCC. Furthermore, HSI does not only facilitate tumor detection but has also helped the feature selection for liver tumor detection in CT images. Wang et al.^435^ proposed to use hyperspectral attention to assist the selection of radiomics features in CT images. They evaluated the correlation between 120 radiomics features extracted from CT images and 103 features from hyperspectral images. Fifty radiomics features were selected based on the strong correlation with HSI, and the classification using these 50 features resulted in a comparable performance to that using all 120 features.

#### 4.2.6 Other cancer types

In addition to the abovementioned cancer types that have been investigated, HSI has also shown potential for the detection of other types of cancers.

For kidney tumor, Biel et al.^577^ used HSI to monitor ${\mathrm{SO}}_{2}$ for tumor microvascular oxygenation assessment in tumor-induced mice. Stewart et al.^45^ evaluated VIS-NIR HSI tumor detection on *ex vivo* human kidney tumor samples, in addition to *in vivo* mice with breast and lung tumor xenograft. They utilized a PLS-DA model with a hard threshold for classification, which was trained with 440 renal tumor spectra and 1090 nontumor spectra from 22 human kidney specimens in the wavelength range of 520 to 1050 nm. The model achieved 93.5% accuracy, 88.6% sensitivity, and 95.4% specificity for renal tumor detection.

Kameyama et al.^578^ investigated intraocular tumors using an NIR HSI system. They imaged ocular fundus lesions of five choroidal melanoma patients and 12 patients with other intraocular tumors. Spectral angles were calculated using the spectra of the nerve head and peripheral region and a self-reference spectrum of the imaged lesion. Then, a discriminative index was obtained for each lesion from its normalized distribution of spectral angles, which they reported to be effective for discriminating uveal melanoma from other intraocular tumors.

In 2022, van Vliet-Pérez et al.^160^ imaged 26 ovarian tissue samples from 10 patients undergoing cytoreductive surgery with a red-near-infrared (RNIR) snapshot mosaic camera (665 to 975 nm, 25 bands). The hypercubes contained 26,446 data points, where they extracted the intensities, derivatives, and intensity ratios as features. With an SVM classifier, they obtained 0.81 sensitivity, 0.70 specificity, and 0.83 AUC based on the relatively small dataset.

Another unique study was carried out by Chen et al.,^410^ where four types of blood cancer samples and one healthy control blood sample were imaged for the detection of blood cancer. Serum samples were obtained by centrifugation and then dripped and air-dried on a substrate, which was scanned using a push-broom HSI system. They retrieved a total of 320 spectra and formed four datasets, each of which is for a binary classification between one cancer type and the health control. Among various traditional machine learning classifiers that were tested, SVM got the best performance with an accuracy of 94.8% for acute myeloid leukemia, 94.5% for chronic myeloid leukemia, 93.1% for multiple myeloma, and 95.6% for lymphoma. The results were very promising; however, the small dataset and unclear patient count and data split raise the possibility of training and testing on the same patient, potentially introducing bias.

Finally, two studies were conducted on cervical cancer detection. In 2016, Wang et al.^26^ investigated HSI cervical neoplasia detection at both the *in vivo* tissue level and cellular level. In 2023, Schimunek et al.^579^ imaged *in vivo* cervical intraepithelial neoplasia from 41 patients in the wavelength range of 500 to 1000 nm and reported a major spectral difference in the 555 to 585 nm wavelength range.

### 4.3 Diagnosis of Nonmalignant Diseases and Conditions

Over the past 10 years, HSIs and their usage in noncancerous disease detection and diagnosis have grown tremendously. The imaging technique seeks to improve patient outcomes through enhancing existing diagnostic methods or providing more efficient and accurate alternatives. In this section, HSI’s role in diagnosing noncancerous-related diseases and conditions, such as dermatological conditions, GI tract diseases, cardiac diseases, neurological diseases, renal and hepatic diseases, dentistry applications, and more, is explored and expanded upon.

#### 4.3.1 Neurological diseases

The CNS comprises the brain and spinal cord, with the brain accounting for over one-fifth of the body’s total oxygen consumption.^580^ This means the system, despite its relatively smaller percentage of body weight, has enormous importance and requires a large amount of metabolic energy to maintain. The peripheral nervous system (PNS) plays a vital role in transmitting signals between the CNS and the rest of the body^581^; thus, diseases affecting the PNS can have wide-ranging consequences. HSI can be used to monitor the metabolic activity and chemical deposits of the nervous system to identify and monitor neurological diseases. Here, we review several diseases and disorders of the CNS and PNS: ischemia, seizures, Alzheimer’s disease, and Parkinson’s disease (PD).

HSI can identify ischemic regions by detecting changes in oxygen saturation and hemoglobin concentration, providing real-time insights into the extent and progression of damage.^44^^,^^302^^,^^529^ Furthermore, these changes can also be useful critical markers of dysfunction before the structural damage occurs, enabling timely or even early intervention for conditions such as neurodegeneration and ischemia.^529^ Cerebrospinal fluid, which cushions the brain and removes metabolic waste from the brain and spinal cord, can also be analyzed using HSI to detect abnormalities.^582^ Spectral variations in cerebrospinal fluid can indicate inflammation, infection, or pathological proteins associated with conditions such as meningitis or neurodegenerative diseases. Mathieu et al.^582^ leveraged HSI to identify the fluorescence emission of the quantum dot nanoparticles, which were used to monitor cerebrospinal fluid uptake in mice. Pichette et al.^108^ developed a snapshot HSI system capable of video-rate responses (20 fps) to monitor ${\mathrm{HbO}}_{2}$ and HHb in the human cortex during open-cranium surgical procedures. Giannoni et al.^65^^,^^302^^,^^531^ used HSI, mostly in the red and NIR range, to track changes in hemoglobin, ${\mathrm{HbO}}_{2}$, and oxidized CCO concentrations. They tested the system on the exposed mouse cortex during normoxic, hypoxic, and hyperoxic states, finding strong correlations between system outputs and oxygen levels. Fu et al.^44^ demonstrated the ability of HSI to identify ischemic regions in rat brains, showing that their results correlated well with histology in both *in vivo* and *ex vivo* experiments.

Seizures result from abnormal electrical activity in the brain, disrupting normal neuronal function and leading to temporary changes in behavior, sensation, or consciousness. Monitoring metabolic activity, such as oxygen consumption and hemoglobin concentration, can help identify regions of heightened neural activity associated with seizures.^188^ HSI provides a noninvasive method to detect these metabolic changes, offering insights into seizure onset and progression, which can aid in diagnosis and guide therapeutic interventions. Noordmans et al.^188^ reported a case of using HSI alongside an electrocardiogram to monitor a seizure episode in a patient. They identified a local increase in oxygenation rate during seizures, corresponding to an area of heightened electrical activity.

Alzheimer’s disease is a neurodegenerative disorder characterized by progressive cognitive decline and memory loss, driven by the accumulation of amyloid beta (Aβ) plaques and neurofibrillary tangles.^75^^,^^318^^,^^381^ Amyloid beta aggregates disrupt neuronal communication, trigger inflammation, and lead to oxidative stress.^583^ Because amyloid beta also accumulates in the retina, HSI can be applied noninvasively to detect these deposits by imaging the retina rather than the exposed cortex. More et al.^88^^,^^139^ studied transgenic mice and found that amyloid beta caused retinal scattering in the 450 to 600 nm range, with amyloidopathy observed before cognitive decline. They also reported a monotonic increase in scattering with age and disease progression. Hadoux et al.^76^ extended these findings to humans, where they achieved 87% AUC in distinguishing mild cognitive impairment in a study of 15 patients and 20 controls. Lemmens et al.^101^ combined HSI with OCT to extract both spectral data and retinal nerve fiber layer thickness, showing a slight improvement in classification accuracy for AD.

Parkinson’s disease is a neurodegenerative disorder characterized by motor deficits, alpha-synuclein aggregation, and iron overload, particularly in the substantia nigra and retina. Retinal changes, such as increased alpha-synuclein levels and iron accumulation, provide a window into the disease mechanism and progression.^87^ Trlin et al.^87^ studied two PD mouse models—one for alpha-synuclein overaccumulation and another for iron deposition—finding consistent spectral differences in the retina compared to healthy controls, especially in the 400 to 600 nm range. Ueda et al.^584^ reported similar results in human participants, confirming similar spectral changes, highlighting the potential of retinal imaging in PD research.

#### 4.3.2 Ophthalmic diseases

The retina, which consists of the optic nerve, is a direct extension of the CNS. Using the retina as a biomarker not only reveals health features that are related to vision, but also to the overall health of the CNS. We focused on two different types of features that are commonly used by HSI: the microvasculature of the retina and the retinal surface, including the retinal pigment epithelium (RPE). With systems being developed that enable easy spectral fundus imaging,^33^^,^^103^^,^^104^^,^^585^ applications were numerous: a literature review conducted by Lemmens et al.^141^ in 2020 found a total of 361 studies that used HSI or MSI to image the retina. Diseases diagnosed included glaucoma, age-related macular degeneration, retinitis pigmentosa, chronic iridocyclitis, Stargardt’s disease, retinopathy, macular edema, choroidal melanoma, diabetes mellitus, Alzheimer’s disease, and Parkinson’s disease. Neurological diseases such as AD and PD have been covered in the last section, whereas diabetic retinopathy will be discussed in Sec. 4.3.8.

The microvasculature of the retina can be used to identify hypoxic events. Lower oxygenation rate to the retina can correspond to lowered metabolic activities. As such, many researchers focused on quantifying oxygen saturation in the venules and arterioles of the retina without the use of angiographic dye.^103^^,^^104^^,^^114^^,^^392^^,^^586^^,^^587^ For example, Mordant et al.^588^ reported significantly higher venular oxygen saturation in glaucoma eyes compared with baseline, regardless of the stage of the disease, whereas Desjardins et al.^586^ observed a 12.5% decrease in mean optical nerve head saturation in retina during systemic hypoxia. Dwight et al.^113^ evaluated a hyperspectral funduscope for oximetry measurement in four diseased eyes and obtained ${\mathrm{sO}}_{2}$ values consistent with published data. Subsequently, authors utilized this system for retinal angiography to map ${\mathrm{HbO}}_{2}$ abundance in the retina.^114^ Shahidi et al.^589^ studied relationships between ${\mathrm{sO}}_{2}$ and the severity of glaucoma. Authors found that the arteriole-venule oxygenation differences did not significantly alter for most of the patients. However, within groups with severe glaucoma and severe field defects, the arteriole-venule differences were significantly lower. Venular oxygenation rate did have a correlation with the severity of glaucoma in patients with highly degraded visual fields, but not in any other groups. Rose et al.^590^ studied radiation retinopathy and found that the average arteriolar oxygen saturation and venular oxygen saturation were higher in the retinopathic eyes compared with those in healthy eyes. In addition, ${\mathrm{sO}}_{2}$ of the retinal microvasculature can be used to identify diabetes, which will be elaborated in Sec. 4.3.8.

The macular pigment, composed primarily of lutein and zeaxanthin,^114^ is concentrated in the central retina and plays a critical role in protecting retinal cells from oxidative damage by filtering harmful blue light. Its unique spectral properties make it an important feature for HSI, allowing the differentiation of healthy retinal tissue from pathological changes associated with conditions such as macular degeneration and other neurodegenerative diseases. In Sec. 4.3.1, we covered how neurodegenerative diseases deposit chromophores into the macula. Diseases such as age-related macular degeneration also affect the macula. Kaluzny et al.^103^ used a method to calculate the macular pigment optical density using the reflectance value measured at 465 and 547 nm wavelengths. They showed that the macula was severely degraded in an 80-year-old individual, compared with that of a healthy 24-year-old volunteer. Fan et al.^461^ studied hydroxychloroquine (quinine) retinopathy, which is the side effect of consuming quinine and manifests as blurred vision and headaches. As quinine exhibits affinity to melanin, quinine can accumulate in the RPE. The authors used ResNet to classify images of retina and found that with the addition of HSI data, the authors improved the classification accuracy over simply using RGB data (from 93% to 96% accuracy).

Last, HSI was proven effective for label-free imaging of corneal epithelium injuries,^339^ which usually requires contact-based dye staining by conventional methods. Reflectance spectra of *ex vivo* porcine eyes revealed spectral differences between injured and healthy eyes. With image enhancement and machine learning approaches, a detection accuracy of up to 100% was achieved.^339^

#### 4.3.3 Dermatological conditions

Nonmalignant dermatological diseases, including PSL, chronic wounds, and inflammatory conditions, are among the most prevalent health concerns worldwide. To improve diagnostic accuracy, several noninvasive imaging modalities have been developed and implemented in dermatology, including dermoscopy, OCT, and reflectance confocal microscopy.^79^^,^^255^ Although these techniques have proven valuable, each comes with specific limitations that hinder their ability to provide comprehensive, quantitative, and depth-resolved tissue characterization. For example, dermoscopy enhances the visualization of subsurface skin structures by magnifying and polarizing the image, but it still primarily relies on morphological features and color interpretation, which can be subjective.^591^ OCT offers cross-sectional images of skin at a micrometer-scale resolution, but its imaging depth is typically limited to 1 to 2 mm,^592^ and its contrast relies on scattering properties rather than biochemical composition.^593^ Similarly, reflectance confocal microscopy allows for high-resolution imaging of the epidermis and superficial dermis,^592^ but its limited penetration depth (∼200 to 300  μm^594^^,^^595^) restricts its utility in evaluating deeper lesions. By contrast, HSI provides spatially resolved spectral information across a wide range of wavelengths, enabling the identification of biochemical and structural tissue features, offering a unique capability to noninvasively quantify tissue composition, perfusion, and oxygenation in a label-free manner. Several researchers have dedicated efforts to developing HSI systems for *in vivo* assessment of skin.^70^^,^^249^^,^^267^^,^^324^^,^^537^ These advantages, together with the improvement of HSI systems, position HSI as a promising imaging modality for a broad spectrum of dermatological applications, including skin characterization, burn and wound assessment, and inflammatory condition evaluation.

##### Estimate of optical and physiological parameters

Skin is the largest organ in the human body.^596^ The optical and biological parameters of skin are important for various purposes such as diagnosis, understanding tissue properties, and tracing the growth of skin cancers.^295^ Most commonly investigated parameters include melanin concentration,^6^^,^^105^^,^^249^^,^^267^^,^^295^^,^^296^^,^^537^ collagen concentration,^295^ oxygen saturation,^6^^,^^105^^,^^249^^,^^267^^,^^295^^,^^296^ blood volume fraction (BVF),^6^^,^^105^^,^^296^ layer thickness,^105^^,^^596^ water content, and subcutaneous reflectance.^295^ Each of these parameters plays a significant role in human skin health. For example, melanin and hemoglobin are two major chromophores that determine human skin color^248^ and are often involved in PSL^78^; oxygenation and perfusion are critical biomarkers for not only the health of skin but also the diagnosis and therapeutic assessment of various conditions, such as the evaluation of peripheral vascular diseases and monitoring flaps in plastic and reconstruction surgery.^378^^,^^597^

A variety of studies have been done to characterize different skin parameters using HSI. Koprowski et al.^598^ conducted a preliminary study to assess melanin and hemoglobin distribution in different segments of human hand skin. Denstedt et al.^296^ used wavelet decomposition to extract features from hyperspectral data in the spectral domain; then, the authors correlated those features with physiological parameters and provided visualization of tissue oxygenation, blood content, and melanin content. Zherebtsov et al.^105^ trained separate MLPs to retrieve BVF, oxygen saturation, melanin concentration, and epidermal thickness from hyperspectral images of phantoms and human volunteers. To solve the inaccurate estimate caused by the interference between melanin absorption and hemodynamics measurements, Vasefi et al.^77^^,^^78^ developed a multimode hyperspectral dermoscope integrating both hyperspectral and polarization-sensitive imaging capabilities. The system was used to map the distribution of melanin and hemoglobin in nevi separately. He et al.^249^^,^^537^ used weighted subtractions between bands to isolate absorption of hemoglobin and melanin, demonstrating the feasibility of HSI for the detection of pimples, nevi, and red spots. A snapshot HSI camera with motion-artifact immunity was employed to map cutaneous blood absorption and perfusion for skin morphological feature analysis,^249^ and later, their high-resolution images were reconstructed from snapshot hyperspectral images for sharper vascular mapping.^537^ To further simplify the system and reduce the cost, they implemented the chromophore analysis using hyperspectral images reconstructed from RGB images captured with a smartphone.^267^ One study by Calin et al.^314^ analyzed skin texture (homogeneity, contrast, entropy, and correlation) in relation to wavelength, age, and sex, suggesting HSI’s value in cosmetics and aging evaluation. Focusing on 650 to 700 nm, where melanin absorption dominates, they developed an analytical model based on spectral unmixing to map melanin concentration across different Fitzpatrick skin types.^143^ In another study by Tsai et al.,^599^ the difference of skin reflectance by demographic variables and body sites was examined in the visible and NIR range, establishing baseline knowledge of skin reflectance that is essential for advancing HSI and AI-based dermatological diagnostics. Furthermore, skin microcirculation was measured for the analysis of certain conditions. For example, Calin et al.^316^ employed HSI to assess the immediate change of skin oxygenation induced by photobiomodulation therapy. Rink et al.^600^ used HSI to quantify skin health and cutaneous perfusion in the residual limbs of individuals with lower limb amputations. Rubins et al.^102^ developed a multimodal device integrating HSI and thermal imaging for skin mottling and oxygen saturation measurement. Chin et al.^376^ used VIS-NIR HSI to quantify HHb concentration in mice that were exposed to different doses of radiation. The results suggested that HSI could detect subclinical microvascular damage, i.e., early HHb concentration drop, before visible skin reactions occur.

**Fig. 16:**
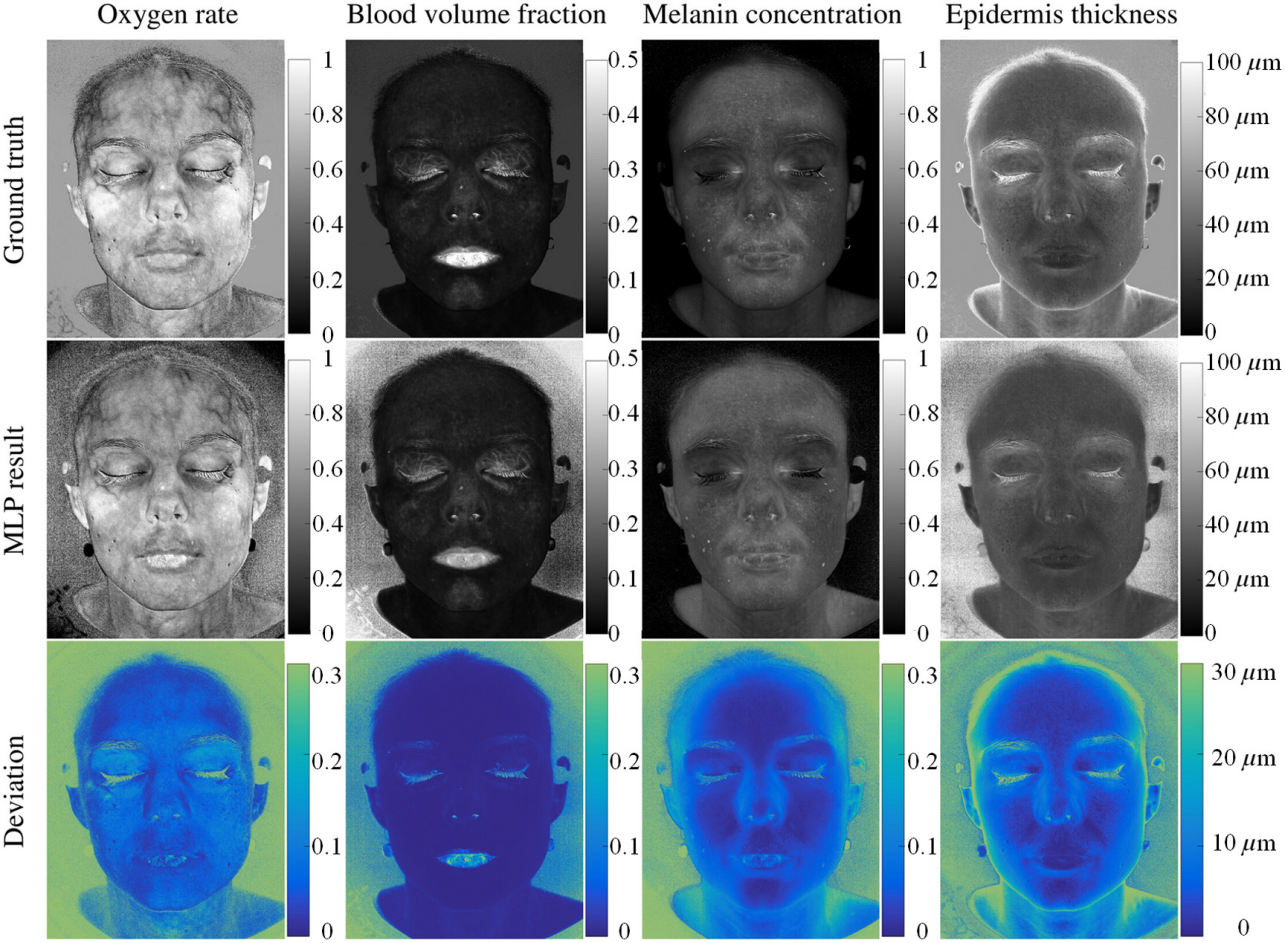
Skin parameter maps obtained using the MLP on a full-face image and the comparison with ground truth obtained using the optimization-based algorithm. Deviation between the two results is displayed in false colors. The figure was reproduced from Ref. 54 with permission from John Wiley and Sons.

To accurately map between the physiological parameters and spectral signatures of skin, many researchers developed human skin models. Forward light transport models usually treat skin as an N-layered (e.g., N=2, 3, 7, 10, or up to 22^6^^,^^53^^,^^105^^,^^295^) Lambertian material and map the physiological parameters to the spectra, mostly by the Monte Carlo algorithm^6^ or Kubelka–Munk methods.^295^ Inverse models, on the contrary, deduce physiological parameters from observed reflectance spectra.^295^ Vyas et al.^295^^,^^596^ used machine learning–based regression to estimate melanin, collagen, oxygen saturation, BVF, and skin thickness. Bjorgan et al.^6^ proposed a deterministic hyperspectral inverse model based on diffusion theory and spectral unmixing to estimate BVF, oxygenation, and melanin content in real time. Gevaux et al.^53^ developed a 3D HSI system to measure complex 3D objects such as the human face. The concentrations of chromophores (oxygen rate, BVF, and melanin concentration) were estimated by minimizing the difference between the measured spectral signatures and the theoretical spectra from a two-flux model. Later, the optimization process was replaced with a neural network for lower computational cost and real-time application.^54^ Their results of human facial skin chromophore estimation are shown in Fig. 16.

##### Quantitative evaluation of cutaneous inflammation and irritation

Inflammation and irritation, often manifesting as erythema, edema, or hyperpigmentation, are hallmark features of numerous dermatological conditions and systemic diseases. HSI has shown increasing promise as a noninvasive tool to evaluate inflammatory skin responses. The ability of hyperspectral data to distinguish among different tissue constituents, such as hemoglobin, melanin, and water, offers a more objective and quantitative approach to characterizing skin inflammation.

Kim et al.^51^ visualized spatiotemporal changes of inflammatory hyperemia in mice using RGB-reconstructed hyperspectral images. Chen et al.^601^ assessed cellulitis in a human patient’s legs using an RNIR snapshot hyperspectral imager. Moving forward, Du et al.^23^ used SWIR HSI to assess allergic contact dermatitis, which is pathophysiologically characterized by increased water content between epidermal skin cells. Three spectral bands, i.e., 1070, 1340, and 1605 nm, were extracted from the SWIR hypercube to form a pseudo-RGB image that displayed a strong contrast of water superior to the conventional RGB photography, and the proposed index served as a quantitative measure of the degree of the allergic reaction. These early demonstrations underscore the physiological specificity of HSI in capturing inflammation-related changes.

**Fig. 17:**
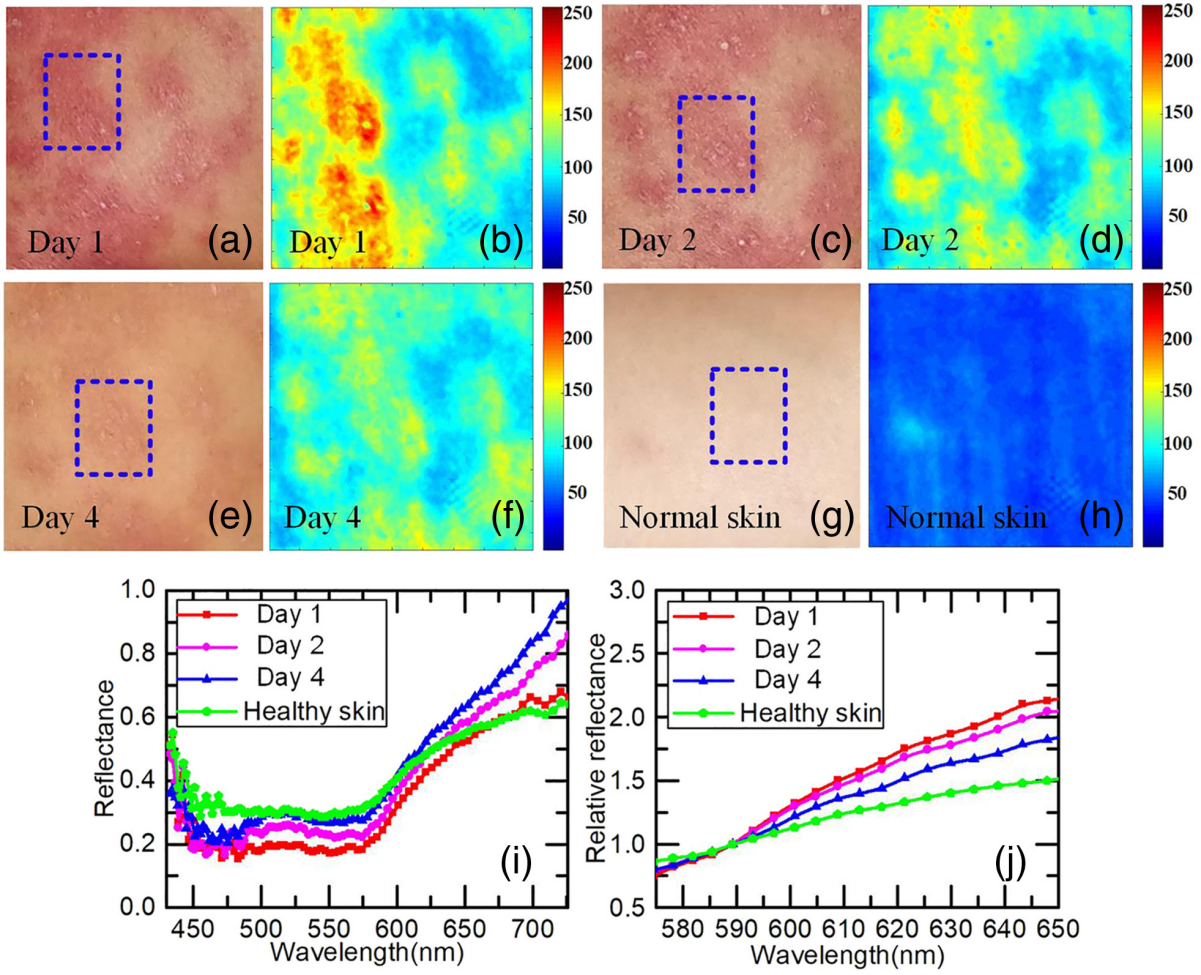
Single systemic lupus erythematosus lesion changes over 4 days. (a), (c), (e), (g) RGB images. (b), (d), (f), (h) Corresponding k-maps. (i) Average reflectance of the dashed boxes in panels (a), (c), (e), and (g). (j) Relative reflectance normalized to 590 nm. In panel (j), the steepness between 575 and 650 nm decreases as the patient’s condition improved.^151^ (j) Reproduced with permission, courtesy of John Wiley and Sons.

Moreover, researchers have paid particular attention to erythema as it is an early indicator of diseases. Zhang et al.^151^ monitored systemic lupus erythematosus patients using a snapshot hyperspectral FTIS, and a rash activity index was calculated based on the 575 to 650 nm bands to grade rash severity. The results indicated a negative correlation between the index and patients’ improvement, demonstrating the potential of the proposed method for auxiliary monitoring of skin lesions, as shown in Fig. 17. Targeting radiotherapy-induced skin erythema, an early stage of radiodermatitis, Abdlaty et al.^30^ proposed a Dawson relative erythema index, which was highly correlated with clinical visual assessment scores and was able to differentiate between irradiated and nonradiated skin regions. Hao et al.^79^ developed a low-cost HSI system that captured 11 spectral bands, and they implemented a Monte Carlo simulation to reconstruct spectra with higher resolution. By testing the system on three volunteers with manually induced erythema, they showed that the proposed method could not only reveal erythema-associated physiological changes, such as BVF and ${\mathrm{sO}}_{2}$, but also achieve erythema detection with 93.1% accuracy. Saknite et al.^37^ segmented skin erythema and hyperpigmentation in patients with cutaneous chronic graft-versus-host disease to present HSI’s ability to distinguish active inflammation from post-inflammatory hyperpigmentation in a challenging dermatological condition.

#### 4.3.4 Diseases of the cardiovascular system

Cardiovascular diseases encompass a wide range of conditions affecting the heart and blood vessels, often leading to impaired circulation, tissue damage, and organ dysfunction. Among these, atherosclerosis, aortic stenosis, and peripheral vascular diseases are significant contributors to morbidity and mortality worldwide, especially with the aging global population.^602^ These conditions often involve vascular narrowing due to plaque buildup, calcification, or endothelial dysfunction, ultimately restricting blood flow and oxygen delivery to critical tissues. In addition, structural heart diseases can cause conditions such as atrial fibrillation and myocardial scarring, which further complicate cardiovascular pathology, necessitating precise diagnostic and therapeutic strategies. HSI has emerged as a promising noninvasive tool for characterizing vascular and cardiac abnormalities, offering detailed spectral insights into tissue composition, perfusion, and metabolic changes.

**Fig. 18:**
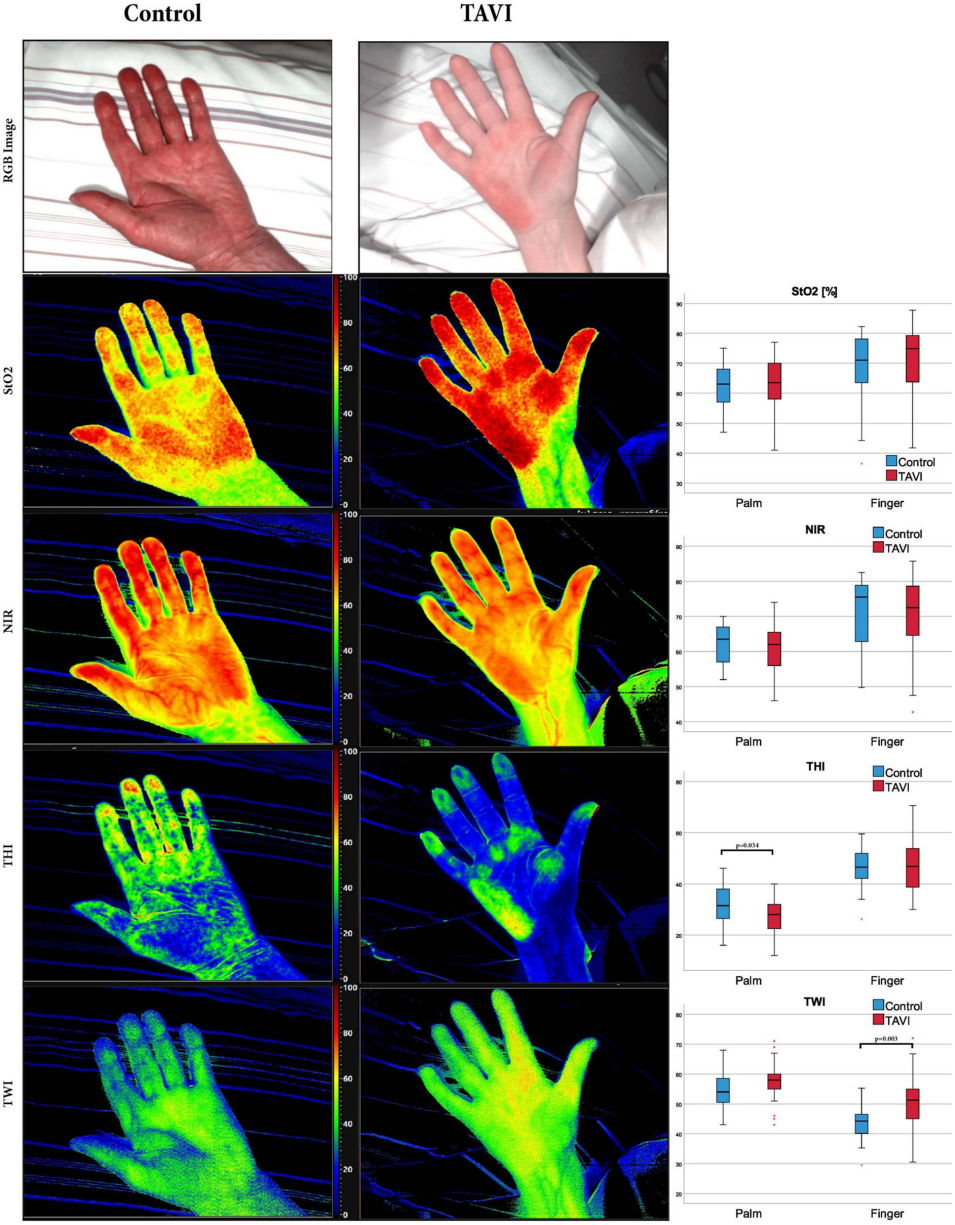
HSI-derived RGB image and parameter maps of the palm. Boxplots show the respective HSI measurements of TAVI and control patients; ${\mathrm{StO}}_{2}$, NIR PI, THI, and TWI are displayed color-coded (scale at the right side of each image). Red/yellow areas indicate high values (50 to 100), green/blue (0 to 50) areas indicate low values. The numerical scale ranges from 0 to 100. ${\mathrm{StO}}_{2}$ is given in %, and THI, NIR PI, and TWI are index values in arbitrary units. Mann–Whitney-U test was used for comparisons between groups.^604^

Atherosclerosis and aortic stenosis, which involve plaque buildup and valvular calcification, respectively, both contribute to the narrowing of vascular structures. To evaluate atherosclerotic plaque, Chihara et al.^603^ conducted *in vivo* HSI in the NIR range, analyzing spectral absorbance differences between normal arteries and atherosclerotic plaques in exposed abdominal aortas of rabbits. By applying an SVM classifier, they achieved an overall accuracy of 82.7% for plaque detection. In addition, they identified the most relevant spectral bands, particularly those associated with cholesterol absorption peaks, which are crucial for distinguishing atherosclerotic plaques from healthy tissue. Dietrich et al.^604^ used HSI on aortic stenosis patients undergoing the transcatheter aortic valve implantation (TAVI) to measure microcirculatory changes. HSI effectively detected changes in tissue oxygenation, hemoglobin levels, and water content, which correlated with complication and recovery outcomes, as shown in Fig. 18, highlighting its potential for bedside perfusion monitoring in cardiovascular interventions.

HSI has shown high reliability and improved sensitivity for peripheral vascular disease assessment,^605^ particularly peripheral arterial disease (PAD), which is the most common form of peripheral vascular disease caused by atherosclerosis and currently affects more than 230 million people worldwide.^606^ Sumpio et al.^607^ evaluated endothelial dysfunction in PAD patients using HSI. Skin oxygenation was measured before and after a brachial artery occlusion test using a commercial HSI system in the 500 to 660 nm wavelength range, and impaired vascular response was found in patients compared with healthy controls, illustrating HSI’s potential for early screening and disease progression tracking. Grambow et al.^536^ utilized HSI for post-procedural follow-up evaluations after surgical or endovascular revascularization in PAD patients. Not only did HSI detect oxygenation and perfusion changes that aligned with clinical improvements but it also demonstrated resilience to external factors such as temperature and physical activity. In another study, Kleiss et al.^608^ found that HSI outperformed angiography and thermal imaging for post-procedural monitoring of PAD, particularly in predicting clinical outcomes, and Ma et al.^609^ demonstrated that HSI-based perfusion monitoring at home was feasible and beneficial for PAD patients after endovascular therapy. Chiang et al.^610^ employed HSI to track peri-wound and foot oxygenation on postoperative days 1, 3, and 5 in patients undergoing infrainguinal bypass surgery, a surgical technique that can be utilized to treat PAD. HSI detected improved perfusion in all patients, despite no significant improvement in wound healing was observed.

HSI combined with a dual-wavelength difference method was employed in an *ex vivo* analysis of porcine carotid arteries to evaluate graft function through hemoglobin concentration and blood flow dynamics, with 625 and 770 nm identified as the optimal wavelengths for accurate hemoglobin measurement.^298^ In another *ex vivo* study, HSI offered detailed, dynamic, and label-free visualization and detection of thrombus formation inside blood pumps, potentially reducing pump failure or embolic complications in patients supported by devices such as left ventricular assist devices or an extracorporeal membrane oxygenation machine.^611^ These studies showcase the vast exploration of HSI application in cardiovascular procedures and mechanical circulatory support.^611^

Furthermore, HSI has demonstrated usefulness for cardiac tissue characterization, particularly in radiofrequency ablation lesion detection. Muselimyan et al.^612^ and Gil et al.^369^ demonstrated the use of HSI for detecting radiofrequency ablation lesions in atrial tissue, aiming to improve visualization during atrial fibrillation treatment. Key spectral changes related to NADH loss and increased scattering were identified. By applying autofluorescence spectral unmixing in the 420 to 720 nm range, they successfully delineated ablated versus unablated tissue, improving lesion visualization beyond standard white light and UV imaging. The lesion boundaries and depths were accurately identified, with strong correlation to histological staining (R=0.99). Building on this work, Asfour et al.^364^ later identified optical bands (480 to 538 nm) that optimized radiofrequency ablation lesion visualization while maintaining over 80% classification accuracy using just four spectral bands. Furthermore, Guan et al.^447^ achieved 94% lesion detection accuracy using unsupervised k-means clustering with four-feature grouping. Swift et al.^613^ extended this research to real-time *in vivo* identification of myocardial scars (collagen deposition, 560 to 650 nm) and radiofrequency ablation lesions (NADH fluorescence loss, 420 to 500 nm) and validated the approach in live pigs.

#### 4.3.5 Diseases of the gastrointestinal tract

HSI has been widely explored for diagnosing various gastrointestinal diseases. By capturing spatial and spectral information, HSI enables precise GI tissue characterization, perfusion assessment, and early detection of GI pathological changes as tissues may appear visually similar under white light and differences may be missed by traditional imaging. From esophageal and gastric imaging to colorectal surgery and small intestine assessment, HSI has demonstrated its potential to enhance endoscopic detection and assist in monitoring gastrointestinal conditions. The following studies highlight key advancements in applying HSI for diseases of the gastrointestinal tract.

To reduce complications during endotracheal intubation, Nawn et al.^614^ explored differentiating tracheal and esophageal tissues through HSI by identifying unique spectral profiles in a pig model. In 2020, Grigoroiu et al.^194^ tested an HSI endoscope with CNN online classification capabilities in *ex vivo* pig esophagus samples as well as human esophagus biopsies, where preliminary classification among normal gastric tissue, normal squamous epithelium, Barrett’s esophagus, and adenocarcinoma was achieved.

Targeting gastric tissues, Gu et al.^363^ developed a flexible hyperspectral gastroscope system based on filter wheels and acquired *in vivo* images of human stomach mucosa. Then, an image entropy-based band selection was carried out to choose the three most useful bands, which were used to form a new RGB image for an enhanced display of the gastric ulcer. Barberio et al.^615^ measured serosal tissue oxygenation in pig models using HSI during the gastric tubulation as a part of a novel hybrid method of ischemic gastric preconditioning aimed at improving gastric conduit perfusion and reducing anastomotic leak rates after an esophagectomy.

Moving on to colon, Yoon et al.^423^ evaluated their hyperspectral endoscope to enhance the detection and resection of colonic polyps, aiming to reduce the incidence of interval cancers between colonoscopy screenings. Images of three tissue types in seven patients undergoing colonoscopy screening were acquired. The study employed SAM to differentiate between normal mucosa, polyps, and post-resection tissue. In addition, a kNN algorithm was trained for real-time classification of tissue types based on hyperspectral data. Okamoto et al.^616^ investigated the use of HSI combined with deep learning to improve surgical outcomes in complete mesocolic excision by differentiating among colon, mesenteric tissue, and retroperitoneal tissue. Conducted on 20 pigs, the study recorded intraoperative HSI data of the sigmoid colon and its surrounding tissues. A CNN was trained to classify the tissues, achieving an overall recognition sensitivity of 79% for retroperitoneal tissue and 86% for colon and mesentery. The findings suggest that HSI and CNNs could enhance tissue identification during surgery, potentially reducing complications.

Finally, two studies by Stergar et al.^20^^,^^617^ demonstrated HSI as a noninvasive tool for diagnosing and monitoring peritoneal inflammation and fibrosis in mouse models. The first study applied HSI to assess peritoneal fibrosis, using BVF and scattering characteristics to distinguish between healthy and fibrotic tissues.^20^ The second study utilized HSI to map vascular oxygenation in peritonitis, revealing significant differences in oxygenation patterns between healthy and diseased tissues.^617^

#### 4.3.6 Hepatic disorders

HSI has been investigated extensively for characterizing thermal effects and detecting metabolic changes in liver diseases. Studies have demonstrated HSI’s potential as a noninvasive, real-time tool for differentiating tissue states and quantifying biochemical changes in hepatic disorders.

To start, HSI has been employed to characterize thermal effects in liver tissues following radiofrequency ablation. Two studies on *ex vivo* bovine liver samples demonstrated HSI’s ability to differentiate normal, thermally affected, and ablated liver regions using machine learning approaches, initially highlighting the importance of key RNIR bands.^443^^,^^505^ Subsequent analyses from both research groups refined the spectral range, yet all identified optimal bands fell within the 600 to 640 nm range.^618^^,^^619^ Notably, this aligns with the findings of an earlier *in vivo* study by De Landro et al.,^620^ where HSI detected spectral changes in ablated tissues in a porcine liver model, particularly an increase at 630 nm attributed to methemoglobin absorption.

**Fig. 19:**
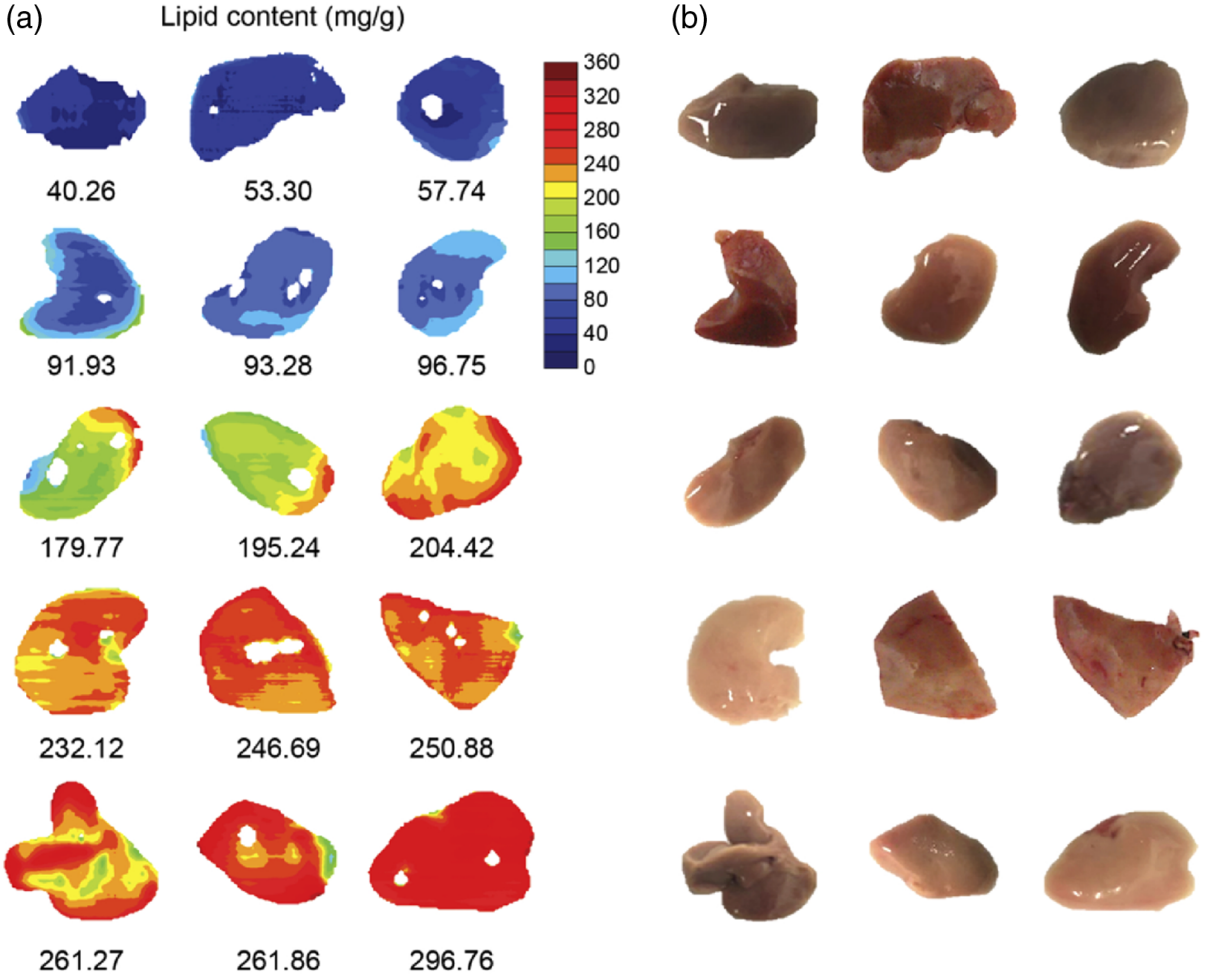
Visualization of the lipid content in mouse liver: (a) Quantitative mapping of the lipid content in the liver samples. The numbers on the right indicate the scale of the lipid content in the corresponding colors, and the values below the images indicate the measured lipid content. It is to be noted that the white regions were excluded from the region-of-interest upon SVR processing because of pixel saturation or artificial noise. (b) Visible images corresponding to the quantitative mapping shown in panel (a).^386^

Beyond liver ablation applications, HSI has been explored as a diagnostic tool for metabolic liver diseases, particularly nonalcoholic fatty liver disease, steatohepatitis, and liver cancer. Okubo et al.^386^ and Mori et al.^621^ used NIR HSI to quantify lipid content and analyze fatty acid distribution in *ex vivo* mouse livers, as shown in Fig. 19. Their studies demonstrated that HSI not only quantified lipid accumulation in fatty liver but also detected metabolic shifts across dietary conditions and disease progression. Importantly, the findings of Mori et al.^621^ revealed distinct lipid profiles in tumor-bearing liver tissues, suggesting that HSI could be used for early metabolic screening of liver cancer.

#### 4.3.7 Burn and wound

Accurate assessment of burns and wounds is essential for guiding treatment decisions and improving patient outcomes. Traditional evaluation methods, such as visual inspection and clinical scoring, are often subjective and may fail to reliably distinguish among different severities of burns or stages of wound healing.^300^ HSI has emerged as a valuable noninvasive technique that provides quantitative and spatially resolved data on tissue oxygenation, hemoglobin concentration, and perfusion, enabling a more objective assessment. HSI has been successfully applied in various aspects of burn and wound management, including burn depth assessment, wound healing monitoring and prediction, and validation of treatment efficacy. By providing real-time physiological insights, HSI significantly advances burn and wound care, facilitating earlier diagnosis, personalized treatment, and improved patient outcomes.

First, HSI was extensively validated in animal models and phantoms, providing foundational insights into its potential for burn and wound assessment. Ghassemi et al.^255^ introduced a PHSI system, which combined Stokes polarimetry and spatial frequency domain imaging for assessing hypertrophic scars on a porcine model. Calin et al.^313^ first evaluated the use of HSI combined with mixture-tuned matched filtering classification for analyzing an open wound in the leg of a dog. Then, they implemented the characterization of burn wounds in a human patient using HSI with linear spectral unmixing.^337^ Chin et al.^622^ measured the change of perfusion and oxygenation of skin with different depths of burn in an animal model. HSI revealed a significant increase in total hemoglobin in intermediate-dermal burns, a reduced increase in deep-dermal burns, and a decrease in perfusion in full-thickness burns. Paluchowski et al.^448^ used an unsupervised spectral-spatial segmentation method with HSI to discriminate burn wounds of varying severity in a porcine model. Hyperspectral images were recorded over an 8-h period to track dynamic changes. An increase in blood absorption was observed in level 2 burns, indicating inflammation, whereas a decrease in blood absorption was seen in level 4 burns, suggesting vascular damage. Meanwhile, both level 2 and level 4 burns displayed an increase in water absorption. Wahabzada et al.^450^ explored HSI wound monitoring in a 3D wound phantom made of a collagen matrix. With an unsupervised clustering method, HSI differentiated between normal and delayed wound healing conditions. Wang et al.^300^ used a NIR HSI system (950 to 1650 nm) with a machine learning ensemble regression model for quantitative burn depth assessment. Graded burn depths from superficial to full-thickness burns were created on the abdomen of a pig model and confirmed via histological analysis, and the proposed method achieved an average relative error of 7% compared with the histology gold standard.

Although animal models have provided valuable insights into the capabilities of HSI for burn and wound assessment, clinical studies on human patients have further demonstrated its effectiveness in real-world scenarios, spanning applications such as burn depth classification, wound perfusion and healing tracking, and infection detection.

HSI has been extensively applied in burn depth classification, leveraging spectral differences in tissue oxygenation and hemoglobin distribution. Parasca et al.^446^ introduced a hyperspectral index-based metric named skin burn spectral index for assessing burn depth. Though images were acquired in the range of 380 to 800 nm, the proposed method exploits spectral differences at 556 and 760 nm and had a similar performance when compared with laser Doppler imaging. Later, Parasca and Calin^144^ expanded on previous classification efforts by applying SVM classification to generate burn depth maps of class 1 to class 4 burns in 14 patients, achieving an overall accuracy of 95.99%. Schulz et al.^401^ created a different burn index based on HSI-derived hemoglobin volume and oxygenation levels, successfully predicting the necessity for skin grafting. The study involved 49 adult patients with second- and third-degree burns, with HSI imaging performed at 24, 48, and 72 h post-injury to evaluate microcirculatory changes. The study found that an increasing burn index over 72 h reflected changes in microcirculation, and by applying a burn index threshold, they predicted the need for a skin graft with 92% sensitivity and 71% specificity.

Meanwhile, McCarthy et al.^623^ used visible-light HSI to assess burn depth in a patient with second- and third-degree burns, capturing changes in ${\mathrm{HbO}}_{2}$ and HHb over the first 5 days post-injury. Their findings revealed significant perfusion changes between 6.5 and 39.3 h post-injury, suggesting its utility for guiding prompt surgical intervention. In two studies of Promny et al., a commercial VIS-NIR HSI system was used to assess burn depth in patients, with one study analyzing 97 burns on upper extremities^624^ and another capturing 118 HSI recordings of burns and healthy skin on hands.^625^ Their findings confirmed HSI’s effectiveness in differentiating burns from normal skin, though its ability to distinguish finer burn depth variations remained a challenge. These studies demonstrate HSI’s role in noninvasive, quantitative burn assessment, providing detailed spatial classification of burn injuries. Beyond burn classification, HSI has been used to predict burn healing potential, assisting in determining the need for surgical intervention.

Meanwhile, HSI has proved useful in wound healing monitoring and prediction. Hypertrophic scars, most commonly caused by burns, develop during the fibroproliferative and remodeling phase of wound healing.^255^ Liu et al.^43^ developed a multiview hyperspectral topographic system for cutaneous wound oxygenation imaging. Kulcke et al.^626^ employed a commercial HSI system to measure perfusion parameters, particularly of transplanted flaps as well as chronic wounds such as ulcers and diabetic foot wounds. Ghassemi et al.^255^ combined polarimetric imaging and HSI in a custom system for an objective evaluation of hypertrophic scars. Specifically, HSI provided quantitative chromophore mapping, revealing a higher hemoglobin concentration, elevated water content, and variable melanin distributions in scars. Zhang et al.^627^ integrated HSI, laser speckle imaging, and thermographic imaging to monitor oxygenation, perfusion, and inflammation-related heat changes, respectively. Validated through an occlusion experiment in a healthy subject’s upper extremity and tested on a 3 mm clinical wound on a subject’s lower extremity, the system tracked dynamic changes in tissue oxygenation and perfusion during healing. Marotz et al.^299^ presented a five-layer tissue model for HSI to estimate perfusion parameters in skin and wounds. The model-based analysis showed improved accuracy in distinguishing between superficial and deep layers and was effective for monitoring wound healing dynamics. Later, they advanced the model by integrating 3D perfusion analysis within the first 72 h of injury and showcased the potential of HSI in guiding early treatment decisions.^535^ Bjorgan et al. introduced an inverse photon transport model for HSI^301^ and used it together with unsupervised clustering and PCA to characterize re-epithelization in an *in vitro* human tissue wound model over 22 days.^532^ These studies collectively demonstrate HSI’s capability to provide quantitative insights into wound healing progression, making it a valuable tool for noninvasive wound monitoring. More recently, HSI has been integrated with machine learning techniques to enhance infection detection in wounds. Ramirez-GarciaLuna et al.^628^ utilized a smartphone-compatible HSI device combined with PCA-kNN clustering for automated wound infection detection. The method achieved 100% sensitivity and 91% specificity for infected wounds, along with 85% sensitivity and 77% specificity for inflamed wounds and 94% sensitivity and 70% specificity for noninflamed wounds. These findings highlight HSI’s potential to enhance infection diagnosis and reduce subjectivity in clinical assessments.

Recent studies have explored HSI’s role in validating novel wound treatment strategies, particularly cold atmospheric plasma (CAP) treatment^629^^–^^632^ and concurrent optical- and magnetic-stimulation therapy,^633^ for enhancing wound healing. For example, Rutkowski et al.^629^ employed HSI to evaluate the impact of CAP on wound healing in patients. The study found that CAP improved perfusion and increased tissue oxygenation, as confirmed by HSI, indicating accelerated healing. Similarly, Schmidt et al.^630^ used HSI in a murine model to demonstrate CAP’s effects on wound oxygenation and perfusion, revealing significant increases in superficial and deep oxygen saturation as well as reduced edema. In their following study^631^ that further investigated CAP’s molecular impact, HSI was employed to confirm enhanced tissue oxygenation and microcirculation. Similar findings of improved deep tissue oxygenation and reduced edema were reported in another human study,^632^ where 10 patients’ wounds were monitored by HSI to validate the efficacy of CAP, showing the potential for HSI to be implemented in patient treatment.

#### 4.3.8 Diabetes

By capturing detailed spectral information across different wavelengths, HSI provides a noninvasive means to analyze skin tissue and detect early signs of diabetic complications such as diabetic foot ulcers (DFU), diabetic retinopathy (DR), and diabetic neuropathy. This advanced imaging technique offers a unique capability to visualize and quantify subtle biochemical changes associated with diabetes, enabling more accurate diagnosis and continuous monitoring. For example, Sheen et al.^634^ used SWIR HSI to examine the feet of patients with type 2 diabetes mellitus for diabetic small-fiber neuropathy assessment. They found that HSIs could identify significant differences in the spectral waveforms between the patients with normal and abnormal sudomotor function, which traditional clinical examinations failed to distinguish.

**Fig. 20:**
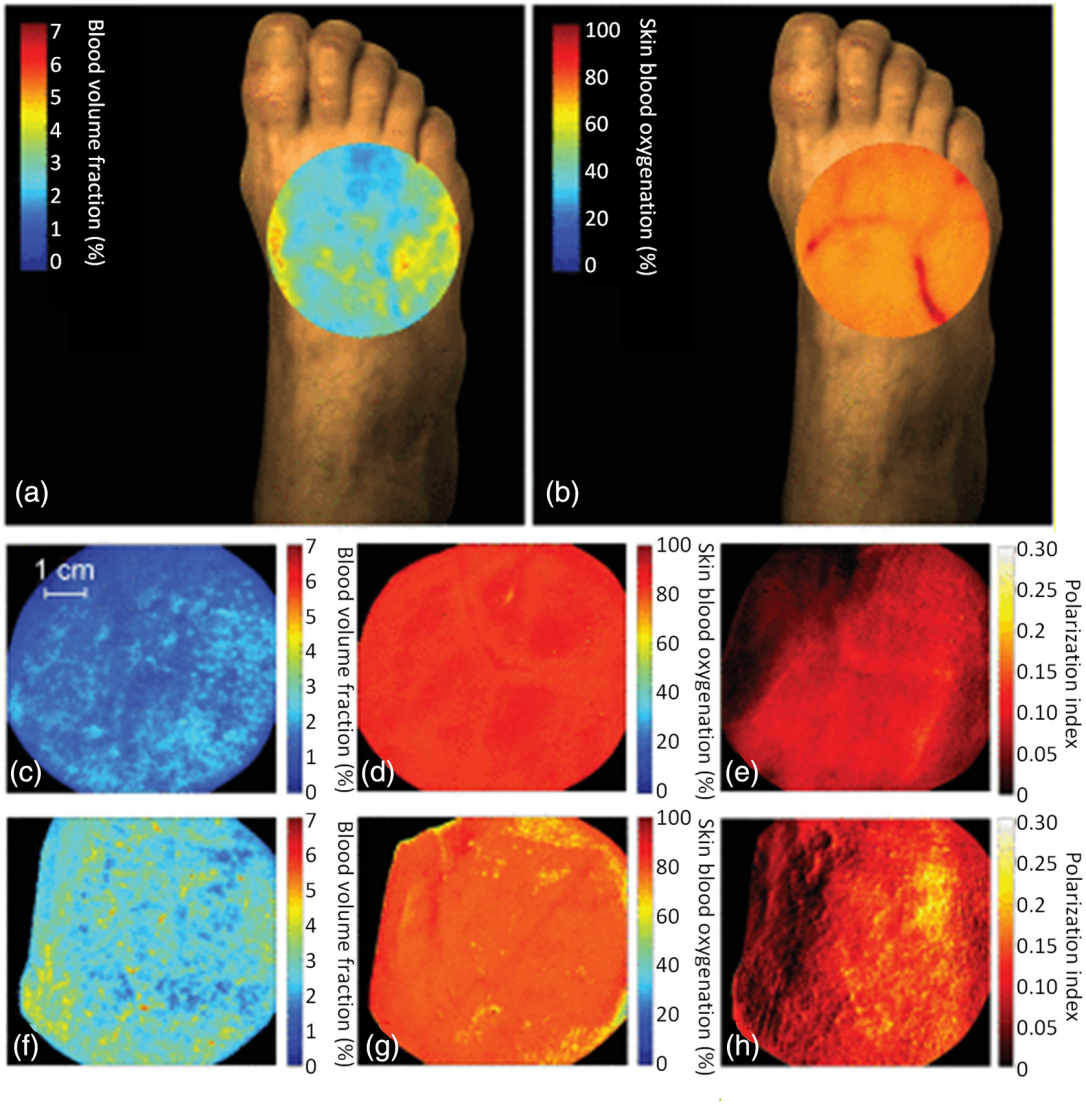
Handheld polarized hyperspectral system is capable of reliably visualizing the BVF and skin blood oxygenation parameters in the dorsal surface of the feet. (a), (b) FOV of the HSI system ($8\times 8\;{\mathrm{cm}}^{2}$) with the calculated parameters of (a) BVF and (b) skin blood oxygenation. (c)–(h) Typical reconstructed maps of the BVF (c), (f), skin blood oxygenation (d), (g), and polarization index (e), (h) for the control [upper row, (c)–(e)] and diabetic [lower row, (f)–(h)] groups, correspondingly. The figure was reproduced with permission from Ref. 154.

For the assessment of diabetic skin complications, Lee et al.^538^ evaluated two handheld HSI devices, whereas Dremin et al.^154^ developed and evaluated their own handheld PHSI system (Fig. 20). Feng et al.^49^ combined HSI with laser speckle contrast imaging to monitor changes in blood flow and blood oxygen levels in diabetic mice. One of the most common diabetic skin complications is DFU, where HSI has been investigated for healing predictions. Jeffcoate et al.^635^ used HSI to measure hemoglobin oxygenation, to predict the healing of DFU. A strong correlation was found between the oxygenation measured by HSI and blood gas analysis in an *in vitro* experiment, and a strong positive correlation was also reported between the HSI-measured oxygenation and the healing time of ulcers in 43 patients. In a following study by Yang et al.,^14^ PCA was applied to hyperspectral data to improve the sensitivity of the prediction of healing. López-Moral et al.^399^ predicted DFU healing in 21 patients using HSI and noninvasive vascular techniques. At 24 weeks, the sensitivity and specificity of prediction using oxygenation measured by HSI were 93% and 71%, whereas those using transcutaneous oxygen pressure were 91% and 100%, respectively. Later, Kounas et al.^403^ monitored 27 patients with DFU using a portable hyperspectral camera over the course of four visits with a 3-week gap between each. Not only did they find an inverse correlation between the ${\mathrm{HbO}}_{2}$ levels at early visits and ulcer size reduction, but they also achieved 85% sensitivity and specificity for the prediction at the second visit.

Hyperspectral images of the retinal microvasculature can be used to identify diabetes, specifically DR. Kashani et al.^636^ measured blood oxygen content in human retinal arteries and veins. They found a significantly lower arterial and higher venous oxygen saturation in proliferative DR patients compared with controls. Tayyari et al.^587^ assessed retinal blood oxygen saturation in patients with nonproliferative DR compared with healthy controls. A correlation between the arterial blood flow and arterial oxygen saturation was found in both the healthy and DR cohorts. In addition, Yao et al.^637^ took RGB ophthalmoscopic images and constructed hyperspectral images, from which oxygenation maps of the arteries and veins were created. It was found that as DR progressed, the oxygen utilization of retinal tissues decreased, resulting in increased oxygen saturation in the veins. Based on the spectral characteristics and oxygen saturation of the arteries, they could identify normal cases, background DR, pre-proliferative DR, and proliferative DR. Wang et al.^392^ used the same HSI construction method and separated arteries and veins using spectral data and PCA. The results showed that HSI effectively identified changes in reflectance associated with DR progression, with sensitivity values for distinguishing arteries and veins decreasing as DR severity increased.

#### 4.3.9 Dentistry

HSI has also emerged as a powerful tool in dentistry, offering advancements in contrast enhancement and disease detection. For example, Hyttinen et al.^638^ implemented partially negative optical filters to enhance the contrast of blood vessels and prosthetic tips in hyperspectral dental images. Urban and Subhash^123^ developed a hyperspectral spatial frequency domain imaging system, which quantified gingival tissue hemoglobin volume fractions, detected dental caries and bacterial activity, and measured tooth color. Later, Urban et al. used it to assess gingival inflammation during induced gingivitis, revealing distinct oxygenation differences between healthy and inflamed gum tissue.^639^

HSI has also been widely applied in fluorescence-based dental analysis, especially for early diagnosis of tooth demineralization and dental plaque as traditional visual inspection methods are naturally subject to individual interpretation and has limitations to the visual field.^640^^,^^641^ El-Sharkawy and Elbasuney^642^ used HSI to study the fluorescence spectra of dental caries induced by a 488 nm laser and a 514 nm laser. Gawad et al.^643^ used HSI to analyze fluorescence signals from 12 pre-diagnosed molars and premolars induced by a 395 nm laser. The study identified distinct wavelengths for abnormalities such as root calculus, white spots, dentin caries, and enamel caries, allowing for clear differentiation between normal and abnormal dental tissues. Roberts et al.^644^ quantified mineral loss in enamel using hyperspectral fluorescence imaging, demonstrating a strong correlation with transverse microradiography results and a slightly weaker correlation with quantitative light-induced fluorescence. In addition, Yan et al.^15^ employed hyperspectral microscopy to analyze the fluorescence spectra of three bacterial species associated with dental plaque. They identified key spectral bands (500 to 510 nm and 635 nm) that enabled differentiation of bacteria based on spectral intensity ratios (510/635  nm and 500/635  nm), achieving ∼99% sensitivity and specificity.

#### 4.3.10 Sepsis

Sepsis is a potentially life-threatening condition characterized by a drastic immune system response and severe microcirculatory dysfunction, making early and accurate detection critical for improving patient outcomes. For example, septic shock patients are commonly found with tissue hypoxia caused by the heterogeneity of microvascular blood flow.^181^ HSI offers a noninvasive, real-time approach to assessing tissue oxygenation and perfusion, providing valuable insights into disease severity and guiding hemodynamic management in septic patients.

Kazune et al.^181^ employed HSI to measure skin oxygen saturation in 30 septic shock patients at baseline and after the increase in mean arterial pressure. They noticed a higher skin oxygen saturation value in the latter condition. In another study, they found that higher mottling scores in septic shock patients correlated with lower microcirculatory oxygen saturation measured by HSI, indicating that HSI provides a more precise assessment of peripheral oxygenation and better predicts 28-day mortality than the mottling score alone.^395^ Dietrich et al.^383^^,^^384^^,^^645^ also carried out a series of studies evaluating an HSI system for bedside monitoring skin microcirculation in patients with sepsis. Oxygenation, perfusion, and water content were measured by HSI alongside standard hemodynamic monitoring. In sepsis patients, HSI revealed lower oxygenation and perfusion index as well as higher tissue water content compared with healthy volunteers, correlating with disease severity and organ dysfunction.^384^ They suggested that the HSI system could monitor microcirculatory perfusion and hemodynamic changes noninvasively without observer variability, thus showcasing promise for easy integration of the system into perioperative and critical care hemodynamic management processes.^645^

#### 4.3.11 Plastic and reconstructive surgery

In plastic and reconstructive surgery, tissue viability is often difficult to assess visually, and flap failure can lead to major complications.^646^ HSI aims to address the critical need for objective, real-time perfusion assessment to guide surgical planning, monitor postoperative recovery, and support timely interventions. This section includes both preoperative and postoperative applications of HSI in plastic and reconstructive surgery, excluding intraoperative use, which is discussed separately in Sec. 4.4.6. Most studies included here are prospective in design.

Preoperatively, HSI has been explored for its role in vascular evaluation and perforator detection. Linek et al.^647^ used a commercial HSI system during the modified Allen’s test (MAT) to quantify perfusion dynamics in the hand, where HSI-derived parameters reflected occlusion and reperfusion changes. Heimes et al.^648^ used the same system to measure collateral perfusion in the hand prior to free flap harvesting. HSI-derived parameters showed a strong correlation with the MAT results while also identifying false-negative and false-positive findings by the MAT. The results of both studies supported the use of HSI as an objective and reproducible tool for enhancing preoperative vascular assessment. Goetze et al.^649^ investigated the feasibility of HSI for preoperative perforator mapping during microvascular flap planning, and HSI was able to detect 97% of the perforators confirmed by Doppler ultrasound. However, in another prospective comparison study, Nischwitz et al.^650^ reported limited utility of HSI for perforator localization, especially when compared with thermal imaging, which showed significantly higher sensitivity.

Postoperatively, many studies have shown that HSI can detect perfusion deficits earlier than conventional methods. In a preclinical mouse study, Chin et al.^651^ demonstrated that high HHb intensity measured by HSI 30 min postoperatively had a strong linear correlation with necrosis observed on postoperative day 7. This provided compelling evidence that HSI can noninvasively and quantitatively predict early cutaneous flap necrosis. In addition, several prospective clinical studies have confirmed HSI’s ability to detect compromised flaps earlier than other methods.^400^^,^^652^^,^^653^ For example, Kohler et al.^652^ conducted a prospective observation cohort study monitoring free flaps from various soft tissue reconstructions for early detection of flap compromise. The ${\mathrm{StO}}_{2}$ and PI measured by HSI accurately predicted all flap failures prior to clinical detection or the Doppler ultrasound. Furthermore, Schulz et al.^654^ carried out a retrospective study evaluating the diagnostic accuracy of HSI, particularly a HSI-derived tissue THI, for detecting venous congestion following free flap surgery. Their results indicated that HSI had a higher sensitivity than clinical assessment in the first 24 h post-op, although its performance was comparable between 48 and 72 h.

#### 4.3.12 Oxygenation, microcirculation, and vascular mapping

HSI offers powerful, noninvasive means to visualize and quantify oxygenation, perfusion, and microvascular dynamics in superficial tissues. This application is not tied to a specific disease but has broad utility in understanding microcirculatory function and detecting peripheral tissue hypoxia—factors relevant to the diagnosis, monitoring, and prognosis of various clinical conditions, such as diabetes, cardiovascular disease, trauma, and sepsis. This section highlights studies using HSI to visualize tissue oxygen saturation, map vascular architecture, and track microcirculation changes.

To diagnose and monitor various diseases through cutaneous oxygenation measurement, Miclos et al.^597^ developed an algorithm based on the modified Beer–Lambert law and nonlinear least squares optimization for estimating oxygenated, deoxygenated, and total hemoglobin, as well as oxygen saturation and scattering. Considering the potential tissue infarction caused by prolonged vasoconstriction after anesthesia injections, Thiem et al.^655^ measured dynamic oxygen saturation from human volunteers’ arms to evaluate the impact of Articaine and epinephrine injections on cutaneous perfusion. Baranoski et al.^656^ examined human skin responses and reported a decrease in hemoglobin concentration associated with increasing anemia severity. To evaluate the effect of water-filtered infrared-A irradiation, Thomsen et al.^657^ employed HSI to assess the oxygenation status of normal skin and superficial tumors in patients. In another study, Dietrich et al.^658^ investigated the use of HSI for evaluating microcirculatory quality during hemodynamic therapy. Hemorrhagic shock was surgically induced in porcine models, and HSI was used to monitor changes in tissue parameters in both the skin and kidney. The results demonstrated significant decreases in HSI-derived oxygen saturation and PI in both organs following shock induction, with a moderate correlation observed between them.

In addition to measuring oxygenation levels, HSI enables detailed spatial mapping of microvascular structures and their dynamic responses to hypoxic conditions or therapeutic interventions. Hamza et al.^659^ proposed a method for visualizing cutaneous blood vessels using HSI-derived three-wavelength indices, resulting in up to 25% increase in contrast ratio. Holmer et al.^660^ proposed algorithms to map tissue parameters in hyperspectral images, including ${\mathrm{StO}}_{2}$, NIR PI, THI, and TWI, which have been extensively utilized by numerous studies for predictive purposes or intraoperative guidance.

To improve monitoring of microcirculation and tissue oxygenation, Feng et al.^48^ accurately mapped oxygen saturation in cutaneous microvessels using simulated hyperspectral data with a lookup-table-based inverse model. Meanwhile, Wang et al.^147^ analyzed vascular oxygenation levels and obtained blood oxygenation maps from *in vivo* hyperspectral images of the dorsal skin fold window chamber in mice. Lucas et al.^451^ used HSI to study dynamic oxygenation and vessel morphology changes during hypoxia and reoxygenation in a hamster model, showing that wide-field HSI surpasses limitations of traditional intravital microscopy by enabling analysis of large microcirculatory networks. Moving on to human studies, Van Manen et al.^161^ used a snapshot HSI camera to quantify cutaneous ${\mathrm{StO}}_{2}$ in human volunteers during occlusion–reperfusion experiments and after vasodilation induction. As a result, HSI detected significant ${\mathrm{StO}}_{2}$ variations during occlusion but no significant changes due to vasodilation. Meanwhile, Mahmoud and El-Sharkawy^661^ implemented a phase analysis method to identify the location of veins and arterioles as well as to enhance the visualization of blood perfusion in hyperspectral images.

#### 4.3.13 Other nonmalignant conditions

Beyond its well-established applications, HSI has shown potential in various emerging fields of biomedical research. Although these applications have received comparatively less attention in the literature, they hold significant promise for future advancements. From assessing tissue characteristics, evaluating vascular health and microcirculatory function, to assisting in conditions related to trauma, lung, kidney, arthritis, and blood, HSI continues to demonstrate its versatility in providing detailed spectral insights that traditional imaging methods often lack.

First, several studies focused on the systematic spectral profiling and differentiation of tissue types in hyperspectral images. Studier-Fischer et al.^354^^,^^662^ introduced a publicly available, annotated dataset featuring thousands of hyperspectral images from 20 physiological organs in pigs. Organ type was found to be the dominant source of spectral variation compared with inter-individual and acquisition condition variability, supported by classification accuracy >95% among different tissue types using a DNN. It shows the potential of HSI being used for automatic tissue identification and context-aware surgical system navigation. Aref et al.^279^ investigated HSI in both transmission and reflection modes in *ex vivo* heart, liver, and kidney tissues, where they identified the optimal bands (640 to 680 nm and 720 to 760 nm) for tissue distinction. Ma et al.^256^ explored polarization properties of various *ex vivo* mouse organs using a custom PHSI system and implemented tissue classification based on the tissue spectra of DOP, DOLP, and DOCP. Targeting mucosa change examinations and potential oral cancer detection, Thiem et al.^663^ classified *ex vivo* human oral tissue samples, including fat, muscle, and mucosa, using a DNN. Aiming at automated classification of anatomical structures in microsurgery, Puustine et al.^111^ employed a hyperspectral surgical microscope to acquire a dataset of four *ex vivo* human placentas, which contained 101 hyperspectral images and detailed annotations. Pathak et al.,^436^ on the other hand, carried out *in vivo* human tissue discrimination based on spectral similarity measures. Moreover, Meng et al.^664^ developed an HSI cryomacrotome, where high-resolution fluorescence volumes of a whole mouse could be acquired. The preliminary testing results showed distinct autofluorescence spectra of brain, liver, and bowels in the 500 to 720 nm range, which is promising for analysis of labeled cells, metastatic spread, and drug biodistribution.

Next, HSI has been actively used in blood composition and hemodynamic studies. Li et al.^106^ used a snapshot hyperspectral camera to acquire transmission data from human volunteers’ fingers, from which they extracted photoplethysmography signals and predicted concentrations of hemoglobin, platelets, and bilirubin. Khatun et al.^303^ detected methemoglobinemia in rats by measuring blood chromophores, including methemoglobin, ${\mathrm{HbO}}_{2}$, and HHb *in vivo*. Aref et al.^440^ developed a custom HSI system in the VIS-NIR wavelength range to visualize arm blood vessels and assist phlebotomy. They first identified 460 and 750 nm for oxygenated arteries and deoxygenated veins. Then, using k-means clustering, vessel segmentation was achieved to improve venous access. Focusing on the potential application for COVID-19 diagnosis, El-Sharkawy et al.^438^ mapped subcutaneous blood circulation by analyzing ${\mathrm{HbO}}_{2}$ and HHb distribution using an HSI-based diffused reflectance imaging system.

In addition to subcutaneous detection of COVID-19, Gomez-Gonzales et al.^387^ conducted a proof-of-concept study demonstrating the potential of hyperspectral reflectance imaging for reagent-free primary screening of SARS-CoV-2 at the point-of-care. A combination of spectral feature descriptors, PLS-DA, and AI was used for the spectral analysis of 72 nasopharyngeal exudate samples across a range of viral loads and 32 fresh saliva samples. The model achieved 100% sensitivity and 87.5% peak specificity, even distinguishing PCR-negative but symptomatic cases.

In musculoskeletal and orthopedic care, common imaging modalities for diagnoses often rely on monochromatic light, missing details in the spectral information of biochemical changes.^665^ HSI, by capturing those tissue-specific spectral signatures, offers a more comprehensive approach for earlier detection of articular pathology, particularly in rheumatoid arthritis, where timely diagnosis is essential for guiding tailored treatments due to variable clinical presentation^666^ and preventing long-term functional loss.^667^ Two articles by Milanic et al.^297^ and Dolenec et al.^665^ targeted rheumatoid arthritis by looking at human fingers and explored the feasibility of using HSI for noninvasive early detection. Milanic et al.^297^ first modeled hyperspectral transmittance and reflectance in human finger joints using Monte Carlo simulations to identify the optimal imaging parameters for arthritis detection. Their results indicated that HSI showed distinguishable spectral patterns with reduced transmission for arthritis joints, particularly in the 800 to 900 nm and 1050 to 1100 nm windows. The findings were validated in the study by Dolenec et al.,^665^ with further analysis of the anatomically related and inter-subject spectral variability.

HSI has been explored in nephrology, offering promising advancements in the classification of urinary stones and the assessment of kidney function. Blanco et al.^334^ used NIR HSI to classify urinary stones and compared it against conventional infrared spectroscopy. Their pixel-wise classification method achieved over 90% accuracy in identifying stone components, demonstrating the potential of HSI for precise urinary stone analysis. Dietrich et al.^604^ used HSI to evaluate microcirculation changes in skin as a potential indicator of kidney dysfunction, which is a common complication of TAVI. The proposed method provided periinterventional monitoring and could possibly improve surgical outcomes.

Regarding autoimmune-related diseases, Teaw et al.^668^ evaluated the feasibility and diagnostic utility of HSI in assessing Raynaud phenomenon associated with systemic sclerosis. Patients and healthy volunteers underwent a cold water immersion challenge, during which HSI-derived ${\mathrm{StO}}_{2}$ as well as ${\mathrm{HbO}}_{2}$ and HHb were measured at their ventral fingertips and palm. HSI revealed a significantly greater reduction in ${\mathrm{HbO}}_{2}$ and ${\mathrm{StO}}_{2}$, along with slower rewarming trajectories, in systemic sclerosis patients compared with healthy controls. Chen et al.^669^ applied SWIR HSI combined with SAM to assess skin involvement in 31 systemic sclerosis patients. Spectral angle values correlated positively with clinically measured skin scores and dermal thickness and were particularly sensitive in patients who exhibited normal skin scores. Collectively, both studies suggested that HSI offers a noninvasive and sensitive tool for the monitoring of system sclerosis.

Katzenschlager et al.^670^ found HSI useful in the trauma resuscitation room for assessing microcirculatory status in trauma patients during early resuscitation. It was noted that HSI could be integrated into trauma workflows without clinically relevant delays despite a 37-s longer primary survey duration. Moreover, HSI revealed early and subtle impairments in tissue perfusion and oxygenation before visible vital signs.

Last, HSI has emerged as a valuable tool for studying lung-related diseases by providing detailed spectral information on tissue oxygenation and vascularization. Fawzy et al.^190^ integrated HSI into an endoscopic imaging system to rapidly map mucosal blood supply in the lungs during bronchoscopy. The system enabled real-time visualization of total hemoglobin concentration and tissue oxygenation levels, which are critical for detecting abnormalities such as hypoxia and early-stage lung cancer.

### 4.4 Intraoperative Image Guidance

Conventional preoperative imaging modalities such as magnetic resonance imaging (MRI) and computed tomography (CT) cannot account for dynamic changes such as brain shift that occur during surgery.^100^ Although tools such as intraoperative ultrasound and MRI offer valuable insights, they are often limited by stringent operating constraints in the clinical environment or spatial resolution.^197^ HSI offers a compelling alternative by providing real-time, high-resolution spectral data potentially without disrupting the surgical workflow.^100^^,^^197^^,^^671^^,^^672^ This capability makes HSI particularly well suited for image-guided procedures. By extending the surgeon’s visual perception beyond the visible spectrum, HSI enables enhanced tissue characterization that surpasses what is possible through direct visualization or palpation alone. In this section, we focus on HSI’s application in intraoperative image guidance. The ultimate objective is the real-time or near-real-time acquisition and analysis of hyperspectral data during surgery, where the extracted information directly informs surgical decisions, such as delineating tumor margins, preserving critical structures, assessing tissue viability, or directing surgical tools.

#### 4.4.1 Neurosurgery

Due to brain tissue being highly functional and less regenerative, it makes intraoperative decision-making in neurosurgery increasingly difficult near functional boundaries or infiltrative gliomas. This has opened an opportunity for HSI to offer a label-free, noncontact, and potentially real-time tool for surgeons. In the past 10 years, HSI has shown its versatility in neurosurgery, such as nerve detection to avoid peripheral nerve damage,^305^ hemodynamics change detection for physiological insights,^108^^,^^188^^,^^531^^,^^673^ and particularly, intraoperative brain tumor delineation.^95^^,^^100^^,^^110^^,^^128^^,^^130^^,^^162^^,^^187^^,^^343^^,^^344^^,^^425^

Noordmans et al.^188^ utilized HSI to monitor cortex oxygenation during brain surgery in an epilepsy patient, revealing the potential of HSI as an intraoperative localizing tool that can lead to insights into seizures. Moreover, HSI’s potential for intraoperative monitoring of oxygenation and hemodynamics has been further proved by more studies. Konecky et al.^115^ adopted hyperspectral optical tomography to detect rapid physiological responses in the rat cortex. For the safety and effectiveness of cerebrovascular reconstruction, Mori et al.^673^ used HSI to assess oxygen saturation change during superficial temporal artery to middle cerebral artery anastomosis procedures. Fu et al.^44^ assessed ischemic stroke in rats based on a ratio of reflectance at 545 and 560 nm bands. Pichette et al.^108^ combined a VIS snapshot hyperspectral camera with a surgical microscope for intraoperative video-rate assessment of neurovascular coupling in a patient undergoing epileptogenic tissue resection. Giannoni et al.,^531^ on the other hand, explored RNIR bands to retrieve the concentration of hemoglobin and CCO from the exposed cerebral cortex of mice.

**Fig. 21:**
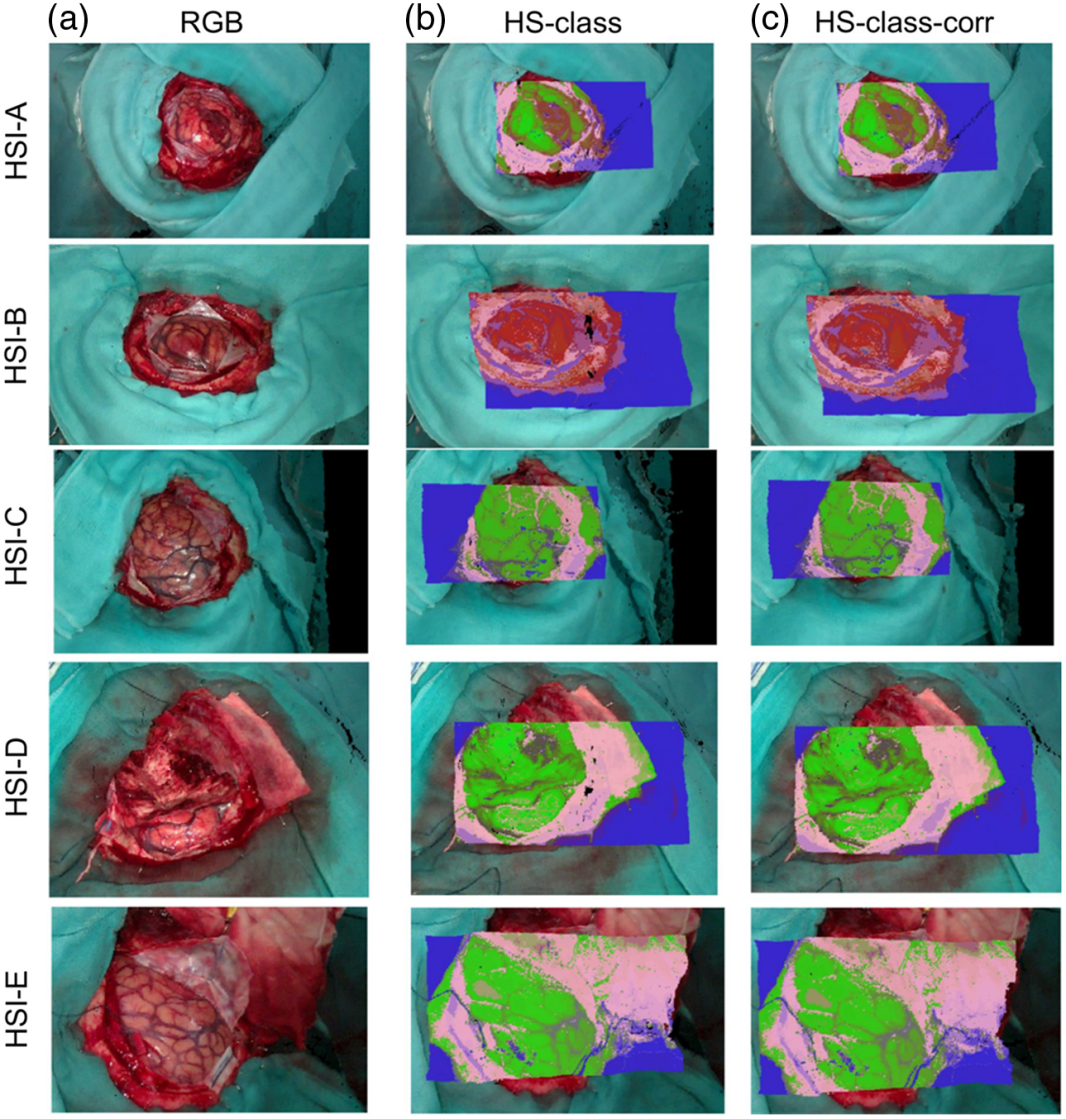
Point cloud results in real operations. Each row refers to a different tumor operation, whereas each column refers to a type of visualization. (a) RGB point cloud, (b) RGB point cloud with hyperspectral classification information, and (c) RGB point cloud with hyperspectral classification information and depth correction. Red pixels refer to tumor tissue, green pixels to healthy tissue, blue pixels to blood, and pink pixels to dura-mater.^162^

Tumor resections are one of the most common and critical procedures in neurosurgery. Accurate intraoperative detection of brain tumors is critical for maximizing tumor resection while preserving healthy tissue, and HSI provides a powerful solution that enables real-time, noninvasive tissue differentiation during neurosurgery. Fabelo et al.^130^^,^^344^ developed a surgical aid visualization system comprising a VIS-NIR camera and an NIR camera, the images of which were registered by Leon et al.^425^ for improved intraoperative brain tumor classification using the full wavelength range. The system was introduced into the brain tumor resection surgical workflow for image guidance.^137^ In a preclinical and first-in-human study, MacCormac et al.^100^ validated a bespoke HSI system, which was safely adopted into the operating room and intraoperatively evaluated on a patient undergoing the removal of a posterior fossa meningioma. Sancho et al.^162^ developed an AR real-time hyperspectral system for the intraoperative classification and display of brain tumor tissues. The system acquired hyperspectral data at 14 fps to detect tumor tissue while the surgeon operated, which was faster than many other HSI systems. Furthermore, it also overlaid the classification results with the RGB point cloud captured by the LiDAR camera, as shown in Fig. 21. Ebner et al.^197^ designed a video-rate real-time HSI system based on a snapshot hyperspectral camera and an exoscope and validated it in a spinal fusion surgery with minimal disruption of the workflow. Last, Huang et al.^274^ validated HSI with wearable AR on a phantom for potential surgical guidance during brain tumor resection.

The surgical microscope is a fundamental tool in neurosurgery, and its integration with HSI has been explored by multiple researchers in brain tumor surgeries. Giannantonio et al.^95^ coupled a spatial–spectral hyperspectral camera to a surgical microscope, and they demonstrated how HSI can serve as an intraoperative diagnostic tool in low-grade glioma surgery. Kifle et al.^187^ evaluated the feasibility of a snapshot hyperspectral camera integrated with a surgical microscope for real-time segmentation of pediatric brain tumors. Puustinen et al.^110^ reported minimal disturbance to the workflow of glioma surgery from utilizing a hyperspectral surgical microscope for intraoperative data acquisition and visualization. These studies present promising developments in merging HSI with the surgical microscope to guide surgical outcomes through quantifying surgical accuracy.

In addition to directly facilitating tumor detection using reflected light, HSI can also improve visualization of brain tumors by enhancing the contrast of fluorescence signals in fluorescence-guided surgery. Bravo et al.^57^ improved the detection limit of PpIX from 0.12  μg/mL by visual to 0.014  μg/mL using HSI, enabling the detection of subtle or non-visible PpIX fluorescence signals. Their clinical evaluation in meningioma and glioma surgeries demonstrated that HSI could enhance detection near margins during late-stage resection and suppress false positives. Lehtonen et al.^542^ investigated the limits of PpIX fluorescence detection of HSI analysis in PpIX concentration samples. Despite the analysis being *in vitro*, HSI could detect PpIX concentration as low as 0.03 to 0.15  μmol/L, more than 10 times lower compared with expert visual evaluation (0.6 to 1.8  μmol/L). Finally, Kaneko et al.^61^ used HSI for objective measurement of PpIX to determine its time course of fluorescence intensity and concentration in malignant gliomas across different intraoperative time points.

#### 4.4.2 Head and neck surgery

The human head and neck region contains a complex array of anatomical structures and diverse tissue types, making it highly beneficial to employ imaging tools capable of accurately differentiating tissues and guiding surgical intervention.

The most actively studied applications of HSI in this region are intraoperative tumor detection and surgical margin assessment. Lu et al.^292^^,^^428^^,^^552^ carried out several studies on the exposed tumor in mice, including *in vivo* tumor vascularity visualization based on spectral unmixing, investigation of the usefulness of different numbers of spectral bands, and exploring various spectral features. Targeting at replacing the time-consuming intraoperative pathologist consultation with intraoperative hyperspectral tumor margin delineation, Halicek et al.^416^^,^^458^ acquired an intraoperative hyperspectral dataset of freshly excised *ex vivo* head and neck tissue samples, where they differentiated tumor and normal tissues around the margin using an inception-based CNN. They also found that specular glares in tissue samples could degrade the classification performance.^185^ Sequentially, based on a part of the dataset, Ma et al.^478^ carried out pixel-level tumor margin detection using a fully convolutional network. Meanwhile, Brouwer de Koning et al.^555^ trained a neural network on hyperspectral images of *ex vivo* human tongue specimens specifically for margin detection in tongue cancer surgery. By combining images from the VIS and NIR cameras, they differentiated tongue tumors from normal muscle with 82.3% accuracy and 0.917 AUC, which could potentially provide more information during surgery for more precise tumor resection.

Beyond oncologic applications, HSI has also shown value for general intraoperative tissue differentiation in head and neck procedures. Wisotzky et al.^35^^,^^189^ conducted a two-part investigation validating HSI for intraoperative tissue differentiation in head and neck surgeries. Using a surgical microscope with either a monochromatic filter-wheel setup or a dual-snapshot-camera configuration, they captured spectral signatures of multiple tissue types, including nerve, parotid gland, muscle, and fat, during mastoidectomy, neck dissection, and parotidectomy. Both systems distinguished nerves from parotid tissue intraoperatively and revealed unique spectral reflectance curves of different tissues, supporting the potential of HSI for real-time, label-free guidance in otolaryngologic procedures. To further improve intraoperative guidance, recent studies have applied machine learning techniques for automated tissue recognition. Barberio et al.^329^ employed a deep model to automatically recognize nerve, artery, vein, muscle, fat, and skin in an animal model. Maktabi et al.^397^ applied SVM classification to automatically discriminate parathyroid, thyroid, and recurrent laryngeal nerve from surrounding tissues and materials in thyroidectomy. Fernando et al.^510^ employed a U-Net to segment thyroid, parathyroid, and muscle in thyroid surgery.

#### 4.4.3 Esophageal surgery

Esophagectomy, or esophageal resection, is a surgical procedure typically performed to treat esophageal cancer or other severe esophageal conditions. To restore gastrointestinal continuity after esophagectomy, reconstruction is most often achieved using a gastric conduit, or alternatively, a colon conduit when the stomach is unavailable or unsuitable. An anastomosis is then performed to connect the remaining esophagus to the conduit. However, the risk of anastomotic leakage remains high, largely due to significant disruption of the blood flow.^271^^,^^674^ Therefore, a reliable intraoperative method to quantify tissue perfusion at the conduit and anastomotic site is crucial for reducing complications and improving surgical outcomes.

A group of researchers based in Europe has conducted a series of studies to explore the clinical utility of HSI in assessing gastric conduit perfusion during esophagectomy.^271^^,^^675^^,^^676^ In a foundational preclinical study, Barberio et al.^271^ validated HSI against confocal laser endomicroscopy and capillary lactate levels to quantify tissue oxygen saturation in pig models. Not only did HSI precisely identify regions of interest with an enhanced reality tool, but it also obtained oxygen saturation measurements that correlated well with the other two modalities, demonstrating its reliable perfusion mapping capability at both serosal and mucosal levels. Köhler et al.^675^ applied HSI clinically to evaluate ischemic conditioning effects and intraoperative perfusion patterns in human gastric conduits. HSI helped distinguish between ischemically conditioned and nonconditioned gastric sleeves, without extending the operative time. Most recently, Hennig et al.^676^ performed a prospective pilot study comparing HSI and indocyanine green (ICG) fluorescence imaging in 13 esophagectomy patients, finding both modalities to be complementary and capable of guiding the anastomotic site selection. Across these studies, HSI proved to be a noninvasive, repeatable, and dye-free alternative for real-time perfusion assessment in esophageal surgery.

**Fig. 22:**
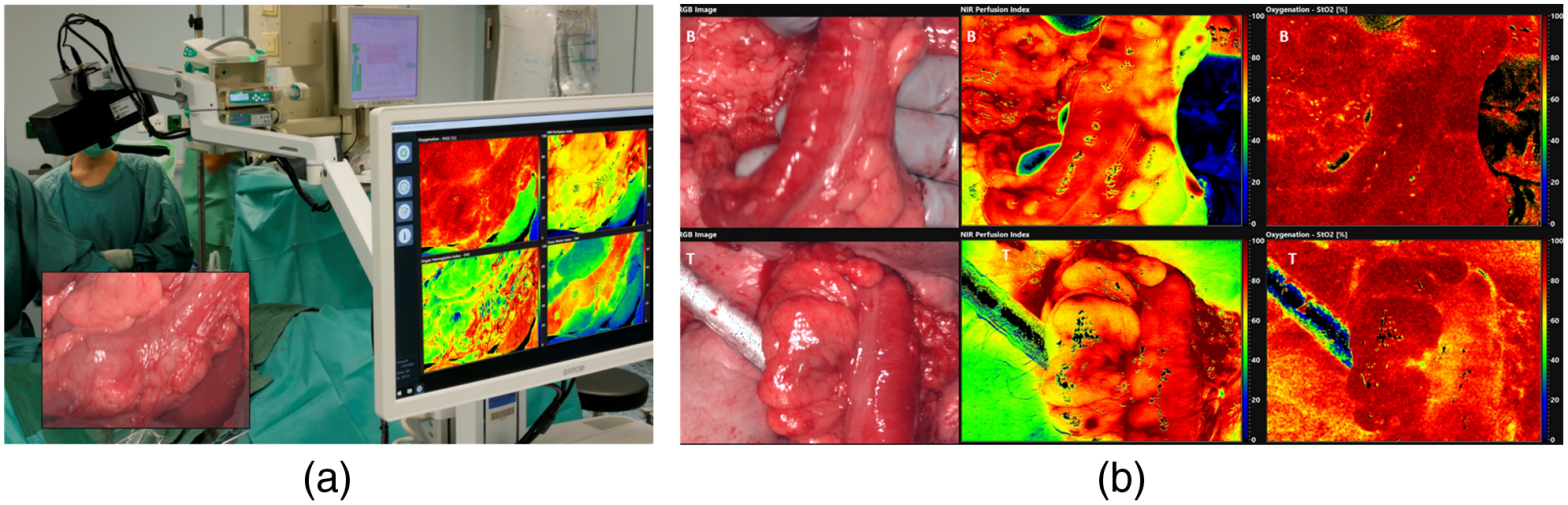
HSI system employed to evaluate perfusion of colon conduit after esophagectomy. (a) HSI system in the operative setting. (b) Intraoperative HSI-measurements of the base (B) of the colon interposition and tip (T) of the colon interposition in the cervical area. Left: HSI-synthetic RGB; middle; color-coded images of NIR perfusion index; right: color-coded images of tissue oxygen saturation.^678^

Moreover, several studies from other groups were carried out for perfusion assessment in esophagectomy. Nickel et al.^674^ employed HSI in a study aiming at optimizing anastomotic technique and gastric conduit perfusion for minimally invasive esophagectomy. Various methods of linear stapled anastomosis were simulated in a live porcine model with HSI, evaluating the oxygenation around the anastomosis site. Schwandner et al.^677^ used HSI to fast quantify oxygen saturation and perfusion of gastric sleeves prior to esophagogastric anastomosis in four patients undergoing esophageal resection. Zimmermann et al.^678^ used HSI to support surgeons selecting the optimal colon segment for the conduit and anastomotic site during surgery, as shown in Fig. 22. HSI offered visualization of the blood flow and oxygen levels before and after the form of conduit, and it was also used to confirm the adequate blood flow at the base and tip of the conduit after being moved into place.

Furthermore, in addition to esophagectomies, Vaz Pimentel et al.^679^ employed HSI in two cases of pediatric esophageal repair surgeries to check the perfusion before creating the anastomosis. Particularly in one case, real-time HSI assessment showed an impaired perfusion at the tip of the lower esophagus, leading to further esophageal removal that ensured a healthy anastomosis.

#### 4.4.4 Bowel surgery

Bowel surgery presents considerable intraoperative challenges, particularly in accurately assessing tissue perfusion, identifying viable margins, and preventing postoperative complications such as anastomotic leakage.^680^ These challenges are compounded by the limitations of subjective visual assessment of bowel tissue^402^^,^^418^ and the transient nature of commonly used perfusion markers such as ICG. As a result, there is a growing need for objective, real-time imaging tools that can guide surgeons during critical decision-making moments. Among various emerging technologies, HSI has become one of the most actively investigated modalities for intraoperative application, with bowel surgery representing a leading area of focus due to the high demand for reliable perfusion assessment.

To meet clinical needs, multiple research groups have focused on developing intraoperative HSI systems that can be integrated into the surgical workflow for both open and minimally invasive procedures. Lee et al.^99^ developed a real-time intraoperative imaging platform integrating visible light imaging, snapshot HSI, and laser speckle contrast imaging within a surgical microscope, which was tested in a rat intestinal ischemia model for perfusion monitoring. Ma et al.^155^^,^^156^ developed a dual-camera surgical microscope system and a laparoscope system, both comprising a hyperspectral camera and a color camera, and tested them on *ex vivo* tissues. With hyperspectral super-resolution reconstruction that fused images from the two cameras, they demonstrated the potential of hyperspectral intraoperative image guidance without compromising the image quality or spectral signatures. Meanwhile, Pruitt et al.^121^^,^^200^^,^^331^ developed a fast dual-hyperspectral-camera laparoscopic imaging system for minimally invasive abdominal surgery. Barberio et al.^270^ introduced an intraoperative guidance system that integrates HSI with enhanced reality to assess bowel perfusion and visualize ischemic gradients across small bowel segments. The system was validated in a porcine model during open surgery and compared with a fluorescence-based enhanced reality system.^270^^,^^681^ The results showed a significant correlation between the time-to-peak measured from fluorescence imaging and tissue oxygenation from HSI. Notably, the prediction model based on HSI for local capillary lactate levels proved to be more accurate than that derived from fluorescence-based enhanced reality. Furthermore, Thomaßen et al.^672^ tested a new HSI laparoscopic system during minimally invasive surgery and compared it with a standard HSI system in open surgery, demonstrating the great potential of intraoperative HSI for future application in minimally invasive surgery.

With technological advances paving the way, a growing number of preclinical and clinical studies demonstrated the feasibility and clinical value of HSI in bowel surgery. Recent works have highlighted its role in quantifying tissue perfusion, guiding resections, and preventing complications, thus showing promising potential for broader clinical adoption.

Mehdorn et al.^682^ investigated assessing intestinal perfusion using HSI in cases of acute mesenteric ischemia during an exploratory laparotomy. The research used intraoperative HSI data recorded from 11 patients, where various HSI-derived physiological parameters were calculated and analyzed. The results indicated significant differences in oxygen saturation and perfusion index between poorly perfused and viable intestinal segments, suggesting that HSI can effectively differentiate between ischemic and well-perfused intestine. Subsequently, they employed HSI and ICG angiography simultaneously to assess intestinal perfusion in a patient with acute mesenteric ischemia, where both methods showed comparable and reproducible performances.^683^ In a prospective study by Son et al.,^684^ both ICG angiography and HSI were employed to assess colonic perfusion status in patients undergoing laparoscopic colorectal cancer resection. HSI-derived tissue oxygen saturation levels were compared with quantitative perfusion parameters from ICG angiography to identify a safe range of ICG values indicative of adequate tissue oxygenation.

Beyond perfusion assessment, another critical application of HSI in bowel surgery is the intraoperative delineation of transection margins. Precise identification of viable tissue boundaries is essential to ensure oncological completeness and reduce postoperative complications. A series of clinical trials has demonstrated how HSI can reveal discrepancies between visually estimated margins and actual perfusion borders, allowing for real-time correction of resection lines and potentially improving surgical outcomes. In a clinical trial involving 24 patients, Jansen-Winkeln et al.^680^ evaluated the use of HSI for the determination of resection margin before and after separation of the marginal artery. In every case, HSI revealed a discrepancy between the surgeon’s intended transection line and the actual tissue boundary. In addition, the planned resection area was adjusted in five cases based on the HSI findings. A comparison between HSI and ICG angiography in 32 patients indicated that both methods had similar results in determining the perfusion border, with the former showing more differences between proximal and distal borders, whereas the latter provided a sharper border zone.^685^ In a subsequent clinical pilot study involving 52 colorectal surgery patients, HSI was employed to detect poorly perfused bowel segments prior to anastomose creation.^686^ As a result, a discrepancy larger than 5 mm between the transection lines determined by the surgeon and HSI was identified in 50% of cases. The study was further extended to 105 colorectal resections, where HSI revealed perfusion deficits and guided resection lines with added information beyond the clinical judgment in over 40% of cases.^671^ Moreover, in a prospective study by Jansen-Winkeln et al.,^687^ HSI was utilized to assess tissue oxygenation and perfusion at key points of the pouch during restorative proctocolectomy with ileal pouch-anal anastomosis. By correlating the intraoperative HSI measurements with postoperative clinical assessment, it was found that HSI could identify differences between patients with and without anastomosis leakage.

In addition to the abovementioned group leading early clinical investigations, several other teams have contributed to expanding the application of HSI in bowel surgery through innovative methodologies and algorithm development. These studies, often focused on preclinical animal models, explored advanced classification techniques and data processing strategies to enhance the diagnostic accuracy and automation potential of HSI.

Clancy et al.^50^ presented an LCTF-based HSI laparoscope designed for intraoperative monitoring of ${\mathrm{StO}}_{2}$ during surgical procedures, including colorectal surgery, and they validated the system *in vivo* during porcine abdominal surgery, where ischemic conditions were induced by occluding the mesenteric arcade. It successfully measured ${\mathrm{StO}}_{2}$ across a range of 30% to 100% with a temporal error of ±7.5%. Correlation was observed between absolute ${\mathrm{StO}}_{2}$ values from the imaging system and blood oxygen saturation measured by a clinical blood gas analyzer, highlighting its potential for real-time assessments of tissue perfusion, whereas the inter-subject variability needs to be addressed. In two studies of Zhang et al.,^357^^,^^402^ they investigated the use of HSI to automatically distinguish between normal and necrotic sites in small intestinal tissue. Using a VIS-NIR hyperspectral camera, hyperspectral images were captured intraoperatively on eight rabbits. In the first study, six supervised classification methods were tested with an artificial neural network achieving the best performance.^402^ In the second study, data analysis was implemented using unsupervised algorithms, including k-means and density peaks clustering, of which the latter was more effective in two band combinations of 500 to 662 nm and 700 to 858 nm.^357^ They also explored using transfer learning to solve the data volume limitation and inter-patient variability. The results indicated that transfer learning significantly enhanced model performance, increasing classification accuracy from below 80% to over 90%.^357^

Last, toward optical biopsy of tumor during image-guided surgery, Manni et al.^418^ investigated colon cancer detection in six *ex vivo* specimens using a CNN named HybridSpectraNet and achieved 0.82 AUC. Wagner et al.^688^ reported the first case in which HSI and ICG fluorescence imaging were used intraoperatively to guide the resection of a jejunal gastrointestinal stromal tumor intertwined with an arteriovenous malformation. HSI successfully differentiated the tumor from surrounding healthy and ischemic bowel based on real-time perfusion and metabolic parameters, whereas ICG visualized the vascular anomaly and facilitated margin planning.

#### 4.4.5 Hepato-pancreato-biliary surgery

Hepato-pancreato-biliary (HPB) surgery encompasses procedures involving the liver, pancreas, and biliary system, which are anatomically interconnected and often share vascular and functional relationships, making them clinically grouped in both surgical and oncological care. HSI has gained increasing attention in HPB surgery for its ability to noninvasively assess tissue oxygenation, perfusion, and composition in real time, especially when procedures may include multiple organs and more complex anatomical structures. By providing quantitative, spatially resolved information across a broad spectral range, HSI supports critical intraoperative decisions that are often limited by the subjective interpretation of visual cues alone.

First, HSI has shown promise in intraoperative tissue characterization and tumor delineation, both of which are essential for achieving complete tumor resection and preserving vital structures. Fernando et al.^510^ acquired an intraoperative dataset of 18 hypercubes from seven liver surgery patients. Different tissue types, including liver, bile duct, artery, and portal vein, were segmented from the images using machine learning models, demonstrating HSI’s potential for automatic anatomical structure identification to support surgeons intraoperatively in liver surgery. Studier-Fischer et al.^354^^,^^662^ collected a dataset of hyperspectral images from 20 physiological organs in pigs, assisting automatic tissue identification and context-aware surgical system navigation. Zhang et al.,^367^ on the other hand, focused on liver tumor delineation in surgical specimens using a U-Net model, potentially facilitating full resection of malignant tumors and reducing recurrence.

Thermal ablation is a widely used minimally invasive technique in liver surgery, where precise monitoring of ablation zones is crucial to ensure complete treatment and prevent damage to surrounding tissue.^620^ As mentioned earlier in Sec. 4.3.6, several *ex vivo* studies using bovine liver samples demonstrated HSI’s ability to differentiate normal, thermally affected, and ablated liver regions using machine learning approaches^443^^,^^505^ and identified optimal bands of 600 to 640 nm range.^618^^,^^619^ De Landro et al.^620^ carried out an *in vivo* study, where HSI detected spectral changes in ablated tissues in a porcine liver model. They analyzed the absorbance variation at three wavelengths targeting deoxyhemoglobin, methemoglobin, and water, and a significant increase up to 40% in the burned region was observed at 630 nm, which was attributed to methemoglobin absorption. Although preliminary, these studies demonstrate HSI’s feasibility for intraoperative thermal monitoring during liver ablation procedures.

**Fig. 23:**
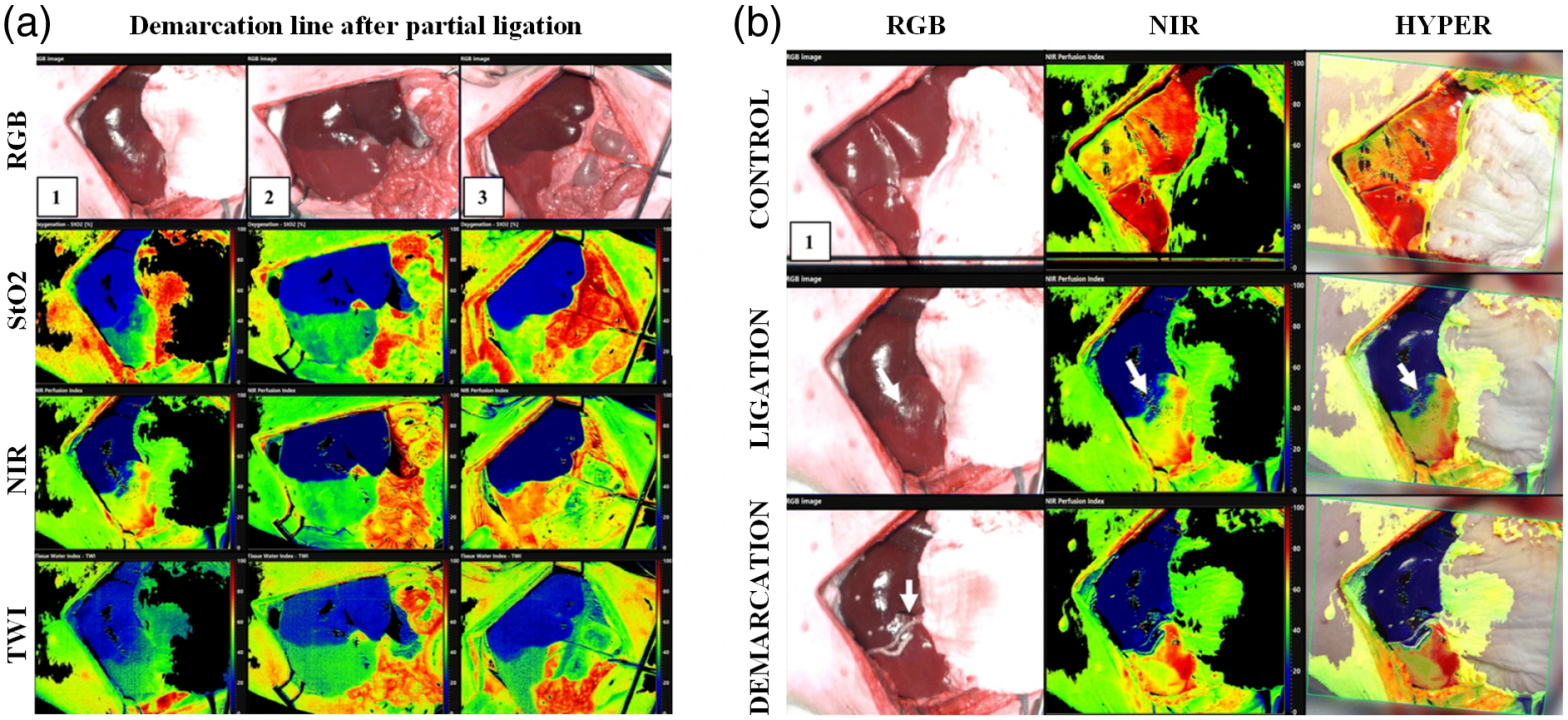
HSI system used in liver surgery. (a) Hyperspectral liver pictures of the three pig models after the ligation of the left hepatic pedicle branch. (b) Enhanced reality provided by the superimposition of the RGB camera with the hyperspectral near-infrared index, obtaining HYPER of pig 1. Unclear demarcation line solved by HSI is highlighted by the white arrows. The figure was reproduced from Ref. 272 with permission from Springer Nature.

Ischemia-reperfusion injury is a major concern in liver surgery as hypoxic damage and oxidative stress during revascularization can lead to significant postoperative complications. Targeting ischemia-reperfusion injuries in a major hepatectomy, Felli et al. first assessed liver viability by correlating HSI measurements of swine liver with biological analysis^689^ as well as classifying perfused and ischemic regions in intraoperative hyperspectral images using a CNN.^285^ The study was later extended to human patient major hepatectomy, demonstrating a correlation between HSI-derived perfusion parameters with clinical outcomes in 15 patients,^690^ suggesting that real-time HSI monitoring may predict postoperative liver dysfunction and complications. Sucher et al.^691^ first evaluated an HSI system in anatomic left liver resection, confirming its ability to identify exact resection planes based on oxygenation and perfusion. Then, Sucher et al.^692^ carried out comprehensive clinical investigations of HSI in major liver resections in 58 patients, to evaluate how portal vein embolization and vascular inflow control (VIC) affect microperfusion tissue oxygenation. HSI was performed at multiple key intraoperative stages with a variety of regions of interest, including embolized versus nonembolized liver tissues, VIC versus non-VIC tissues, and tumor versus nontumor tissues. HSI not only showed high sensitivity in perfusion measurement to guide precise anatomical resection margins but also identified tumor-specific optical signatures, establishing intraoperative tumor differentiation. Moreover, Urade et al.^272^ evaluated the accuracy of the HYPER system to identify the demarcation line during anatomical hepatectomy, as shown in Fig. 23, further supporting the utility of HSI as an intraoperative guidance tool.

Beyond liver procedures, the application of HSI has been extended to pancreatic surgery, where vascular perfusion and tissue viability are also critical determinants of postoperative outcomes. Wakabayashi et al.^273^ implemented real-time pancreatic perfusion assessment in a porcine model of partial pancreatic ischemia, where HSI-derived ${\mathrm{StO}}_{2}$ correlated with local capillary lactate levels and fluorescence-based enhanced reality slope, indicating that HSI may aid in defining resection margins and reducing the risk of pancreatic fistulas by ensuring adequate perfusion.

Moulla et al.^693^ evaluated HSI as a noninvasive tool to assess perfusion of the liver and stomach before and after gastroduodenal artery clamping in 20 patients undergoing pancreatoduodenectomy. HSI identified decreased liver oxygenation in patients with celiac artery stenosis after the clamping, prompting arterial reconstruction (median arcuate ligament release). The study demonstrated that HSI could aid in intraoperative decision-making by identifying ischemic risks in upper abdominal organs. Dietrich et al.^694^ used HSI for perioperative hemodynamic monitoring in pancreatic surgery, where HSI successfully detected oxygenation changes during anesthesia induction and a perioperative increase in tissue water content, which correlated with blood loss, syndecan levels, and surgery duration.

#### 4.4.6 Plastic and reconstructive surgery

HSI has emerged as a promising modality for perioperative monitoring in reconstructive surgery, where accurate and timely assessment of tissue viability is critical to preventing flap failure. Most clinical studies to date have adopted a prospective design and incorporated HSI acquisition at multiple perioperative time points, including intraoperative, early postoperative, and later follow-up stages. This multimodal timeline enables clinicians to track tissue oxygenation and perfusion dynamics beyond what conventional clinical inspection can reveal, offering a noninvasive tool for early complication detection and personalized recovery monitoring.

Thiem et al.^653^ monitored patients with either free or pedicled flaps in the oral and maxillofacial region using HSI from the intraoperative period up to more than 48 h postoperatively. HSI successfully detected all revision-requiring cases before clinical signs became apparent. In a subsequent study by the same group,^695^ HSI was used not only to assess flap viability but also to evaluate donor site recovery and patient-reported outcomes, reaffirming the clinical value of HSI-derived parameters. In a prospective clinical pilot study, Pruimboom et al.^400^ used HSI to identify early signs of flap ischemia and necrosis postoperatively in 10 women undergoing breast reconstruction. A significant difference in ${\mathrm{StO}}_{2}$ values was observed between necrotic flaps and vital flaps, yielding a 93% accuracy for necrosis detection. This was supported by changes in other perfusion-related parameters, including THI, PI, and TWI. Focusing on the impact of spectral analysis on ${\mathrm{StO}}_{2}$ measurement in reconstructive surgery, Merdasa et al.^696^ demonstrated the applicability of HSI in cutaneous forehead flaps. Their analysis showed that using a broad wavelength range, rather than a limited number of bands, produced more accurate and reliable ${\mathrm{StO}}_{2}$ values. Furthermore, a clinical study by Bunke et al.^697^ demonstrated the use of HSI in oculoplastic surgery. Particularly, HSI was employed to track subtle changes in periocular tissue oxygenation induced by the injection of epinephrine-containing local anesthetic, while laser speckle contrast imaging was used alongside to monitor the perfusion change around the injection site.

#### 4.4.7 Transplant surgery

Organ transplantation often represents the final therapeutic option for patients with end-stage organ failure.^330^ To optimize graft outcomes and make full use of the available donor pool, it is essential to assess physiological tissue parameters and organ viability accurately. In this context, machine perfusion techniques, particularly hypothermic and normothermic machine perfusion, have emerged as valuable tools for preserving and evaluating donor organs prior to transplantation.^698^ Although still in early stages of clinical adoption, HSI has shown potential in enhancing transplant surgical decision-making by quantifying perfusion, oxygenation, and tissue water content at critical timepoints such as during graft reperfusion or anastomosis evaluation. The majority of HSI research in transplantation has been focused on kidney and pancreas transplants, with promising results in both *ex vivo* graft assessment and intraoperative monitoring.

Markgraf et al.^330^ determined tissue water content in porcine kidneys during normothermic machine perfusion to evaluate the feasibility of HSI as a non-invasive tool for assessing organ quality. *Ex vivo* kidneys were reperfused with whole blood, sectioned, and partially dehydrated to generate variations in tissue hydration. By applying partial least squares regression, they established a correlation between spectral data and water content, enabling pixel-level visualization of water distribution across kidney tissue. Subsequently, Sommer et al.^474^ developed a CNN named KidneyResNet to predict *ex vivo* kidney functions in pig models. The modal initially achieved 62% classification accuracy, which improved to 100% following a majority decision approach, highlighting the potential of integrating machine learning with HSI data for objective graft evaluation.

Meanwhile, Romann et al.^699^ employed HSI to assess kidney quality and predict delayed graft function in human kidney transplants preserved via static cold storage or hypothermic machine perfusion. Their study showed that kidneys, which later developed delayed graft function, had significantly lower oxygen saturation and perfusion values at 10 and 30 min post-reperfusion, regardless of the preservation technique. Sucher et al.^700^ also evaluated kidney quality in human transplantation. Their findings aligned with Romann et al.,^699^ confirming lower oxygen saturation in delayed graft function cases. In addition, HSI images revealed differences in ureter perfusion after kidney transplantation and a ureteroneocystostomy, suggesting a potential role in identifying viable tissue for anastomosis during surgery.

In a preliminary study on simultaneous pancreas-kidney transplantation, Sucher et al.^701^ extended the application of HSI to pancreas allografts. HSI was used intraoperatively to monitor microcirculatory dynamics immediately after graft reperfusion. The study found significant differences in ${\mathrm{StO}}_{2}$ and TWI between the donor duodenum and recipient jejunum, indicating that HSI can detect subtle perfusion mismatches at anastomotic sites. These insights may enable surgeons to intervene in real time, reducing the risk of complications such as anastomotic leakage or early graft loss.

Furthermore, HSI has proved useful for liver transplantation. Two studies by Fodor et al.^702^ and Kneifel et al.^388^ explored liver viability assessment in liver transplantation, specifically normothermic machine perfusion, where HSI parameters correlated significantly with perfusate lactate and pH, signifying that HSI could provide an early, noninvasive assessment of graft viability before transplantation. Meanwhile, Wagner et al.^390^ quantitatively evaluated hepatic steatosis in liver transplantation using an HSI-derived tissue lipid index, showing the potential for HSI to replace invasive biopsy for assessing steatosis in donor livers.

#### 4.4.8 Other surgeries

Beyond the abovementioned procedures, HSI has also been explored intraoperatively across a wide range of surgical disciplines, including cardiovascular surgery, gastric cancer surgery, musculoskeletal surgery, breast-conserving surgery, and gynecologic oncology, demonstrating its versatility in tissue characterization, perfusion assessment, and margin delineation.

In cardiovascular research and surgery, HSI has demonstrated valuable applications in examining tissue perfusion and oxygen characteristics during and following surgical procedures. For instance, Grambow et al.^703^ applied HSI to assess tissue oxygenation and perfusion in a rat hind limb model following surgical microvascular anastomosis. Sucher et al.^704^ applied HSI intraoperatively in 10 patients undergoing carotid endarterectomy and successfully quantified vascular clamp-induced vascular injuries indicated by hypoperfusion and tissue oxygen deficits.

To help determine the gastrectomy margin, especially the gastric tumor that is not exposed to the mucosal surface, Mitsui et al.^411^ employed NIR HSI with an SVM classifier to identify gastric cancer in surgical specimens. Thanks to the deeper penetration of NIR light, they detected cancerous tissues even with a thickness of >2  mm.

With its rich spectral information, HSI can reveal subtle structural and biochemical differences in cartilage degeneration stages that are not seen visually, aiding intraoperative differentiation. Kistler et al.^398^ focused on cartilage conditions and investigated whether HSI could differentiate between healthy and damaged cartilage during open knee surgery. They found that absorption at 540 nm could effectively discriminate degenerated cartilage and was significantly higher for moderate and severe cartilage damage.

Last, HSI showed utility for surgical interventions in women’s health. Targeting adequate tumor resection margin during breast-conserving surgery, Kho et al.^705^ and Jong et al.^420^ collected hyperspectral images of *ex vivo* breast lumpectomy specimens and discriminated tumor and healthy tissues, with the former using an SVM classifier and the latter using a CNN. Van Vliet-Pérez et al.^160^ carried out tumor detection in surgical specimens of ovarian cancer using an SVM classifier. Puustinen et al.^111^ acquired a hyperspectral image dataset of four human placentas to facilitate the development of automated anatomical structure classifications and detection of anomalies that may not be visible to the naked eye.

### 4.5 Cancer Detection in Histopathology

Following discussions on how hyperspectral technology is implemented on a macroscopic scale, this section and the next focus on the utilization of HSI on a microscopic level. Histopathological evaluation remains the gold standard for disease diagnosis and staging, particularly in oncology.^211^^,^^286^^,^^472^ However, conventional histopathology techniques, such as H&E staining followed by microscopic examination, are labor-intensive, time-consuming, and susceptible to inter-observer variability.^11^^,^^359^^,^^706^ HSI emerged as a powerful tool for augmenting or even replacing certain steps in traditional pathology workflows. By enabling label-free, quantitative, and nondestructive tissue characterization, HSI holds promise for enhancing diagnostic accuracy and providing faster feedback. The following two sections summarize recent advancements in HSI-based histopathology over the past decade, with a focus on its applications such as tumor identification, tissue grading, and cellular activity analysis.

Integrating hyperspectral imaging into histopathological imaging may help facilitate cancer detection as HSI provides extra spectral information compared with traditional RGB imaging techniques. Hyperspectral microscopy has been actively investigated for cancer detection in recent years, including head and neck cancer,^467^^,^^548^ gastrointestinal cancer,^470^ brain tumor,^12^ breast cancer,^707^ pulmonary cancer,^472^ pancreatic cancer,^8^ skin cancer,^28^^,^^315^ and blood cancer.^208^ Here, we discuss several major cancer types that hyperspectral microscopy has been utilized to investigate in the recent decade.

#### 4.5.1 Head and neck cancers

Histological slides of head and neck cancer tissues are important for tissue histopathological analysis and diagnosis. Among all HNCs, SCC is a leading type and has been studied extensively. Bassler et al.^708^ employed darkfield elastic light scattering HSI in a proof-of-concept study for head and neck cancer detection in mouse models of oral SCC. Ou-Yang et al.^202^ combined transmission and fluorescence-based HSI using a push-broom system with hundreds of VIS-NIR spectral bands for the detection of oral SCC in 34 patient samples. The cell nuclei were identified in the basal-cell layer manually, and a fivefold method combined spectral and morphological features to yield good performance on the testing patients. Pertzborn et al.^359^ imaged 23 unstained tissue sections from seven patients with oral SCC and trained a ResNet for tumor recognition, which resulted in a 76% accuracy, 48% sensitivity, and 89% specificity. Note that the absence of staining may hinder morphological feature extraction. Nevertheless, without the influence of stains, the spectral features from an unstained slide reflect just tissue characteristics.

Ma et al.^548^^,^^709^ customized a dual-modal microscope system, which included a spatial–spectral hyperspectral camera and a high-quality color camera. The microscope was used to image head and neck SCC histologic slides obtained from the larynx, hypopharynx, buccal mucosa, and the floor of the mouth from 20 patients. They first proposed a PCA-based automatic image segmentation method to extract image patches of nuclei from hyperspectral histology images. Then, they evaluated the usefulness of morphological and spectral features for the differentiation of cancerous and normal SCC nuclei using only spatial information (RGB patches and CNN), only spectral information (spectral signatures and SVM), and spatial–spectral information (hyperspectral patches and CNN). The results indicated that both features contributed to the differentiation of nuclei; morphological features had a dominant effect, but spectral features provided significant improvement. Then, they further evaluated hyperspectral microscopy in whole-slide cancer detection.^203^ Whole-slide hyperspectral histology images of 32 slides from 16 different patients were acquired using an automated dual-modal hyperspectral microscope, and an inception-based neural network was implemented to carry out patch-based cancer detection in those images. In addition, they used hyperspectral super-resolution reconstruction to fuse hyperspectral and RGB images and generate high-resolution hyperspectral images with better image quality. By comparing the image classification results using RGB, original, and generated hyperspectral images, they proved that the spectral information in HSI helps with differentiation, and the generated hyperspectral images even further improved the performance.

Moreover, Zhou et al.^98^^,^^157^^,^^258^^,^^260^ developed a polarized hyperspectral imaging microscope system and applied it to the observation and detection of SCC in histologic slides. They first employed machine learning classifiers to classify spectral signatures extracted from four Stokes parameters of SCC and normal tissues. SVM delivered the best classification performance, with Stokes parameter S3 outperforming the others. Then, they employed a ResNet50 network to classify PHSI-synthesized RGB patches of cancerous and normal tissues, again comparing all four Stokes parameters. The results showed that S3 and S0 were more effective, with S3 achieving the best accuracy. In addition, they demonstrated that PHSI enhanced the visualization of collagen in SCC slides, which is a key component of the tumor microenvironment. Dense and sparse collagen around tumor cells appeared with high contrast in Stokes parameters images S1, S2, and S3, though indistinct in nonpolarized images.

**Fig. 24:**
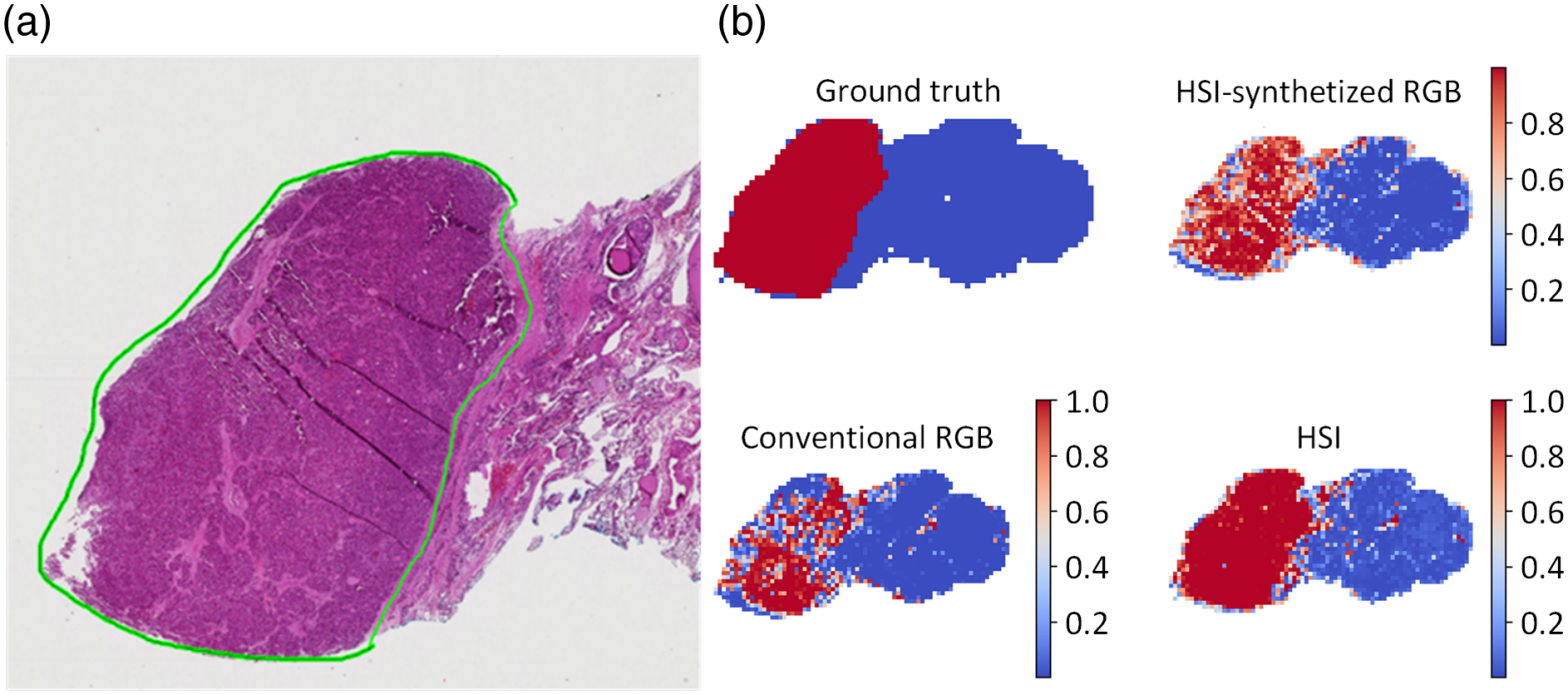
Comparative whole-slide thyroid cancer detection results using HSI, RGB, and HSI-synthesized RGB. (a) WSI of a patient using a whole slide RGB scanner. The tumor margin is outlined by a pathologist in green. (b) Cancer probability predictions made on this patient on a scale of 0 (normal, blue) to 1 (tumor, red).^467^

Meanwhile, for thyroid cancer detection, Tran et al.^93^^,^^467^^,^^480^ carried out automatic cancer detection in H&E-stained whole-slide thyroid cancer slides. They acquired a database of whole-slide hyperspectral and RGB histology images from 33 follicular thyroid carcinoma patients. VGG-19 neural networks were trained for cancer detection using RGB, hyperspectral, and HSI-synthesized RGB images, respectively. The network trained with hyperspectral data achieved an AUC of 0.966, outperforming both RGB (0.933) and HSI-synthesized RGB (0.947). Then, they expanded the database to 49 whole-slide images. With their proposed hyperspectral data augmentation methods and a video transformer, they achieved an F1 score of 88.1% and an accuracy of 89.64% using hyperspectral images. Representative results of whole-slide thyroid cancer detection are shown in Fig. 24.

#### 4.5.2 Gastrointestinal cancers

Gastrointestinal cancer is a broad classification that includes all cancers in the digestive tract organs, and it is another field where hyperspectral microscopy has been actively investigated. Colorectal cancer was one of the most prominent applications. Many preliminary works have been done using HSI/MSI to discriminate between benign adenoma and malignant carcinoma tissues within H&E-stained colon histological slides since a decade ago.^201^ In recent years, studies focused more on the refined methods for colon cancer detection, such as the use of texture features or CNNs, to improve cancer detection performances, with most studies utilizing MSI instead of HSI.^201^ Nevertheless, three studies targeting different perspectives of colon cancer still showed the value of HSI. Deal et al.^66^^,^^220^ employed excitation-scanning hyperspectral microscopy to differentiate neoplasia from normal colon tissue using autofluorescence. Spectral unmixing was applied to separate the autofluorescence signals of different endogenous fluorophores, enabling the observation of pathology-specific changes. Muniz et al.^409^ measured the absorbance of tissue slides in the infrared range using a micro-FTIR system and applied a fully connected DNN to differentiate healthy, inflammatory, and cancerous colon tissues.

In addition to colorectal cancer, HSI has shown promise in esophageal cancer. Maktabi et al.^286^ acquired hyperspectral images of histological specimens from 95 patients and employed an MLP to differentiate squamous epithelium cells, esophageal adenocarcinoma cells, and tumor stroma cells using their spectral signatures. They achieved an accuracy of 78% for both esophageal adenocarcinoma and stroma and 80% for squamous epithelium, indicating the usefulness of spectral features in hyperspectral data.

Several studies have also been done on gastric cancer detection using hyperspectral microscopy. Zhu et al.^206^ captured the transmission spectra of 50 normal and 50 cancerous nuclei from eight samples of human patients. By calculating the Euclidean distance between normal and cancerous spectra, they claimed that using part of the spectra could achieve better differentiation than using the entire spectra. Since 2019, researchers have been actively investigating HSI gastric cancer detection using deep learning approaches. Hu et al.^11^ proposed four deep learning classifiers using the spectral-only, spatial–spectral after dimension reduction, and full spatial–spectral features, respectively. The results determined that the network based on full spatial–spectral patches had the best performance with an accuracy of 97.57%. Liu et al.^470^ used a shallow residual network for gastric cancer detection in hyperspectral images of H&E-stained slides and achieved a 91.44% accuracy, which was 13.87% higher than that of the color images. Tian et al.^207^ studied gastric cancer type identification using a deep residual network and achieved 96.5% accuracy. To use different types of features more efficiently, Zhang et al.^211^ developed a symmetrically deep connected network that combined HSI and RGB features for joint training. They targeted precancerous lesions in gastric cancer and collected 924 images from 74 gastric tissue slides, which, with their model, achieved 96.5% accuracy. Du et al.^469^ also developed a joint classification method, which first uses spatial–spectral features to classify samples and then uses spectral features to refine the pixel-wise probability map, achieving 95.46% accuracy for gastric cancer and 95.89% for thyroid cancer.

Finally, for pancreatic cancer, Ishikawa et al.^8^ compared the cancer detection performance using hyperspectral and RGB images for pancreatic tumors and found a 14% improvement using HSI. In this work, they developed a whole-slide scanning microscope as well as a sophisticated HSI processing pipeline, which involved the construction of high dynamic range images, band reduction, estimation of dye amount, re-estimation of absorbance, and classification of nuclei pixels. Moreover, Notarstefano et al.^246^ employed Fourier-transform infrared hyperspectral imaging to analyze biomedical compositions (lipids, proteins, phosphates, and carbohydrates) in normal, dysplasia, pancreatic neuroendocrine tumor, and pancreatic ductal adenocarcinoma tissue samples.

#### 4.5.3 Skin cancer

HSI, combined with deep learning approaches, has been increasingly explored in skin cancer histopathology, enabling accurate classification and segmentation of melanoma and other cutaneous malignancies in pathological specimens. Wang et al.^315^ constructed a multiscale descriptor incorporating intensity-based spectral features and edge-based spatial features for the detection of melanoma and melanocytes in hyperspectral skin pathological images. Then, they developed a 3D fully convolutional network and compared it with a 2D CNN for melanoma segmentation.^28^ The 3D model, combined with band selection and modified loss function, achieved an overall segmentation accuracy of 92.67%. Tian et al.^434^ applied a deep residual network with HSI to differentiate melanoma and pigmented nevus and achieved 98% accuracy. Liu et al.^389^ implemented cutaneous SCC staging by a series of preprocessing followed by RF classification, which achieved a staging accuracy of 95.2%. Laimer et al.^96^ classified dark pigmented intraoral lesions, including amalgam tattoos, benign, and malignant melanocytic neoplasms in hyperspectral images of H&E-stained histologic slides, highlighting HSI’s potential as a diagnostic tool in digital pathology.

#### 4.5.4 Breast cancer

HSI has shown increasing promise in BC histopathology by enabling detailed spectral analysis of stained and unstained tissue sections, thereby enhancing the accuracy of tumor identification and classification. In a study by Ortega et al.,^707^ a push-broom hyperspectral microscope was used to acquire 112 hyperspectral images of H&E-stained breast cancer slides from two patients. With the help of a pathologist’s annotation, they extracted hyperspectral patches of cells and built a 2D CNN to differentiate tumor and normal cells. Despite the small number of patients, their results showcased that HSI could bring an 8% improvement in classification accuracy compared with RGB imaging. Vega et al.^134^ extended the work with a YOLOX-based object detection network and more biopsy samples from 18 patients. HSI data improved detection precision by 6.3% compared with HSI-synthetic RGB images.

Beyond the discrimination between normal and tumor tissues, Khouj et al.^122^ proposed the detection of specifically ductal carcinoma *in situ* with HSI using unstained and H&E-stained slides, respectively. With a subset of images manually annotated by pathologists, a semi-supervised k-means approach was applied to both types of images, suggesting good discrimination between normal and tumor regions even in unstained samples.

#### 4.5.5 Hepatic cancer

Hepatic cancer, including hepatocellular carcinoma and other malignancies affecting the liver, presents significant diagnostic challenges in histopathology. The integration of HSI into hepatic cancer histology analysis offers a promising avenue for more objective and quantitative tumor detection.

In 2018, Wang et al.^27^ evaluated the capability of microscopic HSI for the early detection of bile duct carcinoma within H&E-stained rat liver samples. By applying a feature extraction method based on the morphological watershed algorithm followed by SVM classification, the study successfully quantified tumor areas in biopsies at different time points. In 2020, Chen et al.^205^ employed an LCTF-based hyperspectral microscope to obtain the spatial–spectral data of H&E-stained hepatic carcinoma tissue slides. They developed an SVM classifier to differentiate the transmission spectra of cancerous and normal nuclei, achieving an impressive sensitivity of 99% and specificity of 98%. Liu et al.^204^ also utilized an LCTF-based system and trained an SVM model. Their approach focused on distinguishing different cell types based on the fluorescent spectral characteristics of 4′,6-diamidino-2-phenylindole (DAPI)-stained slides rather than transmission spectra. Huang et al.^710^ used a CNN for the diagnosis of hepatobiliary tumors, achieving classification accuracy ranging from 85% to 90%. More recently, Cinar et al.^24^ tested a 3D CNN for HCC detection in a liver microarray slide comprising three healthy and 27 HCC cases. Using 270 bands spanning the 400 to 800 nm range, their 3D CNN model achieved a classification accuracy exceeding 96%.

#### 4.5.6 Blood cancers

White blood cells, also called leukocytes, are hematopoietic cells of the immune system that are involved in protecting the body against both infectious diseases and foreign materials. Specifically, the abnormal development and uncontrolled proliferation of lymphocytes, a type of white blood cell, can lead to devastating blood cancers, such as leukemia and lymphoma. Their timely recognition in the peripheral blood is critical to diagnosis and treatment.^158^

Microscopic examination is one of the most common methods for leukemia diagnosis. In the past decade, HSI has been investigated for leukemia cell identification in several studies. Li et al.^209^ used an AOTF-based HSI microscope to observe blood smears. They proposed an automated algorithm to identify the cytoplasm and nucleus from leukocyte cells using both spatial features and spectral signatures. Moving on, Wang et al.^208^ combined spectral and spatial features and used a marker-based learning vector quantization neural network to identify lymphoblasts from lymphocytes in hyperspectral images, which yielded 92.9% accuracy. In two studies by Panda et al.,^483^^,^^711^ they first used Euclidean distance and Mahalanobis distance methods to detect chronic myeloid leukemia neutrophils from hyperspectral images,^711^ and then, they exploited 3D spectral gradient mapping for patient screening, which outperformed windowed SAM and achieved 84.2% accuracy.^483^ Moreover, Zhou et al.^158^ used PHSI to enhance the visualization of white blood cells in leukemia blood smears. Based on their polarized imaging microscope setup, both RGB and hyperspectral images were acquired, and Stokes vectors were calculated. Based on the Stokes vectors, three polarization metrics, DOP, DOLP, and DOCP, were obtained. It was found that the derived polarization metrics could improve the visualization of surface structures (protein patterns) of lymphocytes and enable subclassification of lymphocyte subpopulations.

In addition to leukemia, lymphoma is another type of blood cancer that originates in lymphocytes. Different from leukemia, which typically starts in bone marrow and spreads through the bloodstream, lymphoma usually originates in lymph nodes or the spleen and forms solid tumors in lymphoid tissue. Zelger et al.^460^ applied a CAE to MIR hyperspectral data to diagnose human lymphoma and differentiate between small and large cell lymphoma from unstained slides. The model achieved high classification accuracy, with three spectral bands corresponding to $v_{\mathrm{as}}{\mathrm{CH}}_{3}$, ester carbonyl groups, and amide regions identified for their high diagnostic sensitivity.

#### 4.5.7 Other cancers

Beyond the major cancer types frequently investigated with HSI-based histopathological analysis, several studies have explored its application in other clinically significant malignancies, such as brain, pulmonary, and bladder cancers. Although these cancer types each have limited representation in the literature over the past decade, they collectively demonstrate the expanding versatility of HSI for diverse diagnostic and prognostic tasks.

Ortega et al.^12^^,^^442^ carried out a series of studies for the detection of brain tumors, particularly glioblastoma, in hyperspectral images of histologic slides. They first evaluated several machine learning classifiers for automated brain tumor detection based on tissue spectral signatures.^12^ Then, they used an SVM classifier with superpixels in hyperspectral histologic images to distinguish glioblastoma.^442^ In the subsequent work,^283^ a CNN was employed for patch-wise classification of hyperspectral images. The model achieved 88% sensitivity and 77% specificity for glioblastoma detection, with an improvement of 7% and 8% compared with the sensitivity and specificity using conventional RGB histologic images.

Zhang et al.^472^ proposed a convolution combination unit-based 3D CNN named 3D-PulCNN for classifying pulmonary cancer in hyperspectral histologic images. Despite having relatively less parameters and complexity than a 2D VGGNet, the model achieved superior results. Tian et al.^706^ extracted intracellular fluorescent spectral fingerprints from hyperspectral images of H&E-stained lung cancer sections, to which they applied deep learning models for both gene mutation prediction and pathological classification. Enfield et al.^68^ analyzed spatial tumor-immune cell interactions in immunohistochemistry (IHC)-stained lung adenocarcinoma sections using HSI. By applying spectral unmixing to distinguish immune markers and spatial context modeling to quantify interactions, they proved that HSI-enhanced spatial immunophenotyping provided prognostic insights.

Last, one study by Kujdowicz et al.^241^ focused on the detection of urothelial bladder carcinoma, which is the most common bladder cancer. PLS-DA was applied to the spectra of epithelial and subepithelial tissues extracted from infrared hyperspectral images of cystoscopic biopsies. The model yielded 87% to 88% sensitivity for cancer grading but a lower yet satisfying performance for differentiating between invasive and noninvasive cases, showing the potential of IR HSI for bladder biopsy screening.

### 4.6 Nonmalignant Histopathology Applications

Although much of HSI research in histopathology has focused on cancer diagnosis, an increasing number of studies have demonstrated its potential in a wide range of nonmalignant conditions. These include applications in neuropathology, retinal and dermatological diseases, cardiovascular and pancreatic tissue characterization, immune-related kidney disorders, and even pathogen detection. HSI has also been utilized to study cell viability, subcellular processes, and nanoparticle interactions with biological tissues. These investigations collectively highlight the growing versatility of HSI as a powerful analytical tool for diverse histological contexts beyond oncology.

#### 4.6.1 Neuropathology

Neuropathology, the study of diseases affecting nervous system tissues at the microscopic level, has seen emerging applications of HSI aimed at improving the detection and characterization of pathological changes in brain tissue.

In one study investigating amyloid deposits associated with neurodegenerative diseases, hyperspectral confocal microscopy enabled detailed spectral differentiation of aggregated morphotypes based on their distinct fluorescence emission profiles.^239^ In another study,^41^ a spectral-scanning HSI microscope was employed for *in vitro* classification of mixed neural cultures. The researchers found that intrinsic spectral signatures without any fluorescence dye were sufficient to distinguish major cell types using an RF classifier.

Sacharz et al.^245^ examined the optimal acquisition parameters of FTIR-based HSI in nervous system research. They acquired hyperspectral images of cerebellum sections under various combinations of optical and measurement settings and evaluated their performance in tissue differentiation relative to acquisition time. The results indicated that the classification performance benefited from increased spectral resolution, whereas higher magnification yielded minor improvement and led to longer acquisition time.

#### 4.6.2 Retinopathology

HSI has been actively applied in ophthalmic research to analyze fluorescence and autofluorescence signals in the retina, offering detailed spectral information for disease detection and physiological assessment.

A series of studies have focused on hyperspectral autofluorescence imaging of the RPE and associated layers in healthy and diseased human eyes, with particular focus on AMD. Ben Ami et al.^90^ imaged the fluorescence emission of *ex vivo* human RPE-Bruch’s membrane flatmounts in the visible wavelength range using a hyperspectral microscope and decomposed the resulting hypercubes into five major spectral components, establishing distinct spectral signatures of autofluorescence compounds in healthy RPE tissue. Building on this framework, Tong et al.^89^ analyzed tissues from donors with AMD. In addition to the previously observed spectra in normal tissues, a unique fluorescence emission peaking near 510 nm at 436 nm excitation was found to be specific and sensitive to drusen. Subsequently, Dey et al.^349^ applied tensor decomposition and introduced group hypothesis testing to assess group-level spectral differences. Significant distinctions were revealed between the fluorescence emission spectra in the AMD group and the control group.

To investigate the biochemical processes underlying blood coagulation in retinal vessels, Podlipec et al.^215^ used HSI to capture retinal autofluorescence signals, which were further processed to quantify oxyhemoglobin concentrations in disrupted vessels and evaluate the extent of local coagulation. In a separate study involving retinal organoids,^712^ HSI was used to capture the fluorescence emission spectra, enabling localization of retinol distribution through post-processing analysis and demonstrating HSI’s potential for future monitoring and control of stem-cell-derived organoids.

#### 4.6.3 Dermatopathology

Li et al.^210^ imaged skin sections using an AOTF-based hyperspectral microscope system and produced various modes of images, including single-band images, false color images, virtual 3D surface views, and color-coded spectral clusters. By investigating the histological structures in the sections, HSI showed advantages in visualization enhancement compared with traditional RGB images. However, despite HSI offering practical and cost-effective imaging, a comparison study by Wilson et al.^340^ on melanin composition indicated that HSI does not reveal subtle melanin compositional variations as well as nonlinear pump-probe microscopy, suggesting future work of combining linear HSI with nonlinear imaging for pigment composition analysis.

#### 4.6.4 Cardiovascular pathology

HSI has demonstrated its value in characterizing cardiac tissue and vasculature microscopically. For instance, in the context of detecting plaque-related arterial lesions, Deal et al.^713^ investigated alterations in autofluorescence associated with artery remodeling, a process that may precede plaque formation, using hyperspectral fluorescence excitation scanning microscopy. Zhou et al.^257^ developed a PHSI microscope comprising a hyperspectral camera and two linear polarizers, one positioned between the light source and the sample and the other placed in front of the camera. Unstained slides of chicken heart were imaged at varying polarization angles. Then, the relationship between intensity and polar angle across different wavelengths was analyzed, offering a potential approach for depicting myocardial fiber orientation.

Hypertension, a primary risk factor for cardiovascular disease,^244^ has also been explored through the lens of HSI. Nazeer et al.^244^ examined the biochemical alterations in the salivary glands of hypertensive mice using FTIR HSI microscopy. The resulting infrared spectra revealed elevated levels of triglycerides, carbohydrates, and proteins, along with a reduction in nucleic acids. Through PLS-DA, the study achieved clear differentiation between hypertensive and control salivary gland tissues, suggesting the utility of HSI in identifying systemic biochemical effects of cardiovascular conditions.

#### 4.6.5 Pancreatic pathology

Hyperspectral imaging has also found valuable applications in islet biology and diabetes research, offering new insights into cellular function, stress response, and transplant viability.

To investigate real-time intracellular signaling in pancreatic islets, recent studies have leveraged advanced HSI systems that enable high-dimensional imaging of dynamic molecular events. Lavagnino et al.^117^ employed an imaging system combining snapshot HSI and light-sheet microscopy to measure fast ionic dynamics in pancreatic islets. The system enabled simultaneous imaging of intracellular calcium (${\mathrm{Ca}}^{2+}$) and zinc (${\mathrm{Zn}}^{2+}$) signals in α- and β-cells using multiple fluorescent biosensors. Their results revealed heterogeneous ${\mathrm{Zn}}^{2+}$ uptake in α-cells that correlated with ${\mathrm{Ca}}^{2+}$ activity and may contribute to glucose-mediated suppression of glucagon secretion. Following that, Elliott et al.^714^ extended the use of the microscopy system to simultaneously image two spectrally overlapping Förster resonance energy transfer (FRET) biosensors, one reporting cAMP signaling and the other caspase-3 activation, in pancreatic β-cells exposed to oxidative stress and hyperglycemia, which are key stressors implicated in diabetes pathogenesis.

In addition to functional imaging, HSI has been explored as a noninvasive method to evaluate islet viability prior to transplantation. To enable this noninvasive pretransplant assessment, Campbell et al.^715^ applied hyperspectral fluorescence microscopy to detect islet damage from oxidative stress, hypoxia, cytokine injury, and warm ischemia in a mouse model. Autofluorescence spectra of NAD(P)H, flavins, collagen-I, and cytochrome-C in intact islets distinguished compromised islets and accurately predicted restoration of glucose control following transplantation. These results support the potential of HSI as a predictive tool for islet viability and transplant success.

#### 4.6.6 Membranous nephropathy

Membranous nephropathy (MN) is one of the most common types of adult nephrotic syndrome.^385^ It is histologically divided into primary (idiopathic) MN and secondary MN. The former is often associated with autoantibody-mediated immune complex deposition within the glomerulus, and the latter may result from infections (e.g., hepatitis B virus), malignancies, autoimmune diseases, or drug exposures.^385^^,^^464^ Although the light microscopic findings in primary and secondary forms are often indistinguishable,^385^^,^^464^^,^^716^ subtle differences in the composition and distribution of immune complexes, particularly detectable at the molecular level, are increasingly being leveraged for differential diagnoses.

Three studies from a research group^385^^,^^464^^,^^716^ systematically investigated the use of microscopic HSI to classify idiopathic versus hepatitis B virus-associated MN in renal biopsies. Their earlier works introduced robust regression-based frameworks, including tensor patch-based discriminative linear regression^385^ and spatial–spectral density peaks-based discriminant analysis,^716^ both of which effectively leveraged the spatial–spectral structure of immune complexes, with the former achieving 98.77% classification accuracy based on a 68-patient dataset and the latter 99.36% on a 19-patient dataset. In another work of theirs,^464^ they proposed an integrated deep learning pipeline that achieved 95.04% accuracy in distinguishing two types of MN using a dataset of 20 patients. The collective results highlighted the potential of HSI-based diagnostics in renal pathology.

Furthermore, a study by Liu et al.^717^ investigated the predictive value of HSI for assessing treatment response to cyclophosphamide in primary MN. Hyperspectral images of H&E-stained renal slides were acquired in the VIS-NIR spectral range and analyzed using a 1D CNN. Distinct spectral differences between remission and nonremission groups were observed, particularly in the 525 to 700 nm range. The 1D CNN successfully classified the two groups, achieving an F1 score of 0.85 and an AUC of 0.857, demonstrating the potential of HSI combined with deep learning for predicting treatment efficacy in primary MN.

#### 4.6.7 Infectious pathogens

Accurate and timely detection of infectious pathogens is critical for effective disease control, outbreak prevention, and timely clinical intervention. HSI has emerged as a powerful modality for the label-free, multiplex, and spatially resolved detection of pathogens. Tao et al.^25^^,^^356^ developed deep learning models for the identification of infectious bacterial genera and species based on single-cell hyperspectral microscopic images. They first proposed a custom 3D CNN, named Buffer Net, which achieved 94.9% accuracy in classifying 11 bacterial genera, outperforming 1D CNN, 2D CNN, and 3D ResNet. The results underscored the importance of spatial–spectral features over spectral profiles or morphological data alone. In a subsequent study, they extended the classification from genus to species level by introducing a new model, BI-Net, which incorporated transfer learning to support the identification of uncommon pathogens. A database comprising 22 species (62 strains) was curated, including over 170,000 images of 15 common species and ∼2100 images for seven uncommon species. BI-Net achieved a classification accuracy of 92% for both common and uncommon species.

Human coronaviruses can cause a range of mild to severe intestinal and respiratory infections.^231^ Two studies employed hyperspectral enhanced darkfield microscopy (HS-EDFM) for the detection of human coronaviruses. Gosavi et al.^231^ investigated the scattering spectra of two human coronavirus strains, OC43 and 229E, and established distinct spectral signatures for each. Their results demonstrated the feasibility of strain-level differentiation in infected mammalian cells. Alafeef et al.^718^ focused on the quantitative detection of the highly pathogenic SARS-CoV-2, the virus responsible for COVID-19, which demands sensitive and rapid diagnostics. An ultrasensitive sensor based on hafnium nanoparticles was developed, which exhibited a peak shift in hyperspectral scattering spectra proportional to the concentration of viral RNA.

#### 4.6.8 Cellular pathology

Cellular pathology studies structural and functional dynamics at the cellular level, providing insights into biological processes and diagnosing diseases through cellular characteristics. Traditionally relying on invasive techniques such as chemical staining and IHC,^230^ noninvasive, quantitative tools are now being incorporated for more advanced individual cell analysis.^236^ HSI microscopy has demonstrated growing usage in areas such as differentiating cell types, visualizing cell signaling, metabolism, changes, and protein expression.

**Fig. 25:**
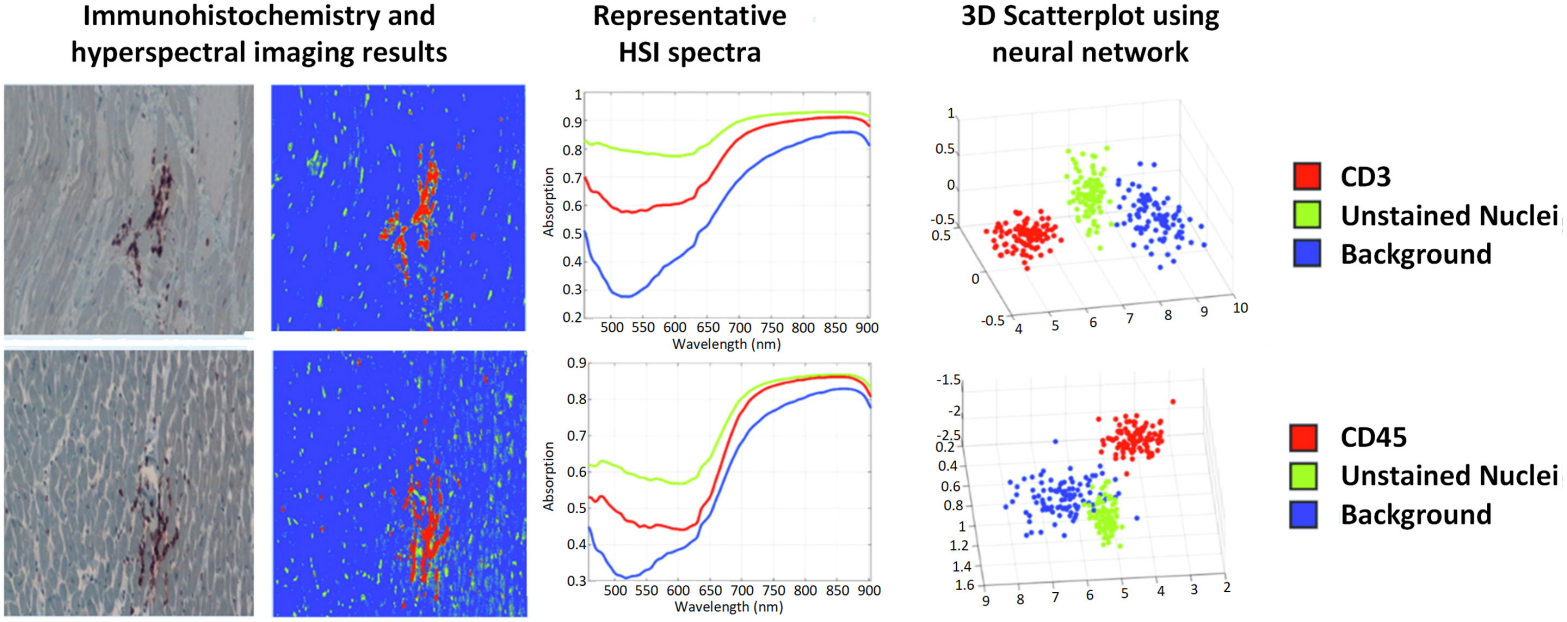
Light microscopic image of a tissue sample stained with CD3/CD45 and the corresponding HSI results. Representative HSI spectra of CD3/CD45 (red), unstained nuclei (green), and background (blue) 3D scatterplot of spatial separation of CD3 and CD45 versus background and unstained nuclei using a small neural network. Figure reproduced with permission from Ref. 719.

Bertani et al.^236^ utilized hyperspectral confocal microscopy and PCA of single cell spectra to discriminate between melanoma and HaCaT cells. To differentiate human adipose-derived stem cells from nondifferentiated control stem cells, Mehta et al.^230^ utilized hyperspectral darkfield microscopy (HS-DFM), which provided improved resolution and contrast visualization to observe lipid droplets during adipogenesis from the conventional staining method. Willenbacher et al.^97^ utilized HSI for quantification of Ki67, a prognostic marker of non-Hodgkin lymphoma, exemplifying the Ki67 proliferation index. HSI was compared against visual assessment (VA) and digital imaging analysis (DIA), and no significant differences were observed when quantifying the protein expression. Later, Brunner et al.^719^ analyzed VIS-NIR hyperspectral images to distinguish and quantify IHC-stained $\mathrm{CD}3^{+}$ and $\mathrm{CD}45^{+}$ inflammatory cells in myocarditis tissue obtained from autopsies, where more leukocytes, as marked by CD antigens, indicated acute myocarditis, as shown in Fig. 25. A small three-layer neural network was trained on the hyperspectral images. Similarly, HSI showcased no significant differences in the amount of ${\mathrm{CD}3}^{+}$ and ${\mathrm{CD}45}^{+}$ cells (p=0.460 and p=0.811, respectively) when compared with VA and DIA evaluation methods, signifying HSI’s accuracy in quantification to further aid diagnostics.

Rich et al.^237^ utilized hyperspectral confocal imaging to visual cAMP gradients in pulmonary microvascular endothelial cells (PMVECs). The results revealed that compared with 2D imaging analysis, the 3D hyperspectral data demonstrated marked cAMP gradients that were otherwise minimally detected by the 2D imaging approach. A few years later, Rich et al.^720^ expanded on this work, utilizing the same system to track second messenger signals, cAMP and ${\mathrm{Ca}}^{2+}$, and demonstrated spatial and kinetic distributions of the messengers in PMVECs. Within the same group, Leavesley et al.^31^^,^^721^ developed a hyperspectral imaging fluorescence excitation scanning microscope to investigate the green fluorescent protein emission in PMVECs and cell signaling of the second messengers, whereas Howard et al.^238^ used a combined hyperspectral and FRET imaging technique to visualize the spatial distribution of cAMP in human airway smooth muscle cells. These works suggest the feasibility of further research in understanding second messenger signals and pathways. Meanwhile, Rehman et al.^81^ utilized HSI to confirm fluorescence quenching—a reduction of fluorescence intensity—of NAD(P)H by a mitochondrial coupling agent in HeLa cells. HSI was combined with unsupervised mixing to isolate the emissions of free and bound NAD(P)H from cell autofluorescence. They found that the uncoupling agent selectivity quenches free and bound NAD(P)H, which validated the results of the unmixing of cell autofluorescence. This demonstrated HSI’s potential in investigating cell metabolism. Last, Wang et al.^84^ utilized HSI to assess the binding of macrophage-derived extracellular vesicle (EV) membrane proteins with their corresponding antibodies in an EV microarray assay. It rapidly measured the spectral signatures of the microarray and generated pixelized label-free images around each microwell.

These previously discussed articles primarily imaged specific cell samples or cellular composition. With a diverse range of histopathology applications where HSI offers molecular-level insights, some recent studies have specifically imaged larger biological samples, such as tissues, organs, and blood smears, and demonstrated its capability to identify and quantify biomolecules such as lipids, proteins, and autofluorescent metabolic cofactors, thereby enabling noninvasive histological analysis and functional tissue assessment. Golovynskyi et al.^59^ utilized a transmission-mode hyperspectral system in the NIR range to distinguish lipid-rich and protein-rich regions in porcine tissue slices. Their continuous multiband unmixing approach revealed lipid droplets as small as 1 to 10  μm, highlighting the technique’s utility for high-resolution deep-tissue lipid mapping. Favreau et al.^67^^,^^216^ employed an excitation-scanning hyperspectral microscope to extract tissue-specific autofluorescence signatures from unlabeled rat organs across 360 to 550 nm. Through PCA and spectral unmixing, the authors visualized distinct structural and metabolic features, including contributions from collagen, elastin, NADH, and FAD. Deal et al.^220^ further applied excitation-scanning HSI for discriminating between normal and cancerous human colon tissue based on unique excitation spectra, achieving successful classification using supervised training algorithms.

HSI has also been explored for blood analysis, where fine spectral differences allow classification of blood cell types and detection of hematologic conditions. Huang et al.^149^ proposed a framework combining a modulated Gabor wavelet and deep CNN kernels, which he used to carry out a binary classification between red blood cells and white blood cells as well as a five-class classification of different types of white blood cells in hyperspectral images of blood smears.

In orthopedic and cartilage research, HSI enables molecular-level assessments of extracellular matrix composition and disease-induced changes. Mahbub et al.^82^ evaluated hyperspectral fluorescence microscopy on articular cartilage slabs from healthy bovine and osteoarthritic human sources to investigate HSI’s potential in uncovering the molecular mechanisms underlying cartilage degradation and repair. HSI successfully identified key metabolic indicators, including collagen types I and II and the coenzymes free NADH and FAD, and it revealed disease- and treatment-related differences in molecular ratios within the superficial and transitional zones of the cartilage. Collectively, these studies demonstrate the versatility of hyperspectral microscopy in capturing intrinsic molecular contrast and underscore its promise for optical tissue diagnostics and metabolic profiling.

#### 4.6.9 Nanopathology

Nanoparticles can be synthesized in precisely controlled sizes and shapes using a variety of materials, offering versatile functionality for biomedical applications such as *in vivo* imaging, targeted drug delivery, and therapy.^227^ HSI, particularly in the form of HS-DFM, has been widely used to visualize and characterize intravenously administered nanomaterials in biological systems. Many studies utilize HS-DFM to analyze the scattering spectra of nanoparticles, whereas a few have employed a specific type of HS-DFM, such as hyperspectral plasmonic coupling microscopy, to measure interparticle plasmonic effects. Compared with techniques such as electron microscopy, HS-DFM provides a faster, cost-effective, and nondestructive alternative for nanoparticle analysis across a wide FOV and with higher throughput.^226^^,^^227^ Although this section overlaps with adjacent sections, the focus here is on the use of HSI for the validation, characterization, and spectral mapping of nanomaterials themselves.

HS-DFM has proven highly effective for the optical characterization of nanoparticles, enabling detailed analysis of their identity, localization, and behavior in biological environments. SoRelle et al.^227^ demonstrated the use of HS-DFM with adaptive image analysis as a nondestructive method to quantitatively identify particle types (e.g., nanorods, nanoshells) in *ex vivo* tissue sections. By capturing differences in accumulation patterns related to particle size and surface coatings, the method enabled sub-organ-level mapping of nanoparticles and their localization in tumors, offering higher throughput and broader FOV than electron microscopy. Peña Mdel et al.^226^ also showed that HSI, in the form of HS-EDFM, can accurately identify, locate, and map nanomaterials in porcine skin tissue samples. The technique was validated against conventional methods, including Raman spectroscopy, energy-dispersive X-ray spectroscopy, and scanning electron microscopy, paving the way for hyperspectral mapping and future semiquantitative analysis of these engineered nanomaterials. Chaudhari et al.,^722^ on the other hand, mapped the 3D rotational and translational dynamics of ultrasmall gold nanorods inside living cells using a polarized darkfield scattering microspectroscopy approach by incorporating a slightly tilted polarizer into a standard HS-DFM setup.

AuNPs have been a major focus of HSI research due to their strong plasmonic scattering properties, which facilitate sensitive detection and spectral analysis across biological environments. Jenkins et al.^539^ developed a rapid, multimodal screening approach to evaluate the agglomeration of AuNPs in whole blood using HS-DFM, mass spectroscopy, and confocal Raman microscopy. HS-DFM provided *in situ* spectral mapping of plasmon resonance, whereas complementary techniques confirmed particle identity and agglomeration status, showing that HSI could distinguish between particles and provide info about nanomedicine administration. Javed et al.^228^ employed HS-DFM to study the three major aggregation states of human islet amyloid polypeptides, which are key contributors to the development of type II diabetes. Specifically, they found a change in the spectral red shift induced by the interaction between the peptide states and AuNPs, potentially facilitating research in protein misfolding and therapeutic treatment deployment against amyloid diseases with nanoparticles. Zhang et al.^58^ used AuNPs as plasmonic labels to map large-scale epidermal growth factor receptor (EGFR) clustering on cell membranes using HS-DFM. By measuring spectral shifts caused by interparticle plasmon coupling, the technique detected differences in EGFR cluster sizes with shorter acquisition times than fluorescence super-resolution microscopy. Targeting polymer-coated gold/copper sulfide nanoparticles, which are a different type of gold-based nanoparticle that serves as plasmonic photothermal agents, Zamora-Perez et al.^723^ employed HS-EDFM to study the impact of physicochemical changes on their optical behavior and functionality. The scattering spectral signatures revealed a red shift induced by the acidic environment of simulated cellular fluid, spectral broadening caused by protein corona formation and ionic strength, as well as spectral narrowing due to nanoparticle degradation. Furthermore, two studies by Patskovsky et al.^224^^,^^724^ investigated nanoparticles in breast cancer cells. In one study, they employed hyperspectral reflected light microscopy to distinguish plasmonic Au/Ag alloy nanoparticles with different spectral signatures, enabling multitarget imaging for cancer diagnostics.^724^ In another study, they utilized a hyperspectral darkfield microscope to detect PEGylated gold nanoparticles functionalized with CD44 antibodies, providing a targeted imaging approach specifically for CD44-expressing breast cancer cells, which is essential for the improvement of nanoplasmonic-based imaging, disease detection, and treatment in a complex biological environment.^224^

HSI has also been applied to study silver nanomaterials, particularly for evaluating uptake and interaction within biological systems. Zucker et al.^223^ utilized a prism-based HSI system to evaluate characteristics, such as cellular uptake, on four different types of AgNPs. Cellular uptake was also looked at when Shannahan et al.^225^ utilized HS-DFM to assess how protein corona influenced AgNP properties and cellular responses. Analysis showcased shifts in scattering spectra and plasmon energies, illustrating protein corona’s influence on the toxicity and uptake capabilities of AgNPs into macrophage cultures. HS-DFM was also utilized by Ray et al.^222^ to measure scattering properties of Ag nanoplates encapsulated within polymer nanoparticles, which served as an optical scattering contrast agent, that were introduced into melanoma cells. Using endmember extraction, a 400% increase in the scattering contrast was observed, which helped identify the nanoplates within the cells, potentially useful for cancer diagnostics and therapeutic treatments.

In regard to carbon nanotubes, hyperspectral imaging has proven particularly valuable due to their chirality-dependent optical properties. Roxbury et al.^69^ employed wide-field NIR hyperspectral microscopy to spatially and spectrally resolve single-walled carbon nanotube fluorescence in living systems such as live HeLa cells, *ex vivo* mouse tissue, and *in vivo* zebrafish endothelium. The study demonstrated that this approach could distinguish up to 17 distinct nanotube chiralities—something conventional microscopy techniques cannot achieve—highlighting HSI’s potential for deep-tissue and single-molecule imaging in biomedical applications. Considering the significant variation of carbon nanotube biocompatibility and biodistribution due to their properties, Galassi et al.^212^ employed infrared hyperspectral fluorescence microscopy to visualize and quantify the bioaccumulation of intravenously administrated nanotubes in mice. HSI enabled label-free, high-resolution visualization of individual nanotubes across the mice organs.

Yakovliev et al.^42^ employed an LCTF-based HSI macroscopic imaging system in the SWIR range to acquire photoluminescence signals from mice administered with two exogenous photoluminescent nanoprobes, one being polymeric nanoparticles loaded with an organic dye and the other rare-Earth fluoride-treated nanoparticles. By applying spectral unmixing, they multiplexed the spectrally overlapping nanoprobes and obtained their spatial distributions from mixed suspensions. Most recently, Zhang et al.^431^ used HS-EDFM to simultaneously visualize and quantify the intracellular bioaccumulation of nanoplastics. They first established a hyperspectral reference library based on extracellular polystyrene nanoplastics and later refined it using intracellular spectra for improved identification accuracy. The results indicated that HS-DFM enabled label-free quantification of nanoplastic accumulation at the single-cell level and was effective across different cell lines and nanoplastic compositions.

#### 4.6.10 Validation of drug delivery and targeted therapy

Tailored therapeutic treatments are crucial for managing cancerous diseases due to diverse tumor characteristics and the short-term and long-term adverse effects of therapies such as chemotherapy.^234^ HSI, specifically hyperspectral darkfield microscopy, has shown usefulness in the validation of particle drug delivery and nanoscale targeted therapeutic agents to allow for more effective, less toxic patient-specific interventions.

Wen et al.^234^ used hyperspectral darkfield microscopy as well as confocal fluorescence imaging to validate the targeted drug delivery of an oxygenated particle platform loaded with doxorubicin, a chemotherapy agent utilized to treat numerous cancers, which they developed for lung cancer theragnostic.

Chen et al.,^725^ on the other hand, utilized hyperspectral darkfield microscopy to validate a nanoconstruct designed to target hypoxic tumor regions for photothermal ablation. HSI provided distinct spectra of gold nanorods, enabling localization of nanoparticles within tumors and confirming their effective accumulation and penetration in hypoxic regions. Tudor et al.^235^ used hyperspectral darkfield microscopy to evaluate the morphological and spectral changes in chondrosarcoma cells treated with iron oxide nanoparticles encapsulating doxorubicin. The nanoparticles enhanced the cytotoxic effects of photon and carbon ion irradiation. Alterations in nuclear spectral signatures indicated treatment-induced cellular modifications, suggesting the potential of HSI for identifying new spectroscopic biomarkers of cancer therapy response.

## 5 Discussion and Conclusion

### 5.1 Major Advances

#### 5.1.1 Hardware development

Over the past decade, hyperspectral imaging systems have undergone significant hardware improvements, making them smaller, lighter, and more practical for both laboratory and clinical applications. Advances such as SRDA snapshot and spatial–spectral cameras have been at the forefront of this trend. These technologies streamline data acquisition by capturing spectral data quickly and efficiently, reducing motion artifacts while maintaining high spatial and spectral resolution. The miniaturization of key components, such as detectors and light sources, has further enhanced maneuverability, especially in constrained environments such as surgical rooms. These improvements not only strengthen portability but also reduce manufacturing and deployment costs, making HSI more accessible to a broader user base. They also enable systems to deliver better performance in terms of resolution and sensitivity, paving the way for improved diagnostic accuracy and broader applications. Specifically, new application-specific systems, such as those based on surgical microscopes, endoscopes, laparoscopes, and ophthalmoscopes, have emerged and are tailored to the unique requirements of intraoperative and clinical use. Encouragingly, several commercial HSI systems are now being actively evaluated in clinical and surgical settings, reflecting the technological progress and growing recognition of HSI’s value among clinicians and biomedical researchers. These trends collectively indicate a shift toward the integration of HSI into real-world medical workflows, with the potential to improve diagnostic accuracy and patient outcomes across a range of specialties.

The integration of HSI with other imaging modalities has become a major trend. Multimodal imaging systems combine HSI with techniques such as fluorescence imaging, angiography, LiDAR, and polarized light imaging, offering complementary information about tissues. Fluorescence can highlight specific biomarkers, whereas polarized imaging provides polarization-resolved details. Image registration techniques and the use of beam splitters have simplified multimodal setups by enabling seamless alignment of different imaging outputs. Faster image processing speeds further facilitate real-time integration of data from multiple modalities. However, combining modalities introduces challenges, such as increased system complexity, data management issues, and higher computational demands for processing multimodal datasets.

Most importantly, commercial and prototype systems have emerged in the past 10 years, showing promising performance in clinical trials. A subset of studies leveraged commercially available platforms and reports prospective human evaluations in real clinical workflows, including perioperative monitoring and outcome-linked assessments (e.g., burn depth/grafting decision support, transplant and perfusion monitoring, diabetic foot ulcer healing prediction, and peripheral arterial disease follow-up). By contrast, many disease-detection and AI-driven decision-support applications, particularly in intraoperative neuro-oncology and dermatologic lesion classification, remain dominated by prototype or research-integrated systems, with evidence primarily consisting of single-center feasibility cohorts, limited patient numbers, and retrospective algorithm validations despite encouraging proof-of-concept performance. Nevertheless, these applications have shown great potential for next-step utilization of HSI. Table 8 summarizes the clinical readiness (e.g., clinical trial types and system commercial availability) of some representative studies for various applications.

**Table 8:** Summary of clinical readiness of representative HSI systems for various applications.

Application	System	System availability	Clinical evidence	Refs.
Burn depth assessment	TIVITA tissue system	Commercial	Prospective, single-center study with 49 patients analyzed, validating graft-need prediction	401
Cervical neoplasia	Custom multiscale AOTF-HSI system	Prototype	Pilot, single-center in-human feasibility study of 26 women (6 cervical neoplasia) with biopsy confirmation	26
Dermatology	Custom dermatologic snapshot HSI system	Prototype	Multi-center prospective data acquisition from 61 subjects (76 lesions) and retrospective validation with histology confirmation	462
Dermatology	SICSURFIS FPI-based handheld imager	Prototype	Single-center, prospective pilot clinical study of 33 patients (42 histology-confirmed lesions) evaluating lesion classification	38,39
Diabetes small-fiber neuropathy	Portable SWIR push-broom HSI scanner	Prototype	Single-center cross-sectional study of 120 patients and 20 controls, evaluating spectral differentiation versus sudomotor function and neuropathy	634
Diabetic foot ulcers	Custom foot-imaging system	Research/prototype	Single-center, prospective cohort dataset of 43 patients, offline analysis for 12-week outcome prediction	14
Diabetic foot ulcers	HyperView handheld HSI device	Commercial	Single-center clinical study of 27 patients with longitudinal follow-up over 12 weeks for healing analysis	403
Free-flap in head and neck reconstruction	TIVITA Tissue system	Commercial	Single-center, prospective randomized controlled clinical trial with 30 patients, serial monitoring to 6 months	695
Intraoperative bowel anastomosis	TIVITA Tissue system	Commercial	Prospective, single-center clinical study of 46 consecutive patients, evaluating HSI for surgical decision.	726
Intraoperative brain tumor detection	SLIMBRAIN system	Research/prototype	Single-center system verification during brain tumor operations	162
Intraoperative brain tumor detection	Custom intraoperative push-broom HSI system	Prototype	Prospective, single-center, *in vivo*, intraoperative clinical system evaluation and data collection of 34 patients (61 images)	137
Iris imaging	Custom iris hyperspectral imaging system	Research/prototype	System feasibility evaluation on 8 healthy volunteers	33
Kidney transplantation	TIVITA Tissue system	Commercial	Retrospective, single-center, cohort study with 38 kidney allografts to predict delayed graft function	699
Laparoscopic colorectal surgery	TIVITA Tissue system	Commercial	Prospective single-center study of 68 patients, using paired HSI ${\mathrm{StO}}_{2}$ versus ICG parameters to define cutoffs	684
Liver transplant	TIVITA Tissue system	Commercial	Prospective, single-center, one-arm study with 25 donor liver allografts, compared to biopsy across workflow timepoints	388,390
Minimally invasive surgery	TIVITA Mini Endoscopy Edition	Commercial	Prospective, single-center system evaluation in 19 patients undergoing gastrointestinal resections	672
Neuro-oncology surgery	Real-time lightfield HSI system	Research integration	Prospective, single-center feasibility study in one patient, includes OR workflow usability assessment	100
Parkinson’s disease	Fundus camera integrated with HSI	Research integration	Single-center case–control study with 20 PD patients and 20 controls	584
Peripheral arterial disease	HyperView HSI system	Commercial	Single-center, prospective cohort pilot study of 34 patients over 6 weeks with clinical improvements.	609
Septic shock	Custom bedside HSI setup	Prototype	Single-center, prospective ICU study of 89 patients, associating oxygenation with mottling severity and 28-day mortality	395
Systemic sclerosis-associated Raynaud phenomenon	HyperView handheld HSI camera with OxyVu	Commercial	Prospective single-center study with 13 patients and 12 healthy controls, analyzing oxygenation dynamics between groups	668
TAVI-related kidney function	TIVITA Tissue system	Commercial	Prospective, single-center observational pilot study with 40 patients and 20 controls, correlating ${\mathrm{StO}}_{2}$ versus creatinine after TAVI.	604
Wound infection	Swift Ray 1 device with Swift skin and wound app	Commercial	Multicenter, prospective outpatient study of 66 patients, evaluating HSI-based classification against clinician-determined infection status	628

#### 5.1.2 Expanded wavelength ranges

Research into the RNIR and SWIR spectral ranges has grown significantly, driven by their ability to probe deeper into biological tissues and detect critical biomarkers. RNIR and SWIR wavelengths are particularly suited for imaging water, hemoglobin, and lipids, enabling applications such as tissue hydration analysis, oximetry, and fat content measurement. RNIR wavelengths can penetrate up to 3 to 5 mm in biological tissue, whereas SWIR can reach even deeper. However, imaging at these wavelengths presents unique engineering challenges. Unlike visible light systems that rely on silicon-based detectors, RNIR and SWIR systems require InGaAs sensors, which are more expensive. In addition, the heat generated by these systems necessitates advanced cooling mechanisms to maintain stability and prevent thermal noise. Overcoming these challenges is crucial for the further adoption of RNIR and SWIR imaging in clinical and industrial settings.

#### 5.1.3 Image processing methods and AI algorithms

Advancements in image processing techniques have transformed how HSI data are analyzed, making it faster, more accurate, and increasingly automated. The adoption of deep learning has been particularly impactful, enabling highly accurate identification and classification of tissues and materials. These algorithms can process vast amounts of spectral and spatial data efficiently, offering potential to match or even surpass human expertise in certain applications. Open-access datasets and big data initiatives have further democratized HSI research, allowing scientists to develop and test algorithms without needing direct access to tissues or imaging systems. Still, publicly available hyperspectral data are still very few. As of this review, we have only found three large datasets for public download: Ortega et al.^442^ published a dataset of histological scans of glioblastoma; Puustinen et al.^111^ published a dataset of hyperspectral placenta; Studier-Fischer et al.^662^ published a dataset of 20 types of pig organs *in vivo*.

Faster processors and improved software now enable near real-time analysis, making HSI more practical for dynamic environments such as operating rooms. Moore’s law was an observation made during the heyday of microprocessors, which predicted that the density of a processing unit within the same chip doubles every two years.^727^ As of now, the observation still holds, meaning computers of tomorrow will continue to have faster processing time compared to computers of today. Graphic processing units, which leverage parallel operations to speed up matrix multiplications, are at the forefront of deep learning. Because deep learning is a rapidly expanding industry, improving GPU capabilities and efficiency is a priority in hardware research. This leads to GPUs becoming more and more powerful. These advances in image processing not only enhance the speed and accuracy of data interpretation but also broaden the accessibility and usability of HSI across disciplines.

#### 5.1.4 Biological and clinical applications

In the past decade, the field of biomedical HSI has witnessed a notable expansion in both the breadth and depth of its applications, far surpassing the foundational studies that characterized the earlier phase. Early research primarily focused on proof-of-concept demonstrations for spectral discrimination of tissue types, disease diagnosis, and surgical visualization, often constrained by bulky instrumentation, slow acquisition speeds, and limited clinical validation. By contrast, recent years have seen HSI evolve into a versatile and powerful tool for real-time, *in vivo*, and clinically translatable imaging.

In the early stage, the majority of biomedical HSI studies concentrated on relatively specific domains, such as basic skin assessment and *ex vivo* tissue analysis. From 2014 onward, advances in imaging hardware, data processing algorithms, and computational resources have catalyzed a dramatic increase in clinical and translational applications. Faster, more compact, and more versatile systems such as snapshot cameras and compact laparoscopic units made HSI increasingly viable for real-time, intraoperative, and point-of-care use.

A key transformation is the diversification of cancer types studied with HSI. In addition to early investigations into cervical, skin, colon, and prostate cancers, the past decade has seen HSI applied to a broader range of malignancies, including brain tumors, esophageal cancers, hepatic malignancies, blood cancers, and emerging reports on lung, intraocular, kidney, and bone cancers. Previously studied areas such as head and neck cancer, breast cancer, and gastrointestinal cancers have experienced significant expansion, both in clinical scope and imaging sophistication. Beyond oncology, nonmalignant diseases such as retinal disorders, cardiovascular diseases, diabetes (particularly diabetic foot), and systemic shock have remained active areas of research. Although these conditions were already explored before 2014, recent studies have provided improved quantification of tissue oxygenation and perfusion, enhanced imaging speed, and deeper understanding of pathophysiological changes through spectral biomarkers. Meanwhile, neurological diseases such as Alzheimer’s and Parkinson’s diseases, as well as burn and wound monitoring, have gained growing attention.

One of the most impactful shifts has been the rise of HSI for intraoperative image guidance. From custom-built prototype systems to commercial-ready platforms, HSI is now being deployed in operating rooms to support critical surgical decisions. Abdominal surgeries remain a major application field, but the most notable growth has occurred in neurosurgery, head and neck surgery, transplant surgery, and especially plastic and reconstructive surgeries, where HSI assists in flap viability evaluation, perfusion monitoring, and margin assessment. Quantitative perfusion and oxygenation maps provided by HSI now complement or challenge traditional tools such as ICG fluorescence.

Histopathological HSI, both in cancer and noncancer contexts, has seen significant growth. HSI has been applied to label-free characterization of formalin-fixed, paraffin-embedded tissues, enabling molecular and morphological insights. Although still largely in the proof-of-concept phase, especially when compared with the mature ecosystem of RGB digital pathology and FDA-approved analysis algorithms, this area is gaining traction. The potential for automated spectral analysis and biomarker quantification without staining promises a disruptive shift in future digital pathology pipelines. Furthermore, an entirely new frontier has emerged in the use of HSI for nanoparticle imaging and characterization. Label-free hyperspectral detection of nanoparticles such as gold nanorods, silica, or polystyrene beads enables visualization of biodistribution and intracellular uptake, enhancing our understanding of therapeutic delivery and nanotoxicology. This was virtually absent from the literature before 2014.

In summary, the last 10 years have shifted HSI from a niche research modality into a clinically adaptable, multipurpose platform, enabling *in vivo* diagnostics, surgical support, and mechanistic biological investigations. This growth reflects not only an increase in the number of applications but also a qualitative leap in clinical readiness and integration with existing medical infrastructure. Although some areas are still in the exploratory phase, the trajectory of interest and technological development indicates a promising and sustained future for biomedical HSI.

### 5.2 Current Bottleneck and Limitations

#### 5.2.1 Cost of HSI camera

Despite being more cost-effective for diagnostic imaging than larger modalities such as MRI and CT, HSI cameras remain relatively expensive as compared with traditional RGB cameras, posing a barrier to widespread adoption. One major factor contributing to the cost is the lack of scalable mass production methods. Unlike RGB cameras, hyperspectral systems require specialized components that introduce variability between individual units, necessitating complex calibration procedures for each camera. These calibration steps are more intricate compared with standard imaging systems, further driving up costs. In addition, the production of essential components such as optical filters and monochromators is resource-intensive and adds to the overall expense. Professional-grade HSI systems continue to cost from a few thousand to tens of thousands of dollars, making them inaccessible to many researchers and practitioners. To address this limitation, some researchers have turned to low-cost alternatives, such as adapting smartphones and inexpensive cameras for hyperspectral imaging. Although these approaches lower the costs, they often come at the expense of image quality, spectral resolution, and system reliability. The trade-offs associated with these budget-friendly solutions highlight the ongoing need for innovation in manufacturing and design to produce high-quality hyperspectral systems at a lower cost.

#### 5.2.2 Biomarker mechanisms

Another significant limitation in HSI research is the lack of comprehensive studies exploring the wide range of biomarkers and chromophores present in the human body. Although the body contains thousands of potential biomarkers and chromophores capable of providing valuable insights into human health, most studies still focus on a few well-known ones, such as hemoglobin, melanin, and lipids. This focus is likely because these chromophores are extensively studied, and their reflectance spectra are readily available in online databases, making them accessible to researchers. However, many lesser-known chromophores and proteins remain underexplored. Researchers interested in studying these compounds often face the challenge of extracting them themselves, a process that can yield inconsistent and unreliable data. In addition, there is no formal framework connecting specific chromophores to physiological processes and diseases, which limits the ability to establish a comprehensive understanding of how spectral changes relate to health and disease. For example, a researcher studying cancer might want to understand how various cancer types influence the concentrations of oxyhemoglobin, deoxyhemoglobin, lipids, water, and other proteins, and how these changes collectively affect the reflectance spectrum of the affected tissue.

Currently, most studies focus on basic relationships, such as how oxygenation rates or lipid spectra change in diseased tissues. Although this information is valuable, it falls short of providing a detailed, interconnected view of biomarker changes and their physiological significance. Bridging this gap should be a key objective for future HSI research, with the goal of linking spectral signatures to complex tissue physiology and disease processes in a systematic and meaningful way.

#### 5.2.3 Challenges in band selection

The lack of comprehensive studies on biomarkers directly contributes to the absence of universally acknowledged optimal spectral bands for HSI. As many biomarkers remain underexplored, there is no standardized reference for selecting spectral bands that provide the most diagnostic relevant information. As a result, most current studies default to using a large number of spectral bands to ensure that key information is not missed. Although this approach maximizes data collection, it also increases computational burden, requires longer acquisition times, and makes real-time analysis more challenging. Unlike the SWIR range, where many molecules exhibit strong absorption at specific wavelengths, the VIS-NIR range lacks clear, dominant absorption peaks for most biological molecules. This makes it difficult to explicitly define optimal spectral bands for specific applications.

Although some studies report similar findings regarding effective spectral bands for certain applications, most band selection results remain highly method dependent. The lack of consistency across studies prevents the establishment of a standardized spectral band framework, which is crucial for improving the reliability and reproducibility of HSI-based medical diagnostics. Addressing this gap requires systematic biomarker studies and the development of standardized band selection protocols tailored to different medical applications. With publicly acknowledged optimal bands, HSI cameras could be designed and manufactured with tailored spectral bands for specific applications. This would enhance diagnostic accuracy, reduce computational complexity, and improve the overall efficiency of HSI in medical and industrial settings.

#### 5.2.4 Data storage and data sharing

Hyperspectral data are commonly stored in binary files using one of three formats: band sequential, band interleaved by pixels, or band interleaved by line. Each image is accompanied by a header file containing sensor parameters, center wavelengths, and file format information. Typically, hardware manufacturers that produce hyperspectral cameras also provide proprietary software for reading and processing hyperspectral images. However, general-purpose, publicly available software libraries are also available for standardized hyperspectral data processing.^728^ Data storage is a critical consideration in hyperspectral imaging due to the large size of the hypercube. For instance, a single image with a resolution of 1000×1000  pixels and 150 wavelengths would require more than one gigabyte storage if double-precision data are used. Although converting the data to an unsigned 8-bit integer format can reduce the storage size, it may reduce precision and lose small spectral signals. Although the images may appear visually similar, the spectral curves reveal slight differences that could impact analysis. Section 3 already discussed spectral selection and dimension reduction techniques as additional methods to reduce data size while preserving key information. The size of the data may also affect the training of neural networks. Villa et al.^729^ investigated the power consumption and training time when reducing the bit depth of input hyperspectral medical images from 64-bit to 16-bit data. They found a ninefold speedup of inference speed, with minimal decrease in accuracy.

The size of hyperspectral image files adds difficulty to data sharing. The large size of hyperspectral data compared with RGB images makes uploading and maintaining these datasets more expensive. Currently, publicly available datasets for hyperspectral biomedical research are limited. Some remote sending HSI data are available, such as the Harvard dataset,^730^ the Hyperion dataset,^731^ or the CAVE dataset,^732^ which primarily feature outdoor scenes or satellite imagery. However, the subjects in these datasets exhibit intrinsically different spectral signatures, which possibly limit their utility for training or evaluating models in biomedical applications.

#### 5.2.5 Dataset size and model training

The large size of HSI data also presents a challenge for analysis, often requiring substantial computational resources and time. Deep learning has emerged as the leading method for handling these large datasets due to its ability to extract features that were previously difficult to identify. Although raw hyperspectral data can be directly fed into these models, combining expert-driven feature extraction with deep learning has been shown to improve system performance and reduce noise, highlighting the importance of domain knowledge in guiding computational approaches. Modern deep learning architectures, such as CNNs, transformers, and large language models, are now being adapted to process HSI data. These models excel at handling high-dimensional data but require considerable computational power due to the sheer size of HSI datasets, which can be orders of magnitude larger than standard RGB images. The relationship between dataset size and training time is often nonlinear, for example, a 10-time increase in data size might mean a 100-fold increase in training, with bottlenecks often occurring in data storage and retrieval systems. To mitigate these effects, researchers are exploring strategies such as hardware-based processing and model optimization techniques such as pruning and quantization.

As models grow in size and complexity, interpretability has become a key challenge. The outputs of advanced neural networks can be difficult to decipher, limiting their utility in applications where understanding the reasoning behind a classification is crucial. Addressing these limitations will require a balance between model performance and interpretability to ensure that HSI analysis remains both effective and efficient.

### 5.3 Future Directions

#### 5.3.1 Future emerging technologies

Several advancements in HSI technology are poised to transform its capabilities and applications. The widespread adoption of SWIR imaging systems will enable deeper tissue penetration and the detection of additional biomarkers, expanding HSI’s utility in fields such as oncology and neurology. Simultaneously, snapshot imaging cameras with increased wavelength coverage and higher spatial resolution are expected to become more prevalent, allowing for more detailed and accurate tissue analysis in both research and clinical settings.

Another emerging area is video HSI, which would allow continuous monitoring of physiological processes for over several minutes or hours. This could provide new insights into dynamic biological processes, such as oxygenation changes, wound healing, or metabolic activity, that are difficult to capture with static imaging. Customized HSI systems tailored to specific tissue types and biomarkers are also likely to rise. Although current systems are often designed to highlight oxygenated and deoxygenated hemoglobin, future systems could be optimized for detecting lipids, melanin, or other chromophores with characteristic absorption peaks.

Foundation models, which are large-scale machine learning models pre-trained on vast amounts of data, are gaining traction in imaging fields such as RGB histopathology and CT scanning. These models can be fine-tuned for specific tasks, providing a robust starting point for diverse applications. For the HSI community, foundation models could accelerate research by enabling rapid image classification, anomaly detection, and feature extraction across different datasets. They could also standardize analysis workflows and reduce the computational burden of training models from scratch.

Another transformative innovation would be the creation of a collective database cataloging the reflectance spectra of all chromophores in the human body. Such a database would provide researchers with invaluable reference data for understanding tissue composition and linking spectral signatures to physiological and pathological states, fostering collaboration and accelerating discoveries in HSI.

#### 5.3.2 Future applications

Cancers—both in diagnostic imaging and intraoperative detection—remain the most extensively studied domain of biomedical HSI. Among them, brain tumors, head and neck cancers, breast cancers, skin cancers, and gastrointestinal malignancies have garnered substantial research interest and demonstrated measurable progress in clinical feasibility. However, other cancer types such as gynecologic malignancies (e.g., ovarian and cervical cancer), pulmonary cancers (e.g., lung adenocarcinoma and small-cell lung cancer), bone tumors (e.g., osteosarcoma), and prostate cancer remain underexplored in hyperspectral imaging research. The limited number of studies in these areas may be attributed to factors such as anatomical inaccessibility, challenges in integrating HSI into endoscopic or minimally invasive platforms, lower disease incidence rates in certain populations, or insufficient spatial resolution and penetration depth of current systems for internal or deep-seated tissues. Furthermore, the lack of large, annotated datasets for these cancers hampers the development and validation of robust classification algorithms. Future research should aim to overcome these technical and logistical barriers by developing application-specific imaging platforms, enhancing data acquisition in challenging anatomical regions, and conducting multicenter clinical studies to validate HSI’s utility across a broader spectrum of oncologic diseases.

Intraoperative HSI applications currently center around tissue perfusion assessment, especially within abdominal surgeries and perioperative reconstructive procedures. These use cases have reached relatively mature stages, with several evaluated directly within surgical workflows using commercially available systems, and have demonstrated reliable performance in real-time monitoring. By contrast, despite promising early results, intraoperative tumor detection using HSI remains largely in the feasibility and proof-of-concept phase. Although numerous studies have demonstrated the spectral distinction between tumor and normal tissues, most have been limited to small-scale experiments involving a narrow range of patients or tissue types, often under controlled conditions. The majority of machine learning or deep learning models have been trained on small, homogeneous datasets, which restrict their ability to generalize across inter-patient variability, tumor heterogeneity, and overall anatomical diversity. As a result, these models tend to perform well in isolated cases but struggle in real-world surgical environments that are complicated by dynamic tissue deformation, varying lighting conditions, bleeding, and instrument-induced artifacts. Future research should focus on developing HSI systems that are robust, rapid, and fully integrated into the surgical workflow. This includes designing compact, sterilizable imaging devices, improving real-time data acquisition and processing pipelines, and implementing adaptive models capable of handling intraoperative variabilities. Multicenter studies with large, diverse patient cohorts are essential to establish clinically reliable training and testing datasets. Furthermore, the development of surgeon-friendly interfaces, possibly augmented by AR with overlaid classification maps, will be key for enhancing intraoperative usability. Particular attention should be given to tumor types and surgical contexts where margin assessment is critical—such as glioma resection in brain surgery, deep-margin control in head and neck procedures, and margin preservation in breast-conserving surgery, where HSI can offer a noncontact, label-free alternative to existing guidance modalities.

Certain areas with a growing body of work still possess extensive untapped potential. Neurological disorders, especially Alzheimer’s disease, deserve greater attention. As a disease with escalating societal and economic burdens, Alzheimer’s is a prime candidate for early and cost-effective detection using HSI, particularly via retinal imaging. HSI’s speed, noninvasiveness, and cost-efficiency make it suitable for mass screening and longitudinal monitoring. Another highly promising domain is dermatology and cosmetics. The increasing demand for personalized skincare, anti-aging therapies, and noninvasive cosmetic monitoring has created a fertile environment for the development of commercial HSI-based devices. These systems could be designed as handheld devices capable of rapid spectral analysis and real-time feedback. Such tools would enable dermatologists and cosmetic professionals to objectively assess skin condition, track changes over time, and personalize treatments based on quantitative biophysical data. For instance, HSI could support early detection of subclinical erythema, assess the efficacy of moisturizers or anti-pigmentation agents, or monitor the vascular response to laser treatments or microneedling. Moreover, the cosmetic industry stands to benefit from the integration of HSI into clinical trials and product validation studies, offering an objective, reproducible means of assessing skin responses across diverse populations and skin types.

Despite substantial progress of HSI in oncology, surgery, and diagnostics, several promising avenues remain underexplored. Emerging application areas with high potential include neurological diseases beyond stroke and Alzheimer’s, such as traumatic brain injury and multiple sclerosis, particularly when paired with hyperspectral retinal imaging. In pulmonary medicine, future work should seek to integrate HSI into bronchoscopic platforms for real-time diagnosis and tissue oxygenation mapping, with applications in chronic obstructive pulmonary disease, pneumonia, and lung cancer. Similarly, HSI-based perfusion and oxygenation measurement could open new avenues in nephrology, hematology, and musculoskeletal diseases. In women’s health, preliminary efforts have focused largely on breast and ovarian cancers, with little attention given to obstetric or gynecologic applications. Developing HSI workflows tailored to maternal-fetal monitoring, placental perfusion, and reproductive medicine would fill a significant clinical gap.

Moreover, HSI offers untapped opportunities in immune-related diseases, infectious pathologies, and neurodegenerative conditions, especially for early detection and therapeutic monitoring. These disorders are characterized by subtle metabolic and biochemical shifts that HSI is uniquely capable of capturing. Expansion into pediatric care, neonatal diagnostics, and maternal-fetal health could also benefit from HSI’s noninvasive and label-free capabilities, especially in monitoring tissue viability, oxygenation, and microcirculatory status in vulnerable populations.

In the domain of cancer histopathology, there has been a surge of studies in the past decade leveraging hyperspectral microscopy to streamline intraoperative consultation and accelerate the diagnostic pipeline. Quantitative evaluations have shown improvements over traditional RGB imaging. However, commercial adoption remains limited due to the lack of foundation models, standardized datasets, and regulatory approval. By contrast, RGB imaging maintains strong support from FDA-cleared devices and large-scale foundation models. Thus, future work should aim to expand hyperspectral histopathology datasets, develop larger and more generalizable models, and ultimately construct HSI-specific foundation models capable of integrating into digital pathology workflows. Standardization is another key priority. A unified framework for acquisition parameters (e.g., spectral range, band count, spatial resolution), annotation protocols, and benchmarking datasets is essential for cross-study comparability and clinical scalability. Open-access databases and multi-institutional collaborations will be critical to overcoming the limitations of small sample sizes and enabling robust validation.

Finally, as the demand for personalized nanomedicine continues to grow, HSI is poised to play a role in nanoparticle tracking, drug delivery visualization, and single-cell biosensing. These applications, when coupled with advanced machine learning and spectral unmixing algorithms, can enable molecular phenotyping, therapy stratification, and real-time drug efficacy monitoring. To achieve clinical viability, future systems should prioritize miniaturization, cost reduction, and regulatory validation, thereby paving the way for broader adoption in both bedside diagnostics and precision medicine.

## Data Availability

There is no code or data associated with this review paper.
